# Alkenyl- and Aryl-Borane Nucleophiles in Enantioselective Iridium-Catalyzed Allylic Substitution of Vinyl Epoxides

**DOI:** 10.1021/jacs.5c10680

**Published:** 2025-10-02

**Authors:** Jiangwei Wen, Lushuai Zhang, Chengyu He, Su-min Song, Jasper L. Tyler, Robert S. Paton, Varinder K. Aggarwal

**Affiliations:** † School of Chemistry, 1980 University of Bristol, Cantock’s Close, Bristol, BS8 1TS, U.K.; ‡ Department of Chemistry, 3447 Colorado State University, Fort Collins, Colorado 80528, United States

## Abstract

The catalytic asymmetric Petasis reaction represents a practical approach for synthesizing highly valuable chiral amine building blocks. However, despite the potential that this reactivity provides, the extension of the mechanistic framework to alternative electrophilic fragments is noticeably underdeveloped. We report herein the first Ir-catalyzed allylation of alkenyl, aryl, and alkynyl boranes with racemic vinyl epoxides or vinyl aziridines via an enantioselective 1,4-boronate rearrangement. Mechanistic studies reveal that the high levels of stereoselectivity arise due to tandem dynamic kinetic resolution and kinetic resolution processes, with computational analysis suggesting that a stabilizing interaction between the alkenyl boronate π-system and the electrophile facilitates the key transition state. The utility of this methodology is demonstrated in a concise, enantioselective two-step synthesis of the phytotoxin (*R*)-pyricuol.

## Graphical abstract

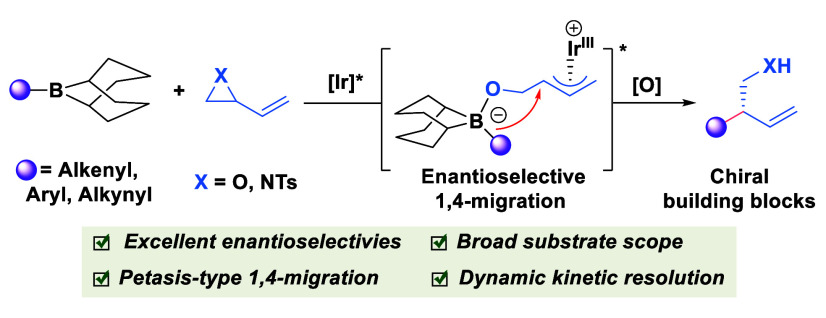

The Petasis reaction is a versatile multicomponent coupling of alkenyl or aryl boronic acids, amines, and carbonyl compounds to generate substituted amines.^1,2^ Its broad scope and high stereoselectivity with chiral catalysts have made it a popular method for synthesizing bioactive molecules, including amino acids, pharmaceuticals, and natural products.^3,4^ The mechanism of this reaction typically involves the formation of an iminium ion and a boronate complex within the same molecule, followed by migration of the boron substituent to the electrophilic iminium ion (Scheme 1A).^5^ Despite over three decades of investigation, few studies have extended this basic mechanistic framework to alternative electrophilic fragments for constructing new classes of chiral building blocks.^6^ We questioned whether a nucleophilic boronate complex could be efficiently generated adjacent to an electrophilic π-allyl complex to enable C–C bond formation with high stereoselectivity.^7,8^ It was hypothesized that such a system could be constructed by reacting a vinyl epoxide with a chiral iridium catalyst in the presence of a Lewis acidic boron reagent (Scheme 1B).^9^ After formation of the π-allyl iridium complex, the alkoxide generated would intercept the boron reagent and trigger a Petasis-type 1,4-migration to furnish the enantioenriched product.^6,10^

**Scheme 1:**
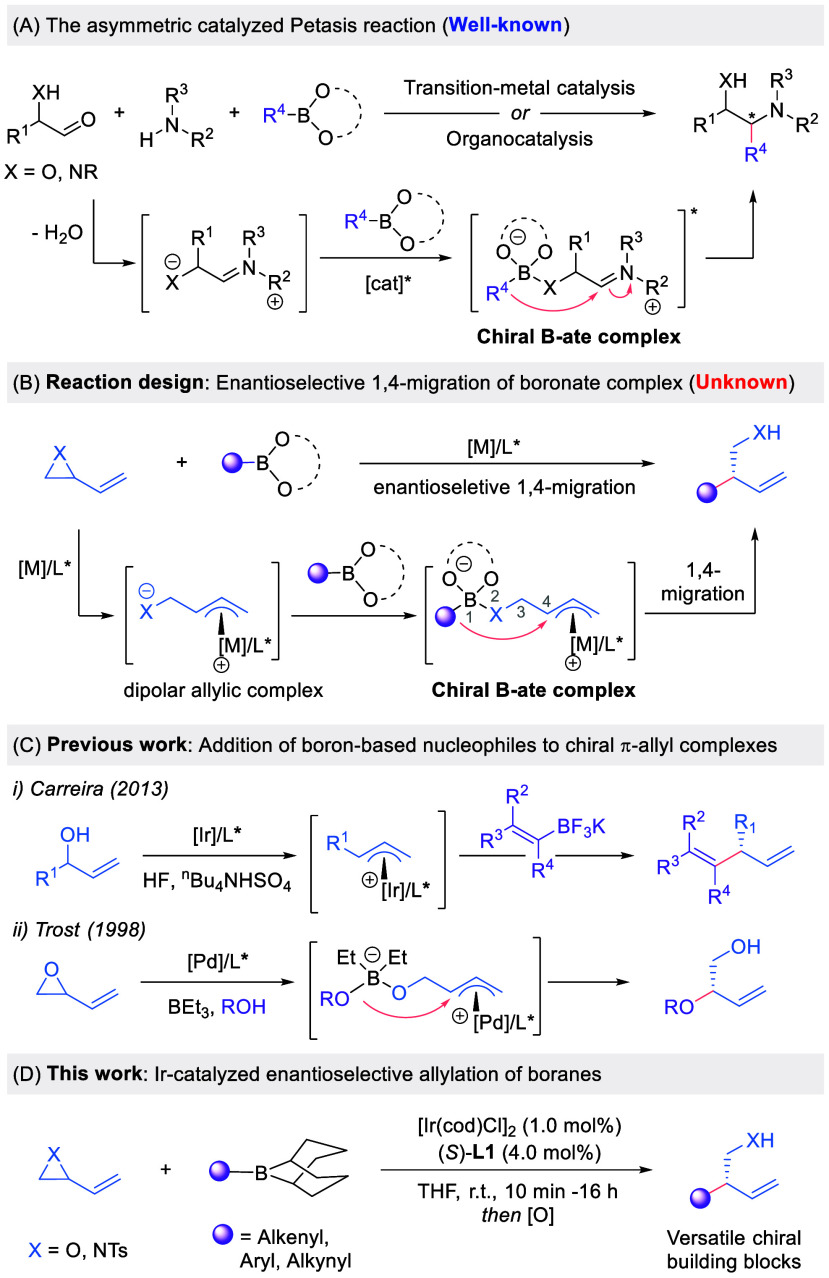
Catalytic Asymmetric Petasis Reaction, Previous Work, and Our Reaction Design

Precedent from Carreira has demonstrated that vinyl trifluoroborates can participate in nucleophilic addition to chiral π-allyl iridium complexes (Scheme 1C,i).^11d^ Rather than engaging preactivated nucleophilic boron species, we considered the simultaneous activation of both the electrophile and the nucleophile (Scheme 1B). Trost used related alkenyl epoxide-derived π-allyl palladium complexes and in situ generated borinic esters to promote the addition of alcohols (Scheme 1C,ii),^12^ while we are proposing to transfer carbon-based nucleophiles. In this Letter, we describe our success in achieving this goal, thereby expanding the generality of the Petasis reaction to a new manifold (Scheme 1D).

Our studies began with the reaction between vinyl epoxide **3a** and either (*E*)-styrylboronic acid or the analogous pinacol boronic ester, in the presence of [Ir­(cod)­Cl]_2_ and chiral ligand (*S*)-**L1**.^11^ Unfortunately, no reaction occurred, and starting materials were returned. We reasoned that the intermediate boronate was insufficiently nucleophilic to react with the π-allyl iridium complex. In order to increase its nucleophilicity, we considered employing boranes instead. Thus, vinyl borane **2a**, synthesized without isolation from phenylacetylene **1** and 9H-BBN^13^ was treated with racemic vinyl epoxide **3a** in the presence of [Ir­(cod)­Cl]_2_ and chiral ligand (*S*)-**L1**. In this instance, the desired product was obtained in 75% yield and 85% enantiomeric excess (ee) with full retention of the *E* geometry of the alkenyl borane. Notably, similar motifs are accessible via Krische’s complementary Ir-catalyzed coupling of allylic acetates and formaldehyde.^14^

After screening various reaction parameters, homoallylic alcohol **4** was generated in 85% yield and 94% ee under the optimized conditions (Table 1, entry 1, see SI for full optimization results). Interestingly, both lowering and increasing the temperature led to lower enantioselectivities (Table 1, entries 2–4), making room temperature the “Goldilocks temperature”. Higher concentrations adversely affected the enantioselectivity (entries 5 and 6), while decreasing the stoichiometry of vinyl epoxide (**3a**) decreased both the yield and the enantioselectivity of the product (entry 7). Notably, the desired product **4** was obtained in 63% yield and 86% ee when 1.0 equiv of vinyl epoxide was used, indicating that the reaction proceeds via a dynamic kinetic resolution (entry 8). Solvent effects were also examined, with THF proving to be optimal (entries 9 and 10). In the absence of the chiral ligand, racemic product **4** was obtained in 94% yield; however, the reaction did not proceed in the absence of the catalyst (entries 11 and 12).

**Table 1:**
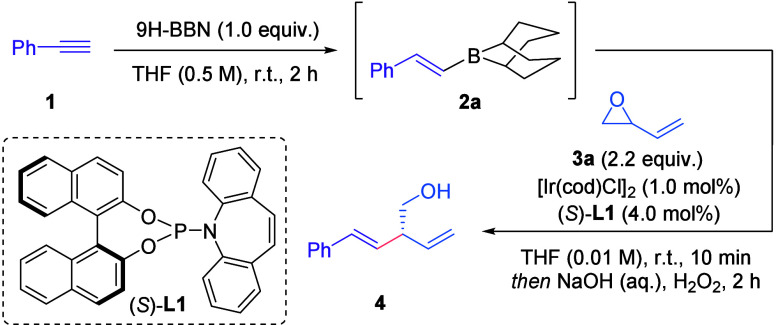
Reaction Optimization^a^

Entry	Variation	Yield 4 (%)^b^	ee (%)
1	none	85 (80)^c^	94
2	–78 °C instead of r.t.	46	48
3	0 °C instead of r.t.	72	80
4	40 °C instead of r.t.	80	77
5	0.04 M instead of 0.01 M	75	91
6	0.02 M instead of 0.01 M	80	91
7	**3a** (2.0 equiv)	70	92
8	**3a** (1.0 equiv)	63	86
9	DCM instead of THF	55	57
10	toluene instead of THF	54	72
11	without (*S*)**-L1**	94	-
12	without [Ir]	0	-

a Reaction conditions: **1** (0.20 mmol, 1.0 equiv), 9H-BBN (1.0 equiv), **3a** (2.2 equiv), [Ir­(cod)­Cl]_2_ (1.0 mol %), (*S*)-**L1** (4.0 mol %), THF (20 mL) at r.t. for 10 min, work up with NaOH (aq.), H_2_O_2_.

b ^1^H NMR yield with CH_2_Br_2_ as the internal standard.

c Isolated yield.

Having established the optimal conditions, we evaluated the scope of this enantioselective 1,4-migration cross-coupling reaction (Table 2A). We initially explored alkenyl boranes derived from the hydroboration of aryl alkynes. The reaction tolerated both electron-donating and electron-withdrawing groups at the *ortho-*, *meta*-, and *para*-positions, giving products **4**–**14** in moderate to high yields and excellent enantioselectivities (81%–97% ee). Interestingly, electron deficient aromatics (**10**–**13**) gave higher enantioselectivity compared to electron rich aromatics **5**–**8** (see SI for discussion). Alkenyl boranes derived from other arenes, including naphthalene, pyrene, and thiophene, were shown to be similarly efficient and selective (**15**–**17**). Notably, alkenyl boranes derived from the hydroboration of alkyl alkynes, including a steroid-derived alkyne, could provide the corresponding products in moderate yields with excellent stereoselectivities (**18**–**24**). This approach is also compatible with silyl-containing terminal and internal alkynes, giving the coupled products in moderate yields but with exceptional levels of stereoselectivity (**25**–**28**). Finally, coupling could be extended to the migration of alkynyl groups with (phenylethynyl)­borane and (thiophen-3-ylethynyl)­borane undergoing coupling with **3a** to generate **29a** and **29b** in high yield and good selectivity, although lower levels of ee were observed with the corresponding alkyl substituted substrates (**29c**). In common with the Petasis reaction, alkyl boranes were determined to be unreactive in this process (see below).^15^

**Table 2:**
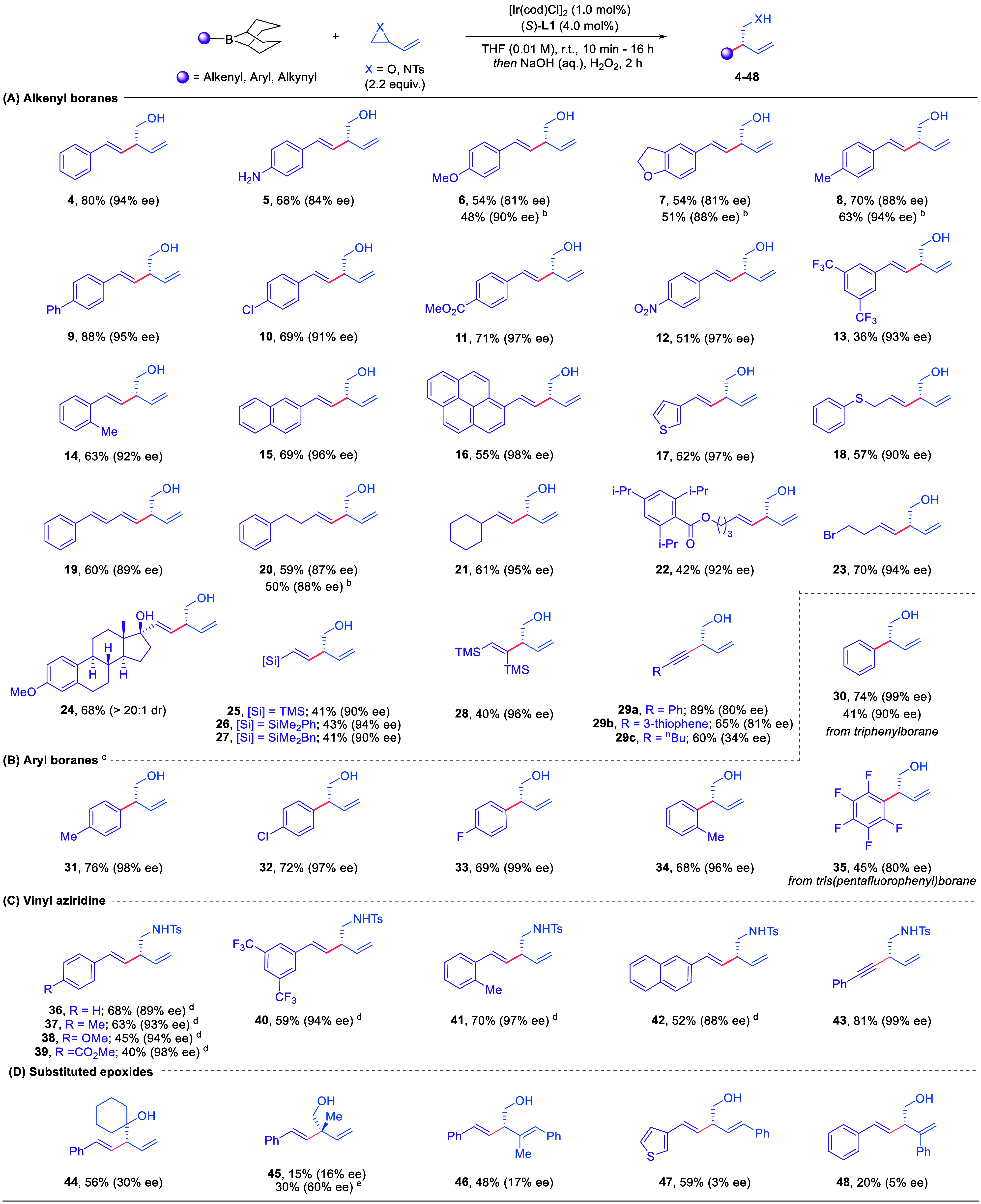
Substrate Scope^a^

a Reactions conducted on a 0.2 mmol scale. Isolated yields given; ee was determined by HPLC analysis.

b Cy_2_BH instead of 9H-BBN.

c DMF as the solvent, [Ir­(cod)­Cl]_2_ (2.0 mol %)/(*S*)-**L1** (8.0 mol %), 36 h.

d THF/toluene (0.1 M, v = 1:1) instead of THF, 18 h.

e 100 °C.

In addition to alkenyl and alkynyl boranes, aryl boranes were compatible with this system (Table 2B) and delivered the desired products in good yields with excellent enantioselectivities (**30**–**34**). It is also noteworthy that commercially available triphenyl- and tris­(pentafluorophenyl)­borane can be directly employed in this protocol, providing **30** and **35** in moderate yields and high ee.

In an attempt to improve the levels of stereoselectivity for electron-rich alkenylborane substrates, we explored the steric effect of the borane substituents. Interestingly, we found that employing more hindered dicyclohexylboranes resulted in improved ee for products derived from electron rich alkenyl boranes (**6**–**8**). We also demonstrated that this Ir-catalyzed allylation of boranes could be extended to vinyl aziridine electrophiles, with the coupled products (**36**–**43**) being delivered in 40–70% yield and uniformly high levels of enantioselectivity (Table 2C). Upon examination of substituted vinyl epoxides, it was ascertained that the desired products could be delivered in moderate yields (Table 2D, **44**–**48**). However, the enantioselectivity was found to decrease significantly for these substrates. It is hypothesized that this phenomenon arises from the slow rate of π-σ-π isomerization between the diastereomers of the intermediate boronate complex, further evidenced by the improvement in the ee of **45** observed upon heating the reaction (see below for mechanistic discussion).

Further studies were undertaken to gain a deeper understanding of the reaction mechanism. As previously noted, the equimolar reaction of vinyl epoxide **3a** with borane **2** resulted in the formation of **4** in 63% yield and 86% ee, clearly demonstrating that the transformation proceeds via a dynamic kinetic resolution, facilitated by isomerization between reactive intermediates (Table 1, entry 8).^9h^ When determining whether a kinetic resolution was occurring simultaneously, we analyzed the outcome of the equimolar reaction between borane **2** and 1-tosyl-2-vinylaziridine, whose lower volatility compared to the vinyl epoxide simplified the reisolation of the electrophile. We observed that the ee of the recovered starting material increases over time (Table S6, entries 1–4), clearly supporting the conclusion that a kinetic resolution is taking place, with one enantiomer reacting preferentially (*k*_*S*_ > *k*_*R*_). To explore the factors that most significantly impact the stereochemical outcome, we employed enantioenriched vinyl epoxide (*R*)-**3a** in combination with either (*S*)-**L1** or (*R*)-**L1** under our reaction conditions (Scheme 2A). Ligand (*S*)-**L1** provided (*R*)-**4** with 78:22 er (mismatched case), while (*R*)-**L1** gave (*S*)-**4** in 99:1 er (matched case).^16^ This shows that the chiral ligand dominates the selectivity outcome over the pre-existing stereochemistry of the epoxide and that the rate of isomerization between the π-allyl Ir complexes (*k*_isom_) is greater than the rate of 1,4 migration in the mis-matched case (*k*_mis_). When these results are taken together, a mechanism for this enantioselective transformation can be proposed (Scheme 2B). Initially, the racemic vinyl epoxide undergoes an Ir-catalyzed partial kinetic resolution ring-opening (*k*_*S*_ > *k*_*R*_) to give an alkoxide appended π-allyl Ir complex which then traps the borane reagent. The resulting boronate complex (**Int-1**) can then undergo π-σ-π isomerization (via **Int-2**) before intramolecular 1,4-migration and subsequent oxidation deliver the product. High selectivity is primarily achieved through dynamic kinetic resolution, facilitated by the isomerization between **Int-1** and **Int-1′** and the disparity between their relative rates of 1,4 migration (*k*_mat_ > *k*_mis_).

**Scheme 2:**
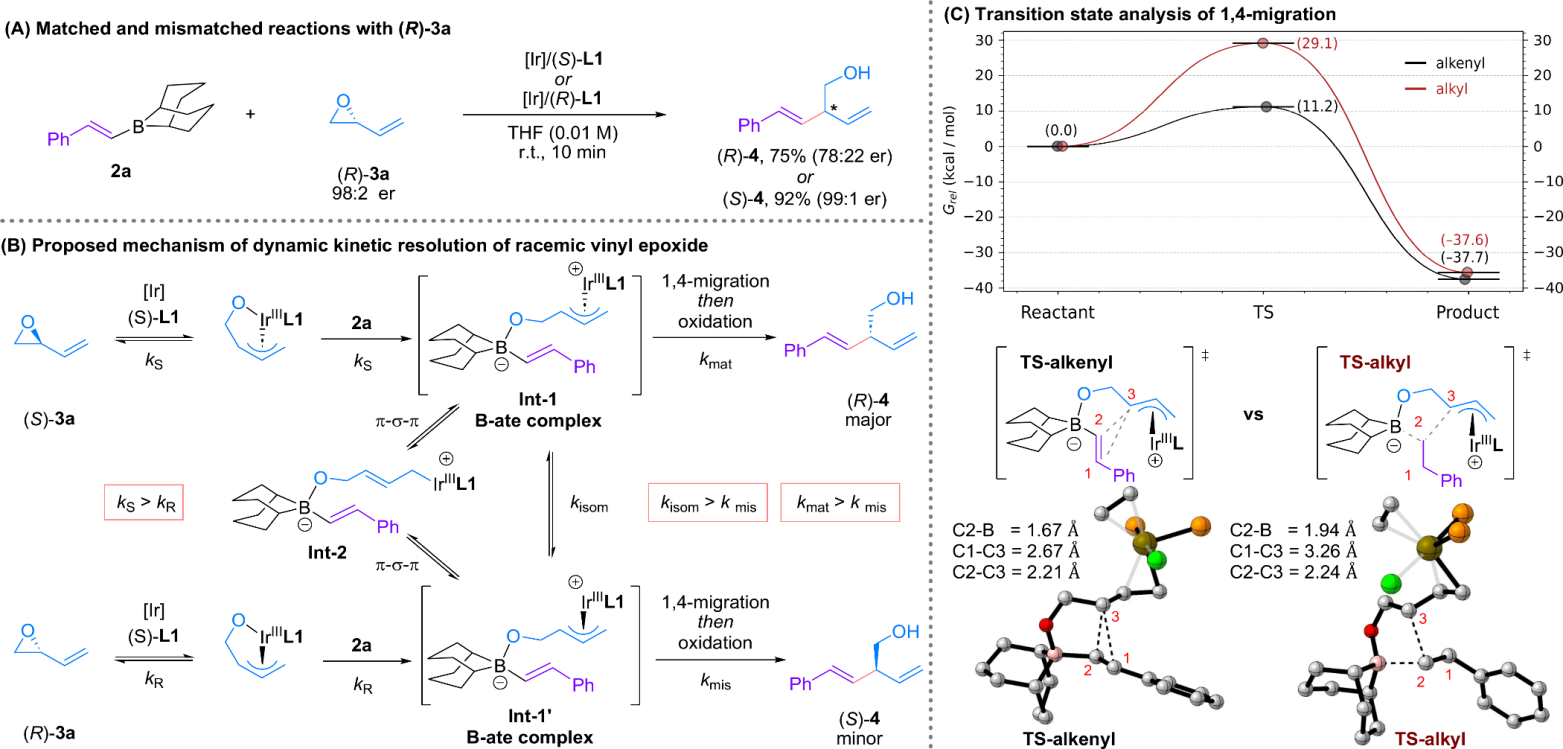
Mechanistic Studies^a^

a All reactions were conducted on a 0.2 mmol scale under the conditions shown in Table 2. Yields are of isolated products; er values were determined by HPLC analysis.

In an effort to understand some of selectivity issues that we observed during this study, we turned to computational analysis with density functional theory (DFT) (Scheme 2C).^17^ Comparing the reactions of boranes with boronic esters, we were surprised to find that the 1,4-migration transition structure (TS) energies were similar (see SI for details). These results suggest that the lack of reactivity of the boronic acid derivatives arises not from insufficient nucleophilicity of the boronate complex but from reduced Lewis acidity, which prevents formation of **Int-1** from the ring-opened π-allyl iridium complex. When modeling the 1,4-migration of alkenyl and alkyl boranes, it was found that there was a very large difference in their respective activation barriers (Δ*G* ^⧧^ = 11.2 kcal/mol vs 29.1 kcal/mol, Scheme 2C). Notably, in the TS for alkenyl migration there was a significant interaction between the C1 carbon of the migrating group and the π-allyl complex (**TS-alkenyl**). The stabilization of the TS from the alkenyl C1 carbon, as well as the later transition state and poorly aligned bond angles of the C2–B bond in relation to the π-system of the allyl complex of **TS-alkyl**, provides a rationale for the observed desirable reactivity of alkenyl groups relative to alkyl groups. In a broader context, this observation may also offer a conceivable explanation for the limited migratory aptitude of B–C­(sp^3^) bonds in related Petasis-type transformations.^2a^

To highlight the synthetic potential of this enantioselective cross-coupling reaction, we explored both the derivatization of the products and their application in bioactive motif synthesis. The reaction was successfully scaled up, delivering product **9** in 76% yield (0.95 g) while maintaining excellent enantioselectivity, demonstrating the method’s robustness (Scheme 3A). The synthetic versatility of the product motif was demonstrated through several transformations: iodoetherification afforded compound **49** as a single diastereomer in 93% yield; selective hydroboration–oxidation of the terminal alkene furnished diol **50**; hydroformylation of the terminal alkene followed by oxidation produced lactone **51**; and cross-metathesis with vinyl-Bpin delivered alkenylboronic ester **52** in 96% yield. Finally, the newly developed transformation was applied to the concise synthesis of the phytotoxin (*R*)-pyricuol (Scheme 3B).^18^ The key diene motif was efficiently constructed via the Ir-catalyzed allylation of the propyne-derived borane, generating the desired homoallyl alcohol **54** in 63% yield and 92% ee. From this chiral fragment, the natural product could be directly accessed in 56% yield via a Heck reaction with aryl bromide **55**. Overall, our synthesis delivers (*R*)-pyricuol (**56**) in just two steps, representing a substantial efficiency improvement on the previously reported 13-step route.^19^

**Scheme 3:**
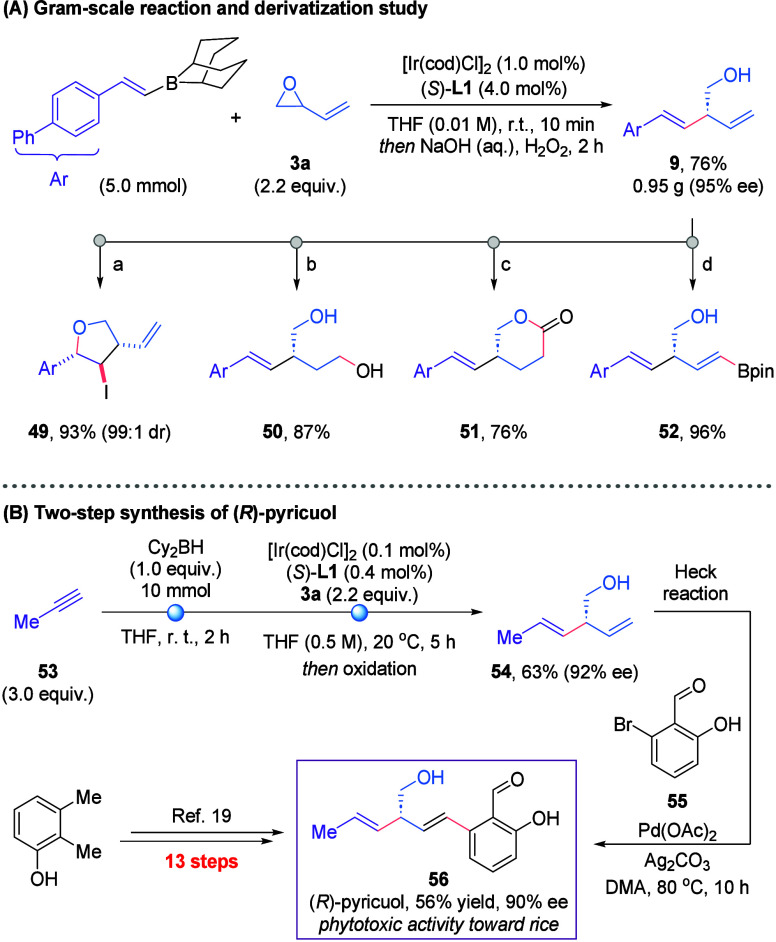
Derivatization Study and Application to the Synthesis of (*R*)-Pyricuol^a^

a (a) I_2_, NaHCO_3_. (b) 9H-BBN, then H_2_O_2_, NaOH. (c) Rh_2_(OAc)_4_, PPh_3_, CO/H_2_ (25 bar), then PCC, AcONa. (d) Vinyl-Bpin, Grubbs II.

In conclusion, we have developed an iridium-catalyzed, enantioselective borane allylation reaction that proceeds via a combination of kinetic and dynamic kinetic resolution. This transformation affords enantioenriched homoallyl alcohols bearing synthetically useful alkenyl and primary alcohol handles, as was highlighted by the two-step enantioselective synthesis of (*R*)-pyricuol. Mechanistic studies suggest that a key stabilizing interaction between the alkenyl boronate π-system and the electrophile plays a crucial role in transition state stabilization, offering a plausible explanation for the limited migratory aptitude of B–C­(sp^3^) bonds in related Petasis-type transformations.

## Supplementary Material



## Notes

Experimental procedures, materials, methods, characterization data, NMR spectra, and HPLC traces for new compounds (PDF)

## #

Key Laboratory of Green Natural Products and Pharmaceutical Intermediates in Colleges and Universities of Shandong Province, School of Chemistry and Chemical Engineering, Qufu Normal University, Qufu, 273165, Shandong, China

## $

Precise Synthesis and Function Development Key Laboratory of Sichuan Province, College of Chemistry and Chemical Engineering, China West Normal University, No. 1, Shida Road, Nanchong 637002, China.
