# Supplementary material for: Alkenyl- and Aryl-Borane Nucleophiles in Enantioselective Iridium-Catalyzed Allylic Substitution of Vinyl Epoxides
Source: J Am Chem Soc. 2025 Oct 2;147(41):36940–6. doi: 10.1021/jacs.5c10680 (PMC12532211; doi:10.1021/jacs.5c10680)

SUPPORTING INFORMATION

Alkenyl- and Aryl-Borane Nucleophiles in Enantioselective Iridium-Catalyzed Allylic Substitution of Vinyl Epoxides

Jiangwei Wen,[‡] Lushuai Zhang,[‡] Chengyu He,[‡] Su-Min Song, Jasper L. Tyler, Robert S. Paton,* Varinder K. Aggarwal*

School of Chemistry, University of Bristol, Cantock's Close, Bristol, BS8 1TS, U.K.

Correspondence to: v.aggarwal@bristol.ac.uk

TABLE OF CONTENTS

1. Materials and General Methods 4
1.1. Glassware, Solvents and Reagents 4
1.2. Instrumentation 4
1.3. Naming of Compounds 4
2. Experimental Data 4
2.1 Starting Material Synthesis 5
2.1.1 Synthesis of (*E*)-2-styrylepoxide (3b) 5
2.1.2 Synthesis of (*E*)-2-(1-phenylprop-1-en-2-yl)epoxide (3c) 5
2.1.3 Synthesis of 2-(1-phenylvinyl) epoxide (3d) 6
2.1.4 Synthesis of 2-vinyl-1-oxaspiro[2.5]octane (3e) 7
2.1.5 Synthesis of 1-tosyl-2-vinylaziridine (3f) 9
2.1.6 Synthesis of (*R*)-butadiene monoxide (*R*)-3a 9
2.1.7 Synthesis of 1-chloro-4-ethynylbenzene 10
2.1.8 Synthesis of 5-ethynyl-2,3-dihydrobenzofuran 11
2.1.9 Synthesis of benzyl(ethynyl)dimethylsilane 11
2.1.10 Synthesis of aryl boranes 12
2.1.11 Synthesis of 9-(phenylethynyl)-9-borabicyclo[3.3.1]nonane (29') 12
2.2 Optimization of Reaction Conditions 13
Table S1. Optimization of reaction solvent [a] 13
Table S2. Optimization of stoichiometry and concentration [a] 14
Table S3. Optimization of aryl migration conditions [a] 14
Table S4. Optimization of conditions for vinyl aziridine [a] 15
Table S5. Optimization of alkynyl migration conditions [a] 15
2.3 General Procedures 16
2.3.1 General procedure I 16

2.3.2 General procedure II 16
2.4 Mechanism Study 17
2.4.1 Investigation of the kinetic resolution of vinyl aziridine 17
Table S6. Investigation of the kinetic resolution of vinyl aziridine [a] 17
2.4.2 HPLC analysis for reactions with enantioenriched (*R*)-3a 19
2.4.3 Electronic effect of alkenyl borane on enantioselectivity 21
2.4.4 Unsuccessful employment of boronic esters 21
2.5 Computational details 23
2.5.1. General information 23
2.5.2. Transition structures for the 1,4-migration 24
2.5.3. Thermochemical data 30
Table S7. Compiled thermochemical data in atomic units for all structures 31
2.5.4. XYZ coordinates 32
2.6. Characterization Data for Products 58
2.7 Synthetic Applications 89
Table S8. Optimization of the synthesis of 54 [a] 92
3. References 95
4. NMR Spectra 97

1. MATERIALS AND GENERAL METHODS

1.1. Glassware, Solvents and Reagents

All manipulations were performed with oven-dried (130 °C for a minimum of 12 h) or flame-dried glassware using standard Schlenk techniques under an atmosphere of nitrogen, unless otherwise stated. All anhydrous solvents were commercially supplied or dried using an Anhydrous Engineering alumina column drying system (dichloromethane, toluene, diethyl ether, and tetrahydrofuran). Reagents were purchased from commercial sources and used as received. **(*S*)-L1** refers to 5-((11b*S*)-dinaphtho[2,1-*d*:1',2'-*f*][1,3,2]dioxaphosphepin-4-yl)-5*H*-dibenzo[*b*,*f*]azepine which was purchased from commercial sources.

1.2. Instrumentation

Thin layer chromatography (TLC) was performed using Merck Kieselgel 60 F254 fluorescent treated silica, which was visualised under UV light, or by staining with aqueous basic potassium permanganate followed by heating, or Hanessian's stain (CAM stain) followed by heating, or *p*-anisaldehyde solution followed by heating.

Flash column chromatography (FCC) was carried out using Sigma-Aldrich silica gel (60 Å, 230-400 mesh, 40-63 μm), Biotage Isolera™ flash purification system or potassium permanganate impregnated silica gel. In cases where automated column chromatography was employed, the solvent gradient and flow rate are indicated.

NMR spectra were recorded at various field strengths, as indicated, using Bruker 400 MHz, Varian VNMR 400 MHz, or Bruker Cryo 500 MHz for ^{1}H, ^{11}B, and ^{13}C acquisitions. All NMR spectra were recorded at 25 °C unless otherwise stated. Chemical shifts (δ) are reported in parts per million (ppm) and referenced to $CDCl_3$ (^{1}H: 7.26 ppm; ^{13}C: 77.16 ppm). Coupling constants (*J*) are given in Hertz (Hz) and refer to apparent multiplicities (s = singlet, d = doublet, t = triplet, q = quartet, quin = quintet, hex = hextet, h = heptet, m = multiplet, br = broad signal, dd = doublet of doublets, etc.). The ^{1}H NMR spectra are reported as follows: chemical shift (multiplicity, coupling constants, number of protons).

HPLC analyses were performed on Agilent 1100 system and Agilent 1260 Infinity II with Daicel Chiralpak columns.

High resolution mass spectra (HRMS) were recorded on a Bruker Daltonics MicrOTOF II by Electrospray Ionisation (ESI); a Thermo Scientific QExactive by Electron Ionisation (EI); a Thermo Scientific Orbitrap Elite by ESI or Atmospheric Pressure Chemical Ionisation (APCI); or a Bruker UltrafleXtreme by Matrix-assisted Laser Desorption/Ionisation (MALDI).

Gas chromatography–mass spectrometry (**GC-MS**) was recorded on an Agilent 6890 Series GC and 5973 detectors using a HP-5MS UI column (15 m × 0.25 mm × 0.25 μm).

1.3. Naming of Compounds

Compound names are those generated by ChemDraw Professional 20.0 software (PerkinElmer), following the IUPAC nomenclature.

2. EXPERIMENTAL DATA

2.1 Starting Material Synthesis

2.1.1 Synthesis of (*E*)-2-styrylepoxide (3b)

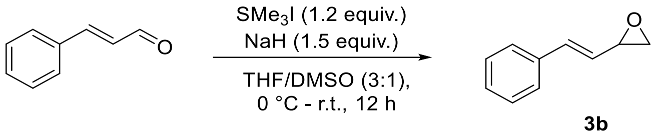

3b

According to previous methods.[1a] To an oven-dried flask was added NaH (60% dispersion in oil, 600 mg, 15 mmol, 1.5 equiv.) and washed by petroleum ether (3 × 5 mL). The solvent was then removed by vacuum and, under an N_2 atmosphere, dry THF (15 mL) and dry DMSO (5 mL) were added. Following this, a solution of SMe_3I (2.4 g, 12 mmol, 1.2 equiv.) in DMSO and cinnamaldehyde (1.3 g, 10 mmol, 1.0 equiv.) were added. The reaction was stirred at 0 °C for 30 min then r.t. for 12 h. The reaction was quenched with ice cold water (2.0 mL） and extracted with ethyl acetate (3 × 20 mL). The combined organic layers were dried with $MgSO_4$, filtered, and concentrated in vacuo. Purification by silica gel flash column chromatography (hexane/ethyl acetate/triethylamine = 91:6:3, R_f = 0.7) afforded **3b** as a yellow oil (1.2 g, 8.2 mmol, 82%).

NMR Spectroscopy (see spectra):

^{1}H NMR (400 MHz, $CDCl_3$): δ 7.42 – 7.36 (m, 2H), 7.36 – 7.29 (m, 2H), 7.27 (td, *J* = 6.6, 3.0 Hz, 1H), 6.82 (d, *J* = 16.0 Hz, 1H), 5.88 (dd, *J* = 16.0, 7.9 Hz, 1H), 3.52 (ddd, *J* = 8.1, 4.3, 2.5 Hz, 1H), 3.05 (dd, *J* = 5.3, 4.0 Hz, 1H), 2.77 (dd, *J* = 5.3, 2.7 Hz, 1H).

^{13}C NMR (101 MHz, $CDCl_3$): δ 136.2, 134.6, 128.7, 128.2, 127.1, 126.5, 52.7, 49.3.

The analytical data of the compound was in complete agreement with the literature.[1a]

2.1.2 Synthesis of (*E*)-2-(1-phenylprop-1-en-2-yl)epoxide (3c)

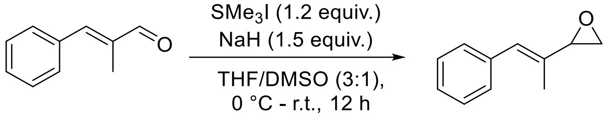

3c

According to previous methods.[1a] To an oven-dried flask was added NaH (60% dispersion in oil, 600 mg, 15 mmol, 1.5 equiv.) and washed by petroleum ether (3 x 5.0 mL). The solvent was then removed by vacuum and, under an N_2 atmosphere, dry THF (15 mL) and dry DMSO (5 mL) were added. Following this, a solution of SMe_3I (2.4 g, 12.0 mmol, 1.2 equiv.) in DMSO and α-methyl-*trans*-cinnamaldehyde (1.4 g, 10 mmol, 1.0 equiv.) were added. The reaction was stirred at 0 ℃ for 30 min then r.t. for 12 h. The reaction was quenched with ice cold water (2.0 mL) and extracted with ethyl acetate (3 × 20 mL). The combined organic layers were dried with $MgSO_4$, filtered, and concentrated in vacuo. Purification by silica gel flash column chromatography (hexane/ethyl acetate/triethylamine = 91:6:3, R_f = 0.7) afforded **3c** as a colourless oil (1.4 g, 8.8 mmol, 87%).

NMR Spectroscopy (see spectra):

^{1}H NMR (400 MHz, $CDCl_3$): δ 7.38 – 7.28 (m, 4H), 7.24 (t, *J* = 7.8 Hz, 1H), 6.68 (s, 1H), 3.50 (dd, *J* = 4.4, 2.5 Hz, 1H), 2.94 (dd, *J* = 5.3, 4.0 Hz, 1H), 2.82 (dd, *J* = 5.2, 2.8 Hz, 1H), 1.75 (d, *J* = 1.5 Hz, 3H).

^{13}C NMR (101 MHz, $CDCl_3$): δ 137.2, 134.1, 129.0, 128.9, 128.3, 126.8), 56.2, 46.9, 11.9.

The analytical data of the compound was in complete agreement with the literature.[1a]

2.1.3 Synthesis of 2-(1-phenylvinyl) epoxide (3d)

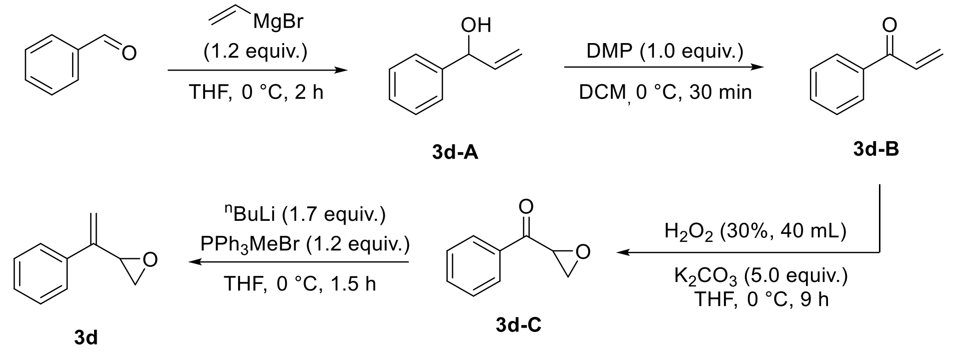

1-Phenylprop-2-en-1-ol (3d-A): According to previous methods.[1b] To an oven-dried flask was added benzaldehyde (2.1 g, 20 mmol, 1.0 equiv.) and THF (50 mL) before being cooled to 0 °C. Then, vinyl magnesium bromide (1.0 M in THF, 24 mL, 24 mmol, 1.2 equiv.) was added under a nitrogen atmosphere. After 2 h, the reaction was quenched with sat. aq. NH_4Cl solution, diluted with diethyl ether (50 mL) and the aqueous layer was separated. The aqueous layer was then extracted with ethyl acetate (3 x 150 mL) and the combined organic layers were washed with aq. sat. NaCl solution and dried over Na_2SO_4. The organic layers were concentrated in vacuo and after silica column chromatography (hexane/ethyl acetate = 91:9, R_f = 0.4), compound **3d-A** was obtained as a colourless oil (2.3 g, 17.0 mmol, 85%).

^{1}H NMR (400 MHz, $CDCl_3$): δ 7.39 – 7.32 (m, 4H), 7.28 (ddd, *J* = 8.4, 5.3, 2.8 Hz, 1H), 6.10 – 5.99 (m, 1H), 5.35 (dd, *J* = 17.0, 1.3 Hz, 1H), 5.19 (dd, *J* = 9.2, 1.1 Hz, 2H), 2.10 (s, 1H).

^{13}C NMR (101 MHz, $CDCl_3$): δ 142.6, 140.3, 128.6, 127.8, 126.4, 115.2, 75.4.

1-Phenylprop-2-en-1-one (3d-B): To an oven-dried flask was added 1-phenylprop-2-en-1-ol (**3d-A**) (2.3 g, 17 mmol, 1.0 equiv.) and DCM (50 mL) before being cooled to 0 °C. Then, DMP (7.2 g, 17 mmol, 1.0 equiv.) was added and the reaction was monitored by TLC. After 30 minutes, the reaction was diluted with water (10 mL) and the aqueous layer was separated. The aqueous layer was extracted with dichloromethane (3 x 50 mL), washed with brine and dried over Na_2SO_4. The organic layers were concentrated in vacuo and after purification by column chromatography (hexane/ethyl acetate = 95:5, R_f = 0.3), compound **3d-B** was obtained as a colourless oil (1.7 g, 13.1 mmol, 77%).

^{1}H NMR (400 MHz, $CDCl_3$): δ 7.97 – 7.90 (m, 2H), 7.60 – 7.52 (m, 1H), 7.51 – 7.43 (m, 2H), 7.15 (dd, *J* = 17.1, 10.6 Hz, 1H), 6.43 (dd, *J* = 17.2, 1.7 Hz, 1H), 5.92 (dd, *J* = 10.6, 1.7 Hz, 1H).

^{13}C NMR (101 MHz, $CDCl_3$): δ 191.1, 137.3, 133.0, 132.4, 130.3, 128.7, 128.7.

Oxiran-2-yl(phenyl)methanone (3d-C): To an oven-dried flask was added 1-phenylprop-2-en-1-one (**3d-B**) (1.70 g, 12.8 mmol, 1.00 equiv.), K_2CO_3 (8.8 g, 64 mmol, 5.0 equiv.) and THF (50 mL) before being cooled to 0 °C. Then, 30% wt aq. H_2O_2 solution (40 mL) was added slowly, and the reaction mixture was stirred for 9 h at 0 °C. After completion was confirmed by TLC analysis, the reaction was quenched with sat. aq. NH_4Cl solution, and the resulting mixture was extracted with ethyl acetate (3 x 50 mL). The combined organic layers were washed with brine and dried over Na_2SO_4. The organic layers were concentrated in vacuo and after chromatography (hexane/ethyl acetate = 91:9, R_f = 0.5), compound **3d-C** was obtained as a white solid (1.7 g, 12.3 mmol, 88%).

^{1}H NMR (400 MHz, $CDCl_3$): δ 8.05 (d, *J* = 7.0 Hz, 2H), 7.63 (t, *J* = 7.5 Hz, 1H), 7.51 (t, *J* = 7.6 Hz, 2H), 4.25 (dd, *J* = 4.5, 2.5 Hz, 1H), 3.13 (dd, *J* = 6.6, 4.4 Hz, 1H), 2.98 (dd, *J* = 6.5, 2.5 Hz, 1H).

^{13}C NMR (101 MHz, $CDCl_3$): δ 194.7, 135.4, 134.0, 128.9, 128.4, 51.2, 47.6.

2-(1-Phenylvinyl)epoxide (3d): To an oven-dried flask was added methyltriphenyl phosphonium bromide (4.8 g, 13.5 mmol, 1.2 equiv.) and THF (50 mL) before being cooled to 0 °C. *n*BuLi (1.6 M in hexane, 12.0 mL, 19.2 mmol, 1.70 equiv.) was added slowly at 0 °C and the reaction was allowed to stir at 0 °C for 1 h. At which point, 2,3-epoxy-1-(phenyl)-1-propanone **3d-C** (1.7 g, 11.3 mmol, 1.0 equiv.) was added. The reaction was monitored by TLC and after completion (ca. 30 min), the reaction mixture was quenched with water (10 mL). The resulting mixture was extracted with ethyl acetate (3 x 50 mL). The combined organic layers were washed with brine and dried over Na_2SO_4. The organic layers were concentrated in vacuo, until the solid began to precipitate out. At this point, hexane was added to further precipitate the solid. The mixture was filtered, and the remaining solvent was removed. Compound (±)-**3d** was obtained as a colourless oil (0.82 g, 5.65 mmol, 50%) after purification by chromatography (with hexane as eluent) using neutral alumina.

NMR Spectroscopy (see spectra):

^{1}H NMR (400 MHz, $CDCl_3$): δ 7.46 (dd, *J* = 8.2, 1.6 Hz, 2H), 7.37 – 7.32 (m, 3H), 5.45 (d, *J* = 1.2 Hz, 1H), 5.38 (t, *J* = 1.1 Hz, 1H), 3.68 (ddd, *J* = 4.0, 2.6, 1.1 Hz, 1H), 3.05 (dd, *J* = 6.0, 4.1 Hz, 1H), 2.63 (dd, *J* = 6.0, 2.6 Hz, 1H).

^{13}C NMR (101 MHz, $CDCl_3$): δ 144.7, 138.0, 128.8, 128.5, 128.1, 126.3, 112.7, 52.4, 49.6.

The analytical data of the compound was in complete agreement with the literature.[1b]

2.1.4 Synthesis of 2-vinyl-1-oxaspiro[2.5]octane (3e)

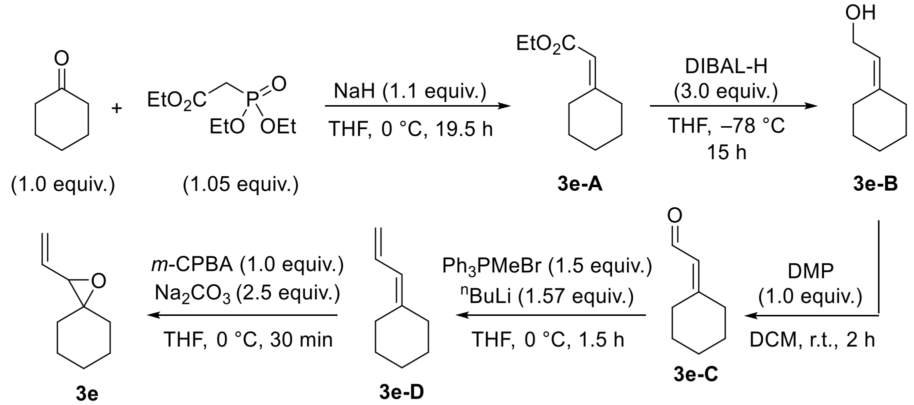

Ethyl 2-cyclohexylideneacetate(3e-A): According to previous methods.[1b] To an oven-dried flask was added sodium hydride (60% dispersion in oil, 0.89 g, 22.4 mmol, 1.1 equiv.) and THF (50 mL) before being cooled to 0 °C. A solution of triethyl phosphonoacetate (4.8 g, 4.36 mL, 21.4 mmol, 1.05 equiv.) in THF (15 ml) was then added slowly over 30 minutes at 0 °C. The reaction mixture was stirred at 0 °C for 1 h, then cyclohexanone (2.1 mL, 20.4 mmol, 1.0 equiv.) was added dropwise at the same temperature. The reaction mixture was then stirred at room temperature for 18 h. After completion, the reaction was quenched with water (10 mL) and the resulting mixture was extracted with diethyl ether (3 x 50 mL). The combined organic layers were washed with brine, dried over Na_2SO_4 and evaporated under reduced pressure. The residue was purified by silica column chromatography (hexane/ethyl acetate = 91:9, R_f = 0.7) to give compound **3e-A** as a colourless oil (3.1 g, 19.2 mmol, 94%).

^{1}H NMR (400 MHz, $CDCl_3$): δ 5.58 (s, 1H), 4.12 (q, *J* = 7.1 Hz, 2H), 2.81 (t, *J* = 5.7 Hz, 2H), 2.18 (t, *J* = 6.0 Hz, 2H), 1.66 – 1.54 (m, 6H), 1.26 (t, *J* = 7.2 Hz, 3H).

^{13}C NMR (101 MHz, $CDCl_3$): δ 166.9, 163.5, 113.0, 59.5, 38.0, 29.8, 28.6, 27.8, 26.3, 14.3.

2-Cyclohexylideneethan-1-ol (3e-B): To an oven-dried flask was added ethyl 2-cyclohexylideneacetate (**3e-A**) (3.1 g, 18 mmol, 1.0 equiv.) and dry THF (30 mL) before being cooled

to −78 °C. Then, DIBAL-H (1.0 M in DCM, 54 mL, 54 mmol, 3.0 equiv.) was added slowly at −78 °C for 3 h. After the reduction was complete (as determined by TLC analysis), the reaction was quenched with a sat. aq. solution of Rochelle's salt (10 mL). The biphasic mixture was stirred 12 h, extracted with ethyl acetate (4 x 50 mL) and dried over by Na_2SO_4. The solvent was then removed under reduced pressure. The crude alcohol was purified by silica column chromatography (hexane/ethyl acetate = 91:9, R_f = 0.4) to afford the **3e-B** as a colourless oil (2.10 g, 16.7 mmol, 93%).

^{1}H NMR (400 MHz, $CDCl_3$): δ 5.34 (t, *J* = 7.2 Hz, 1H), 4.11 (d, *J* = 8.3 Hz, 2H), 2.16 (t, *J* = 5.8 Hz, 2H), 2.09 (t, *J* = 5.9 Hz, 2H), 1.53 (s, 6H), 1.27 (s, 1H).

^{13}C NMR (101 MHz, $CDCl_3$): δ 144.5, 120.3, 58.6, 37.0, 28.9, 28.4, 27.9, 26.7.

2-Cyclohexylideneacetaldehyde (3e-C): To an oven-dried flask was added cyclohexylideneethan-1-ol (**3e-B**) (2.1 g, 16.0 mmol, 1.0 equiv.) and dry DCM (20 mL). Then, Dess-Martin periodinane (DMP) (6.8 g, 16 mmol, 1.0 equiv.) was added to the mixture at room temperature and the reaction was monitored by TLC until complete. After 2 h, water (30 mL) was added to the reaction and resulting mixture was extracted with DCM (3 x 50 mL). The combined organic layers were washed with brine, dried over Na_2SO_4 and evaporated under reduced pressure. The crude product was used directly in the next step without further purification.

Allylidenecyclohexane (3e-D): To an oven-dried flask was added methyltriphenyl phosphonium bromide (12.9 g, 36.0 mmol, 1.50 equiv.) and THF (90 mL) before being cooled to 0 °C. Then, *n*BuLi (1.60 M in hexane, 23.6 mL, 37.7 mmol, 1.57 equiv.) was added and the reaction was allowed to stir at 0 °C for 1 h. Following this, a solution of 2-cyclohexylideneacetaldehyde **3e-C** (2.98 g, 24.0 mmol, 1.0 equiv.) in THF (20 ml) was added. After 30 minutes, the reaction mixture was quenched with water, and the resulting mixture was extracted with diethyl ether (3 x 50 mL). The combined organic layers were washed with brine and dried over Na_2SO_4. The organic layers were concentrated in vacuo, until the solid began to precipitate out. At this point, hexane was added to further precipitate the solid. The mixture was filtered through celite, and the solvent was removed under reduced pressure. The crude product **3e-D** was then used in the next step without further purification.

2-vinyl-1-oxaspiro[2.5]octane (3e): To an oven-dried flask was added crude allylidenecyclohexane **3e-D** (0.88 g, 7.2 mmol, 1.0 equiv.) and dry THF (20 mL) before being cooled to 0 °C. Then, *m*-chloroperbenzoic acid (1.23 g, 7.20 mmol, 1.00 equiv.) and sodium carbonate (1.9 g, 18 mmol, 2.5 equiv.) in dichloromethane (14 ml) were added at 0 °C. The reaction mixture was stirred at 0 °C and monitored by TLC until completion. After 30 minutes, the mixture was filtered through a celite pad, washing with dichloromethane. Removal of the solvent gave crude 2-vinyl-1-oxaspiro[2.5]octane (±)-**3e**, which was further purified by flash chromatography (hexane/ethyl acetate = 95:5, R_f = 0.5) using neutral alumina to give the 2-vinyl-1-oxaspiro[2.5]octane (±)-**3e** as colourless oil (0.50 g, 3.6 mmol, 50%).

NMR Spectroscopy (<u>see spectra</u>):

^{1}H-NMR (400 MHz, $CDCl_3$): δ 5.75 (ddd, *J* = 17.4, 10.5, 7.3 Hz, 1H), 5.43 (dt, *J* = 17.3, 1.5 Hz, 1H), 5.31 (dd, *J* = 10.5, 2.4 Hz, 1H), 3.18 (d, *J* = 7.3 Hz, 1H), 1.79 – 1.64 (m, 2H), 1.58 – 1.54 (m, 5H), 1.51 – 1.43 (m, 3H).

^{13}C-NMR (101 MHz, $CDCl_3$): δ 133.2, 120.0, 64.8, 64.5, 35.4, 29.4, 25.6, 25.1, 24.8.

The analytical data of the compound was in complete agreement with the literature.[1b]

2.1.5 Synthesis of 1-tosyl-2-vinylaziridine (3f)

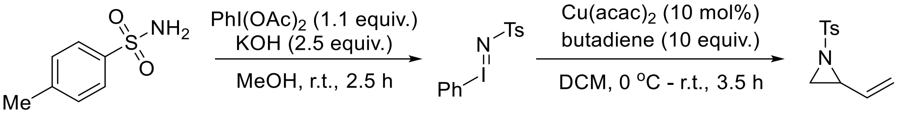

(1.0 equiv.) **3f**

Following a reported procedure.[2] An oven dried flask was charged with toluene-4-sulfonamide (5.1 g, 30 mmol, 1.0 equiv.), potassium hydroxide (4.2 g, 75 mmol, 2.5 equiv.) and methanol (45.0 mL, 0.67 M). The solution was stirred at room temperature for 10 minutes and then cooled to 0 °C in an ice bath. Once a white precipitate had fully formed, (diacetoxy)iodobenzene (10.6 g, 33 mmol, 1.1 equiv.) was added to the flask in portions over 15 min. The reaction was stirred at 0 °C for 1 h and then at room temperature for 1.5 h. After this time, the reaction mixture was poured into a flask containing water and ice, leading to formation of a precipitate. The flask was stored at 4 °C for 16 h, and the precipitate was collected by filtration, washing with water and methanol, then dried under vacuum to provide the desired product as a pale-yellow solid (7.8 g, 20.9 mmol, 70%).

An oven dried flask was charged with 4-methyl-*N*-(phenyl-λ^3-iodaneylidene)benzenesulfonamide (3.7 g, 10 mmol, 1.0 equiv.), and then purged with nitrogen before dichloromethane (20 mL) and butadiene (2.77 M in hexane, 36.1 mL, 10.0 equiv.) were added. The flask was cooled to 0 °C and copper acetylacetenoate (261 mg, 1.00 mmol, 10 mol%) was added in a single portion. The flask was stirred at 0 °C for 15 min, warmed to room temperature, and then stirred for 3 h. The reaction mixture was filtered through a pad of silica gel to remove copper salts, and the solvent was removed under reduced pressure. The crude residue was purified by column chromatography (hexane/ethyl acetate = 80:20, R_f = 0.4) to provide 1-tosyl-2-vinylaziridine **3f** as white solid (1.44 g, 6.5 mmol, 65%).

NMR Spectroscopy (see spectra):

^{1}H NMR (400 MHz, $CDCl_3$): δ 7.83 (d, *J* = 8.4 Hz, 2H), 7.34 (d, *J* = 8.3 Hz, 2H), 5.57 – 5.38 (m, 2H), 5.24 (dd, *J* = 10.2, 1.5 Hz, 1H), 3.27 (td, *J* = 7.0, 4.4 Hz, 1H), 2.78 (d, *J* = 7.2 Hz, 1H), 2.44 (s, 3H), 2.22 (d, *J* = 4.6 Hz, 1H).

^{13}C NMR (101 MHz, $CDCl_3$): δ 144.7, 135.2, 133.1, 129.8, 127.9, 120.5, 41.1, 34.3, 21.8.

The analytical data of the compound was in complete agreement with the literature.[2]

2.1.6 Synthesis of (*R*)-butadiene monoxide (*R*)-3a

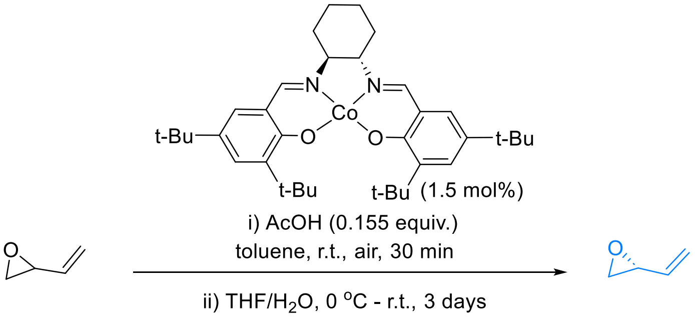

3a
(1.0 equiv.) **(*R*)-3a**

The synthesis and ee determination of ***(R)*-3a** was performed according to a previous report.[3] To a solution of (*R*,*R*)-(-)-N,N'-bis(3,5-di-tert-butylsalicylidene)-1,2-cyclohexane-diaminocobalt (0.181 g, 0.30 mmol, 0.015 equiv.) in toluene (1.5 mL) was added acetic acid (0.178 mL, 3.1 mmol, 0.155 equiv.) and the mixture was stirred at r.t. under air for 30 min. The mixture was concentrated in vacuo to give

a dark brown residue. The residue was placed under an N_2 atmosphere and butadiene monoxide (1.61 mL, 20.0 mmol) and tetrahydrofuran (0.5 mL) were added before the resulting solution was cooled to 0 °C. Water (0.25 mL, 14.0 mmol) was added dropwise, and the mixture was allowed to slowly warm to r.t. and then stirred for 3 days. Distillation of the reaction mixture (r.t., 10 mmHg, −78 °C collection vessel) afforded a colourless liquid (931 mg) that was found by ^{1}H NMR to comprise of (*R*)-butadiene monoxide **(*R*)-3a** (0.543 g, 7.70 mmol) and tetrahydrofuran (0.388 g, 5.39 mmol), with the molar ratio 1.42:1.

Determination of enantiomeric excess of (*R*)-butadiene monoxide (*R*)-3a

To a solution of 2-napthalenethiol (8.0 mg, 0.05 mmol) in methanol (0.5 mL) at 0 °C was added triethylamine (7.0 µL, 0.05 mmol) followed by a solution of (*R*)-butadiene monoxide **(*R*)-3a** (~3.0 mg, ~0.04 mmol) in $CDCl_3$ (1.0 mL). The resulting mixture was stirred at 0 °C for 17 h. The pure product was subjected to flash chromatography (hexane/ethyl acetate = 75:25, R_f = 0.5) to give the targeted ring-opened products with 96% ee.

^{1}H NMR (400 MHz, $CDCl_3$) δ 7.85 (d, *J* = 2.0 Hz, 1H), 7.82 – 7.72 (m, 3H), 7.53 – 7.42 (m, 3H), 5.91 (ddd, *J* = 17.3, 10.5, 5.8 Hz, 1H), 5.34 (dt, *J* = 17.1, 1.4 Hz, 1H), 5.20 (dt, *J* = 10.5, 1.3 Hz, 1H), 4.26 (dddt, *J* = 8.4, 5.5, 4.1, 1.3 Hz, 1H), 3.27 (dd, *J* = 13.7, 4.1 Hz, 1H), 3.06 (dd, *J* = 13.6, 8.4 Hz, 1H), 2.49 (s, 1H).

^{13}C NMR (101 MHz, $CDCl_3$) δ 138.5, 133.7, 132.4, 132.1, 128.8, 128.6, 128.0, 127.8, 127.2, 126.8, 126.1, 116.5, 70.6, 41.8.

The analytical data of the ring-opened products were in complete agreement with the literature.[3]

HPLC: Chiralpak OD column (250 mm), detected at 230 nm, hexane/i-propanol = 95/5, flow = 1.0 mL/min, retention time: 20.0 min (major), 29.4 min (minor). 98:2 e.r.

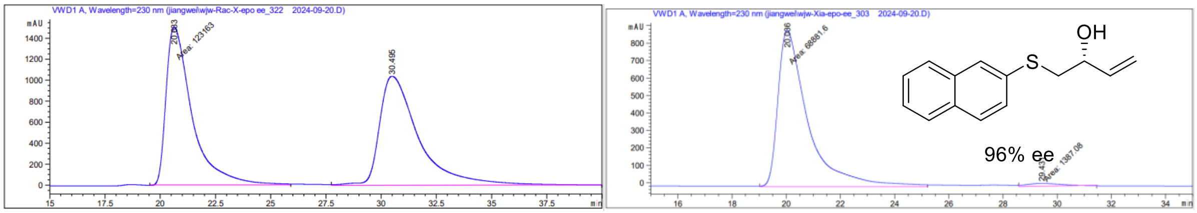

Peak #	RetTime [min]	Type	Width [min]	Area [mAU*s]	Height [mAU]	Area %
1	20.633	MM	1.3644	1.23163e5	1504.44653	48.9869
2	30.495	VBA	1.8639	1.28257e5	1040.05884	51.0131

Peak #	RetTime [min]	Type	Width [min]	Area [mAU*s]	Height [mAU]	Area %
1	20.036	MM	1.2832	6.88816e4	894.69104	98.0260
2	29.431	MM	0.9842	1387.07861	16.58244	1.9740

2.1.7 Synthesis of 1-chloro-4-ethynylbenzene

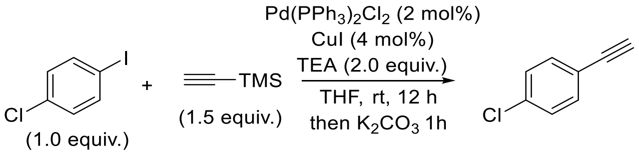

According to a previously reported procedure.[4] To a solution of 1-chloro-4-iodobenzene (1.2 g, 5 mmol, 1.0 equiv.), CuI (38 mg, 0.2 mmol, 4 mol%) and $Pd(PPh_3)_2Cl_2$(70.2 mg, 0.1 mmol, 2 mol%) in THF (20 mL) was added Et_3N (1.4 mL, 10 mmol, 2.0 equiv.), ethynyltrimethylsilane (1.0 mL, 7.5 mmol, 1.5 equiv.) dropwise. The resulting mixture was stirred at room temperature for 12 h, after the reaction was complete, the reaction was quenched with a mixture of water and ice and extracted with ethyl acetate (3×20 mL). The insoluble compounds were removed by filtration through a short pad of silica, the solvent was removed under reduced pressure and the crude (4-

chlorophenyl)ethynyl)trimethylsilane was dissolved in dichloromethane/methanol (20 mL each) and was stirred with an excess of potassium carbonate at room temperature for 1 h. After completion, the solution was filtered and concentrated in vacuo; the crude mixture was then purified by silica gel column chromatography (hexane/ethyl acetate = 80:20, R_f = 0.5) to afford the desired product as yellow solid (435 mg, 3.12 mmol, 64% yield).

NMR Spectroscopy (see spectra):

^{1}H NMR (400 MHz, $CDCl_3$) δ 7.4 – 7.3 (m, 2H,), 7.3 – 7.2 (m, 2H,), 3.1 (s, 1H,).

^{13}C NMR (101 MHz, $CDCl_3$) δ 134.9, 133.4, 128.7, 120.6, 82.5, 78.2.

The analytical data are in accordance with the literature.[4]

2.1.8 Synthesis of 5-ethynyl-2,3-dihydrobenzofuran

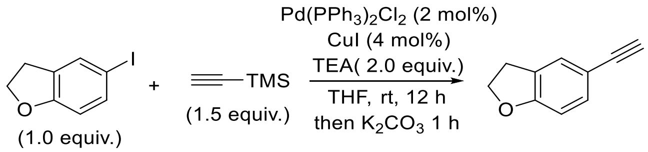

According to a previously reported procedure.[4] To a solution of 5-iodo-2,3-dihydrobenzofuran (2.4 g, 10 mmol, 1.0 equiv.), CuI (76 mg, 0.4 mmol,4 mol%) and $Pd(PPh_3)_2Cl_2$ (140 mg, 0.2 mmol, 2 mol%) in dried THF (20 mL) was added Et_3N (2.8 mL, 20 mmol, 2.0 equiv.) and ethynyltrimethylsilane (2.1 mL, 15 mmol, 1.5 equiv.) dropwise. The resulting mixture was stirred at room temperature for 12 h, then quenched with a mixture of water and ice and extracted with ethyl acetate (3×20 mL). The insoluble compounds were removed by filtration through a short pad of silica, the solvent was removed under reduced pressure and the crude product ((2,3-dihydrobenzofuran-5-yl)ethynyl)trimethylsilane was dissolved in dichloromethane/methanol (40 mL each) and was stirred with an excess of potassium carbonate at room temperature for 1 h. After completion, the solution was filtered and concentrated in vacuo; the crude mixture was then purified by silica gel column chromatography (hexane/ethyl acetate = 70:30, R_f = 0.6) to afford the desired product as yellow solid (1.06 g, 7.4 mmol, 74% yield).

NMR Spectroscopy (see spectra):

^{1}H NMR (400 MHz, $CDCl_3$) δ 7.35 – 7.19 (m, 2H), 6.69 (d, J = 8.3 Hz, 1H), 4.56 (t, J = 8.8 Hz, 2H), 3.22 – 3.10 (m, 2H), 2.94 (s, 1H).

^{13}C NMR (126 MHz, $CDCl_3$) δ 160.7, 132.8, 128.9, 127.4, 113.9, 109.5, 84.2, 75.2, 71.6, 29.4.

2.1.9 Synthesis of benzyl(ethynyl)dimethylsilane

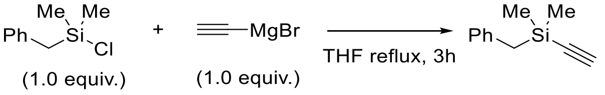

According to a previously reported procedure.[5] To a solution of Benzylchlorodimethylsilane (2.7 mL, 15 mmol, 1.0 equiv.) in dried THF (40 mL) was added 0.5 M ethynylmagnesium bromide (30 mL, 15 mmol, 1.0 equiv.) dropwise. The resulting mixture was heated to reflux. for 3 h then poured into separatory funnel containing water and ether, the separated organic phase was washed with water and brine, then dried over anhydrous Na_2SO_4, filtered and concentrated to give a yellow oil. The crude product was purified by distillation to afford the desired product as a colourless oil (1.5 g, 8.6 mmol, 57% yield)

NMR Spectroscopy (see spectra):

^{1}H NMR (400 MHz, $CDCl_3$) δ 7.28 – 7.20 (m, 2H), 7.15 – 7.06 (m, 3H), 2.43 (s, 1H), 2.24 (s, 2H), 0.17 (s, 6H).

^{13}C NMR (126 MHz, $CDCl_3$) δ 160.7, 132.8, 128.9, 127.4, 113.9, 109.5, 84.2, 75.2, 71.6, 29.4.

The analytical data are in accordance with the literature.[5]

2.1.10 Synthesis of aryl boranes

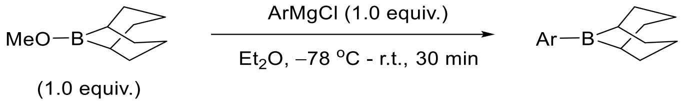

According to a previously reported procedure.[6] To a Schlenk flask inside a glovebox was added 9-methoxy-9-borabicyclo[3.3.1]nonane (0.761 g, 5.00 mmol, 1.00 equiv.) and anhydrous diethyl ether (5.0 mL). The resulting solution was cooled to −78 °C and the corresponding Grignard reagent (5.0 mmol, 1.0 equiv.) was added dropwise. The reaction mixture gradually warmed to room temperature and stirred for 30 minutes. The solvent was then removed under reduced pressure. The resulting white solid was suspended in anhydrous hexane (6.0 mL) and stirred at room temperature for 3 h, to precipitate the magnesium salts. Once the precipitate had settled, the supernatant was transferred to another Schlenk flask. The solid residue was washed with additional anhydrous hexane (10 mL) and combined with the supernatant. The combined hexane solutions were concentrated, and the remaining salts were removed using a microporous filter. The resulting colourless liquid was used for the subsequent reactions without further purification.

Note: The analogous organolithium species can also be used in this synthesis. However, a lower yield and ee value of the final product was observed when using aryl boranes derived from the corresponding organolithium rather than the Grignard (from 74% yield and 99% ee to 54% yield and 72% ee in the case of 9-phenyl-9-borabicyclo[3.3.1]nonane).

2.1.11 Synthesis of 9-(phenylethynyl)-9-borabicyclo[3.3.1]nonane (29’)

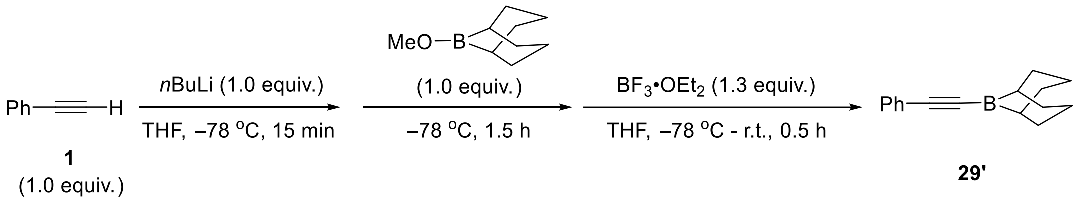

According to a modified literature procedure.[7] To a 50 mL Schlenk flask was added ethynylbenzene (1.02 g, 10.0 mmol, 1.00 equiv.) and dry THF (15 mL) before being cooled to −78 °C. *n*BuLi in hexanes (2.5 M, 4.0 mL, 10 mmol, 1.0 equiv.) was slowly added to the mixture and stirred for 15 min at −78 °C. 9-Methoxy-9-borabicyclo[3.3.1]nonane in hexanes (1.0 M, 10 mL, 10 mmol, 1.0 equiv.) was added and the mixture was stirred for 1.5 h at −78 °C. $BF_3{\cdot}OEt_2$ (1.6 mL, 13 mmol, 1.3 equiv.) was then added, after which time the reaction was further stirred at −78 °C for 0.5 h before warming up to room temperature. All volatiles were removed under vacuum, and the mixture was dissolved in dry benzene (15 mL). The suspension was then filtered via cannula filtration into another N_2-filled 50 mL Schlenk flask. The resulting solution was concentrated under vacuum, yielding the pale-yellow crude product. The Schlenk flask was then transferred into the glovebox, and the crude product was washed sequentially with pentane (3 x 10 mL). Redissolving the white solid in 30 mL of benzene separated the product from remaining $LiBF_4$ after filtration through a medium frit. The filtrate was concentrated to yield THF adduct **29′** after drying which was used for the subsequent reactions without further purification.

^{1}H NMR ($CDCl_3$, 400 MHz): δ 7.51 – 7.39 (m, 2H), 7.36 – 7.18 (m, 3H), 4.04 (s, 4H), 2.24-2.04 (m, 10H), 1.82-1.74 (m, 2H), 1.30 (s, 3H), 1.21-1.15 (m, 4H).

^{11}B NMR ($CDCl_3$, 161 MHz): δ 26.5 ppm.

2.2 Optimization of Reaction Conditions

Table S1. Optimization of reaction solvent [a]

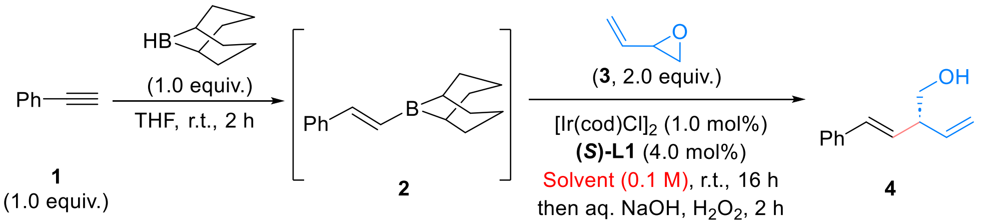

Entry	Solvent (0.1 M)	Yield of **4** (%) [b]	ee **4** (%)
1	THF	75	85
2	1,4-dioxane	50	72
3	2-MeTHF	65	85
4	Et_2O	55	80
5	MeOCy	71	74
6	DCM	71	57
7	DCE	55	67
8	EtOAc	70	76
9	CH_3CN	62	50
10	Toluene	60	72

[a] Reaction conditions: **1** (0.2 mmol, 1.0 equiv.), 9H-BBN (1.0 equiv.) **3a** (2.0 equiv.), $[Ir(cod)Cl]_2$ (1.0 mol%), **(*S*)-L1** (4.0 mol%), the THF was carefully removed using a vacuum pump, and then the corresponding solvent (2.0 mL) was added at r.t. for 16 h, work up with aq. NaOH (3.0 M, 1.0 mL), aq. H_2O_2 (30% wt, 0.3 mL). [b] NMR yield with CH_2Br_2 as internal standard.

Table S2. Optimization of stoichiometry and concentration [a]

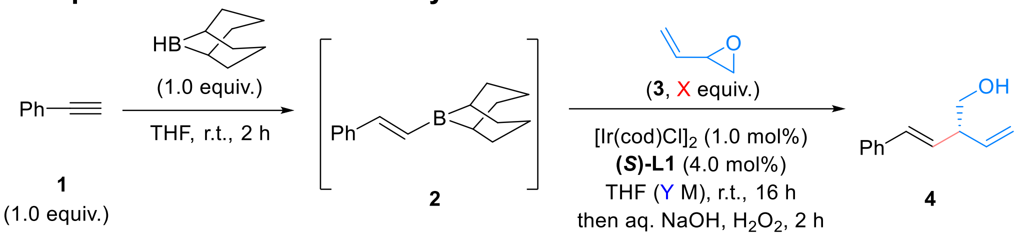

Entry	**3** (X equiv.)	THF (Y M)	Yield of **4** (%) [b]	ee **4** (%)
1	2.0	0.1	75	85
2	2.2	0.1	75	86
3	2.2	0.04	75	91
4	2.2	0.02	80	91
5	2.2	0.01	75 (70) [c]	94
6	2.0	0.01	70	92
7	3.0	0.01	76	94
8	2.2	0.01	72 [c,d]	94
9	2.2	0.01	80 [c,e]	94

[a] Reaction conditions: **1** (0.2 mmol, 1.0 equiv.), 9H-BBN (1.0 equiv.) **3a** (X equiv.), $[Ir(cod)Cl]_2$ (1.0 mol%), **(*S*)-L1** (4.0 mol%), with THF as the solvent at r.t. for 16 h, work up with aq. NaOH (3.0 M, 1.0 mL), aq. H_2O_2 (30% wt, 0.3 mL). [b] NMR yield with CH_2Br_2 as internal standard. [c] Isolated yield. [d] Reaction time was 3 h. [e] Reaction time was 10 min.

Table S3. Optimization of aryl migration conditions [a]

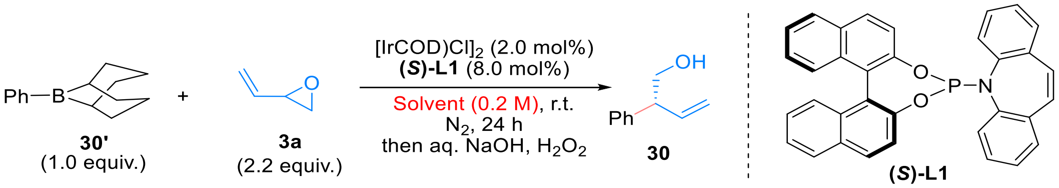

Entry	Solvent	Yield of **30** (%)	ee **30** (%)
1	THF	8	-
2	1,4-dioxane	trace	-
3	DCM	trace	-
4	CH_3CN	trace	-
5	Toluene	trace	-
6	DMF	52	99
7	DMSO	41	96
8	DMF (36 h)	80 (74) [b]	99
9	without $[Ir(cod)Cl]_2$	0	-

[a] Reaction conditions: **30′** (0.20 mmol, 1.0 equiv.), **3a** (2.2 equiv.), $[Ir(cod)Cl]_2$ (2.0 mol%), **(*S*)-L1** (8.0 mol%), solvent (0.2 M) at r.t. for 24 h, work up with aq. NaOH (3.0 M, 1.0 mL), aq. H_2O_2 (30% wt, 0.3 mL). NMR yield with CH_2Br_2 as internal standard, ee determined by chiral HPLC analysis. [b] Isolated yield.

Table S4. Optimization of conditions for vinyl aziridine [a]

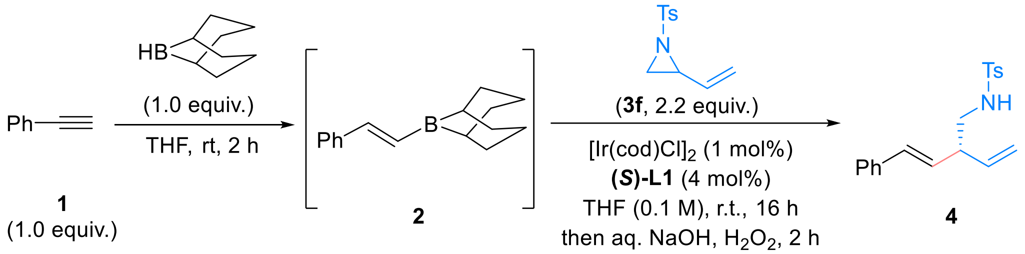

Entry	Variation	Yield of **4** (%)	ee **4** (%)
1	none	34	89
2	0.01 M	30	89
3	0.1 M	34	89
4	**3** (2.2 equiv.), THF/toluene (1:1) 0.1 M	68	89
5	without $[Ir(cod)Cl]_2$	0	-

[a] Reaction conditions: **1** (0.20 mmol, 1.0 equiv.), 9H-BBN (1.0 equiv.), **3f** (2.2 equiv.), $[Ir(cod)Cl]_2$ (1.0 mol%), **(*S*)-L1** (4.0 mol%), with THF or THF/toluene as solvent at r.t. for 16 h, work up with aq. NaOH (3.0 M, 1.0 mL), aq. H_2O_2 (30% wt, 0.3 mL). NMR yield with CH_2Br_2 as internal standard, ee determined by chiral HPLC.

Table S5. Optimization of alkynyl migration conditions [a]

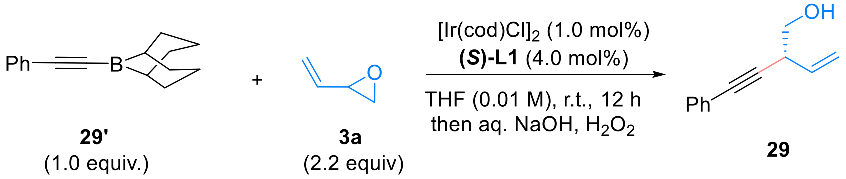

Entry	Variation	Yield of **29** (%)	ee **29** (%)
1	none	75	59
2	0.1 M	73	53
3	80 °C	77	60
4	–30 °C	78	32
5	DCM	73	10
6	EtOAc	75	26
7	acetone	77	26
8	1,4-dioxane	78	46
9	diglyme	67	40
10	2-Me-THF	77	48
11	**3** (1.5 equiv.)	74	59
12	$[Ir(cod)Cl]_2$ (5.0 mol%)/**(*S*)-L1** (20 mol%)	78	52
13	THF (0.001 M)	89	80
14	without $[Ir(cod)Cl]_2$	0	-

[a] Reaction conditions: **29'** (0.10 mmol, 1.0 equiv.), **3a** (2.2 equiv.), $[Ir(cod)Cl]_2$ (1.0 mol%), **(*S*)-L1** (4.0 mol%), THF at r.t. for 12 h, work up with aq. NaOH (3.0 M, 1.0 mL), aq. H_2O_2 (30% wt, 0.3 mL). Isolated yields given, ee determined by chiral HPLC.

2.3 General Procedures

2.3.1 General procedure I

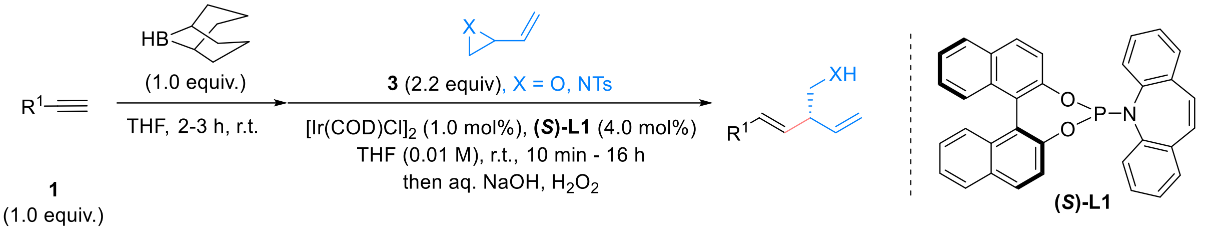

In an argon-filled glovebox, alkyne **1** (0.2 mmol, 1.0 equiv.) and 9-borabicyclo[3.3.1]nonane (9*H*-BBN, 0.5 M solution in THF, 0.4 mL, 0.2 mmol, 1.0 equiv.) were added to an oven-dried scintillation vial and capped with a rubber septum. The sealed vial was removed from the glove box and stirred for 2-3 h at room temperature. Simultaneously, $[Ir(COD)Cl]_2$ (1.3 mg, 0.002 mmol, 1.0 mol%) and (***S***)-**L1** (4.0 mg, 0.008 mmol, 4.0 mol%) were added to a separate oven-dried flask (50 mL) and capped with a rubber septum in a glovebox. Then, anhydrous THF (15 mL) and vinyl epoxide or aziridine **3** (0.44 mmol, 2.2 equiv.) were added. The in situ formed alkenyl-BBN intermediate solution was completely transferred into the flask containing catalyst and ligand, rinsing with 4.5 mL THF to ensure complete transfer. The reaction was stirred at ambient temperature until complete (10 min - 16 h). After the reaction was complete, aq. NaOH (3.0 M, 1.0 mL) and aq. H_2O_2 (30% wt, 0.3 mL) were added. After vigorous stirring for 2 h at room temperature, 10 mL of water was added, and the resulting mixture was extracted with dichloromethane (3 × 10 mL). The combined organic layers were dried with $MgSO_4$, filtered, and concentrated in vacuo. The crude product was purified by flash column chromatography on silica gel to afford the desired product.

Note: When employing Cy_2BH rather than 9*H*-BBN, the same procedure as described above is used. When 1-tosyl-2-vinylaziridine is employed instead of the vinyl epoxide, THF/toluene (0.1 M, v = 1:1) instead of THF is used as the reaction solvent.

2.3.2 General procedure II

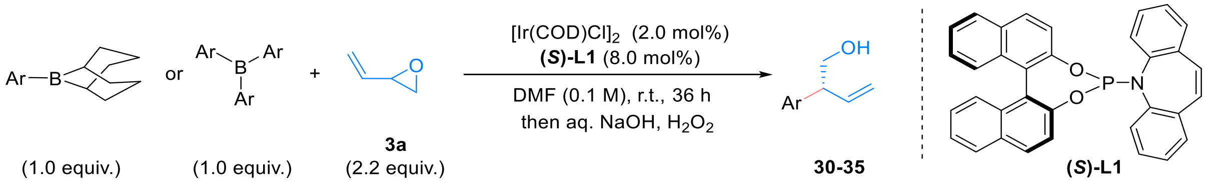

To an oven-dried 5 mL flask in an argon-filled glovebox was added $[Ir(COD)Cl]_2$ (2.6 mg, 0.004 mmol, 2 mol%) and (***S***)-**L1** (8.0 mg, 0.016 mmol, 8 mol%) and the vessel was capped with a rubber septum. Then, anhydrous DMF (2.0 mL) and vinyl epoxide or aziridine **3a** (0.44 mmol, 2.2 equiv.) were added. The corresponding borane (0.2 mmol, 1.0 equiv.) was next added into the flask. The reaction was stirred for 36 h at ambient temperature. After the reaction was complete, aq. NaOH (3.0 M, 1.0 mL) and aq. H_2O_2 (30% wt, 0.3 mL) were added. After vigorous stirring for 2 h at room temperature, 10 mL of water was added, and the resulting mixture was extracted with dichloromethane (3 × 10 mL). The combined organic layers were dried with $MgSO_4$, filtered, and concentrated in vacuo. The crude product was purified by flash column chromatography on silica gel to afford the desired product.

2.4 Mechanism Study

2.4.1 Investigation of the kinetic resolution of vinyl aziridine

Table S6. Investigation of the kinetic resolution of vinyl aziridine [a]

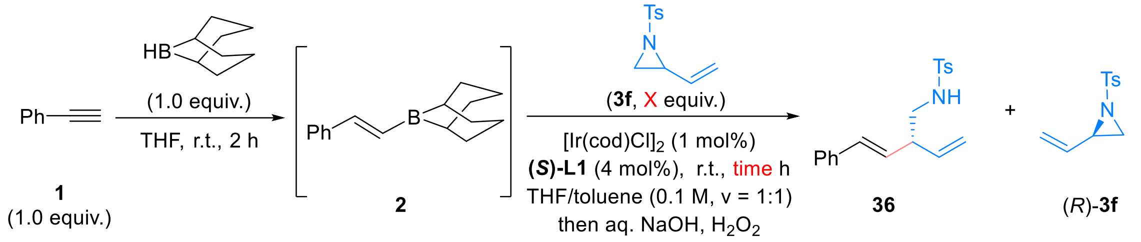

Entry	**3f** (X equiv.)	time (h)	Yield **36** (%)	ee **36** (%)	(*R*)-**3f** (recovered)
1	1.0	0.25	16	88	0.52 equiv., 68% ee
2	1.0	1	22	88	0.50 equiv., 77% ee
3	1.0	6	26	88	0.34 equiv., 95%ee
4	1.0	16	30	88	0.20 equiv., 95% ee
5	2.2	16	68	89	0.62 equiv., 95% ee

[a] Reaction conditions: **1** (0.20 mmol, 1.0 equiv.), 9H-BBN (1.0 equiv.), **3f** (X equiv.), $[Ir(cod)Cl]_2$ (1.0 mol%), **(*S*)-L1** (4.0 mol%), THF/toluene (v = 1:1, 0.1 M) at r.t. for the specified time, work up with aq. NaOH (3.0 M, 1.0 mL), aq. H_2O_2 (30% wt, 1.0 mL), NMR yield with CH_2Br_2 as internal standard, ee determined by chiral HPLC. [b] Isolated yield. **HPLC**: Chiral column AD-H, hexane: isopropanol = 97:3, flow rate = 1.0 mL/min, wavelength = 230 nm.

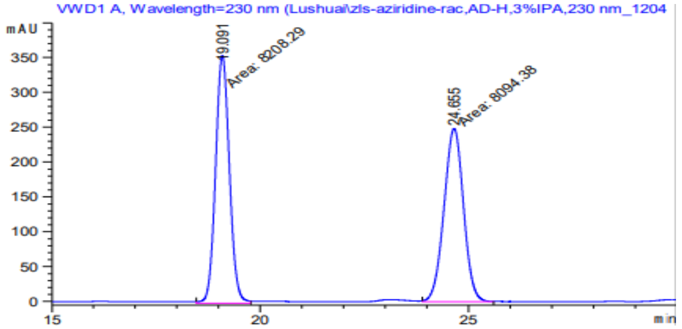

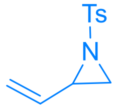

3f

>> *rac*-**3f**

Peak #	RetTime [min]	Type	Width [min]	Area [mAU*s]	Height [mAU]	Area %
1	19.091	MM	0.3852	8208.29492	355.17242	50.3494
2	24.655	MM	0.5428	8094.37988	248.53102	49.6506

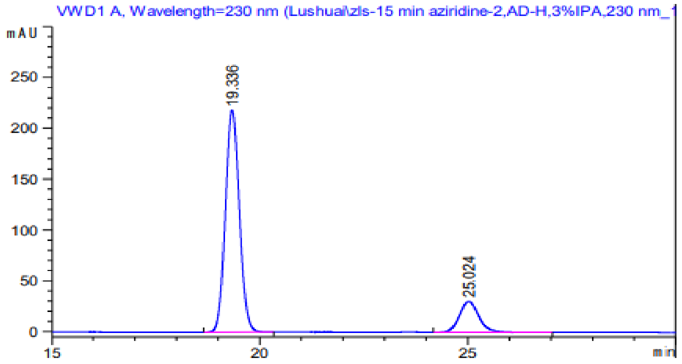

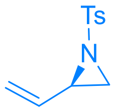

(*R*)-**3f**
68% ee

>> *0.25 h, (R)*-**3f** (recovered)

Peak #	RetTime [min]	Type	Width [min]	Area [mAU*s]	Height [mAU]	Area %
1	19.336	BB	0.3665	5116.73047	218.44547	84.0682
2	25.024	BB	0.4941	969.67657	30.19541	15.9318

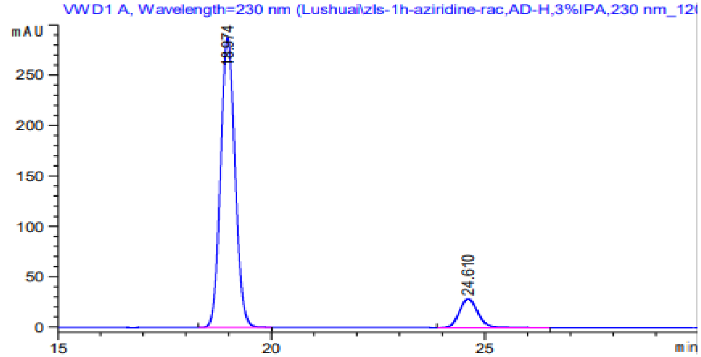

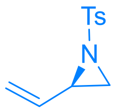

(*R*)-**3f**
77% ee

>> *1.0 h, (R)*-**3f** (recovered)

Peak #	RetTime [min]	Type	Width [min]	Area [mAU*s]	Height [mAU]	Area %
1	18.974	BB	0.3685	6790.16357	287.76831	88.7341
2	24.610	BB	0.4686	862.09186	28.56574	11.2659

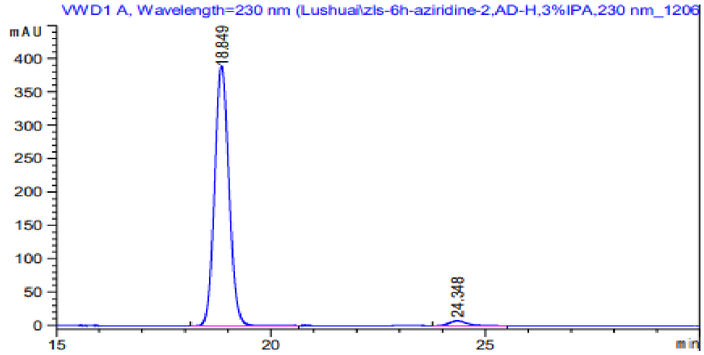

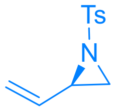

(*R*)-**3f**
95% ee

>> *6.0 h, (R)*-**3f** (recovered)

Peak #	RetTime [min]	Type	Width [min]	Area [mAU*s]	Height [mAU]	Area %
1	18.849	BB	0.3611	9029.63477	388.98984	97.8345
2	24.348	BB	0.4393	199.86028	6.97222	2.1655

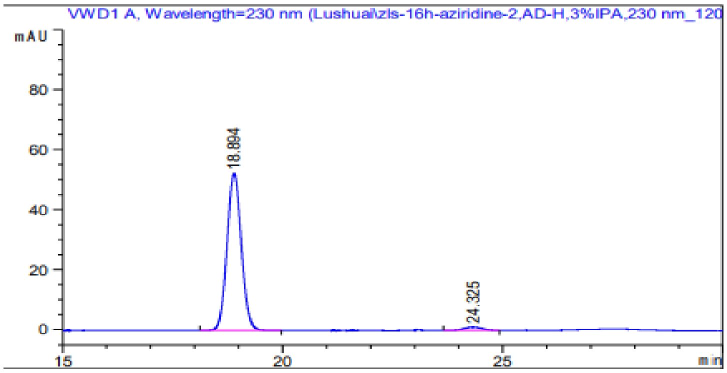

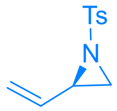

(*R*)-**3f**
95% ee

>> *16 h, (R)*-**3f** (recovered)

Peak #	RetTime [min]	Type	Width [min]	Area [mAU*s]	Height [mAU]	Area %
1	18.894	BB	0.3549	1185.84338	52.28475	97.4622
2	24.325	BB	0.4346	30.87822	1.09240	2.5378

2.4.2 HPLC analysis for reactions with enantioenriched (*R*)-3a

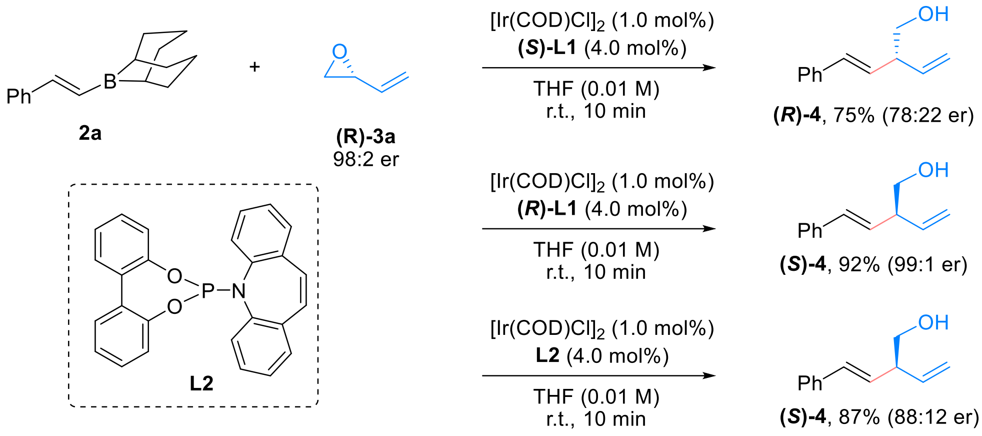

Scheme S1. Employing both enantiomers of ligand **L1** and racemic ligand **L2** to enantioenriched epoxide **(*R*)-3a**. The yield of the desired product was determined by ^{1}H NMR spectroscopy using CH_2Br_2 as an internal standard. **HPLC**: Chiral column IB, hexane: isopropanol = 95:5, flow rate = 1.0 mL/min, wavelength = 230 nm.

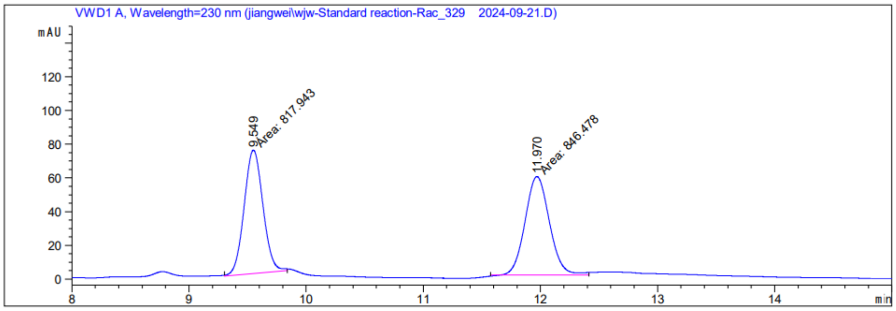

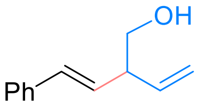

4

>> *racemic*-**4**

Peak #	RetTime [min]	Type	Width [min]	Area [mAU*s]	Height [mAU]	Area %
1	9.549	MM	0.1860	817.94263	73.28624	49.1428
2	11.970	MM	0.2417	846.47815	58.36524	50.8572

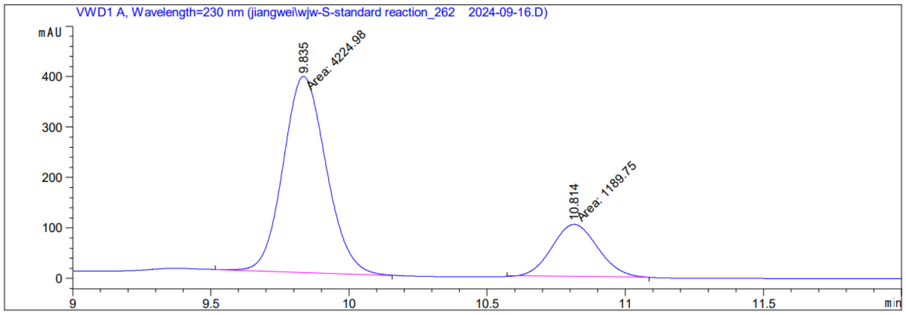

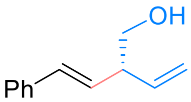

(*R*)-4
78 : 22 er

>> **(*R*)-4** from **(*S*)-L1**

Peak #	RetTime [min]	Type	Width [min]	Area [mAU*s]	Height [mAU]	Area %
1	9.835	MM	0.1809	4224.97705	389.24991	78.0276
2	10.814	MM	0.1921	1189.74512	103.24044	21.9724

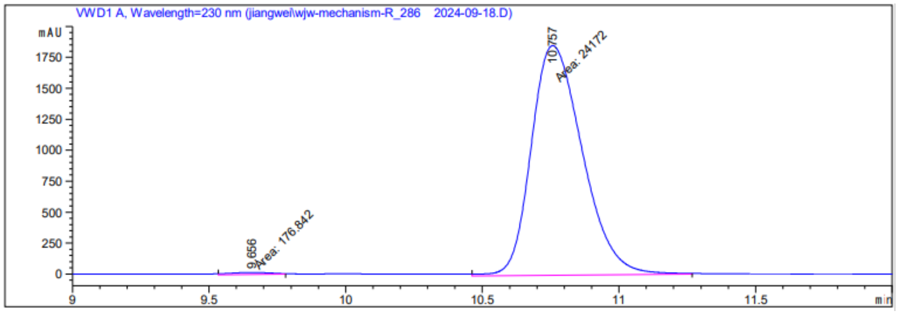

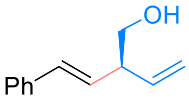

(*S*)-4
99 : 1 er

>> **(*S*)-4** from **(*R*)-L1**

Peak #	RetTime [min]	Type	Width [min]	Area [mAU*s]	Height [mAU]	Area %
1	9.656	MM	0.1737	176.84244	16.97177	0.7263
2	10.757	MM	0.2171	2.41720e4	1855.41760	99.2737

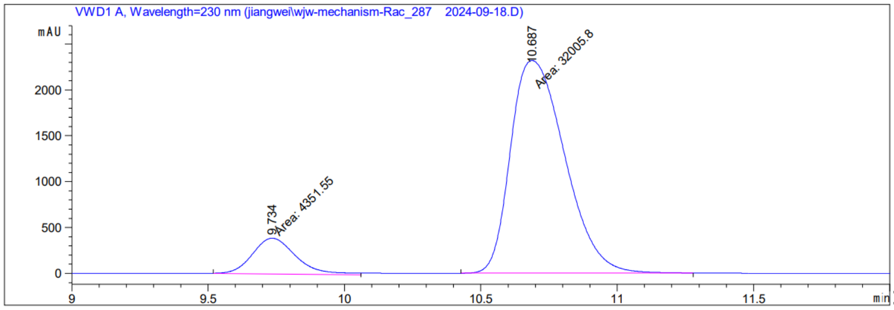

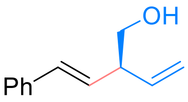

(*S*)-4
88 : 12 er

>> **(*S*)-4** from **L2**

Peak #	RetTime [min]	Type	Width [min]	Area [mAU*s]	Height [mAU]	Area %
1	9.734	MM	0.1860	4351.54834	389.99005	11.9688
2	10.687	MM	0.2303	3.20058e4	2315.92920	88.0312

2.4.3 Electronic effect of alkenyl borane on enantioselectivity

(A) Electronic effect of R on ee

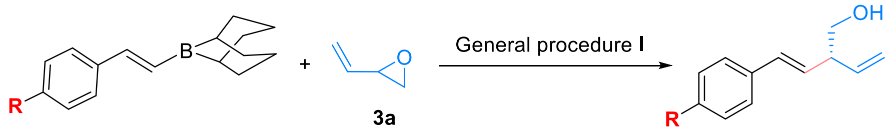

Products	R	% ee
**5**	NH_2	84
**6**	OMe	81
**8**	Me	88
**4**	H	94
**9**	Ph	96
**10**	Cl	92
**11**	CO_2Me	97
**12**	NO_2	97

(B) Hammet plot

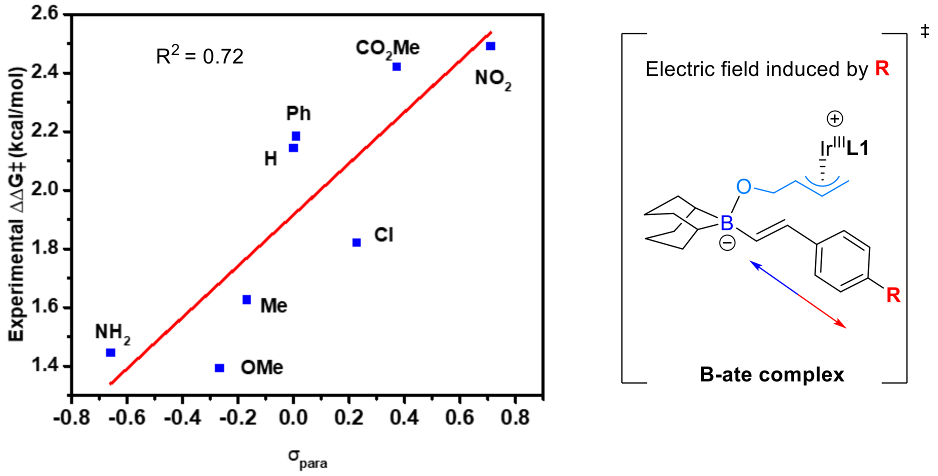

Scheme S2. Electronic effect of alkenyl borane on enantioselectivity.

We sought to glean insight into the basis for the electronic effects on ee revealed in the substrate scope study (Scheme S2A), where a higher ee was observed with substrates bearing more electron-deficient aromatic rings. The enantioselectivity data, expressed as $\Delta\Delta G^{\ddagger} = -RT\ln$ (enantiomeric ratio), was plotted against σ_{para} (Scheme S2B) and a correlation was observed. Our rationale for this observation is that electron-deficient aromatics reduce the nucleophilicity of the alkene moiety, providing more time for π-σ-π isomerization to occur, thereby leading to higher enantioselectivity via dynamic kinetic resolution. In contrast, electron-donating groups increase the nucleophilicity of the alkene moiety, resulting in shorter reaction times, incomplete π-σ-π isomerization and thus reduced enantioselectivity.

2.4.4 Unsuccessful employment of boronic esters

When attempting to employ boronic esters, the desired product was not observed and only the returned alkenyl boronic ester starting material could be detected. It was initially postulated that the intermediate boronate was insufficiently nucleophilic to react with the π-allyl iridium complex. To explore this further, we employed ^{11}B NMR studies that showed that in the presence of the epoxide and Ir catalyst system, no boronate complex formation was detected, even at prolonged reaction times (Figure S1). This observation is also consistent with the later computational results that showed that the activation energies for the 1,4-migration reactions of boranes with boronic esters were similar (see Section 2.5). These results indicate that the formation of the key π-allyl complex is not occurring with boronic ester substrates under the reaction conditions.

To better understand why boronate formation does not occur in the case of boronic ester substrates we targeted the reisolation of the vinyl epoxide from our standard reaction which is typically not observed due to its volatility. Therefore, after 6 h of reaction time, ring opening with naphthalene-2-thiol was performed and the resulting products (**5a** and **6a**) were clearly detected, confirming that the vinyl epoxide is not consumed under these conditions. Given that we employ highly coordinating solvents in our reaction system (THF, DMF, DMSO), it is unlikely that boron coordination to the epoxide is required to promote Ir activation. Therefore, it is most probable that the inability of the desired boronate complex to form in the case of boronic ester substrates is due to the lower Lewis acidity and hence inability to react with the Ir allyl complex.

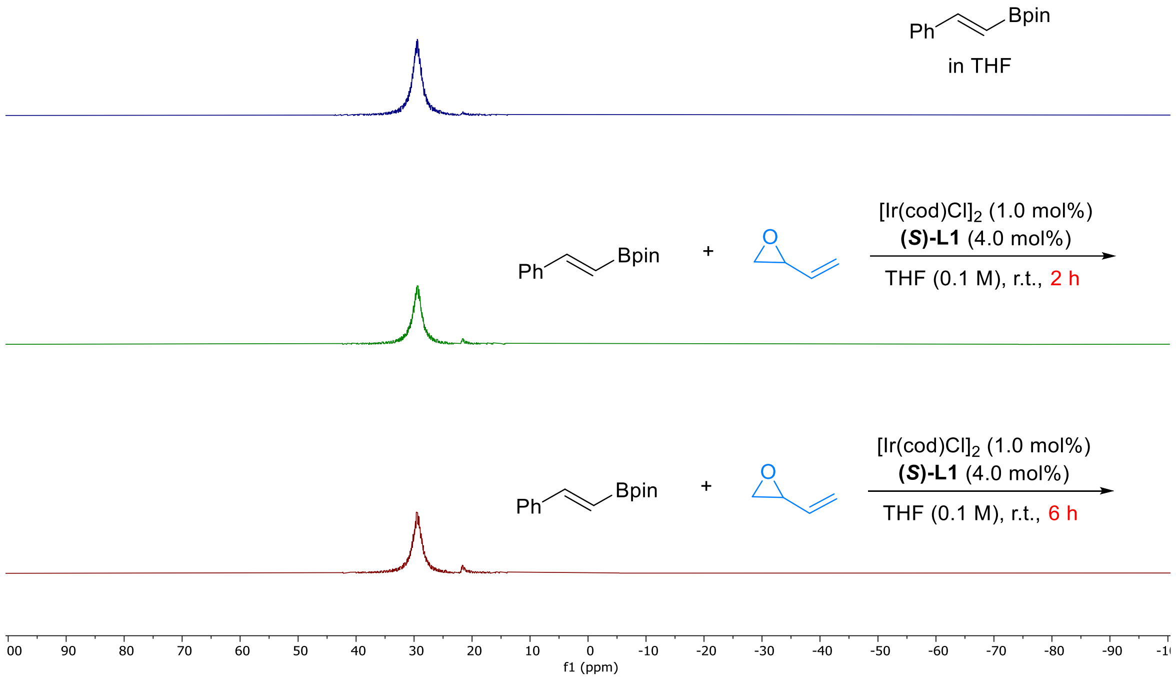

Figure S1. ^{11}B NMR study of the allylation reaction of alkenyl boronic esters.

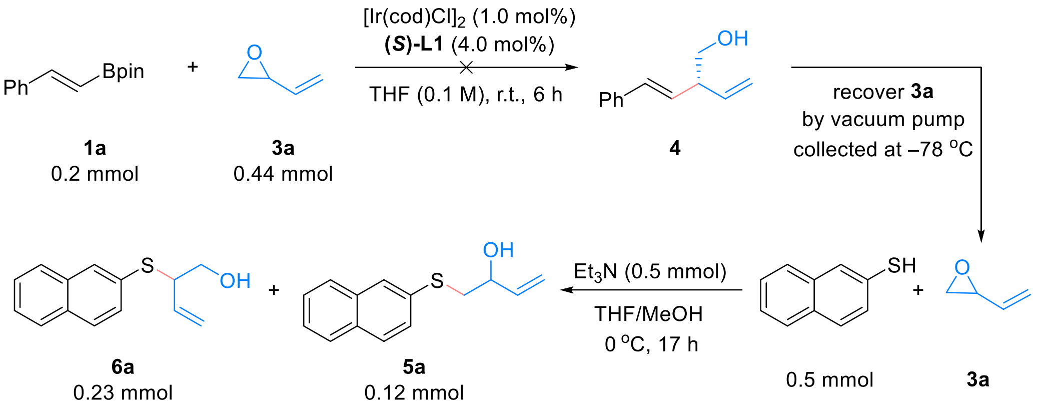

Reaction performed according to a modified general procedure **I**. After the reaction was complete reduced pressure was applied to the reaction vessel and the vinyl epoxide (**3a**) and THF were collected at –78 ˚C. To this solution was added MeOH (1.0 mL) before being cooled to 0 ˚C. To this was added naphthalene-2-thiol (80.1 mg, 0.50 mmol) and Et_3N (69.7 µL, 0.5 mmol) and stirred for 17 h. When the reaction was complete, the solvent was removed under vacuum and the adducts were obtained by column chromatography.

1-(Naphthalen-2-ylthio)but-3-en-2-ol (5a): Purification by flash column chromatography (hexane/ethyl acetate = 91:9, R_f = 0.35) gave the title compound (26.3 mg, 0.12 mmol, 26% yield w.r.t **3a**) as a colourless oil.

NMR Spectroscopy (see spectra):

^{1}H NMR (400 MHz, $CDCl_3$): δ 7.85 (d, *J* = 2.0 Hz, 1H), 7.82 – 7.72 (m, 3H), 7.53 – 7.42 (m, 3H), 5.91 (ddd, *J* = 17.3, 10.5, 5.8 Hz, 1H), 5.34 (dt, *J* = 17.1, 1.4 Hz, 1H), 5.20 (dt, *J* = 10.5, 1.3 Hz, 1H), 4.26 (dddt, *J* = 8.4, 5.5, 4.1, 1.3 Hz, 1H), 3.27 (dd, *J* = 13.7, 4.1 Hz, 1H), 3.06 (dd, *J* = 13.6, 8.4 Hz, 1H), 2.49 (s, 1H).

^{13}C NMR (101 MHz, $CDCl_3$): δ 138.5, 133.7, 132.4, 132.1, 128.8, 128.6, 128.0, 127.8, 127.2, 126.8, 126.1, 116.5, 70.6, 41.8.

2-(Naphthalen-2-ylthio)but-3-en-1-ol (6a): Purification by flash column chromatography (hexane/ethyl acetate = 91:9, R_f = 0.2) gave the title compound (52.9 mg, 0.23 mmol, 53% yield w.r.t **3a**) as a colourless oil.

NMR Spectroscopy (see spectra):

^{1}H NMR (400 MHz, $CDCl_3$): δ 7.94 (d, *J* = 1.8 Hz, 1H), 7.85 – 7.74 (m, 3H), 7.53 – 7.45 (m, 3H), 5.84 (dddd, *J* = 16.9, 10.0, 8.2, 1.6 Hz, 1H), 5.26 – 5.13 (m, 2H), 3.89 (q, *J* = 7.0 Hz, 1H), 3.81 – 3.68 (m, 2H), 2.03 (s, 1H).

^{13}C NMR (101 MHz, $CDCl_3$): δ 135.2, 132.6, 132.2, 130.3, 128.6, 127.8, 127.5, 126.7, 126.5, 118.4, 63.8, 54.7.

2.5 Computational details

2.5.1. General information

Geometry optimizations for all stationary points were carried out using the hybrid density functional B3LYP,[10] employing the LANL2DZ basis set[11] for iridium and Pople's double-ζ 6-31+G(d) basis set[12]

for all other elements. Subsequently, single-point energies were calculated at the optimized geometries using the hybrid meta-GGA functional M06[13] combined with the SDD basis set[14] for iridium and the larger Pople's triple-ζ 6-311+G(d,p) basis set for the remaining elements. For the full ligand system, geometry optimization was performed using LANL2DZ basis set[11] for all elements. To account for solvent effects, the integral equation formalism version of the polarizable continuum model (IEF-PCM) incorporating the SMD solvation method[15] was utilized, with dichloromethane specified as the solvent (solvent=dichloromethane). This computational approach was selected based on the successful validation reported by Ready and Liu[16] for the allylation reactions of vinyl boronate esters, demonstrating good agreement with experimental results. IRC calculations were performed on the transition structures (TS) for the model system to confirm their connections between local minima.[17] All density functional theory (DFT) calculations were conducted using *Gaussian 16* (revision C.01),[18] employing the "ultrafine" pruned (99,590) integration grid for accurate numerical evaluation of exchange-correlation functionals and their derivatives. Conformational analyses for stationary points were conducted manually by systematically rotating dihedral angles around rotatable single bonds and bonds in formation, thereby identifying the lowest-energy conformations for both ground and transition states. Single-point energy input files were automatically generated using the AQME program's *qprep* function.[19] Molecular visualizations were prepared with *CYLview*.[20] Vibrational frequency calculations were used to confirm the nature of stationary points (either minimal or first-order saddle points) on the potential energy surface (PES) and to compute thermal corrections to Gibbs free energies (G). Thermochemical corrections were obtained using Grimme's quasi-harmonic approximation (QHA) model[21] for entropy, applying a frequency cutoff of 100.0 cm^{-1} with the *GoodVibes* program[22] at a temperature of 298.15 K (25 °C), unless specified otherwise. Additionally, standard-state corrections (1 atm to 1 M) were applied to all calculations to account for solution-phase reaction conditions.[23]

2.5.2. Transition structures for the 1,4-migration

In this work, our computational studies focused on the origins of the 1,4 alkyl, alkenyl, and aryl migration reactivity in the boronate system. To reduce computational costs, we simplified the full catalyst system. With these models, we were able to obtain two possible TS conformations for alkyl and alkenyl group migration, respectively (Figure S2 and S3). The activation energy barrier for the 1,4 alkyl group migration was estimated to be 29.1 kcal/mol (in CH_2Cl_2), whereas for the 1,4 alkenyl group migration, it was significantly lower, 11.2 kcal/mol. It was calculated that in the transition structure (TS) for alkenyl migration, a significant interaction between both the C1 and C2 carbon of the migrating group and π-allyl complex is present, as determined by the similarity of the C1−C3 and C2−C3 bond lengths (**Alkenyl-TS**). This effect was also observed in the case of phenyl migration (Figure S4 and S5). Conversely, no such C1 proximity to the π-allyl complex is observed in the much later TS calculated for alkyl migration (**Alkyl-TS**). The stabilisation of the TS from the alkenyl C1 carbon, as well as the late TS and poorly aligned bond angles of the C2−B bond in relation to the π-system of the allyl complex, can therefore account for the significant difference in the respective activation energies ($\Delta G^{\ddagger}$ = 11.2 kcal/mol vs 29.1 kcal/mol).

Subsequently, 1,4-migration in the pinacol boronic ester system was calculated. We calculated the activation energies of alkyl, alkenyl, and phenyl groups undergoing 1,4-migration (Figure S6 and S7), which showed a similar trend to the borane system. Alkenyl migration showed the lowest barrier (12.2 kcal/mol), whereas alkyl provided the highest barrier (23.0 kcal/mol). Most significantly, the barrier to alkenyl migration in the boronic ester system is very similar to that of the borane system (12.2 kcal/mol vs 11.2 kcal/mol). As a final benchmark, we computed the key 1,4-alkyl-migration transition state with the full ligand system, optimized at a reduced basis set to manage computational cost, and compared it with the reduced model (**Alkyl-TS**). A geometrical comparison between two structures showed that the key C1–B, C1–C3, and C2–C3 bond lengths does not change significantly (Figure S8). This

consistency between the transition state geometries confirms that the simplified model efficiently captures the key environments of the full ligand system.

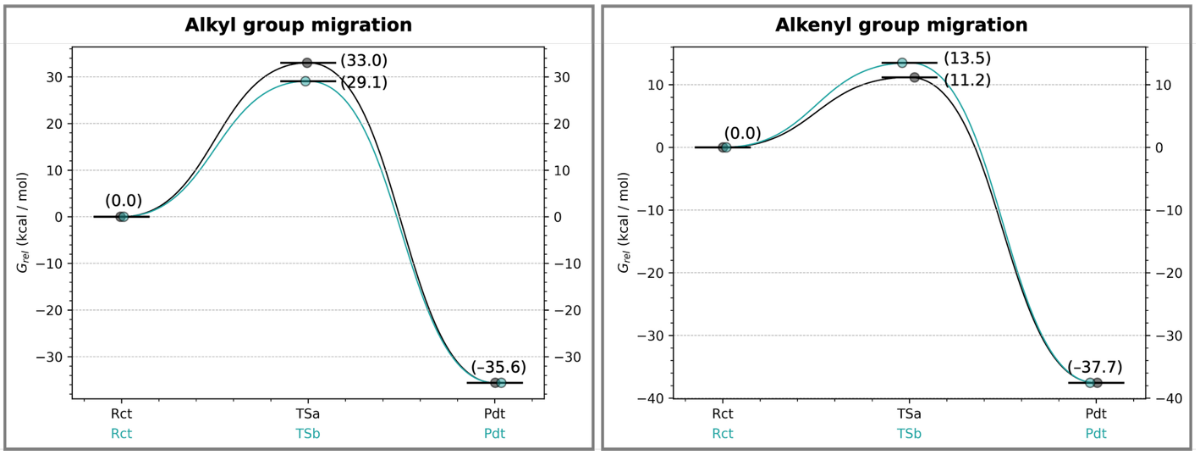

Figure S2. Gibbs free energies surface (273 K, 1 M) at the M06/6-311+G(d,p), SDD[Ir]//B3LYP/6-31+G(d), LANL2DZ [Ir] level of theory for the 1,4-migration of alkyl and alkenyl groups.

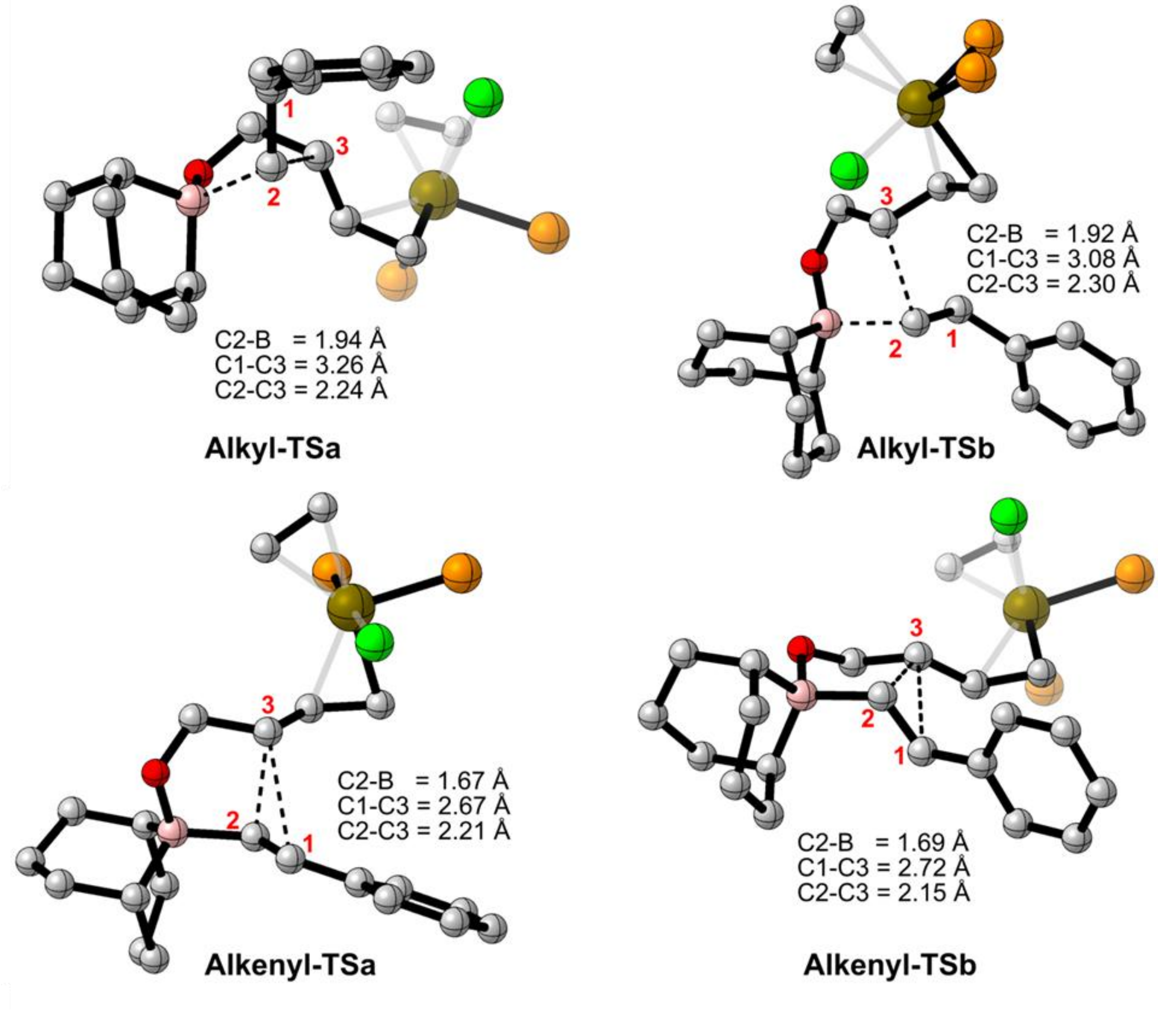

Figure S3. The DFT optimized geometry for 1,4-migration TS of alkyl and alkenyl groups.

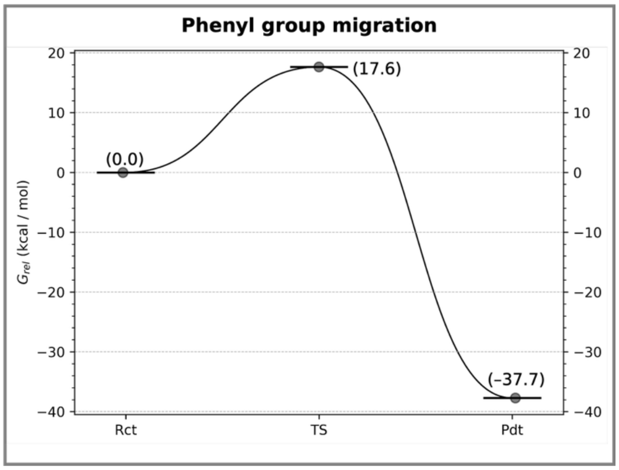

Figure S4. Gibbs free energies surface (273 K, 1 M) at the M06/6-311+G(d,p), SDD[Ir]//B3LYP/6-31+G(d), LANL2DZ [Ir] level of theory for the 1,4-migration of phenyl groups.

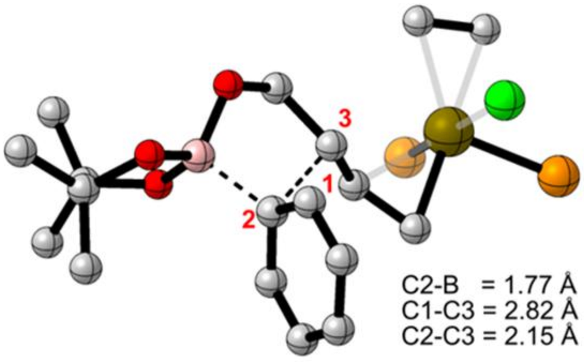

Phenyl-TS

Figure S5. The DFT optimized geometry for the 1,4-migration TS of phenyl groups.

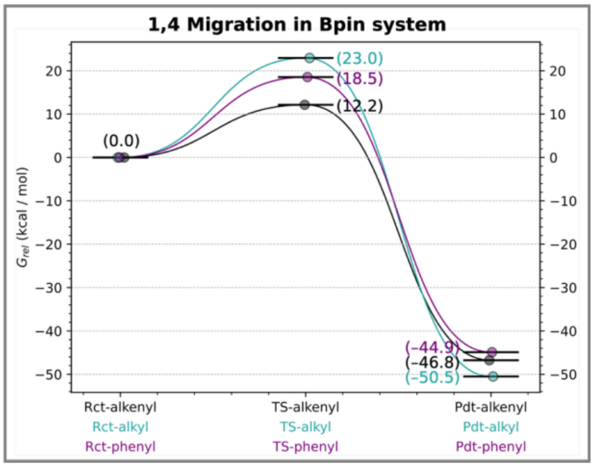

Figure S6. Gibbs free energies surface (273 K, 1 M) at the M06/6-311+G(d,p), SDD[Ir]//B3LYP/6-31+G(d), LANL2DZ [Ir] level of theory for the 1,4-migration TS of alkyl, alkenyl and phenyl groups in the boronic ester system.

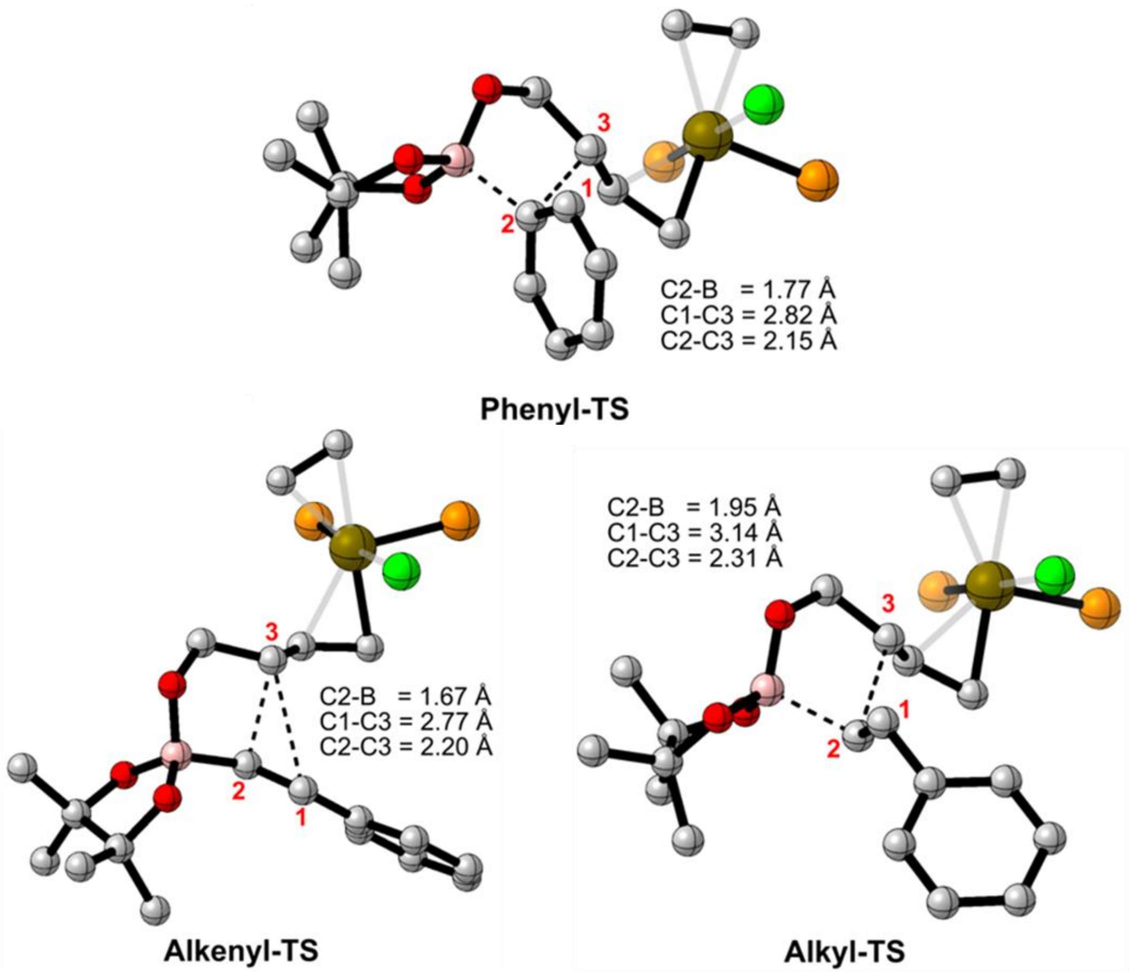

Figure S7. The DFT optimized geometry for the 1,4-migration of alkyl, alkenyl and phenyl groups in the boronic ester system.

Subsequently, we computed the 1,4 alkyl and alkenyl migration TSs with the full Carreira phosphoramidite ligands at the same level of theory as the model system (Figures S8). Conformers were generated using CREST,[22] constraining breaking and forming bonds at the values obtained for the model system: the most stable diastereomeric structures leading to opposite product enantiomers were then optimized at the DFT level of theory.

Consistent with our model studies, there is a large difference in reactivity between the alkyl and alkenyl migration propensity: the activation barrier for 1,4 alkyl migration was substantially higher (12.9 kcal/mol) than for the alkenyl case. As above, we attribute this difference to having to undergo more substantial C−B elongation and distortion (>1.9 Å for alkyl migration vs < 1.7 Å for alkenyl migration) to enable the alkyl group to interact with the allyl electrophile. Again, consistent with the model studies, there is little C−B elongation in the diastereomeric alkenyl migration TSs, and the forming C−C distances are also similar in both structures. Consistent with experiment, the (*R*)-alkenyl migration TS is energetically more favorable than the corresponding 1,4 (S)-alkenyl migration ($\Delta\Delta G^{\ddagger}$ 5.8 kcal/mol in CH_2Cl_2). In the (S)-alkenyl pathway, steric repulsion between the migrating alkenyl group and the bulky phosphine substituents of the Carreira ligand elongates the C1–C3 bond (For (R): 2.78 Å and for (S): 2.94 Å), resulting in a less favourable enantiomeric TS. In the (R)-alkenyl TS, such steric

distortion is absent, and the C1–C3 and C2–C3 contacts remain short and properly oriented toward the π-allyl fragment, thereby providing additional stabilisation. Geometrical analysis revealed that the crucial interaction between both the C1 and C2 carbons of the migrating group and the π-allyl complex is maintained in the full ligand system (**(R)- and (S)-Full-Alkenyl TSs**).

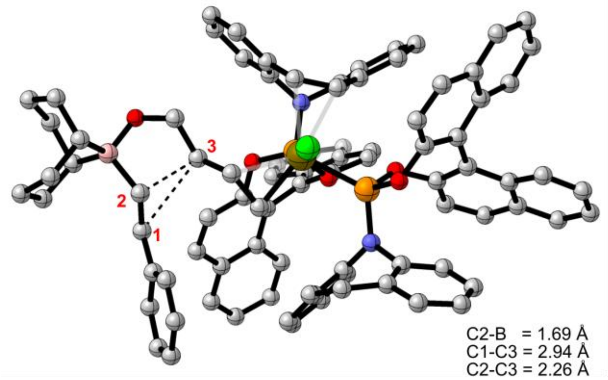

(S)-Full-Alkenyl migration TS

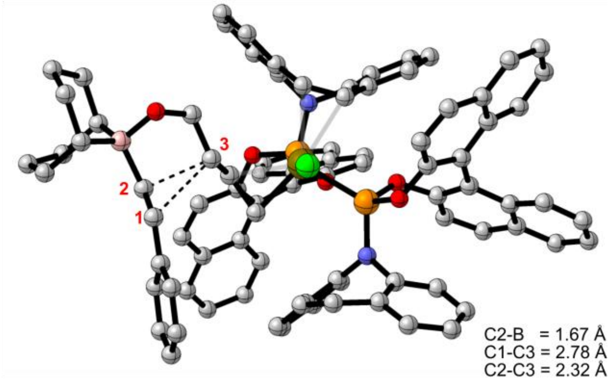

(R)-Full-Alkenyl migration TS

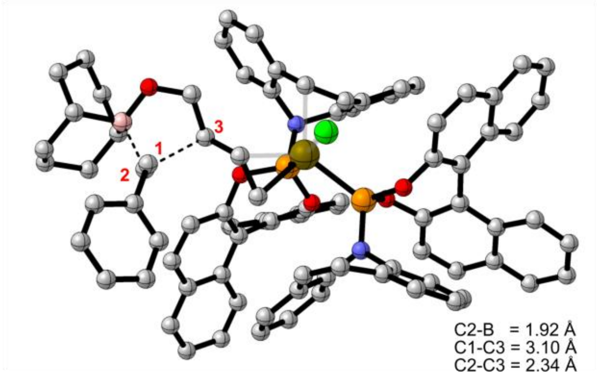

(S)-Full-Alkyl migration TS

Figure S8. The DFT optimized geometry for 1,4 migration of the alkenyl and the alkyl group with the untruncated Carreira ligand.

2.5.3. Thermochemical data

Legend:

E_{SPC} = energy obtained in the single-point energy corrections

E = energy obtained in geometry optimizations

ZPE = zero-point energy

H_{SPC} = enthalpy corrected with E_{SPC}

T·S = temperature times entropy with no correction

T·qh-S = temperature times entropy with quasi-harmonic S correction

G(T) = Gibbs free energy corrected only with E_{SPC}

qh-G(T) = Gibbs free energy with E_{SPC} and quasi-harmonic S correction

v_{imag} = imaginary frequencies

Table S7. Compiled thermochemical data in atomic units for all structures

Name	E_{SPC}	E	ZPE	H_{SPC}	T·S	T·qh-S	G(T)	qh-G(T)	ν_{imag}/cm^{-1}
Alkenyl-Rct	-2207.517658	-2208.370270	0.542675	-2206.941305	0.096675	0.090025	-2207.037980	-2207.031331	
Alkenyl-TSa	-2207.500086	-2208.353326	0.542053	-2206.925173	0.094754	0.088359	-2207.019927	-2207.013532	-276.08
Alkenyl-TSb	-2207.496732	-2208.350009	0.541841	-2206.922180	0.093286	0.087666	-2207.015466	-2207.009846	-277.46
Alkenyl-Pdt	-2207.577950	-2208.439448	0.544569	-2206.999403	0.100289	0.091791	-2207.099692	-2207.091194	
Alkyl-Rct	-2208.745382	-2209.587882	0.565945	-2208.145672	0.096420	0.089867	-2208.242092	-2208.235538	
Alkyl-TSa	-2208.691474	-2209.537547	0.564668	-2208.093269	0.096220	0.089670	-2208.189489	-2208.182939	-557.47
Alkyl-TSb	-2208.697005	-2209.543653	0.564374	-2208.098859	0.097493	0.090326	-2208.196352	-2208.189185	-524.15
Alkyl-Pdt	-2208.802534	-2209.663469	0.568343	-2208.199894	0.100947	0.092360	-2208.300841	-2208.292254	
Phenyl-Rct	-2130.157511	-2130.963323	0.509664	-2129.616548	0.090236	0.085006	-2129.706784	-2129.701555	
Phenyl-TS	-2130.128870	-2130.931746	0.508375	-2129.589828	0.088446	0.083627	-2129.678274	-2129.673456	-356.88
Phenyl-Pdt	-2130.217631	-2131.032617	0.511067	-2129.674777	0.094088	0.086891	-2129.768864	-2129.761668	
Alkyl-Rct-Bpin	-2281.884592	-2282.715498	0.536428	-2281.312380	0.100727	0.093644	-2281.413108	-2281.406025	
Alkyl-TS-Bpin	-2281.847600	-2282.675599	0.535521	-2281.276761	0.099384	0.092693	-2281.376144	-2281.369454	-537.42
Alkyl-Pdt-Bpin	-2281.965832	-2282.801757	0.539072	-2281.390729	0.104873	0.095776	-2281.495602	-2281.486505	
Alkenyl-Rct-Bpin	-2280.666159	-2281.499657	0.513138	-2280.117440	0.101086	0.093678	-2280.218526	-2280.211118	
Alkenyl-TS-Bpin	-2280.647872	-2281.483137	0.512751	-2280.100547	0.097598	0.091214	-2280.198145	-2280.191762	-268.75
Alkenyl-Pdt-Bpin	-2280.742139	-2281.577849	0.515519	-2280.190999	0.103245	0.094634	-2280.294243	-2280.28563	
Phenyl-Rct-Bpin	-2203.306985	-2204.094368	0.479794	-2202.793758	0.095942	0.089362	-2202.889700	-2202.883120	
Phenyl-TS-Bpin	-2203.278591	-2204.063431	0.479025	-2202.767095	0.091111	0.086477	-2202.858206	-2202.853572	-382.95
Phenyl-Pdt-Bpin	-2203.380934	-2204.170632	0.482461	-2202.865259	0.096487	0.089411	-2202.961746	-2202.954670	
(S)-alkenyl-full-ligand-Rct	-5152.280876	-5154.504958	1.363927	-5150.834092	0.200927	0.180675	-5151.035019	-5151.014768	
(S)-alkenyl-full-ligand-TS	-5152.273425	-5154.491877	1.364079	-5150.827528	0.196614	0.177964	-5151.024142	-5151.005492	-251.98
(S)-alkenyl-full-ligand-Pdt	-5152.350508	-5154.583207	1.366876	-5150.900487	0.204442	0.182349	-5151.104929	-5151.082836	
(R)-alkenyl-full-ligand-Rct	-5152.275717	-5154.505641	1.363079	-5150.829620	0.202351	0.181484	-5151.031971	-5151.011104	
(R)-alkenyl-full-ligand-TS	-5152.280877	-5154.501193	1.363748	-5150.835081	0.199670	0.179412	-5151.034750	-5151.014493	-192.57
(R)-alkenyl-full-ligand-Pdt	-5152.358245	-5154.594675	1.366788	-5150.908358	0.204892	0.182472	-5151.113250	-5151.090829	
(S)-alkyl-full-ligand-Rct	-5153.489509	-5155.712540	1.387053	-5152.019340	0.201381	0.181180	-5152.220721	-5152.200520	
(S)-alkyl-full-ligand-TS	-5153.469477	-5155.679282	1.386646	-5152.000068	0.199115	0.179898	-5152.199183	-5152.179966	-511.85

(S)-alkyl-full-ligand-Pdt	-5153.577890	-5155.801068	1.390965	-5152.103750	0.202716	0.181552	-5152.306466	-5152.285302	

2.5.4. XYZ coordinates

65
Alkenyl-Rct
Eopt -2208.370270

```
C    1.414328    1.268691    0.266418
H    1.194392    1.680437   -0.716329
H    1.718062    2.015267    0.998178
C   -0.961117   -1.849749    0.404163
H   -0.293520   -2.720547    0.281121
H   -1.153785   -1.755077    1.489042
O   -2.117252   -2.083318   -0.336471
C   -4.027025   -1.140153    1.227412
C   -4.338911   -1.267204   -1.355110
H   -3.376123   -0.977853    2.108736
C   -4.572091   -2.584856    1.355906
C   -5.142273   -0.064306    1.316102
H   -3.878329   -1.205768   -2.360170
C   -4.920998   -2.693717   -1.220262
C   -5.400370   -0.141193   -1.285110
H   -5.140463   -2.710263    2.295621
H   -3.710226   -3.264641    1.427123
C   -5.464602   -3.053047    0.182735
H   -4.666268    0.914315    1.454598
H   -5.764011   -0.222635    2.216562
C   -6.086427    0.031110    0.091232
H   -4.117660   -3.402142   -1.469544
H   -5.722460   -2.868755   -1.961345
H   -4.907568    0.806514   -1.550069
H   -6.187655   -0.290767   -2.045959
H   -5.594963   -4.144985    0.246288
H   -6.600037    1.005043    0.114379
H   -6.470572   -2.635310    0.304462
H   -6.882954   -0.715347    0.187692
B   -3.203525   -1.011526   -0.198826
C   -2.516642    0.450718   -0.457965
H   -2.317107    0.711592   -1.506308
C   -2.303251    1.438780    0.443859
H   -2.457307    1.214153    1.501669
C   -1.921368    2.834106    0.198063
C   -1.746154    3.698123    1.299511
C   -1.785936    3.381500   -1.096750
C   -1.464900    5.054689    1.120856
H   -1.853892    3.296651    2.305702
C   -1.504950    4.734457   -1.276528
H   -1.923184    2.745996   -1.967193
C   -1.347839    5.581614   -0.169740
H   -1.346670    5.701598    1.987361
H   -1.417536    5.135961   -2.283733
H   -1.140889    6.639166   -0.314725
Ir   2.681948   -0.446734    0.042524
P    3.291746   -0.450847    2.251639
C    0.581854    0.188423    0.754801
P    4.482433    0.891152   -0.613026
C   -0.182669   -0.641981   -0.064510
H    0.466845    0.063605    1.829988
H   -0.158383   -0.481095   -1.137622
Cl   2.176094   -0.471172   -2.412730
C    3.973062   -2.290635   -0.344822
H    4.769912   -2.364653    0.392264
H    4.295883   -2.237466   -1.380754
C    2.678042   -2.720903   -0.037215
H    1.988264   -2.986164   -0.833189
H    2.442778   -3.114042    0.949271
H    2.447732    0.224499    3.153918
H    3.407424   -1.713450    2.865838
H    4.539387    0.119353    2.570144
H    5.436262    1.263241    0.356450
H    5.309790    0.370120   -1.623742
H    4.145585    2.144716   -1.155308
H    4.131765    2.141804    0.842020
```

65
Alkenyl-TSa
Eopt -2208.353326

```
C    1.270631    0.809805    1.077878
H    1.213651    1.743735    0.523534
H    1.477513    0.937998    2.139523
C   -1.050942   -1.638729   -0.917631
H   -0.434783   -2.032759   -1.741085
H   -0.983809   -2.358616   -0.082453
O   -2.356034   -1.477967   -1.374714
C   -3.580855   -1.809986    0.950270
C   -4.674309   -0.454390   -0.983721
H   -2.677834   -2.115037    1.512571
C   -4.210803   -3.113174    0.397954
C   -4.479926   -1.063181    1.969790
H   -4.548961    0.223443   -1.848754
C   -5.307993   -1.756914   -1.536985
C   -5.570480    0.289546    0.036274
H   -4.494359   -3.795791    1.219538
H   -3.434388   -3.638206   -0.177917
C   -5.441641   -2.914442   -0.518770
H   -3.880981   -0.259999    2.427171
H   -4.764400   -1.729075    2.804302
C   -5.767148   -0.432999    1.389175
H   -4.679242   -2.100116   -2.370504
H   -6.305373   -1.556063   -1.968433
H   -5.114908    1.272744    0.232577
```

H -6.565583 0.501687 -0.393592
H -5.632645 -3.850548 -1.066523
H -6.177016 0.280674 2.120933
H -6.332594 -2.756165 0.099417
H -6.534527 -1.207164 1.280565
B -3.219984 -0.819593 -0.319843
C -2.351207 0.519145 0.181864
H -2.148737 0.657421 1.248293
C -2.200612 1.648613 -0.588870
H -2.392302 1.550846 -1.659824
C -1.789901 2.991209 -0.185557
C -1.609348 3.973805 -1.185069
C -1.566897 3.363064 1.160269
C -1.214661 5.271614 -0.858992
H -1.779345 3.704169 -2.225207
C -1.174760 4.660217 1.485198
H -1.709738 2.633950 1.952504
C -0.994720 5.620030 0.478638
H -1.078988 6.009998 -1.645408
H -1.011301 4.928895 2.526152
H -0.690098 6.631114 0.737340
Ir 2.587009 -0.543766 0.026277
P 2.868258 -1.927104 1.816260
C 0.385704 -0.266628 0.710860
P 4.439132 0.805346 0.546346
C -0.411040 -0.327554 -0.469624
H 0.135153 -0.997397 1.477691
H -0.173647 0.373802 -1.260621
Cl 2.395098 0.951800 -1.983598
C 3.949943 -1.832240 -1.222135
H 4.643480 -2.405070 -0.609228
H 4.413003 -1.195144 -1.970907
C 2.628746 -2.279452 -1.405063
H 2.071925 -1.973979 -2.286616
H 2.282723 -3.191906 -0.924071
H 1.915697 -1.850546 2.851888
H 2.873793 -3.311815 1.551675
H 4.064098 -1.797903 2.550916
H 5.253770 0.455577 1.644714
H 5.415768 0.953954 -0.456700
H 4.179290 2.156566 0.847736
65
Alkenyl-TSb
Eopt -2208.350009
C 1.371738 1.264955 0.251344
H 1.172215 1.692774 -0.729006
H 1.665142 1.997801 1.001513
C -0.967214 -1.869858 0.356014
H -0.212428 -2.648904 0.162087
H -1.107514 -1.827165 1.452986
O -2.132239 -2.189224 -0.326714
C -3.981084 -1.164272 1.232680
C -4.293538 -1.261587 -1.352145
H -3.329666 -1.018057 2.115355
C -4.551356 -2.603051 1.356052
C -5.074270 -0.068103 1.317930
H -3.836464 -1.198422 -2.357576
C -4.890165 -2.688918 -1.228728
C -5.346458 -0.131153 -1.266855
H -5.133821 -2.714692 2.288050
H -3.699291 -3.291718 1.442618
C -5.433582 -3.064837 0.171424
H -4.577269 0.907251 1.425488
H -5.678574 -0.193164 2.234278
C -6.035081 0.019455 0.108465
H -4.094606 -3.399251 -1.492775
H -5.695931 -2.841780 -1.969050
H -4.846325 0.819617 -1.510538
H -6.128116 -0.262305 -2.035901
H -5.553839 -4.158251 0.223037
H -6.562077 0.985662 0.141800
H -6.443965 -2.658522 0.293048
H -6.818506 -0.739811 0.206430
B -3.153279 -1.086659 -0.190909
C -2.293086 0.350489 -0.433937
H -2.173099 0.639427 -1.483447
C -2.195984 1.390043 0.461988
H -2.269748 1.145907 1.523396
C -1.962973 2.805979 0.205086
C -1.824686 3.678856 1.308051
C -1.867831 3.351824 -1.096061
C -1.594196 5.042306 1.123186
H -1.901818 3.272995 2.314478
C -1.638187 4.712929 -1.279128
H -1.976097 2.706249 -1.962599
C -1.499369 5.563563 -0.172098
H -1.491145 5.697401 1.984594
H -1.568171 5.117031 -2.285914
H -1.321751 6.625683 -0.321361
Ir 2.694628 -0.430295 0.049592
P 3.282692 -0.424592 2.247804
C 0.582848 0.142551 0.688796
P 4.496560 0.943221 -0.626591
C -0.387594 -0.553711 -0.120669
H 0.465476 0.001813 1.762634
H -0.256851 -0.469133 -1.196059
Cl 2.161672 -0.474983 -2.411136
C 3.966678 -2.239054 -0.323094
H 4.769920 -2.342381 0.404443
H 4.286905 -2.221961 -1.361573
C 2.659092 -2.662359 -0.000948
H 1.978210 -2.958407 -0.794544
H 2.444074 -3.083570 0.979159
H 2.409722 0.214304 3.151876
H 3.436053 -1.683603 2.864381
H 4.505731 0.183464 2.596679
H 5.464411 1.335874 0.324606
H 5.339708 0.445248 -1.639407
H 4.177998 2.201413 -1.176538
65

Alkenyl-Pdt
Eopt -2208.439448

C	1.244650	0.816228	1.067753
H	1.198851	1.753598	0.517466
H	1.482257	0.933529	2.124770
C	-1.067148	-1.628678	-0.933376
H	-0.425652	-1.953903	-1.760983
H	-0.972609	-2.368780	-0.119952
O	-2.386944	-1.516877	-1.382852
C	-3.561984	-1.800838	0.960142
C	-4.663910	-0.448266	-0.978016
H	-2.656196	-2.105782	1.511996
C	-4.199834	-3.108889	0.407590
C	-4.460413	-1.054981	1.976914
H	-4.551798	0.222739	-1.848224
C	-5.308796	-1.755042	-1.523958
C	-5.550412	0.295552	0.050494
H	-4.480062	-3.778545	1.239585
H	-3.425321	-3.639352	-0.163823
C	-5.434133	-2.913579	-0.505243
H	-3.862759	-0.248359	2.430478
H	-4.736194	-1.723917	2.810605
C	-5.751271	-0.432549	1.398770
H	-4.690497	-2.097187	-2.364891
H	-6.309171	-1.547344	-1.942374
H	-5.087145	1.275192	0.252122
H	-6.542183	0.517365	-0.379884
H	-5.620704	-3.849599	-1.053754
H	-6.164188	0.277312	2.131814
H	-6.324147	-2.760787	0.114886
H	-6.515328	-1.208808	1.285798
B	-3.221384	-0.866000	-0.356929
C	-2.054931	0.442552	0.146775
H	-1.997076	0.587269	1.228410
C	-2.118857	1.641768	-0.587639
H	-2.218643	1.527127	-1.668884
C	-1.844678	2.996929	-0.175083
C	-1.657616	3.974753	-1.185359
C	-1.595721	3.361072	1.171426
C	-1.246187	5.265938	-0.863247
H	-1.825544	3.698992	-2.223638
C	-1.197791	4.655069	1.489045
H	-1.714058	2.624047	1.959636
C	-1.021878	5.610257	0.475314
H	-1.100548	6.002051	-1.649100
H	-1.015698	4.925624	2.525716
H	-0.705739	6.618393	0.731016
Ir	2.590433	-0.545529	0.025662
P	2.867391	-1.913701	1.811886
C	0.396336	-0.267818	0.678807
P	4.452591	0.825317	0.539280
C	-0.637367	-0.242279	-0.409892
H	0.157831	-0.989462	1.461328
H	-0.273724	0.356013	-1.246661
Cl	2.379391	0.940939	-1.996824
C	3.927065	-1.823143	-1.208055
H	4.628690	-2.410369	-0.616784
H	4.388005	-1.209097	-1.978084
C	2.594381	-2.277474	-1.380314
H	2.044053	-1.994674	-2.273783
H	2.269046	-3.205673	-0.914099
H	1.909545	-1.845329	2.845437
H	2.885305	-3.302461	1.561965
H	4.054554	-1.783258	2.563491
H	5.287046	0.493185	1.632045
H	5.439863	0.986892	-0.455079
H	4.222648	2.184419	0.844375

67
Alkyl-Rct
Eopt -2209.587882

C	-1.185206	0.178073	-1.465188
H	-0.884536	1.221128	-1.547232
H	-1.339923	-0.310822	-2.425702
C	0.607443	-0.976422	1.808835
H	1.129581	-0.324807	2.528969
H	-0.218624	-1.449001	2.368944
O	1.430814	-1.952797	1.273906
C	2.465005	-2.283267	-1.070510
C	3.989772	-1.725808	0.958316
H	1.511298	-2.147285	-1.614111
C	2.541411	-3.794240	-0.711599
C	3.583004	-1.844335	-2.047218
H	4.149261	-1.182040	1.908992
C	4.072143	-3.232278	1.314613
C	5.114503	-1.269633	-0.003428
H	2.541033	-4.413044	-1.627628
H	1.623636	-4.054893	-0.165581
C	3.755077	-4.206153	0.155269
H	3.337508	-0.840292	-2.422814
H	3.599194	-2.494182	-2.941358
C	5.011572	-1.814506	-1.449838
H	3.357250	-3.419039	2.127910
H	5.069492	-3.485438	1.718486
H	5.114986	-0.172313	-0.042059
H	6.107004	-1.547562	0.396104
H	3.574146	-5.210318	0.570791
H	5.660462	-1.212845	-2.105426
H	4.639189	-4.314323	-0.483346
H	5.432287	-2.825607	-1.480345
B	2.507875	-1.392430	0.326627
C	2.398763	0.321024	0.009744
H	1.565360	0.706335	-0.594448
C	2.739706	1.398491	1.089478
H	1.867183	1.636608	1.712074
C	3.275986	2.694038	0.498664
C	2.415380	3.712558	0.049625
C	4.660458	2.900781	0.356675
C	2.916191	4.885749	-0.527337
H	1.339166	3.588198	0.157765
C	5.168256	4.070072	-0.220261

H 5.349545 2.134513 0.706306
C 4.296838 5.069577 -0.667694
H 2.227255 5.658349 -0.862601
H 6.244135 4.201920 -0.314892
H 4.687830 5.981188 -1.113619
Ir -2.763382 -0.002319 -0.017535
P -3.383475 -2.080026 -0.750350
C -0.583012 -0.629310 -0.424880
P -4.294022 1.113418 -1.402550
C -0.007639 -0.110771 0.733131
H -0.491435 -1.699723 -0.580156
H -0.051838 0.960556 0.893622
Cl -2.241837 2.333888 0.774449
C -4.352586 -0.065767 1.609268
H -5.162425 -0.737956 1.334028
H -4.643076 0.961541 1.810037
C -3.162867 -0.571312 2.144703
H -2.522478 0.066358 2.747056
H -3.017396 -1.642347 2.265737
H -4.496724 -2.145104 -1.609294
H -2.426994 -2.809353 -1.481513
H -3.749557 -3.007500 0.244379
H -3.729257 1.973297 -2.361599
H -5.165533 0.346309 -2.202082
H -5.198201 1.975840 -0.758025
H 3.497529 0.990842 1.770562
H 3.220658 0.389596 -0.716452
67
Alkyl-TSa
Eopt -2209.537547
C -0.855943 -0.593076 -1.607801
H -0.925468 0.390257 -2.069175
H -0.842692 -1.419539 -2.316221
C 1.160006 0.043314 1.662716
H 1.504179 0.986265 2.115333
H 0.434978 -0.397460 2.363917
O 2.194444 -0.850991 1.432741
C 3.147551 -1.743532 -0.816102
C 4.569830 -0.180643 0.702134
H 2.172091 -2.008809 -1.254864
C 3.609219 -2.977314 0.006068
C 4.083922 -1.425616 -2.009373
H 4.613923 0.673917 1.398362
C 5.048728 -1.418406 1.513863
C 5.495242 0.156532 -0.490272
H 3.714217 -3.859282 -0.648662
H 2.811439 -3.214585 0.723124
C 4.929929 -2.779223 0.786327
H 3.577843 -0.692980 -2.657332
H 4.216321 -2.323152 -2.636991
C 5.483603 -0.875406 -1.643083
H 4.444963 -1.463053 2.429346
H 6.095138 -1.281359 1.835593
H 5.201104 1.138227 -0.892081
H 6.535596 0.283220 -0.146175
H 5.032839 -3.587587 1.525967
H 5.934628 -0.420744 -2.537627
H 5.776100 -2.903619 0.102536
H 6.140352 -1.710867 -1.381237
B 3.039262 -0.506996 0.242469
C 2.363439 1.031436 -0.606659
H 1.540111 1.162228 -1.318848
C 2.699719 2.383383 0.068516
H 3.026133 2.225897 1.102478
C 1.578777 3.403337 0.057580
C 1.004388 3.874222 1.247562
C 1.088408 3.908829 -1.158846
C -0.027499 4.820120 1.228605
H 1.367355 3.495678 2.200944
C 0.061468 4.855321 -1.185689
H 1.520143 3.555764 -2.093507
C -0.502736 5.315235 0.010829
H -0.460906 5.164944 2.164375
H -0.300798 5.233191 -2.138874
H -1.306142 6.047227 -0.008015
Ir -2.296521 -0.724819 0.004890
P -2.166131 -2.990830 0.170864
C -0.084658 -0.739829 -0.401245
P -4.153454 -0.730947 -1.443082
C 0.457988 0.338115 0.364722
H 0.363602 -1.705613 -0.190553
H 0.018479 1.320188 0.227819
Cl -2.537707 1.759304 -0.139981
C -3.676029 -0.607504 1.780242
H -4.225137 -1.531182 1.952160
H -4.290267 0.281360 1.663978
C -2.340004 -0.479845 2.217552
H -1.938810 0.506527 2.434293
H -1.844824 -1.305283 2.724988
H -3.249655 -3.748779 -0.315115
H -1.089841 -3.623329 -0.482726
H -2.040635 -3.521747 1.471115
H -3.952201 -0.210288 -2.736109
H -4.787931 -1.950709 -1.762966
H -5.265082 0.032378 -1.039114
H 3.567795 2.826072 -0.441752
H 3.153994 0.772400 -1.322104
67
Alkyl-TSb
Eopt -2209.543653
C 1.117701 0.956167 0.738462
H 1.137278 1.784622 0.034736
H 1.230566 1.258217 1.777776
C -1.021286 -1.751765 -1.089496
H -0.429675 -2.217765 -1.889111
H -0.942459 -2.399168 -0.201701
O -2.340986 -1.625937 -1.535059
C -3.208224 -1.403784 0.940524
C -4.687383 -0.787833 -1.109857
H -2.226357 -1.414581 1.439146

C	-3.660817	-2.887956	0.815361
C	-4.146626	-0.585892	1.863592
H	-4.744096	-0.385769	-2.137197
C	-5.202230	-2.252659	-1.183659
C	-5.549332	0.129689	-0.211469
H	-3.711945	-3.352371	1.814806
H	-2.892553	-3.441203	0.257734
C	-5.019938	-3.089015	0.106305
H	-3.638340	0.346150	2.130672
H	-4.289693	-1.125018	2.815735
C	-5.541448	-0.237011	1.288773
H	-4.653368	-2.749225	-1.994725
H	-6.267474	-2.272643	-1.470264
H	-5.187668	1.164007	-0.321700
H	-6.594132	0.143065	-0.563616
H	-5.139610	-4.154489	-0.140653
H	-5.964221	0.599218	1.865357
H	-5.827600	-2.862211	0.809724
H	-6.225720	-1.075686	1.452409
B	-3.153401	-0.855260	-0.596251
C	-1.598891	3.217723	0.298335
C	-0.745455	3.780732	1.260826
C	-1.888781	3.977476	-0.847030
C	-0.194405	5.054890	1.088613
H	-0.501890	3.208026	2.153500
C	-1.343141	5.252968	-1.026159
H	-2.545704	3.564270	-1.608532
C	-0.490527	5.798269	-0.059237
H	0.469143	5.463936	1.847313
H	-1.582694	5.820292	-1.922660
H	-0.061445	6.787259	-0.199733
Ir	2.428950	-0.613721	0.037466
P	2.485056	-1.691277	2.042151
C	0.245355	-0.158863	0.466596
P	4.347548	0.672129	0.450852
C	-0.403278	-0.403048	-0.771289
H	-0.123667	-0.741099	1.308893
H	-0.060759	0.171911	-1.621574
Cl	2.480772	0.534493	-2.185932
C	3.697727	-2.217150	-0.923513
H	4.304495	-2.760244	-0.201624
H	4.249465	-1.751847	-1.735873
C	2.336458	-2.536197	-1.099166
H	1.856545	-2.311722	-2.047278
H	1.869744	-3.325861	-0.514298
H	1.525661	-1.309799	3.001235
H	2.301166	-3.088608	2.014609
H	3.668120	-1.598824	2.801399
H	5.077199	0.464664	1.640224
H	5.376913	0.578882	-0.503720
H	4.169441	2.067551	0.500534
C	-2.322408	0.952940	-0.723675
H	-1.633557	1.291803	-1.500422
H	-3.281953	1.115744	-1.230652
C	-2.213402	1.843698	0.516856
H	-1.645426	1.339319	1.310007
H	-3.218201	1.990075	0.933969

67

Alkyl-Pdt

Eopt -2209.663469

C	-1.161647	0.172937	-1.456834
H	-0.880711	1.220524	-1.556136
H	-1.349670	-0.322883	-2.408967
C	0.639075	-0.968677	1.801394
H	1.153430	-0.410462	2.600265
H	-0.256264	-1.427076	2.239445
O	1.452032	-1.984109	1.275739
C	2.459508	-2.299976	-1.084382
C	3.990853	-1.715311	0.950599
H	1.499709	-2.151244	-1.601291
C	2.546740	-3.812161	-0.726454
C	3.578837	-1.832613	-2.049958
H	4.126034	-1.174369	1.900129
C	4.082693	-3.233691	1.304522
C	5.102536	-1.249770	-0.019868
H	2.554344	-4.419594	-1.645929
H	1.628445	-4.079167	-0.186975
C	3.765427	-4.206529	0.142485
H	3.328126	-0.821513	-2.407385
H	3.581605	-2.466789	-2.951135
C	5.008632	-1.807309	-1.459901
H	3.380763	-3.424826	2.125733
H	5.087226	-3.463628	1.694325
H	5.078598	-0.150539	-0.068406
H	6.092418	-1.504456	0.392093
H	3.589333	-5.207848	0.562071
H	5.652514	-1.203831	-2.116185
H	4.648159	-4.310409	-0.496421
H	5.430800	-2.816773	-1.487288
B	2.502683	-1.608912	0.360837
C	2.079587	0.485270	0.050246
H	1.671296	1.055564	-0.789861
C	2.708460	1.429685	1.094065
H	1.926473	1.715564	1.809646
C	3.277915	2.703402	0.489775
C	2.411946	3.719400	0.046201
C	4.659724	2.904679	0.349585
C	2.909165	4.891898	-0.528731
H	1.336899	3.590880	0.157083
C	5.163972	4.077881	-0.226632
H	5.349677	2.138326	0.696613
C	4.290499	5.075375	-0.670587
H	2.219480	5.664667	-0.860972
H	6.239147	4.211114	-0.324279
H	4.679821	5.987584	-1.116420
Ir	-2.766748	-0.011325	-0.012563
P	-3.376062	-2.076672	-0.740118
C	-0.603417	-0.596519	-0.382464
P	-4.306255	1.118961	-1.402410
C	0.318830	-0.039148	0.650281

H	-0.516428	-1.670095	-0.534118
H	0.025072	0.966673	0.947858
Cl	-2.236039	2.329126	0.785655
C	-4.327570	-0.071369	1.594171
H	-5.149959	-0.746116	1.360441
H	-4.626861	0.947674	1.826493
C	-3.119976	-0.585847	2.124403
H	-2.498146	0.046036	2.753528
H	-3.009038	-1.655411	2.291320
H	-4.488625	-2.164402	-1.602824
H	-2.425409	-2.815522	-1.474803
H	-3.744857	-3.019793	0.242398
H	-3.788487	1.991809	-2.383251
H	-5.196361	0.375868	-2.212526
H	-5.230684	1.992929	-0.793119
H	3.483618	0.918838	1.673024
H	2.865079	-0.087006	-0.466629

61
Phenyl-Rct
Eopt -2130.963323

C	-1.465880	1.594307	-0.025702
H	-1.205946	1.842531	1.001023
H	-1.848943	2.435175	-0.600980
C	1.044677	-1.328477	-0.771027
H	0.431716	-2.246478	-0.785920
H	1.216224	-1.050914	-1.827675
O	2.216176	-1.597894	-0.073439
C	4.288975	-0.539986	-1.292578
C	4.178639	-0.671691	1.302074
H	3.780561	-0.407535	-2.266713
C	4.915588	-1.955315	-1.336778
C	5.341735	0.593933	-1.194634
H	3.577788	-0.632382	2.230191
C	4.800141	-2.090025	1.256925
C	5.234376	0.456879	1.410859
H	5.650885	-2.034621	-2.158477
H	4.112047	-2.666206	-1.578025
C	5.594815	-2.419913	-0.027761
H	4.832082	1.552269	-1.359586
H	6.086037	0.509463	-2.007711
C	6.103854	0.676849	0.149614
H	3.979053	-2.814685	1.352621
H	5.459720	-2.257965	2.127896
H	4.710590	1.395803	1.635493
H	5.909015	0.280241	2.268311
H	5.759569	-3.508005	-0.077242
H	6.592944	1.661086	0.221378
H	6.596485	-1.980136	0.039339
H	6.923024	-0.050858	0.144785
B	3.231794	-0.453413	-0.024045
C	2.457488	1.029229	-0.003631
C	2.158780	1.705923	1.201449
C	2.291017	1.795779	-1.181556
C	1.835112	3.066871	1.245793
H	2.231143	1.163886	2.143075
C	1.963443	3.152599	-1.164277
H	2.467362	1.324785	-2.147180
C	1.766329	3.806690	0.059790
H	1.659975	3.555458	2.203078
H	1.887932	3.709649	-2.097157
H	1.552254	4.873342	0.086768
Ir	-2.627667	-0.212959	-0.046985
P	-3.386218	0.150508	-2.176456
C	-0.616458	0.659533	-0.735263
P	-4.451381	0.861470	0.955719
C	0.211486	-0.263977	-0.098853
H	-0.552084	0.725344	-1.818929
H	0.235193	-0.280812	0.985241
Cl	-1.952406	-0.644436	2.335277
C	-3.758452	-2.187244	0.043141
H	-4.596919	-2.190141	-0.650149
H	-4.009699	-2.365025	1.084977
C	-2.458424	-2.433976	-0.413855
H	-1.699560	-2.794913	0.274366
H	-2.266110	-2.614362	-1.469329
H	-2.619602	1.007671	-2.989090
H	-3.505925	-0.986692	-2.999044
H	-4.666916	0.720126	-2.311011
H	-5.489610	1.349798	0.136495
H	-5.175440	0.103886	1.892947
H	-4.141412	2.007100	1.710804

61
Phenyl-TS
Eopt -2130.931746

C	-1.451312	1.593880	0.124366
H	-1.205397	1.776829	1.168453
H	-1.854708	2.461874	-0.394701
C	1.071120	-1.264900	-0.797724
H	0.359726	-2.104264	-0.767269
H	1.220546	-1.005585	-1.862552
O	2.238300	-1.636851	-0.149700
C	4.272826	-0.488209	-1.302129
C	4.144463	-0.700259	1.288418
H	3.770451	-0.332216	-2.274121
C	4.903379	-1.907262	-1.380074
C	5.322064	0.640649	-1.161009
H	3.540476	-0.683367	2.213878
C	4.762287	-2.123147	1.208587
C	5.203561	0.421663	1.427710
H	5.648395	-1.953766	-2.194211
H	4.102953	-2.607247	-1.656512
C	5.566799	-2.417157	-0.078752
H	4.807711	1.605554	-1.283625
H	6.059301	0.591538	-1.982012
C	6.087029	0.667301	0.182133
H	3.936452	-2.844365	1.275911
H	5.410875	-2.315609	2.081546
H	4.678092	1.359120	1.663606
H	5.863865	0.226753	2.291107
H	5.723868	-3.503976	-0.161482

H	6.588434	1.641824	0.287781
H	6.570391	-1.987197	0.012071
H	6.895536	-0.071141	0.155498
B	3.211458	-0.498669	-0.040523
C	2.247429	0.983310	0.003497
C	2.075091	1.677820	1.236574
C	2.189608	1.804818	-1.163626
C	1.873051	3.055684	1.309255
H	2.092655	1.105365	2.161691
C	1.987675	3.178823	-1.112442
H	2.297798	1.333166	-2.138060
C	1.826582	3.811166	0.131141
H	1.747728	3.542422	2.273918
H	1.953988	3.765007	-2.028450
H	1.663021	4.885603	0.178205
Ir	-2.627642	-0.210492	-0.056545
P	-3.389507	0.350532	-2.126193
C	-0.624068	0.686239	-0.629863
P	-4.466401	0.745330	1.076544
C	0.441374	-0.102587	-0.063489
H	-0.569864	0.828939	-1.707967
H	0.404347	-0.249562	1.011191
Cl	-1.895403	-0.868339	2.263568
C	-3.706263	-2.175872	-0.166290
H	-4.553106	-2.153192	-0.850431
H	-3.942610	-2.491293	0.846764
C	-2.394938	-2.345169	-0.662975
H	-1.634594	-2.784641	-0.022807
H	-2.218032	-2.441995	-1.732023
H	-2.703024	1.379799	-2.802117
H	-3.384332	-0.666811	-3.102375
H	-4.719995	0.804188	-2.230855
H	-5.540726	1.280356	0.332320
H	-5.172893	-0.087382	1.965612
H	-4.198630	1.834353	1.930466

61
Phenyl-Pdt
Eopt -2131.032617

C	-1.415053	1.582946	-0.033511
H	-1.193922	1.864702	0.994330
H	-1.830599	2.391822	-0.633999
C	1.104318	-1.322408	-0.743926
H	0.379869	-2.134603	-0.631604
H	1.270999	-1.159696	-1.823925
O	2.285000	-1.663718	-0.068406
C	4.266286	-0.515707	-1.295702
C	4.168232	-0.653282	1.313379
H	3.744578	-0.400954	-2.258738
C	4.921778	-1.934764	-1.332645
C	5.296286	0.631664	-1.187077
H	3.555285	-0.622928	2.228223
C	4.815768	-2.071050	1.272244
C	5.199440	0.495533	1.401545
H	5.662184	-1.980810	-2.148480
H	4.132829	-2.653205	-1.590179
C	5.601269	-2.395927	-0.020523
H	4.761281	1.583449	-1.326394
H	6.019695	0.572416	-2.017341
C	6.075137	0.703122	0.145387
H	4.007491	-2.804498	1.388905
H	5.485132	-2.207439	2.137968
H	4.650966	1.429501	1.597885
H	5.859405	0.347756	2.272561
H	5.761870	-3.483185	-0.071881
H	6.570617	1.683023	0.215081
H	6.602905	-1.958388	0.043028
H	6.886391	-0.032066	0.136807
B	3.278344	-0.619942	-0.020533
C	1.940496	0.973720	-0.033128
C	1.980482	1.692970	1.197239
C	2.100643	1.760656	-1.216017
C	1.912298	3.082480	1.241926
H	1.906907	1.132079	2.126237
C	2.031108	3.147288	-1.184189
H	2.132193	1.260024	-2.180775
C	1.858353	3.805819	0.044194
H	1.834735	3.596347	2.196911
H	2.051357	3.717701	-2.109864
H	1.700281	4.881523	0.065379
Ir	-2.630947	-0.221951	-0.054945
P	-3.370024	0.128013	-2.168277
C	-0.598327	0.596862	-0.674708
P	-4.472786	0.871873	0.969982
C	0.636512	-0.043940	-0.065032
H	-0.548737	0.653776	-1.763155
H	0.450830	-0.272712	0.986251
Cl	-1.923235	-0.647274	2.329068
C	-3.725886	-2.154957	0.046045
H	-4.567924	-2.215027	-0.642407
H	-3.972440	-2.371071	1.082830
C	-2.408748	-2.397182	-0.425202
H	-1.662878	-2.791380	0.260061
H	-2.241015	-2.637876	-1.473551
H	-2.599808	0.970379	-2.998319
H	-3.502586	-1.006615	-2.997057
H	-4.644977	0.708277	-2.337920
H	-5.538110	1.361698	0.178254
H	-5.220081	0.147164	1.921887
H	-4.218777	2.032834	1.732143

65
Alkyl-Rct-Bpin
Eopt -2282.715498

C	-1.279566	0.460869	-1.422549
H	-1.046612	1.510315	-1.251116
H	-1.426300	0.207099	-2.471098
C	0.495765	-1.350161	1.557221
H	-0.061883	-1.241561	2.501070
H	0.240081	-2.330895	1.124612
O	1.865388	-1.259581	1.843135
C	3.600755	-2.866263	-0.539995

C	4.753483	-1.925555	-0.002785
B	2.656186	-0.990405	0.599631
C	2.360044	0.583267	-0.009178
H	1.467545	0.822848	-0.595210
C	2.593791	1.713207	1.025328
H	1.725857	1.821625	1.689810
C	2.910799	3.057344	0.398986
C	1.921269	4.033667	0.191906
C	4.221154	3.352056	-0.021233
C	2.225587	5.256908	-0.418654
H	0.901307	3.836518	0.518296
C	4.532041	4.570357	-0.631939
H	5.006149	2.614900	0.137307
C	3.532917	5.530600	-0.835369
H	1.441552	5.997495	-0.563146
H	5.554627	4.773331	-0.943462
H	3.772073	6.481031	-1.306906
Ir	-2.809829	-0.173079	-0.048659
P	-3.316297	-2.048107	-1.265053
C	-0.605092	-0.531499	-0.609281
P	-4.431334	1.149527	-1.105898
C	-0.023047	-0.273054	0.628866
H	-0.479723	-1.537311	-1.002617
H	-0.018564	0.744151	1.002018
Cl	-2.404118	1.902675	1.304807
C	-4.361174	-0.739340	1.514877
H	-5.135272	-1.368398	1.081174
H	-4.706284	0.185835	1.967626
C	-3.132080	-1.286343	1.896605
H	-2.515586	-0.778982	2.632984
H	-2.919435	-2.341805	1.743447
H	-2.335018	-2.510365	-2.162350
H	-3.594992	-3.212070	-0.522367
H	-4.445865	-1.979320	-2.102219
H	-3.938370	2.242574	-1.840347
H	-5.284111	0.541965	-2.049188
H	-5.356725	1.778074	-0.254911
H	3.435428	1.417387	1.664992
H	3.161979	0.659518	-0.759137
O	2.440020	-2.032542	-0.441449
O	4.076989	-1.098748	0.944095
C	5.894511	-2.651548	0.720632
H	6.404760	-3.363295	0.058473
H	6.637775	-1.918791	1.060207
H	5.533637	-3.191828	1.600866
C	5.370847	-1.045280	-1.111363
H	6.000257	-0.278414	-0.643444
H	6.002574	-1.631029	-1.791266
H	4.609206	-0.537452	-1.709474
C	3.402963	-4.119934	0.336608
H	2.487243	-4.634850	0.018830
H	4.236247	-4.827920	0.241424
H	3.290329	-3.852927	1.392272
C	3.763602	-3.312011	-1.999963
H	4.685665	-3.890347	-2.144396
H	2.920081	-3.953672	-2.286491
H	3.780159	-2.457820	-2.683652

65

Alkyl-TS-Bpin

Eopt -2282.675599

C	-1.352208	0.334947	-1.567701
H	-1.165699	1.402815	-1.466752
H	-1.581488	0.021762	-2.585196
C	0.578329	-1.354812	1.357784
H	-0.034891	-1.274652	2.267413
H	0.357941	-2.326657	0.885603
O	1.920527	-1.248851	1.714296
C	3.812149	-2.705040	-0.534653
C	4.899890	-1.721528	0.054408
B	2.723370	-0.893317	0.517081
C	2.227070	0.634497	-0.049292
H	1.391620	0.871662	-0.709815
C	2.404263	1.763180	0.998724
H	1.558178	1.780875	1.698789
C	2.570657	3.145968	0.397907
C	1.486592	4.032355	0.280260
C	3.823705	3.574320	-0.077348
C	1.643572	5.298310	-0.297054
H	0.508912	3.731208	0.651597
C	3.987868	4.837456	-0.654266
H	4.680455	2.908584	0.011797
C	2.895845	5.706878	-0.768401
H	0.788182	5.966588	-0.371815
H	4.969188	5.145822	-1.008894
H	3.021695	6.691355	-1.213101
Ir	-2.733721	-0.276211	-0.034865
P	-3.165262	-2.304118	-1.007689
C	-0.570601	-0.582050	-0.770529
P	-4.506362	0.786815	-1.118077
C	0.119834	-0.255946	0.409954
H	-0.416189	-1.590440	-1.148024
H	0.005492	0.740193	0.816052
Cl	-2.439970	1.968426	1.056402
C	-4.198024	-0.748631	1.656465
H	-4.942691	-1.472055	1.331236
H	-4.589336	0.202719	2.005570
C	-2.925545	-1.169274	2.055422
H	-2.322151	-0.537137	2.700356
H	-2.653248	-2.221679	2.028200
H	-4.476616	-2.513112	-1.475682
H	-2.410857	-2.640253	-2.147963
H	-2.980598	-3.443961	-0.201453
H	-4.152845	1.800375	-2.027454
H	-5.375588	0.008537	-1.909946
H	-5.423913	1.466309	-0.297363
H	3.291223	1.527486	1.599308
H	3.060395	0.748209	-0.769796
O	2.643631	-1.878926	-0.582315
O	4.133990	-0.874302	0.916235
C	5.997287	-2.402963	0.880987

H 6.564366 -3.124229 0.278002
H 6.703417 -1.648579 1.251029
H 5.582305 -2.925017 1.748277
C 5.569253 -0.856667 -1.032713
H 6.151569 -0.064922 -0.545418
H 6.253482 -1.441086 -1.660761
H 4.829896 -0.379361 -1.683975
C 3.541559 -3.912424 0.386504
H 2.656694 -4.446787 0.017678
H 4.380451 -4.619896 0.396881
H 3.339370 -3.593038 1.413138
C 4.111985 -3.221557 -1.946263
H 5.050224 -3.790848 -1.973105
H 3.306474 -3.891363 -2.274396
H 4.182042 -2.403786 -2.670039
65
Alkyl-Pdt-Bpin
Eopt -2282.801757
C -1.264901 0.454744 -1.418814
H -1.061033 1.512779 -1.262133
H -1.442113 0.189857 -2.460820
C 0.536482 -1.296012 1.537360
H -0.030743 -1.163400 2.464580
H 0.265078 -2.265899 1.099033
O 1.913712 -1.272316 1.865319
C 3.633529 -2.867200 -0.561049
C 4.777303 -1.910861 -0.031807
B 2.707465 -1.256990 0.725911
C 2.005466 0.719179 -0.025496
H 1.441386 1.250591 -0.795824
C 2.542278 1.726991 1.019328
H 1.763120 1.894075 1.773645
C 2.898608 3.068514 0.393766
C 1.903416 4.042247 0.191758
C 4.206375 3.365631 -0.024889
C 2.200618 5.265932 -0.416053
H 0.885076 3.838248 0.517724
C 4.509888 4.589489 -0.634829
H 4.995924 2.633018 0.131047
C 3.507295 5.544488 -0.835232
H 1.413564 6.003541 -0.557135
H 5.530733 4.796722 -0.948501
H 3.741559 6.496134 -1.306520
Ir -2.813964 -0.193069 -0.044621
P -3.303087 -2.056385 -1.252216
C -0.626262 -0.506057 -0.569346
P -4.449011 1.142237 -1.103629
C 0.276862 -0.153548 0.550562
H -0.470220 -1.505313 -0.975208
H -0.026970 0.764406 1.048257
Cl -2.402612 1.888368 1.316072
C -4.330535 -0.752302 1.498322
H -5.114114 -1.400254 1.108212
H -4.688751 0.152228 1.983594
C -3.078779 -1.300283 1.873389
H -2.487961 -0.806217 2.640300
H -2.896872 -2.368242 1.768748
H -2.324568 -2.525739 -2.153445
H -3.582306 -3.234072 -0.526585
H -4.429088 -2.012989 -2.100908
H -4.012524 2.253492 -1.856389
H -5.320918 0.558162 -2.051778
H -5.400982 1.786754 -0.286733
H 3.410760 1.318676 1.545513
H 2.808527 0.239605 -0.590763
O 2.448846 -2.037740 -0.420638
O 4.070908 -1.082456 0.929901
C 5.910128 -2.622454 0.707884
H 6.422100 -3.332285 0.046973
H 6.648074 -1.885534 1.046466
H 5.546589 -3.162903 1.586125
C 5.371447 -1.009885 -1.121755
H 5.999868 -0.245557 -0.650445
H 6.000395 -1.587296 -1.808877
H 4.601555 -0.501969 -1.711323
C 3.430434 -4.111051 0.321302
H 2.514792 -4.623197 0.003815
H 4.264358 -4.815379 0.224866
H 3.321530 -3.849778 1.379156
C 3.773520 -3.292178 -2.018974
H 4.697953 -3.862670 -2.169018
H 2.931904 -3.937261 -2.298125
H 3.781364 -2.433329 -2.696037
63
Alkyl-Rct-Bpin
Eopt -2281.499657
C 1.437965 1.130229 0.604848
H 1.283970 1.784482 -0.250790
H 1.654468 1.661742 1.530234
C -0.777051 -1.987500 -0.244824
H -0.120973 -2.765000 -0.669044
H -0.926081 -2.235449 0.820355
O -1.979349 -2.011119 -0.963005
C -4.841310 -1.729883 0.781950
C -5.350006 -1.098674 -0.573971
B -2.989091 -0.999085 -0.508338
C -2.358559 0.499759 -0.639411
H -2.129141 0.833185 -1.657974
C -2.289291 1.423271 0.343317
H -2.515684 1.097309 1.361100
C -2.003727 2.856626 0.220670
C -2.004077 3.654739 1.383464
C -1.796549 3.499400 -1.019798
C -1.828488 5.038656 1.314018
H -2.165909 3.177954 2.348695
C -1.621500 4.879913 -1.090695
H -1.795134 2.915423 -1.935911
C -1.642032 5.660170 0.074664
H -1.845616 5.632640 2.225242
H -1.477152 5.354490 -2.058890

H -1.516303 6.738520 0.014126
Ir 2.792167 -0.436024 0.045651
P 3.207614 -1.034092 2.218367
C 0.598508 -0.045564 0.723606
P 4.580490 1.055133 -0.058021
C -0.026925 -0.677956 -0.347582
H 0.404860 -0.448257 1.716415
H 0.069880 -0.251087 -1.340209
Cl 2.473399 0.208065 -2.356086
C 4.219072 -2.070828 -0.707307
H 4.957360 -2.311506 0.054593
H 4.615768 -1.720101 -1.655791
C 2.934490 -2.610255 -0.645296
H 2.321811 -2.666660 -1.540420
H 2.640551 -3.259715 0.176332
H 2.314341 -0.553292 3.194764
H 3.217741 -2.414731 2.497451
H 4.444502 -0.637401 2.761871
H 5.463412 1.123284 1.038326
H 5.475122 0.868773 -1.125978
H 4.234173 2.408879 -0.217275
O -3.493001 -1.260762 0.861342
O -4.171850 -1.108953 -1.382270
C -6.438461 -1.901416 -1.294322
H -7.340623 -2.000535 -0.676512
H -6.722997 -1.386534 -2.220995
H -6.088908 -2.902779 -1.563127
C -5.847325 0.352761 -0.401395
H -5.129087 0.966377 0.148569
H -5.981979 0.800415 -1.393979
H -6.811602 0.394167 0.120992
C -5.603311 -1.267211 2.029740
H -6.666218 -1.536632 1.973593
H -5.183441 -1.751496 2.921129
H -5.528229 -0.184681 2.171136
C -4.836178 -3.271058 0.744267
H -4.298624 -3.645446 1.624792
H -5.850292 -3.690646 0.765493
H -4.322610 -3.643542 -0.147679
63
Alkenyl-TS-Bpin
Eopt -2281.483137
C 1.503277 1.090326 0.715850
H 1.351038 1.794876 -0.098855
H 1.803890 1.556006 1.653095
C -0.882364 -1.884212 -0.171629
H -0.207544 -2.566739 -0.709204
H -0.930596 -2.227735 0.877410
O -2.125591 -1.912909 -0.799982
C -5.111700 -1.730544 0.662924
C -5.403653 -0.764463 -0.550927
B -3.067315 -0.927356 -0.215614
C -2.174892 0.422999 0.245816
H -2.027485 0.561667 1.320514
C -2.065093 1.552519 -0.528425
H -2.228566 1.440928 -1.602271
C -1.743041 2.914655 -0.115866
C -1.605672 3.910908 -1.109419
C -1.561057 3.292634 1.234726
C -1.292075 5.227567 -0.772428
H -1.746262 3.636563 -2.152685
C -1.249532 4.608800 1.570251
H -1.674495 2.553166 2.022018
C -1.111514 5.581785 0.569950
H -1.189637 5.976858 -1.553535
H -1.117300 4.881855 2.614477
H -0.870750 6.607855 0.836812
Ir 2.765025 -0.523164 0.027352
P 3.293490 -1.232770 2.126733
C 0.641028 -0.060790 0.800862
P 4.608623 0.927800 -0.137300
C -0.255276 -0.502029 -0.218380
H 0.486966 -0.509736 1.780856
H -0.128484 -0.088493 -1.212605
Cl 2.319327 0.227081 -2.337396
C 4.021702 -2.162263 -0.874893
H 4.803265 -2.502118 -0.197745
H 4.369754 -1.815003 -1.843989
C 2.703995 -2.636431 -0.733384
H 2.036999 -2.641797 -1.591127
H 2.451883 -3.333938 0.062630
H 2.450725 -0.828402 3.181268
H 3.333903 -2.627226 2.329416
H 4.551606 -0.857364 2.638958
H 5.551200 0.960444 0.912967
H 5.463804 0.742443 -1.239919
H 4.328682 2.302473 -0.264464
O -3.765007 -1.388292 1.006646
O -4.128577 -0.679044 -1.193587
C -5.832481 0.644302 -0.094278
H -5.815578 1.318051 -0.959795
H -6.848686 0.651470 0.319590
H -5.150206 1.048653 0.660802
C -6.431384 -1.292237 -1.558205
H -7.407230 -1.457961 -1.083448
H -6.571801 -0.559575 -2.363270
H -6.102807 -2.230862 -2.014520
C -6.008238 -1.514653 1.886627
H -7.065220 -1.682384 1.642077
H -5.732530 -2.223313 2.678248
H -5.902714 -0.503409 2.291499
C -5.164575 -3.217641 0.259314
H -4.765521 -3.822566 1.083255
H -6.189595 -3.554788 0.059303
H -4.552613 -3.410734 -0.627087
63
Alkenyl-Pdt-Bpin
Eopt -2281.577849
C 1.487828 1.092184 0.716389
H 1.353219 1.804556 -0.095142

H	1.827781	1.542132	1.648701
C	-0.906955	-1.870610	-0.137965
H	-0.196672	-2.493583	-0.690644
H	-0.946417	-2.233773	0.905321
O	-2.158285	-1.938489	-0.759001
C	-5.131421	-1.693585	0.662285
C	-5.408342	-0.739905	-0.561808
B	-3.092144	-0.965806	-0.213884
C	-1.912919	0.376568	0.253919
H	-1.932571	0.537733	1.335536
C	-1.962276	1.554730	-0.508396
H	-2.009894	1.422924	-1.591010
C	-1.752348	2.922597	-0.097062
C	-1.591899	3.908936	-1.104330
C	-1.536516	3.298722	1.252891
C	-1.236758	5.215501	-0.777334
H	-1.736142	3.627341	-2.144625
C	-1.190219	4.606903	1.574994
H	-1.636475	2.559082	2.041087
C	-1.038571	5.568194	0.563627
H	-1.112602	5.957236	-1.561639
H	-1.030224	4.883253	2.613794
H	-0.762804	6.587399	0.822231
Ir	2.758187	-0.536401	0.026033
P	3.298620	-1.241244	2.112394
C	0.638694	-0.058006	0.784069
P	4.624332	0.905557	-0.168615
C	-0.486709	-0.395651	-0.155253
H	0.501164	-0.494030	1.774935
H	-0.241757	-0.105518	-1.178465
Cl	2.285561	0.216110	-2.336039
C	3.970012	-2.174819	-0.878410
H	4.758051	-2.542584	-0.222460
H	4.310987	-1.854456	-1.859935
C	2.639926	-2.637716	-0.716612
H	1.973753	-2.657285	-1.574963
H	2.402148	-3.352204	0.069186
H	2.469332	-0.836408	3.179618
H	3.340097	-2.635368	2.326980
H	4.561239	-0.873972	2.623988
H	5.593686	0.941426	0.860688
H	5.478198	0.722827	-1.275955
H	4.387899	2.291252	-0.297704
O	-3.780162	-1.336747	1.019403
O	-4.114018	-0.646320	-1.180346
C	-5.847551	0.669069	-0.127518
H	-5.821864	1.333991	-0.999437
H	-6.869195	0.675435	0.271612
H	-5.177956	1.082492	0.634324
C	-6.409836	-1.284746	-1.585379
H	-7.392567	-1.451636	-1.126163
H	-6.539749	-0.560221	-2.399087
H	-6.067424	-2.225632	-2.026026
C	-6.045236	-1.474156	1.869941
H	-7.095839	-1.649156	1.605562
H	-5.780473	-2.177596	2.669331
H	-5.952965	-0.460087	2.270489
C	-5.168175	-3.185280	0.272533
H	-4.780789	-3.780738	1.108280
H	-6.190352	-3.523192	0.062420
H	-4.545969	-3.389408	-0.603935

59

Phenyl-Rct-Bpin

Eopt -2204.094368

C	1.369794	0.987689	1.209321
H	1.331845	1.983158	0.771021
H	1.538276	0.985891	2.285080
C	-0.890168	-1.129321	-1.188405
H	-0.305975	-1.483329	-2.051797
H	-0.943468	-1.955670	-0.460599
O	-2.164180	-0.772386	-1.656342
C	-4.638085	-1.534597	0.488984
C	-5.364984	-0.361710	-0.282366
B	-3.023273	-0.169533	-0.592322
C	-2.361050	1.251111	-0.068025
C	-2.264550	1.582488	1.296484
C	-2.192012	2.306805	-0.989011
C	-2.125632	2.903081	1.729695
H	-2.357556	0.787709	2.034026
C	-2.061695	3.635616	-0.575790
H	-2.224144	2.086970	-2.055567
C	-2.061058	3.942308	0.791257
H	-2.096541	3.130879	2.794427
H	-1.983876	4.433674	-1.312390
H	-1.995797	4.976987	1.121497
Ir	2.656191	-0.260768	0.023956
P	2.872217	-1.827101	1.684029
C	0.453045	-0.012693	0.701778
P	4.552386	0.930724	0.686566
C	-0.102130	0.012141	-0.574509
H	0.151578	-0.828491	1.355719
H	0.088013	0.870504	-1.208144
Cl	2.564941	1.452409	-1.806008
C	4.008753	-1.494298	-1.359442
H	4.673189	-2.126024	-0.774156
H	4.494709	-0.786314	-2.024766
C	2.688321	-1.878137	-1.593815
H	2.137482	-1.456801	-2.429629
H	2.292022	-2.800726	-1.176251
H	1.889406	-1.826992	2.691794
H	2.863529	-3.175727	1.278374
H	4.051111	-1.780645	2.452550
H	5.330788	0.419203	1.744167
H	5.527193	1.136470	-0.305051
H	4.315087	2.244977	1.126428
O	-3.281243	-1.085976	0.541149
O	-4.349725	0.105780	-1.169823
C	-4.697455	-2.869435	-0.279864
H	-4.027307	-3.589558	0.206855
H	-5.706969	-3.300157	-0.285119

H -4.365213 -2.746812 -1.315515
C -5.135967 -1.766127 1.920894
H -6.199259 -2.039107 1.937331
H -4.573427 -2.589841 2.379655
H -4.997916 -0.878448 2.545545
C -6.576934 -0.791210 -1.117083
H -7.359545 -1.239273 -0.490781
H -7.009039 0.086257 -1.615049
H -6.297301 -1.510489 -1.892856
C -5.801035 0.786050 0.653812
H -6.109893 1.642997 0.042447
H -6.651244 0.497411 1.284969
H -4.984916 1.114939 1.302615

59

Phenyl-TS-Bpin

Eopt -2204.063431

C 1.354011 0.973307 1.207183
H 1.323387 1.980463 0.795187
H 1.522152 0.941431 2.283025
C -0.929361 -1.056449 -1.225531
H -0.284968 -1.323380 -2.074928
H -0.935565 -1.904293 -0.522166
O -2.211695 -0.783886 -1.711161
C -4.626260 -1.540180 0.487944
C -5.358236 -0.355819 -0.260268
B -3.035137 -0.229452 -0.636178
C -2.124670 1.204393 -0.072979
C -2.149092 1.538387 1.306038
C -2.109277 2.298478 -0.979876
C -2.191527 2.857144 1.751381
H -2.148685 0.732235 2.035822
C -2.154182 3.624520 -0.551572
H -2.072778 2.090299 -2.047952
C -2.195697 3.907775 0.820340
H -2.221752 3.076509 2.816783
H -2.153175 4.435974 -1.276081
H -2.224848 4.939614 1.163347
Ir 2.664975 -0.266841 0.023724
P 2.867661 -1.838529 1.659156
C 0.482298 -0.025070 0.643973
P 4.573846 0.943005 0.699463
C -0.314263 0.155122 -0.538913
H 0.174698 -0.850939 1.283409
H 0.001637 0.952873 -1.200320
Cl 2.557330 1.466376 -1.801983
C 3.976656 -1.469005 -1.353740
H 4.646548 -2.132310 -0.809593
H 4.464888 -0.779964 -2.037829
C 2.635730 -1.841056 -1.573953
H 2.093327 -1.427459 -2.419769
H 2.256477 -2.786534 -1.191809
H 1.869741 -1.861191 2.654046
H 2.874228 -3.187123 1.248302
H 4.029912 -1.803562 2.455788
H 5.367147 0.444763 1.756001
H 5.568046 1.168707 -0.272195
H 4.366178 2.261502 1.150662
O -3.260014 -1.095488 0.516393
O -4.338236 0.138579 -1.141102
C -4.692516 -2.868626 -0.289920
H -4.018522 -3.591431 0.186300
H -5.702555 -3.296706 -0.287133
H -4.370818 -2.741426 -1.328301
C -5.099215 -1.780133 1.924783
H -6.162383 -2.050944 1.952682
H -4.531922 -2.608593 2.367707
H -4.950648 -0.897259 2.553667
C -6.566802 -0.780549 -1.101331
H -7.343766 -1.239452 -0.476529
H -7.005076 0.099973 -1.587589
H -6.285931 -1.490419 -1.884936
C -5.786695 0.781102 0.685542
H -6.107297 1.639920 0.083464
H -6.626251 0.484971 1.326451
H -4.961884 1.108531 1.325425

59

Phenyl-Pdt-Bpin

Eopt -2204.170632

C 1.343096 0.969474 1.211698
H 1.328542 1.981018 0.808911
H 1.544455 0.924707 2.281940
C -0.950232 -1.030347 -1.194973
H -0.294908 -1.274647 -2.035619
H -0.963855 -1.884740 -0.502581
O -2.245100 -0.778310 -1.699560
C -4.658011 -1.521718 0.494231
C -5.380713 -0.330378 -0.251538
B -3.120832 -0.341305 -0.691874
C -1.832908 1.210659 -0.058067
C -2.050349 1.524040 1.311212
C -2.010487 2.288136 -0.972962
C -2.176824 2.843545 1.750220
H -1.997062 0.728466 2.049345
C -2.128404 3.610108 -0.546608
H -1.910434 2.087883 -2.037866
C -2.140700 3.896094 0.825052
H -2.245351 3.055357 2.814944
H -2.151320 4.416245 -1.276098
H -2.138553 4.927575 1.169382
Ir 2.660664 -0.278453 0.020516
P 2.861469 -1.848228 1.646182
C 0.482916 -0.017831 0.633488
P 4.582304 0.932488 0.692827
C -0.497512 0.252049 -0.482602
H 0.179989 -0.839593 1.283766
H -0.018728 0.908764 -1.209135
Cl 2.545950 1.461284 -1.803960
C 3.955682 -1.466935 -1.353271
H 4.628849 -2.144269 -0.829412
H 4.444883 -0.789662 -2.049159

C 2.605699 -1.840382 -1.572341
H 2.073089 -1.437720 -2.429818
H 2.239497 -2.799914 -1.211919
H 1.870813 -1.873434 2.650357
H 2.861142 -3.200107 1.241421
H 4.025571 -1.828570 2.443315
H 5.394557 0.436703 1.739468
H 5.583743 1.173523 -0.271002
H 4.405745 2.252679 1.160379
O -3.275906 -1.076953 0.514375
O -4.344126 0.161052 -1.134864
C -4.711630 -2.845141 -0.291003
H -4.041793 -3.568554 0.188405
H -5.721975 -3.270026 -0.295984
H -4.385231 -2.718672 -1.328251
C -5.128051 -1.762168 1.925962
H -6.191824 -2.028642 1.944990
H -4.566085 -2.594832 2.366029
H -4.979660 -0.881679 2.557547
C -6.576077 -0.747937 -1.108601
H -7.358386 -1.206488 -0.491323
H -7.006520 0.135658 -1.594912
H -6.289059 -1.456385 -1.890782
C -5.794264 0.809881 0.686663
H -6.106265 1.670799 0.083960
H -6.637463 0.518810 1.323705
H -4.967478 1.128188 1.328834

171
(S)-alkenyl-full-ligand-Rct
Eopt -5154.504958

Ir 0.080443 -1.101439 -0.322020
P 0.215361 1.038199 -1.094592
P -1.923291 -0.885903 0.862370
N -0.136267 1.123207 -2.773689
O -0.725112 2.146047 -0.365497
O 1.683451 1.776243 -1.158182
O -2.899614 -2.192430 1.003851
O -2.894155 0.135303 0.000914
C 0.826202 0.465324 -3.639154
C -1.487447 0.831802 -3.209115
C -0.757295 3.477038 -0.825661
C 2.179554 2.648270 -0.181063
C -2.816865 -1.202066 3.469342
C -1.050377 0.410504 3.171106
C -3.783395 -2.666519 0.039392
C -4.220895 0.391194 0.353398
C 1.632182 1.237815 -4.476986
C 0.919196 -0.943629 -3.633059
C -2.205059 1.825046 -3.880747
C -2.033392 -0.451349 -3.018253
C 0.354274 4.293283 -0.698454
C -1.989189 3.921788 -1.350382
C 1.547746 3.855329 0.078865
C 3.392119 2.268590 0.438828
C -4.167129 -0.853871 3.560966
C -2.282886 -2.204189 4.308431
C -1.207170 1.783708 2.952495
C -0.074044 -0.063209 4.080202
C -4.880642 -1.919060 -0.370932
C -3.552556 -3.993484 -0.392450
C -5.192558 -0.593174 0.236443
C -4.506383 1.709049 0.776949
C 2.531248 0.614407 -5.344731
H 1.545566 2.319660 -4.441424
C 1.837864 -1.551493 -4.511185
C 0.084838 -1.793191 -2.767773
C -3.488661 1.556489 -4.357717
H -1.745322 2.796157 -4.030483
C -3.337489 -0.697087 -3.484775
C -1.248260 -1.557953 -2.450995
C 0.293611 5.588774 -1.335491
C -2.089931 5.200359 -1.843953
H -2.833006 3.241460 -1.355798
C 2.074262 4.664163 1.156229
C 3.952577 3.084240 1.390521
H 3.874363 1.342481 0.149459
C -5.024689 -1.559177 4.406577
H -4.548359 -0.041907 2.954728
C -3.172984 -2.931106 5.126434
C -0.847699 -2.434730 4.457856
C -0.379571 2.702588 3.598215
H -1.981432 2.126433 2.275657
C 0.767204 0.883661 4.699067
C 0.111278 -1.481051 4.397094
C -5.704725 -2.479225 -1.421053
C -4.399861 -4.570515 -1.305281
H -2.701678 -4.525428 0.016237
C -6.506944 -0.285341 0.756417
C -5.784582 2.041690 1.155138
H -3.704429 2.439372 0.784529
C 2.626368 -0.782305 -5.366134
H 3.158435 1.216066 -5.996879
H 1.918330 -2.635209 -4.525401
H 0.459788 -2.798073 -2.596431
C -4.056507 0.293235 -4.152085
H -4.042348 2.328643 -4.885283
H -3.771604 -1.681980 -3.344572
H -1.815975 -2.406700 -2.083485
C 1.435959 6.427869 -1.474643
C -0.948452 6.046510 -1.891894
H -3.035312 5.561765 -2.240742
C 1.405425 5.820746 1.651676
C 3.299165 4.278315 1.797148
H 4.890536 2.804138 1.862734
C -4.530076 -2.621813 5.172446
H -6.076134 -1.288657 4.453430
H -2.773023 -3.724730 5.753321
H -0.555845 -3.425939 4.800797
C 0.616591 2.249298 4.470707
H -0.508713 3.765867 3.416882

H	1.540351	0.528377	5.376201
H	1.119655	-1.760887	4.698196
C	-6.726792	-1.735487	-2.078946
C	-5.471772	-3.824231	-1.866820
H	-4.240214	-5.595651	-1.630226
C	-7.522320	-1.272182	0.916079
C	-6.804352	1.051807	1.189383
H	-6.019052	3.056389	1.467155
H	3.324588	-1.271165	-6.040011
H	-5.055580	0.077070	-4.520419
C	1.347234	7.657594	-2.095618
H	2.396262	6.087794	-1.103044
C	-1.012679	7.329949	-2.503860
C	1.935265	6.568003	2.684918
H	0.454486	6.115651	1.223028
C	3.830677	5.080069	2.845896
H	-5.195838	-3.191027	5.816064
H	1.271172	2.958796	4.969801
C	-7.497158	-2.299064	-3.076704
H	-6.894374	-0.699904	-1.806502
C	-6.296242	-4.384731	-2.882641
C	-8.765309	-0.947101	1.421043
H	-7.311235	-2.302529	0.654189
C	-8.104282	1.361720	1.679818
C	0.109358	8.124641	-2.602575
H	2.238217	8.271552	-2.200023
H	-1.965782	7.663829	-2.907885
C	3.168316	6.207644	3.281595
H	1.396280	7.440318	3.046373
H	4.766919	4.773535	3.307001
C	-7.295546	-3.643111	-3.475513
H	-8.262941	-1.701152	-3.564771
H	-6.108828	-5.410568	-3.192226
C	-9.071542	0.386066	1.789732
H	-9.514942	-1.725199	1.541608
H	-8.311456	2.385116	1.984524
H	0.051919	9.099432	-3.079891
H	3.578921	6.810446	4.087486
H	-7.915756	-4.078213	-4.254943
H	-10.058515	0.630400	2.173946
Cl	-0.097933	-3.546660	0.140132
C	1.440546	-0.793409	1.294015
H	1.685781	0.256338	1.432619
H	1.136053	-1.285422	2.209457
O	5.029122	-1.592298	-1.667964
C	6.559306	-3.732764	-1.529894
C	7.551655	-1.334220	-1.367874
H	5.738200	-4.440106	-1.300356
C	6.671498	-3.680562	-3.073690
C	7.818260	-4.300126	-0.827560
H	7.460554	-0.287756	-1.018292
C	7.642900	-1.266084	-2.913607
C	8.832328	-1.906811	-0.706747
H	6.937623	-4.671007	-3.487189
H	5.672369	-3.446911	-3.470091

C	7.668256	-2.636091	-3.632680
H	7.578188	-4.432802	0.239398
H	8.065511	-5.308886	-1.206031
C	9.083079	-3.417342	-0.933852
H	6.771128	-0.702278	-3.272457
H	8.533546	-0.692730	-3.230736
H	8.762812	-1.732550	0.376119
H	9.727485	-1.352717	-1.043815
H	7.459967	-2.481713	-4.703511
H	9.827998	-3.769215	-0.202788
H	8.683576	-3.047164	-3.594705
H	9.548130	-3.568955	-1.914469
B	6.234894	-2.222843	-0.943591
C	5.895480	-2.263795	0.662046
H	5.283743	-3.108869	1.012440
C	6.231032	-1.366024	1.615572
H	6.840440	-0.504506	1.328223
C	5.878192	-1.372828	3.051839
C	6.296111	-0.298650	3.866132
C	5.143915	-2.406786	3.673652
C	5.995093	-0.251084	5.230545
H	6.868794	0.509896	3.414795
C	4.843401	-2.363378	5.035802
H	4.809931	-3.257706	3.085698
C	5.264827	-1.284813	5.826296
H	6.333994	0.592688	5.828125
H	4.280119	-3.178304	5.486065
H	5.031222	-1.255618	6.887956
C	2.289967	-1.608922	0.447956
C	2.895731	-1.231720	-0.730865
H	2.367166	-2.659037	0.715734
H	2.916303	-0.195160	-1.046604
N	-1.935816	-0.528550	2.526170
C	3.754320	-2.167113	-1.544681
H	3.771318	-3.159172	-1.069856
H	3.307985	-2.295516	-2.543397

171
(S)-alkenyl-full-ligand-TS
Eopt -5154.491877

Ir	0.045428	-1.121555	-0.683171
P	0.333805	1.107119	-0.876415
P	-1.926860	-1.088777	0.628919
N	0.006211	1.641263	-2.485834
O	-0.593785	2.053443	0.078332
O	1.830604	1.786708	-0.725802
O	-3.009017	-2.325232	0.495206
O	-2.869672	0.175331	0.111526
C	0.900420	1.125282	-3.504801
C	-1.363572	1.560992	-2.957744
C	-0.649617	3.443277	-0.106884
C	2.298460	2.516595	0.369061
C	-2.772113	-1.974249	3.134638
C	-0.864010	-0.497238	3.117866
C	-3.936701	-2.479352	-0.526588
C	-4.159191	0.403577	0.582843

C	1.757640	2.001483	-4.172695
C	0.860088	-0.248333	-3.832490
C	-1.982172	2.727020	-3.417739
C	-2.023236	0.319439	-3.021549
C	0.463130	4.229236	0.137819
C	-1.901539	3.977316	-0.481596
C	1.662445	3.666568	0.816743
C	3.518867	2.066336	0.926070
C	-4.082111	-1.557691	3.388545
C	-2.301304	-3.195111	3.667061
C	-0.888085	0.895608	3.253075
C	0.111724	-1.265691	3.795429
C	-4.988993	-1.586426	-0.693191
C	-3.805323	-3.657393	-1.299630
C	-5.200691	-0.452653	0.252039
C	-4.343321	1.571789	1.358204
C	2.570195	1.529646	-5.206643
H	1.773513	3.047418	-3.880597
C	1.693492	-0.703668	-4.873516
C	-0.021084	-1.205827	-3.142680
C	-3.282416	2.677136	-3.921383
H	-1.433328	3.662124	-3.381644
C	-3.344417	0.292404	-3.505206
C	-1.331426	-0.945267	-2.722422
C	0.386407	5.619248	-0.247593
C	-2.017488	5.326369	-0.716875
H	-2.748597	3.308576	-0.580070
C	2.180215	4.294240	2.015036
C	4.080536	2.730709	1.987189
H	4.012910	1.207019	0.491931
C	-4.967379	-2.382235	4.084697
H	-4.411833	-0.593726	3.022283
C	-3.221166	-4.030045	4.335527
C	-0.890920	-3.577098	3.651723
C	0.068233	1.549051	4.031053
H	-1.660499	1.461148	2.743711
C	1.080953	-0.580701	4.556284
C	0.159686	-2.728109	3.743991
C	-5.862300	-1.795601	-1.827622
C	-4.701244	-3.918927	-2.306334
H	-2.986347	-4.329532	-1.072724
C	-6.478112	-0.217378	0.887121
C	-5.585677	1.866158	1.865705
H	-3.492833	2.222375	1.530442
C	2.527286	0.176828	-5.563466
H	3.234945	2.213440	-5.727576
H	1.675989	-1.757489	-5.140529
H	0.250933	-2.250476	-3.263593
C	-3.967894	1.456386	-3.952417
H	-3.758853	3.584780	-4.282620
H	-3.868398	-0.657034	-3.557096
H	-1.971552	-1.810010	-2.583171
C	1.527352	6.471088	-0.273336
C	-0.873788	6.169212	-0.660485
H	-2.977599	5.753505	-0.995974

C	1.491475	5.338288	2.698607
C	3.414006	3.833623	2.583317
H	5.029786	2.398625	2.399543
C	-4.541668	-3.636523	4.538287
H	-5.988093	-2.051138	4.256932
H	-2.871964	-4.985056	4.721481
H	-0.685915	-4.644098	3.723464
C	1.060659	0.806413	4.681134
H	0.041247	2.631017	4.125054
H	1.850419	-1.159098	5.061390
H	1.146260	-3.164697	3.888280
C	-6.840429	-0.841771	-2.232986
C	-5.726363	-2.984998	-2.620433
H	-4.615300	-4.829390	-2.894479
C	-7.557627	-1.144852	0.815112
C	-6.671518	0.968687	1.674470
H	-5.741607	2.770355	2.449056
H	3.156970	-0.196033	-6.366881
H	-4.982736	1.409258	-4.338379
C	1.422881	7.793088	-0.657217
H	2.498512	6.069039	-0.005502
C	-0.953419	7.542566	-1.026032
C	2.012462	5.916543	3.839321
H	0.531064	5.678717	2.329787
C	3.938938	4.464992	3.745493
H	-5.231000	-4.294205	5.061623
H	1.813530	1.308312	5.283272
C	-7.658291	-1.068939	-3.322130
H	-6.934463	0.090863	-1.688576
C	-6.598301	-3.201100	-3.724415
C	-8.763665	-0.892200	1.437625
H	-7.425287	-2.076686	0.277453
C	-7.935491	1.213967	2.281694
C	0.169266	8.342813	-1.023434
H	2.312795	8.416998	-0.682461
H	-1.919132	7.943789	-1.325317
C	3.257916	5.492051	4.363078
H	1.456405	6.703939	4.342174
H	4.884992	4.107313	4.145837
C	-7.551661	-2.267180	-4.070057
H	-8.387485	-0.316092	-3.611368
H	-6.484336	-4.116533	-4.300969
C	-8.967014	0.307422	2.163512
H	-9.563959	-1.625318	1.373347
H	-8.063093	2.127491	2.858346
H	0.099638	9.387865	-1.314170
H	3.662666	5.964022	5.254629
H	-8.208155	-2.438657	-4.919290
H	-9.925780	0.499475	2.638343
Cl	-0.319904	-3.593243	-0.834696
C	1.540521	-1.492152	0.819619
H	1.884096	-0.596991	1.332777
H	1.191036	-2.273755	1.486130
O	5.209765	-1.224635	-2.472028
C	6.810060	-3.225598	-1.902522

C	7.427009	-0.779147	-1.259637
H	6.034967	-4.005711	-2.027194
C	7.454169	-3.022493	-3.298737
C	7.792408	-3.752815	-0.828428
H	7.109753	0.220272	-0.904558
C	8.039308	-0.560965	-2.669126
C	8.456285	-1.309531	-0.228828
H	7.952973	-3.947853	-3.639718
H	6.637957	-2.835838	-4.010606
C	8.464425	-1.853977	-3.405210
H	7.212187	-3.995946	0.075607
H	8.255334	-4.703403	-1.148935
C	8.919789	-2.772014	-0.433839
H	7.288506	-0.041964	-3.280558
H	8.913479	0.112661	-2.616415
H	8.008760	-1.233029	0.772669
H	9.350990	-0.661687	-0.205105
H	8.626447	-1.620999	-4.469374
H	9.395780	-3.127992	0.493145
H	9.441230	-2.180308	-3.030811
H	9.708575	-2.803933	-1.193328
B	6.146237	-1.788578	-1.425024
C	5.231777	-2.080275	-0.039808
H	4.826906	-3.093606	0.055568
C	5.377602	-1.410058	1.149442
H	5.787926	-0.398932	1.118042
C	5.112603	-1.894439	2.503998
C	5.437084	-1.063328	3.600585
C	4.590834	-3.179444	2.780945
C	5.258453	-1.494839	4.915104
H	5.845848	-0.073932	3.407424
C	4.409632	-3.609044	4.094352
H	4.337940	-3.847924	1.963436
C	4.744716	-2.772177	5.168362
H	5.522654	-0.839096	5.741262
H	4.011767	-4.603039	4.284558
H	4.609054	-3.115199	6.191105
C	2.274346	-1.929390	-0.342829
C	3.347266	-1.246594	-0.965490
H	2.162010	-2.971009	-0.628420
H	3.522222	-0.207544	-0.716245
N	-1.873767	-1.160932	2.333859
C	3.908225	-1.670213	-2.298579
H	3.798816	-2.764728	-2.413923
H	3.272134	-1.205738	-3.067355

171

(S)-alkenyl-full-ligand-Pdt

Eopt -5154.583207

Ir	-0.048100	-1.011076	-0.869538
P	0.375856	1.159779	-0.547135
P	-2.174562	-1.220547	0.464596
N	0.129404	2.065879	-1.998172
O	-0.549893	1.882615	0.589712
O	1.888181	1.757019	-0.238643
O	-3.306210	-2.336888	-0.008574
O	-3.054324	0.178991	0.226592
C	1.101373	1.745233	-3.031562
C	-1.204963	2.055944	-2.568963
C	-0.615475	3.274677	0.708838
C	2.382435	2.331128	0.926547
C	-3.207996	-2.623487	2.668399
C	-1.245456	-1.267068	3.066622
C	-4.145629	-2.190855	-1.103876
C	-4.365539	0.344783	0.654092
C	1.948818	2.745808	-3.511409
C	1.148886	0.432417	-3.546479
C	-1.824288	3.280562	-2.833732
C	-1.813978	0.837525	-2.921527
C	0.484129	3.990585	1.145206
C	-1.863712	3.869281	0.421784
C	1.708041	3.307796	1.651422
C	3.696116	1.923698	1.270895
C	-4.521398	-2.213261	2.917103
C	-2.817220	-3.949596	2.962917
C	-1.193955	0.078638	3.448898
C	-0.346846	-2.203683	3.633161
C	-5.157424	-1.237464	-1.117720
C	-3.982715	-3.143217	-2.137738
C	-5.405863	-0.359552	0.062082
C	-4.574739	1.297225	1.679423
C	2.847883	2.462312	-4.542226
H	1.890201	3.739494	-3.076155
C	2.061732	0.169597	-4.585592
C	0.271006	-0.649942	-3.044665
C	-3.076454	3.312993	-3.449980
H	-1.311410	4.197861	-2.561897
C	-3.080423	0.891375	-3.532136
C	-1.131329	-0.461512	-2.739234
C	0.393388	5.433723	1.091463
C	-1.994601	5.234474	0.497344
H	-2.695230	3.231817	0.144029
C	2.259285	3.672865	2.940744
C	4.282002	2.371691	2.427749
H	4.238236	1.285011	0.583943
C	-5.481520	-3.123881	3.361649
H	-4.794491	-1.180740	2.741543
C	-3.809174	-4.863099	3.377519
C	-1.423057	-4.387718	2.964074
C	-0.238985	0.523004	4.364915
H	-1.904774	0.775607	3.018349
C	0.615720	-1.728700	4.546935
C	-0.352946	-3.628158	3.296546
C	-5.948622	-1.123695	-2.322970
C	-4.806187	-3.110263	-3.235791
H	-3.196510	-3.881590	-2.032796
C	-6.717520	-0.231217	0.655808
C	-5.840816	1.511066	2.168495
H	-3.721698	1.849189	2.059969
C	2.896521	1.171200	-5.082949
H	3.504473	3.241832	-4.919775

H	2.110380	-0.834095	-5.002382
H	0.526940	-1.624673	-3.451070
C	-3.705672	2.110810	-3.794912
H	-3.555282	4.266181	-3.659283
H	-3.566719	-0.037485	-3.817002
H	-1.765399	-1.313063	-2.967152
C	1.518356	6.285355	1.286805
C	-0.867373	6.051155	0.787880
H	-2.952231	5.706750	0.291948
C	1.537673	4.458477	3.885625
C	3.560244	3.205854	3.323258
H	5.295981	2.070258	2.679096
C	-5.129277	-4.462662	3.569044
H	-6.502011	-2.789840	3.529816
H	-3.518147	-5.892144	3.576244
H	-1.260774	-5.456096	2.830108
C	0.671838	-0.385894	4.915039
H	-0.206378	1.572132	4.645409
H	1.325306	-2.436580	4.969388
H	0.604940	-4.133726	3.409560
C	-6.873534	-0.061643	-2.541419
C	-5.784916	-2.088207	-3.374142
H	-4.696338	-3.848872	-4.026298
C	-7.814089	-1.065209	0.290619
C	-6.934351	0.733864	1.697833
H	-6.012021	2.251130	2.946546
H	3.588152	0.944666	-5.890556
H	-4.679658	2.123155	-4.277149
C	1.396925	7.659333	1.223055
H	2.493380	5.848380	1.470545
C	-0.965744	7.470761	0.755057
C	2.080511	4.784630	5.113102
H	0.533717	4.791809	3.647745
C	4.104497	3.578961	4.583585
H	-5.875005	-5.183383	3.894771
H	1.421330	-0.047982	5.626045
C	-7.619460	0.015426	-3.700921
H	-6.983490	0.713234	-1.791270
C	-6.582408	-1.993822	-4.549286
C	-9.053750	-0.920766	0.880806
H	-7.669651	-1.841084	-0.452465
C	-8.230649	0.876467	2.268889
C	0.140762	8.263930	0.972010
H	2.276745	8.282510	1.362737
H	-1.933369	7.917485	0.537197
C	3.383688	4.356439	5.465300
H	1.497558	5.373203	5.817337
H	5.099492	3.223799	4.842847
C	-7.488565	-0.967592	-4.712500
H	-8.309992	0.843715	-3.840277
H	-6.449505	-2.744350	-5.325589
C	-9.275244	0.071607	1.867564
H	-9.867100	-1.580144	0.587934
H	-8.374222	1.625292	3.044906
H	0.056765	9.347041	0.935577

H	3.804062	4.629530	6.429807
H	-8.088580	-0.901404	-5.616551
H	-10.259436	0.182634	2.315503
Cl	-0.498964	-3.392155	-1.583472
C	1.114881	-1.955364	0.755477
H	1.355057	-1.303881	1.591109
H	0.556803	-2.849344	1.009242
O	5.176758	-0.182112	-1.402502
C	6.642815	-1.655302	-3.060392
C	7.651066	0.336651	-1.687830
H	5.771388	-2.276777	-3.308405
C	7.087419	-0.948472	-4.377951
C	7.717997	-2.600147	-2.443019
H	7.454993	1.130346	-0.951286
C	8.089045	1.024792	-3.014687
C	8.709679	-0.625775	-1.075836
H	7.377265	-1.704107	-5.125030
H	6.206999	-0.434320	-4.792262
C	8.232482	0.079311	-4.229262
H	7.242457	-3.152078	-1.618432
H	8.014701	-3.358990	-3.184198
C	8.986428	-1.904560	-1.898283
H	7.336378	1.791461	-3.254944
H	9.036817	1.566125	-2.865409
H	8.352146	-0.922301	-0.077924
H	9.658615	-0.090533	-0.912204
H	8.280691	0.685778	-5.145217
H	9.534255	-2.618966	-1.267156
H	9.191110	-0.445835	-4.175298
H	9.660706	-1.669004	-2.727520
B	6.354457	-0.509956	-2.006472
C	4.277764	-2.369549	0.353455
H	4.444296	-3.251389	-0.267109
C	4.843176	-2.279429	1.570319
H	4.655672	-1.380561	2.159310
C	5.714393	-3.270799	2.227896
C	6.279997	-2.949107	3.477884
C	6.013384	-4.534006	1.675000
C	7.115806	-3.845892	4.149515
H	6.059552	-1.981128	3.923705
C	6.847363	-5.429836	2.344356
H	5.589351	-4.821724	0.716692
C	7.404751	-5.092227	3.585410
H	7.539314	-3.570285	5.112546
H	7.062184	-6.397918	1.897622
H	8.053299	-5.793908	4.104071
C	1.989505	-1.968105	-0.352534
C	3.364321	-1.321197	-0.249902
H	1.964104	-2.841818	-0.999163
H	3.302670	-0.477166	0.438021
N	-2.232195	-1.704378	2.113863
C	3.910373	-0.830622	-1.595580
H	4.000509	-1.662819	-2.302831
H	3.234586	-0.087280	-2.018220

171

(S)-alkenyl-full-ligand-Rct
Eopt -5154.505641

C	1.608685	0.341052	1.413144
H	1.923182	-0.533262	1.977354
H	1.124783	1.096957	2.024711
O	5.423026	-0.276831	-1.656126
C	7.802047	0.793380	-1.267854
C	7.527728	-1.682412	-2.015620
H	7.441104	1.655530	-0.672404
C	7.785302	1.249181	-2.747711
C	9.217107	0.469251	-0.726103
H	6.964348	-2.633675	-1.965208
C	7.479230	-1.229693	-3.496990
C	8.962327	-1.995089	-1.518369
H	8.472525	2.100114	-2.910326
H	6.776908	1.631264	-2.964797
C	8.118332	0.149567	-3.785828
H	9.133057	0.300838	0.359362
H	9.895943	1.333872	-0.842798
C	9.893471	-0.769425	-1.356919
H	6.422754	-1.192216	-3.796095
H	7.959425	-1.977895	-4.154225
H	8.881622	-2.492351	-0.541474
H	9.459221	-2.722611	-2.185826
H	7.796690	0.491317	-4.782676
H	10.759456	-1.057327	-0.740185
H	9.206153	0.037376	-3.858937
H	10.312257	-0.495272	-2.331701
B	6.828612	-0.528692	-1.078326
C	6.658494	-0.919736	0.510682
H	6.628613	-0.086316	1.229681
C	6.498472	-2.148167	1.051740
H	6.508863	-3.014608	0.384329
C	6.286817	-2.511256	2.469930
C	6.028708	-3.858206	2.801392
C	6.332461	-1.581181	3.531789
C	5.817281	-4.260681	4.123403
H	5.990323	-4.597246	2.002971
C	6.123711	-1.979990	4.852816
H	6.540329	-0.535145	3.321462
C	5.862879	-3.322858	5.160342
H	5.618330	-5.307729	4.342269
H	6.169008	-1.240042	5.649473
H	5.702385	-3.630596	6.190931
C	2.558301	0.850632	0.426592
C	3.568938	0.106192	-0.139591
H	2.500907	1.896856	0.146338
H	3.671675	-0.943384	0.119173
Ir	0.543993	-0.290565	-0.303105
P	-0.420594	1.726552	-0.758859
P	-1.294077	-1.329364	0.663217
N	-0.639760	1.935713	-2.452642
O	-1.851863	2.045240	-0.058909
O	0.441466	3.108771	-0.504196
N	-1.466556	-1.386853	2.353224
O	-1.516114	-2.938702	0.454753
O	-2.646056	-0.742020	-0.081951
C	0.601433	1.991078	-3.204161
C	-1.616517	1.106830	-3.128586
C	-2.558481	3.226525	-0.350909
C	0.327212	3.929832	0.621990
C	-1.904006	-2.577454	3.066208
C	-1.095494	-0.310295	3.236623
C	-2.053409	-3.547002	-0.673850
C	-3.922806	-1.235010	0.190159
C	0.982510	3.193155	-3.803229
C	1.402802	0.832750	-3.317911
C	-2.682646	1.729836	-3.783121
C	-1.457803	-0.292038	-3.175015
C	-2.063758	4.455725	0.049395
C	-3.801740	3.065129	-0.998855
C	-0.870310	4.556961	0.935621
C	1.518946	4.161970	1.347867
C	-3.256593	-2.926476	3.110443
C	-0.941724	-3.371628	3.727381
C	-1.873867	0.851756	3.284351
C	0.022414	-0.462897	4.090804
C	-3.380145	-3.351808	-1.037986
C	-1.196880	-4.453723	-1.340660
C	-4.294530	-2.510266	-0.213251
C	-4.803883	-0.345837	0.846459
C	2.165694	3.258597	-4.542241
H	0.347031	4.065583	-3.683252
C	2.605060	0.928089	-4.047116
C	1.023498	-0.464570	-2.732449
C	-3.619539	0.964656	-4.478527
H	-2.762321	2.811147	-3.745529
C	-2.428499	-1.049566	-3.857599
C	-0.269228	-0.967620	-2.637469
C	-2.764562	5.630381	-0.417108
C	-4.535898	4.177140	-1.334670
H	-4.152963	2.064185	-1.221475
C	-0.925859	5.308751	2.170298
C	1.497472	4.968400	2.458797
H	2.445622	3.715563	1.007222
C	-3.662176	-4.111327	3.726581
H	-3.988585	-2.278042	2.645372
C	-1.367276	-4.583443	4.310713
C	0.441247	-2.943844	3.927847
C	-1.539875	1.891475	4.152720
H	-2.740615	0.933114	2.636906
C	0.351025	0.610589	4.943750
C	0.851718	-1.668848	4.122301
C	-3.830167	-3.988212	-2.256960
C	-1.656698	-5.145333	-2.433869
H	-0.188660	-4.580518	-0.962849
C	-5.584776	-2.990903	0.229346
C	-6.076311	-0.757547	1.161563
H	-4.455827	0.654053	1.083028
C	2.974977	2.122344	-4.664671

H	2.459112	4.193662	-5.011770
H	3.239767	0.050406	-4.136009
H	1.832546	-1.183873	-2.642340
C	-3.494320	-0.429742	-4.507228
H	-4.443712	1.453077	-4.991627
H	-2.320206	-2.128823	-3.899731
H	-0.361216	-2.037794	-2.482452
C	-2.240552	6.946209	-0.267889
C	-4.020775	5.480728	-1.096301
H	-5.500841	4.068911	-1.823597
C	-2.143872	5.814872	2.709499
C	0.276695	5.528323	2.921876
H	2.413878	5.162253	3.010509
C	-2.709016	-4.956801	4.307132
H	-4.715559	-4.378211	3.737762
H	-0.626846	-5.215512	4.795529
H	1.169889	-3.738312	4.078790
C	-0.419147	1.770537	4.983217
H	-2.148847	2.790652	4.179215
H	1.221574	0.515490	5.588686
H	1.887275	-1.515339	4.421824
C	-5.094102	-3.707775	-2.852082
C	-2.965850	-4.911686	-2.937195
H	-1.012109	-5.857392	-2.943429
C	-5.991343	-4.349013	0.086062
C	-6.486933	-2.092251	0.894759
H	-6.768856	-0.077512	1.651368
H	3.900376	2.169431	-5.232224
H	-4.221449	-1.032428	-5.044393
C	-2.931971	8.047426	-0.731742
H	-1.273274	7.085758	0.201952
C	-4.720812	6.637603	-1.540512
C	-2.163120	6.524728	3.893768
H	-3.078010	5.627114	2.192377
C	0.228810	6.284156	4.126579
H	-3.014123	-5.892147	4.769264
H	-0.150037	2.578234	5.658777
C	-5.495975	-4.334842	-4.014776
H	-5.748969	-2.974795	-2.395215
C	-3.416825	-5.558371	-4.122157
C	-7.228422	-4.777207	0.524157
H	-5.310787	-5.066226	-0.358162
C	-7.766293	-2.557681	1.311059
C	-4.192631	7.898129	-1.360684
H	-2.501876	9.039101	-0.616173
H	-5.677233	6.503313	-2.040878
C	-0.965219	6.779158	4.605598
H	-3.109962	6.888369	4.285085
H	1.156284	6.450258	4.670058
C	-4.659570	-5.283991	-4.651884
H	-6.463019	-4.091813	-4.448292
H	-2.749585	-6.264792	-4.610849
C	-8.137269	-3.872068	1.125698
H	-7.504711	-5.822526	0.410652
H	-8.439259	-1.854635	1.796992
H	-4.731020	8.774811	-1.711335
H	-0.993206	7.349239	5.530564
H	-4.992115	-5.778702	-5.560846
H	-9.112947	-4.220211	1.454834
Cl	1.605314	-2.532731	-0.107036
C	4.609559	0.674479	-1.057643
H	4.092582	1.274287	-1.829356
H	5.181428	1.418205	-0.467049

171

(R)-alkenyl-full-ligand-TS

Eopt -5154.501193

C	1.713615	-0.854317	0.953009
H	1.645119	-1.777433	1.522160
H	1.739323	0.039470	1.569843
O	4.888292	-2.845036	-2.259481
C	6.689331	-0.943079	-1.886865
C	7.199927	-3.432956	-1.328730
H	5.975658	-0.098234	-1.921245
C	7.170670	-1.166691	-3.342454
C	7.815649	-0.504750	-0.915205
H	6.836892	-4.412205	-0.964625
C	7.680949	-3.659945	-2.785454
C	8.328707	-2.992649	-0.364618
H	7.677840	-0.266535	-3.734607
H	6.277022	-1.308169	-3.967662
C	8.104381	-2.383225	-3.550385
H	7.347710	-0.211244	0.037749
H	8.322622	0.404296	-1.285786
C	8.891337	-1.573884	-0.613691
H	6.855606	-4.134566	-3.334306
H	8.520724	-4.377819	-2.813956
H	7.931964	-3.031628	0.661719
H	9.168524	-3.709847	-0.387714
H	8.155774	-2.613133	-4.626239
H	9.472012	-1.258247	0.267244
H	9.126570	-2.109260	-3.265742
H	9.612320	-1.607086	-1.437737
B	5.982217	-2.333372	-1.346231
C	5.262802	-2.094904	0.146869
H	5.302653	-1.100489	0.603009
C	4.965741	-3.118588	1.011864
H	4.903840	-4.126649	0.595277
C	4.756051	-3.069733	2.458694
C	4.442567	-4.266970	3.141023
C	4.899329	-1.889004	3.222610
C	4.279668	-4.287769	4.526751
H	4.332089	-5.185699	2.568811
C	4.741181	-1.911362	4.607058
H	5.150612	-0.954704	2.729139
C	4.430924	-3.109264	5.266791
H	4.039685	-5.221221	5.030078
H	4.864763	-0.993930	5.177566
H	4.311553	-3.122411	6.347352
C	2.541079	-0.869363	-0.232889
C	3.152257	-2.031616	-0.773864

H	2.921542	0.071651	-0.613141
H	2.830599	-2.993771	-0.395916
Ir	0.221645	-0.826839	-0.578155
P	0.251128	1.402173	-0.898633
P	-1.803721	-0.990072	0.636965
N	-0.151073	1.833255	-2.518089
O	-0.703802	2.310601	0.062158
O	1.691272	2.209700	-0.853911
N	-1.816666	-0.987429	2.343575
O	-2.722371	-2.353821	0.520801
O	-2.875286	0.129853	0.035995
C	0.822453	1.400654	-3.504381
C	-1.497629	1.564940	-2.981738
C	-0.803076	3.700251	-0.114845
C	2.191732	2.898592	0.250555
C	-2.627527	-1.887683	3.144121
C	-0.922658	-0.183667	3.135873
C	-3.579772	-2.660738	-0.526004
C	-4.200484	0.209651	0.454378
C	1.582244	2.357366	-4.180345
C	0.985036	0.021709	-3.764388
C	-2.273976	2.636039	-3.433437
C	-1.983725	0.244976	-3.036866
C	0.278046	4.523477	0.152876
C	-2.067813	4.189132	-0.507782
C	1.518519	3.989192	0.782926
C	3.463085	2.488939	0.716705
C	-3.986089	-1.634228	3.353718
C	-2.020067	-3.024900	3.721207
C	-1.132916	1.196072	3.240954
C	0.126871	-0.805804	3.851440
C	-4.733660	-1.920872	-0.756120
C	-3.261772	-3.831455	-1.254444
C	-5.114552	-0.786091	0.134221
C	-4.557785	1.376855	1.168613
C	2.510082	1.956895	-5.144034
H	1.439193	3.407153	-3.941497
C	1.933279	-0.360157	-4.734189
C	0.198571	-1.016758	-3.076483
C	-3.556889	2.408759	-3.933139
H	-1.859299	3.637949	-3.395282
C	-3.286676	0.037311	-3.525436
C	-1.140083	-0.914257	-2.704071
C	0.145448	5.919474	-0.198154
C	-2.235539	5.535833	-0.722039
H	-2.883972	3.487268	-0.636083
C	2.060060	4.585334	1.984336
C	4.035556	3.124230	1.790690
H	3.981055	1.687120	0.203471
C	-4.777887	-2.545647	4.054403
H	-4.425354	-0.730691	2.949533
C	-2.844124	-3.951282	4.393895
C	-0.572519	-3.226394	3.741129
C	-0.290894	1.983221	4.026705
H	-1.960399	1.644998	2.702260
C	0.977760	0.014047	4.621099
C	0.361475	-2.250561	3.831987
C	-5.536886	-2.286417	-1.903007
C	-4.081559	-4.244782	-2.274987
H	-2.364611	-4.375502	-0.982322
C	-6.431423	-0.688657	0.723435
C	-5.843291	1.531695	1.627952
H	-3.802246	2.137809	1.333303
C	2.680593	0.594771	-5.422211
H	3.100561	2.702326	-5.670042
H	2.072805	-1.416131	-4.949869
H	0.613261	-2.019312	-3.131555
C	-4.064593	1.104331	-3.972093
H	-4.156107	3.243138	-4.288186
H	-3.674821	-0.974931	-3.576487
H	-1.665649	-1.847908	-2.533275
C	1.247705	6.821606	-0.187969
C	-1.131260	6.425424	-0.617758
H	-3.206598	5.927958	-1.014283
C	1.357514	5.567368	2.740684
C	3.338446	4.156099	2.475471
H	5.019444	2.824052	2.142606
C	-4.208626	-3.722667	4.555300
H	-5.836648	-2.343128	4.193104
H	-2.388128	-4.844294	4.815331
H	-0.236229	-4.257229	3.839754
C	0.772940	1.388630	4.715892
H	-0.461888	3.053773	4.097811
H	1.800124	-0.450042	5.159730
H	1.391332	-2.557426	4.003391
C	-6.622930	-1.490596	-2.370045
C	-5.216710	-3.472948	-2.646200
H	-3.852920	-5.151581	-2.829755
C	-7.381481	-1.749290	0.665277
C	-6.799729	0.495072	1.449283
H	-6.131142	2.432580	2.164426
H	3.402014	0.276624	-6.170164
H	-5.062415	0.917854	-4.359971
C	1.089645	8.147913	-0.537321
H	2.233360	6.457717	0.080210
C	-1.267163	7.803596	-0.946752
C	1.901051	6.115398	3.885663
H	0.369594	5.881305	2.423393
C	3.882404	4.755160	3.646042
H	-4.822721	-4.448113	5.082841
H	1.436533	1.995982	5.325951
C	-7.370574	-1.866762	-3.468380
H	-6.858768	-0.560616	-1.865761
C	-6.018920	-3.845944	-3.761236
C	-8.630686	-1.626342	1.239874
H	-7.113920	-2.678874	0.176068
C	-8.104700	0.602374	2.008082
C	-0.181913	8.652654	-0.905327
H	1.951756	8.810208	-0.533895
H	-2.245726	8.169559	-1.249485

C	3.183287	5.720119	4.339349
H	1.335456	6.855538	4.446376
H	4.859456	4.423651	3.990734
C	-7.080444	-3.064979	-4.165852
H	-8.186918	-1.231810	-3.804069
H	-5.764110	-4.756573	-4.299034
C	-9.009015	-0.432422	1.902221
H	-9.329064	-2.457948	1.187348
H	-8.366689	1.515480	2.538242
H	-0.294173	9.701478	-1.167715
H	3.604295	6.167240	5.236175
H	-7.682486	-3.354887	-5.023238
H	-10.000531	-0.343807	2.338767
Cl	0.209890	-3.332159	-0.557178
C	3.753755	-2.046317	-2.162681
H	2.974217	-2.461889	-2.823907
H	3.936182	-1.008558	-2.485942

171

(R)-alkenyl-full-ligand-Pdt

Eopt -5154.594675

C	1.562167	-0.074675	1.268830
H	1.564625	-0.815612	2.061743
H	1.298497	0.928917	1.587493
O	5.071547	-2.743219	-1.018094
C	7.078687	-1.566466	-2.303430
C	7.269830	-3.987328	-1.317051
H	6.478327	-0.658141	-2.450363
C	7.388668	-2.139990	-3.720762
C	8.338086	-1.154138	-1.483175
H	6.764466	-4.785822	-0.753432
C	7.579215	-4.539347	-2.740238
C	8.522663	-3.553412	-0.502978
H	7.969867	-1.405453	-4.300301
H	6.429967	-2.253124	-4.250253
C	8.126122	-3.498417	-3.743592
H	7.988624	-0.632545	-0.579180
H	8.926697	-0.416140	-2.050892
C	9.264713	-2.313464	-1.051302
H	6.644791	-4.958466	-3.144642
H	8.285505	-5.382186	-2.674380
H	8.192667	-3.337419	0.524554
H	9.232682	-4.392077	-0.423995
H	8.060059	-3.916501	-4.758442
H	9.952665	-1.942624	-0.277916
H	9.193345	-3.338446	-3.561835
H	9.900765	-2.607159	-1.892224
B	6.344797	-2.718696	-1.502546
C	4.638086	-0.792397	1.148214
H	5.129524	0.112901	0.786903
C	5.008765	-1.342409	2.318295
H	4.497291	-2.250376	2.641445
C	6.049892	-0.861857	3.245454
C	6.294557	-1.593543	4.424759
C	6.820764	0.298117	3.019105
C	7.267524	-1.188124	5.342991
H	5.712198	-2.492101	4.618883
C	7.792447	0.703607	3.934260
H	6.662291	0.888083	2.120400
C	8.022321	-0.035910	5.102736
H	7.434836	-1.772942	6.244493
H	8.374107	1.601023	3.735916
H	8.780380	0.283677	5.813472
C	2.446483	-0.272473	0.179848
C	3.543191	-1.339840	0.256747
H	2.740316	0.597118	-0.401486
H	3.137569	-2.249404	0.706081
Ir	0.279589	-0.720894	-0.403033
P	-0.043891	1.350587	-1.163165
P	-1.755431	-1.016329	0.965736
N	-0.320775	1.374219	-2.861929
O	-1.264004	2.217706	-0.503612
O	1.202360	2.439823	-1.161258
N	-1.812250	-0.774220	2.663900
O	-2.451028	-2.516949	1.058663
O	-2.994846	-0.187681	0.210735
C	0.838846	0.945141	-3.626769
C	-1.512391	0.682400	-3.323288
C	-1.594267	3.486038	-1.000527
C	1.409197	3.413888	-0.187419
C	-2.494231	-1.654233	3.593494
C	-1.088105	0.288237	3.310446
C	-3.194182	-3.102939	0.045940
C	-4.325020	-0.279087	0.598297
C	1.433718	1.836316	-4.522012
C	1.325613	-0.368795	-3.460863
C	-2.423185	1.387158	-4.114898
C	-1.698933	-0.683138	-3.032714
C	-0.733645	4.556958	-0.823541
C	-2.858153	3.606005	-1.619083
C	0.485074	4.427920	0.024404
C	2.649506	3.363120	0.491654
C	-3.884410	-1.612074	3.734756
C	-1.733162	-2.560867	4.365261
C	-1.496527	1.616256	3.139123
C	0.014107	-0.017702	4.144374
C	-4.446906	-2.612793	-0.304554
C	-2.653034	-4.285910	-0.510965
C	-5.047659	-1.451479	0.414151
C	-4.901038	0.895843	1.137268
C	2.522627	1.425545	-5.294552
H	1.033257	2.842697	-4.606636
C	2.420424	-0.762106	-4.254500
C	0.721777	-1.328228	-2.505402
C	-3.551593	0.743987	-4.626778
H	-2.232101	2.434207	-4.328360
C	-2.848499	-1.308978	-3.549446
C	-0.696925	-1.476701	-2.287572
C	-1.072929	5.794013	-1.488584
C	-3.240018	4.814703	-2.149109
H	-3.503559	2.735780	-1.664391

C	0.737182	5.344236	1.114483
C	2.943135	4.297255	1.453666
H	3.357998	2.584464	0.233529
C	-4.543824	-2.520726	4.564527
H	-4.450215	-0.878057	3.175050
C	-2.419486	-3.492795	5.171395
C	-0.274539	-2.506308	4.445179
C	-0.808417	2.657806	3.763526
H	-2.356152	1.826474	2.511362
C	0.702650	1.052038	4.751180
C	0.480411	-1.384473	4.384818
C	-5.132536	-3.263145	-1.399399
C	-3.352243	-4.970737	-1.473829
H	-1.685166	-4.621301	-0.155396
C	-6.379676	-1.504244	0.973518
C	-6.207591	0.886886	1.561916
H	-4.292626	1.792327	1.198508
C	3.010522	0.119647	-5.161493
H	2.984313	2.117301	-5.994261
H	2.804197	-1.774731	-4.157305
H	1.291024	-2.247827	-2.410351
C	-3.763786	-0.608784	-4.335990
H	-4.256912	1.292838	-5.245528
H	-3.009610	-2.362919	-3.341270
H	-1.021921	-2.489723	-2.071310
C	-0.172369	6.894668	-1.568620
C	-2.347614	5.921914	-2.137413
H	-4.214830	4.924483	-2.617773
C	-0.228072	6.291485	1.563496
C	1.987822	5.285190	1.816200
H	3.899055	4.269320	1.970721
C	-3.809067	-3.482209	5.268338
H	-5.627023	-2.485951	4.646741
H	-1.839144	-4.211889	5.745289
H	0.221774	-3.434471	4.724112
C	0.298030	2.373253	4.572172
H	-1.132864	3.684336	3.617415
H	1.565799	0.829151	5.374447
H	1.539089	-1.478551	4.622209
C	-6.321787	-2.740207	-1.985256
C	-4.589638	-4.467946	-1.962114
H	-2.950783	-5.889683	-1.894433
C	-7.133766	-2.710858	1.055428
C	-6.966877	-0.314565	1.525006
H	-6.662174	1.789046	1.964125
H	3.852615	-0.211682	-5.764134
H	-4.637790	-1.121340	-4.729379
C	-0.523360	8.060564	-2.219537
H	0.813949	6.809534	-1.126010
C	-2.689397	7.145777	-2.778821
C	0.042058	7.149819	2.611034
H	-1.200781	6.331060	1.086540
C	2.245337	6.197053	2.877787
H	-4.315879	-4.207217	5.900197
H	0.842058	3.178774	5.058454
C	-6.957858	-3.389980	-3.024474
H	-6.728445	-1.803531	-1.621479
C	-5.279003	-5.125804	-3.019481
C	-8.402164	-2.729605	1.600238
H	-6.697713	-3.637273	0.699490
C	-8.286726	-0.360467	2.056551
C	-1.799890	8.198362	-2.818534
H	0.189421	8.879553	-2.276016
H	-3.665106	7.226892	-3.252822
C	1.296169	7.117381	3.268699
H	-0.719391	7.854023	2.937160
H	3.205355	6.140973	3.386230
C	-6.444808	-4.605488	-3.540142
H	-7.858614	-2.959184	-3.455009
H	-4.855214	-6.044259	-3.420027
C	-8.997624	-1.540983	2.090244
H	-8.947019	-3.668692	1.658120
H	-8.716342	0.556775	2.453583
H	-2.067225	9.125374	-3.319283
H	1.499576	7.804839	4.085739
H	-6.960070	-5.112726	-4.351877
H	-10.001819	-1.567372	2.505580
Cl	0.726878	-3.128787	0.159231
C	4.101625	-1.691945	-1.128114
H	3.298096	-2.069743	-1.760643
H	4.537895	-0.807925	-1.606348

173
(S)-alkyl-full-ligand-Rct
Eopt -5155.712540

Ir	0.094206	-0.886358	-0.514116
P	-0.004967	1.382613	-0.798091
P	-1.827698	-1.126710	0.723965
N	-0.319658	1.775184	-2.445312
O	-1.074197	2.228689	0.087025
O	1.387107	2.241925	-0.640451
O	-2.680529	-2.514242	0.576705
O	-2.906148	-0.032129	0.124935
C	0.705970	1.340849	-3.375905
C	-1.642255	1.534367	-2.981950
C	-1.235162	3.615725	-0.104395
C	1.749137	2.952277	0.510673
C	-2.600596	-2.097559	3.198989
C	-0.983137	-0.309824	3.251944
C	-3.542452	-2.843086	-0.465705
C	-4.241017	0.012672	0.534459
C	1.529570	2.293837	-3.978116
C	0.860630	-0.036448	-3.654630
C	-2.380744	2.619847	-3.461243
C	-2.149117	0.222790	-3.071498
C	-0.212119	4.494485	0.211194
C	-2.502323	4.036579	-0.560774
C	0.997188	4.032482	0.947876
C	2.959234	2.563089	1.126883
C	-3.971859	-1.898226	3.380460
C	-1.959355	-3.211460	3.782758

C	-1.262872	1.057263	3.355458
C	0.056394	-0.889798	4.017898
C	-4.705915	-2.120798	-0.701113
C	-3.210026	-4.018421	-1.178609
C	-5.123303	-1.002280	0.192407
C	-4.630149	1.162799	1.257250
C	2.516104	1.890904	-4.880420
H	1.392518	3.341776	-3.728877
C	1.874510	-0.421669	-4.554942
C	0.011160	-1.073101	-3.046931
C	-3.647439	2.416310	-4.009926
H	-1.951504	3.614321	-3.403460
C	-3.443378	0.040285	-3.595778
C	-1.334892	-0.953176	-2.752223
C	-0.391728	5.875873	-0.171609
C	-2.723331	5.371526	-0.801270
H	-3.278471	3.295970	-0.716054
C	1.405126	4.673998	2.178937
C	3.409062	3.239071	2.233947
H	3.528741	1.742824	0.708362
C	-4.741365	-2.847323	4.054727
H	-4.438074	-1.008463	2.976119
C	-2.760802	-4.177877	4.426006
C	-0.505923	-3.343768	3.856176
C	-0.500377	1.874086	4.190740
H	-2.083106	1.474551	2.782220
C	0.828614	-0.038362	4.833804
C	0.369036	-2.320754	3.999834
C	-5.493861	-2.498615	-1.854966
C	-4.018749	-4.445947	-2.202115
H	-2.312775	-4.555313	-0.895045
C	-6.448951	-0.943872	0.768245
C	-5.923909	1.279877	1.704456
H	-3.896083	1.940443	1.437012
C	2.684346	0.531180	-5.170557
H	3.158515	2.633322	-5.345920
H	2.010960	-1.477154	-4.775752
H	0.418688	-2.080010	-3.069517
C	-4.183390	1.123527	-4.064923
H	-4.215562	3.262632	-4.386774
H	-3.847868	-0.964533	-3.664504
H	-1.874802	-1.873789	-2.554887
C	0.666457	6.827703	-0.120382
C	-1.667675	6.313794	-0.663259
H	-3.697595	5.712376	-1.142722
C	0.611895	5.657856	2.837472
C	2.638047	4.287094	2.803882
H	4.349136	2.955899	2.700289
C	-4.136669	-4.006374	4.556218
H	-5.809736	-2.687459	4.172988
H	-2.277966	-5.053862	4.852938
H	-0.122232	-4.357143	3.960274
C	0.553200	1.323456	4.929550
H	-0.725558	2.934387	4.261497
H	1.646029	-0.468380	5.407808

H	1.406554	-2.572355	4.214601
C	-6.583033	-1.714530	-2.333976
C	-5.154032	-3.683648	-2.591174
H	-3.779304	-5.356114	-2.746472
C	-7.371943	-2.026431	0.688109
C	-6.852509	0.222415	1.503405
H	-6.239432	2.167172	2.247769
H	3.456372	0.213537	-5.865981
H	-5.172103	0.959171	-4.484784
C	0.465913	8.138867	-0.502844
H	1.651835	6.513355	0.205841
C	-1.849465	7.678640	-1.024552
C	1.033094	6.251149	4.011032
H	-0.348246	5.939561	2.420742
C	3.056109	4.932382	4.001377
H	-4.732812	-4.761167	5.062537
H	1.155648	1.954930	5.577011
C	-7.313332	-2.099274	-3.440779
H	-6.834493	-0.786745	-1.833112
C	-5.939257	-4.066105	-3.715094
C	-8.629380	-1.939703	1.251096
H	-7.077712	-2.943718	0.191322
C	-8.165236	0.292140	2.049735
C	-0.807023	8.577087	-0.944769
H	1.295186	8.840911	-0.468270
H	-2.826980	7.994248	-1.382342
C	2.275346	5.901100	4.594617
H	0.400786	6.992481	4.493407
H	4.001546	4.631564	4.447203
C	-7.002813	-3.295314	-4.133283
H	-8.132081	-1.473177	-3.786841
H	-5.670147	-4.975305	-4.248089
C	-9.042902	-0.762843	1.922592
H	-9.307114	-2.786939	1.182163
H	-8.454441	1.192473	2.587280
H	-0.953103	9.615011	-1.232521
H	2.600091	6.384332	5.512486
H	-7.591672	-3.591773	-4.997488
H	-10.040692	-0.703132	2.349486
Cl	0.191520	-3.373889	-0.618945
C	1.521787	-0.892617	1.067574
H	1.685349	0.113018	1.448974
H	1.238059	-1.586048	1.851159
C	4.179707	-1.488734	-1.748338
H	3.688585	-1.491789	-2.738294
H	4.241040	-2.544119	-1.434989
O	5.433446	-0.897400	-1.911760
C	7.971035	-0.642709	-1.543445
C	6.358295	0.449970	0.180838
H	8.202036	-1.467458	-2.240765
C	8.014301	0.689884	-2.362295
C	9.090236	-0.641172	-0.468483
H	5.408894	0.424328	0.747673
C	6.391390	1.783981	-0.639522
C	7.497220	0.459926	1.235251

H	8.956130	1.226565	-2.154600
H	8.043376	0.468773	-3.440210
C	6.825194	1.644089	-2.116345
H	9.126346	-1.622783	0.026943
H	10.081786	-0.502070	-0.936869
C	8.901759	0.451837	0.601497
H	5.398340	2.256930	-0.636017
H	7.053243	2.511458	-0.138459
H	7.397303	-0.411923	1.897942
H	7.415961	1.346683	1.890744
H	5.969898	1.262763	-2.681881
H	9.665900	0.342367	1.386825
H	7.061139	2.640851	-2.524005
H	9.088661	1.433311	0.141603
B	6.509006	-0.885832	-0.801997
C	6.629211	-4.873411	0.063862
C	5.429444	-5.607211	0.042278
C	7.667488	-5.343951	0.888714
C	5.266878	-6.760149	0.818942
H	4.612554	-5.271655	-0.594546
C	7.512967	-6.496005	1.667117
H	8.609933	-4.799296	0.918279
C	6.309021	-7.210437	1.637451
H	4.327995	-7.308862	0.780546
H	8.334098	-6.838627	2.293565
H	6.187140	-8.107826	2.239699
C	2.544682	-1.439928	0.170074
C	3.241417	-0.775012	-0.801796
H	2.683484	-2.517556	0.218406
H	3.159829	0.303673	-0.912113
N	-1.807921	-1.154555	2.422077
C	6.785826	-3.597530	-0.738344
H	7.822609	-3.527845	-1.094263
H	6.156961	-3.659597	-1.636643
C	6.441257	-2.308359	0.054036
H	7.117763	-2.276042	0.920983
H	5.438594	-2.442315	0.498891

173

(S)-alkyl-full-ligand-TS

Eopt -5155.679282

Ir	-0.031922	-0.881532	-1.033653
P	0.346164	1.283871	-0.560137
P	-1.999964	-1.201203	0.326464
N	0.077660	2.272429	-1.943717
O	-0.560144	1.951214	0.622970
O	1.863734	1.812352	-0.187545
O	-3.155992	-2.255893	-0.192385
O	-2.870074	0.212388	0.295658
C	0.971342	2.011517	-3.058444
C	-1.290062	2.347270	-2.429727
C	-0.511750	3.331368	0.876248
C	2.319922	2.068666	1.109716
C	-2.878481	-2.859397	2.392365
C	-0.901069	-1.555314	2.850060
C	-4.100294	-1.997165	-1.176466
C	-4.137589	0.337431	0.855622
C	1.831381	3.020979	-3.493729
C	0.912335	0.763031	-3.714420
C	-1.878868	3.606807	-2.569860
C	-1.970412	1.178852	-2.823298
C	0.632941	3.908965	1.399509
C	-1.698581	4.056729	0.635303
C	1.750062	3.059748	1.896166
C	3.447242	1.324717	1.527313
C	-4.164673	-2.496756	2.802461
C	-2.468755	-4.209619	2.458499
C	-0.849671	-0.290210	3.447077
C	0.030096	-2.557103	3.215874
C	-5.103670	-1.053132	-0.990290
C	-4.042443	-2.837271	-2.313658
C	-5.233400	-0.301754	0.291497
C	-4.240571	1.185694	1.982529
C	2.630809	2.817012	-4.621376
H	1.857006	3.961234	-2.950482
C	1.723360	0.581802	-4.851578
C	0.021646	-0.332072	-3.272369
C	-3.170677	3.722151	-3.085172
H	-1.313200	4.486152	-2.279622
C	-3.279896	1.314171	-3.320074
C	-1.319023	-0.145857	-2.834038
C	0.669732	5.350364	1.477647
C	-1.710589	5.416770	0.832705
H	-2.579679	3.524452	0.296009
C	2.243708	3.207490	3.248917
C	3.989892	1.537693	2.769884
H	3.884953	0.604195	0.849475
C	-5.087997	-3.470247	3.187585
H	-4.446480	-1.451487	2.800943
C	-3.427000	-5.181825	2.814051
C	-1.078398	-4.626214	2.289184
C	0.134392	0.010431	4.389523
H	-1.587981	0.454401	3.169465
C	1.027202	-2.222208	4.154588
C	0.009997	-3.912033	2.660621
C	-6.007405	-0.819767	-2.095868
C	-4.970367	-2.697009	-3.315695
H	-3.254393	-3.579993	-2.361130
C	-6.484413	-0.230481	1.013143
C	-5.454639	1.352643	2.603537
H	-3.350543	1.698761	2.330794
C	2.565415	1.598431	-5.306207
H	3.297228	3.604386	-4.963143
H	1.678533	-0.363702	-5.386630
H	0.239594	-1.292438	-3.730818
C	-3.874493	2.568808	-3.451837
H	-3.624318	4.703439	-3.197572
H	-3.820238	0.424073	-3.628277
H	-1.992367	-0.977843	-3.012318
C	1.860827	6.071362	1.777214
C	-0.522964	6.102763	1.207966

H	-2.620611	5.986640	0.661794
C	1.610043	4.040766	4.216156
C	3.392342	2.458108	3.671093
H	4.868323	0.980226	3.084684
C	-4.725215	-4.822377	3.168042
H	-6.089953	-3.172924	3.485629
H	-3.125047	-6.226462	2.837766
H	-0.923864	-5.658990	1.981198
C	1.079039	-0.960313	4.742248
H	0.164536	0.997032	4.843164
H	1.759830	-2.977871	4.428661
H	0.973416	-4.418733	2.633643
C	-6.939759	0.257820	-2.115738
C	-5.953269	-1.671651	-3.250648
H	-4.943280	-3.350567	-4.184327
C	-7.617984	-1.023451	0.671191
C	-6.594230	0.626988	2.160726
H	-5.547393	2.014529	3.461144
H	3.177974	1.434465	-6.188914
H	-4.881925	2.647657	-3.851724
C	1.864363	7.450620	1.836557
H	2.785166	5.528777	1.943916
C	-0.492520	7.522773	1.301802
C	2.108337	4.159674	5.498575
H	0.709922	4.582507	3.950193
C	3.896436	2.618588	4.992120
H	-5.445126	-5.588237	3.445023
H	1.851224	-0.732718	5.472355
C	-7.794476	0.449794	-3.183144
H	-6.969708	0.953519	-1.285051
C	-6.861935	-1.462143	-4.325956
C	-8.796679	-0.936123	1.384600
H	-7.550227	-1.723478	-0.153646
C	-7.831393	0.715342	2.859445
C	0.674862	8.187330	1.612212
H	2.790757	7.976610	2.053397
H	-1.409254	8.074572	1.105774
C	3.273989	3.457516	5.891405
H	1.595935	4.796859	6.215187
H	4.777757	2.050495	5.281449
C	-7.771733	-0.426787	-4.295598
H	-8.487695	1.287395	-3.170789
H	-6.811347	-2.130686	-5.182649
C	-8.916602	-0.044751	2.479300
H	-9.639958	-1.562987	1.105419
H	-7.895340	1.384724	3.714620
H	0.689958	9.272560	1.671880
H	3.662114	3.569035	6.900562
H	-8.457500	-0.271463	-5.124582
H	-9.854384	0.023039	3.024727
Cl	-0.513054	-3.210952	-1.850251
C	1.443732	-1.662101	0.356403
H	1.819730	-0.928761	1.064142
H	1.071737	-2.572465	0.811446
C	3.730974	-1.459938	-2.787165
H	3.196044	-0.815906	-3.507485
H	3.445288	-2.496263	-3.026709
O	5.100351	-1.304617	-2.944467
C	7.444331	-1.392153	-2.006724
C	5.983759	0.673704	-1.378317
H	7.500760	-2.469358	-2.232126
C	8.021127	-0.618245	-3.247729
C	8.341241	-1.133600	-0.770027
H	5.010745	1.129672	-1.145244
C	6.578387	1.461443	-2.601737
C	6.881093	0.879635	-0.128631
H	9.042056	-0.276208	-3.015856
H	8.129677	-1.306768	-4.097589
C	7.179597	0.587615	-3.726535
H	8.031975	-1.783750	0.062583
H	9.384446	-1.415428	-0.991880
C	8.309352	0.331469	-0.300099
H	5.793663	2.086237	-3.050789
H	7.343481	2.166852	-2.241090
H	6.417102	0.398483	0.745163
H	6.940451	1.953063	0.118122
H	6.354755	0.204853	-4.335947
H	8.862823	0.433394	0.645154
H	7.791794	1.218793	-4.388905
H	8.847026	0.953770	-1.029253
B	5.894449	-0.917790	-1.774677
C	5.433656	-4.233545	0.674735
C	4.596434	-4.287392	1.803872
C	6.648602	-4.936702	0.723011
C	4.958707	-5.017424	2.939429
H	3.648979	-3.752114	1.789166
C	7.018614	-5.668421	1.858265
H	7.310763	-4.914668	-0.140520
C	6.175094	-5.711311	2.972864
H	4.291264	-5.048119	3.798138
H	7.964325	-6.205994	1.868885
H	6.458256	-6.280322	3.855235
C	2.098253	-1.823807	-0.911359
C	3.204631	-1.087891	-1.437358
H	1.941819	-2.784218	-1.391825
H	3.394938	-0.084183	-1.086616
N	-1.942424	-1.861849	1.903490
C	5.038471	-3.427563	-0.551748
H	5.694461	-3.709720	-1.383190
H	4.022275	-3.717932	-0.848018
C	5.102821	-1.906228	-0.333554
H	6.060602	-1.651831	0.142523
H	4.391531	-1.597814	0.438976

173
(S)-alkyl-full-ligand-Pdt
Eopt -5155.801068

Ir	-0.016781	-0.828681	-1.047330
P	0.452765	1.201935	-0.262016
P	-2.146404	-1.235576	0.176654
N	0.354609	2.390886	-1.508784

O	-0.529501	1.767945	0.921531
O	1.953203	1.602487	0.311475
O	-3.313116	-2.153023	-0.561177
O	-2.961899	0.218133	0.269753
C	1.327764	2.230871	-2.576446
C	-0.957967	2.578971	-2.109225
C	-0.474541	3.107699	1.324735
C	2.299580	1.734804	1.656925
C	-3.279283	-3.089805	1.952796
C	-1.292871	-1.944202	2.712146
C	-4.128466	-1.714615	-1.593713
C	-4.271079	0.340454	0.714486
C	2.199082	3.283877	-2.864799
C	1.308055	1.056537	-3.358104
C	-1.486579	3.871758	-2.158697
C	-1.625553	1.493821	-2.709510
C	0.636099	3.594048	1.993576
C	-1.622655	3.890457	1.072860
C	1.687287	2.663564	2.488300
C	3.380018	0.933091	2.095715
C	-4.586462	-2.709117	2.271712
C	-2.933630	-4.459773	1.915450
C	-1.228130	-0.746167	3.433497
C	-0.434559	-3.021205	3.041680
C	-5.099804	-0.741975	-1.385563
C	-3.990508	-2.401726	-2.823243
C	-5.330231	-0.158390	-0.032584
C	-4.457977	1.040694	1.929981
C	3.049383	3.201649	-3.969509
H	2.185773	4.166692	-2.231590
C	2.181035	0.992367	-4.460729
C	0.371650	-0.061556	-3.093700
C	-2.708788	4.106306	-2.790757
H	-0.927968	4.684683	-1.706142
C	-2.862667	1.749746	-3.329671
C	-1.028309	0.142409	-2.782467
C	0.699626	5.018176	2.223830
C	-1.620597	5.222530	1.410616
H	-2.484973	3.422486	0.612020
C	2.066936	2.666392	3.885943
C	3.822466	1.013369	3.392236
H	3.859445	0.267812	1.391500
C	-5.582206	-3.668777	2.461630
H	-4.826711	-1.656466	2.349711
C	-3.960569	-5.413771	2.075507
C	-1.552721	-4.930014	1.826600
C	-0.301251	-0.584549	4.464436
H	-1.909873	0.058268	3.179680
C	0.503990	-2.828209	4.075548
C	-0.469050	-4.313022	2.353575
C	-5.867286	-0.311191	-2.533272
C	-4.796035	-2.074219	-3.885691
H	-3.237448	-3.178079	-2.892425
C	-6.644533	-0.115709	0.567304
C	-5.721607	1.188163	2.448950
H	-3.589875	1.458416	2.429288
C	3.030619	2.054647	-4.772434
H	3.718852	4.025590	-4.201984
H	2.174858	0.102420	-5.086129
H	0.586587	-0.944584	-3.689752
C	-3.400550	3.036385	-3.370974
H	-3.115895	5.113436	-2.830234
H	-3.392744	0.927128	-3.801629
H	-1.703192	-0.602658	-3.192856
C	1.878847	5.668918	2.686857
C	-0.450537	5.830455	1.943120
H	-2.501389	5.834341	1.231558
C	1.364901	3.415922	4.874589
C	3.163346	1.853429	4.328204
H	4.664299	0.408612	3.720282
C	-5.272871	-5.028580	2.338627
H	-6.596958	-3.352884	2.689135
H	-3.704009	-6.469195	2.017885
H	-1.417695	-5.935214	1.430420
C	0.570811	-1.630879	4.785099
H	-0.259989	0.353024	5.011739
H	1.181318	-3.642578	4.323073
H	0.470629	-4.863316	2.351127
C	-6.742647	0.812920	-2.498135
C	-5.729573	-1.006686	-3.782012
H	-4.705645	-2.609400	-4.827988
C	-7.767767	-0.794004	0.011017
C	-6.837940	0.587270	1.805237
H	-5.875368	1.736199	3.375443
H	3.682530	1.985345	-5.639450
H	-4.353135	3.205851	-3.866411
C	1.909248	7.034491	2.888049
H	2.774371	5.084034	2.867415
C	-0.395158	7.232150	2.184371
C	1.752231	3.397998	6.200050
H	0.498463	4.000778	4.589016
C	3.554833	1.873616	5.696180
H	-6.046599	-5.781831	2.463736
H	1.298135	-1.512404	5.584057
C	-7.468919	1.194117	-3.609000
H	-6.828601	1.393694	-1.586836
C	-6.506576	-0.602775	-4.904100
C	-9.009555	-0.739068	0.611782
H	-7.642432	-1.380794	-0.891824
C	-8.136007	0.647063	2.386781
C	0.758443	7.826351	2.650263
H	2.827165	7.507164	3.228578
H	-1.280982	7.828451	1.976463
C	2.869530	2.634852	6.618654
H	1.188502	3.973385	6.930311
H	4.399997	1.260138	6.000649
C	-7.366459	0.471953	-4.823376
H	-8.121162	2.062214	-3.551493
H	-6.394734	-1.154031	-5.835388
C	-9.205903	0.003739	1.802271

H	-9.843999	-1.276655	0.168172	H	6.118370	1.318842	-0.303407
H	-8.261526	1.198981	3.315825	H	6.567967	2.781782	-1.172560
H	0.793140	8.899054	2.822516	H	6.875784	-0.116744	-4.992192
H	3.170955	2.638867	7.662959	H	8.512593	1.713941	0.009144
H	-7.950941	0.775768	-5.688141	H	8.131677	1.057259	-5.367614
H	-10.191529	0.048715	2.258480	H	8.778781	1.875020	-1.716469
Cl	-0.549034	-3.012331	-2.206717	B	6.347760	-0.468959	-2.388730
C	1.205274	-2.007704	0.369646	C	5.287467	-4.493523	0.819813
H	1.503022	-1.471839	1.267302	C	4.754345	-5.023135	2.007198
H	0.634940	-2.913222	0.543903	C	6.664087	-4.649319	0.585497
C	4.011933	-1.387232	-2.238857	C	5.568598	-5.683518	2.933608
H	3.684661	-0.494458	-2.776952	H	3.688657	-4.921725	2.204510
H	3.655483	-2.264951	-2.789093	C	7.483804	-5.308487	1.508246
O	5.446824	-1.483190	-2.263663	H	7.096232	-4.253933	-0.331824
C	7.897647	-0.810464	-2.372411	C	6.938861	-5.827990	2.687878
C	6.081759	1.090314	-2.476876	H	5.132160	-6.089670	3.843523
H	8.083419	-1.892281	-2.324537	H	8.546203	-5.420237	1.302948
C	8.571332	-0.220046	-3.657707	H	7.573283	-6.343972	3.404676
C	8.466315	-0.168780	-1.069843	C	2.010592	-1.931760	-0.788923
H	5.021924	1.368842	-2.502108	C	3.437559	-1.392264	-0.804148
C	6.794328	1.652444	-3.755695	H	1.859611	-2.729566	-1.510624
C	6.691106	1.687861	-1.167613	H	3.459382	-0.365304	-0.431209
H	9.421362	0.420241	-3.379970	N	-2.263523	-2.096931	1.659889
H	8.991109	-1.035259	-4.261959	C	4.408027	-3.749976	-0.165359
C	7.591301	0.585929	-4.534255	H	4.815887	-3.868366	-1.175592
H	8.026670	-0.677874	-0.198213	H	3.406923	-4.197405	-0.164953
H	9.552578	-0.339724	-1.011053	C	4.301842	-2.246122	0.171640
C	8.177960	1.340293	-0.968766	H	5.311444	-1.821659	0.207224
H	6.044448	2.085051	-4.429444	H	3.878630	-2.139031	1.177664
H	7.465707	2.478361	-3.479537				

2.6. Characterization Data for Products

(*R*,*E*)-4-phenyl-2-vinylbut-3-en-1-ol (4)

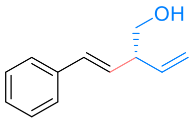

Prepared following general procedure **I**, with a reaction time of 10 min. Purification by flash column chromatography (hexane/ethyl acetate = 92:8, R_f = 0.2) gave the title compound (27.8 mg, 0.16 mmol, 80% yield, 94% ee) as a colourless oil.

NMR Spectroscopy (<u>*see spectra*</u>):

^{1}H NMR (400 MHz, $CDCl_3$): δ 7.42 – 7.36 (m, 2H), 7.31 (t, *J* = 7.6 Hz, 2H), 7.26 – 7.20 (m, 1H), 6.51 (d, *J* = 16.0 Hz, 1H), 6.14 (dd, *J* = 16.0, 7.7 Hz, 1H), 5.84 (ddd, *J* = 17.4, 10.0, 7.4 Hz, 1H), 5.22 (m, 2H), 3.66 (d, *J* = 6.8 Hz, 2H), 3.14 (p, *J* = 7.2 Hz, 1H), 1.71 (s, 1H).

^{13}C NMR (101 MHz, $CDCl_3$): δ 137.4, 137.1, 132.4, 128.7, 127.6, 126.3, 117.4, 65.3, 50.2.

HRMS (ESI) m/z calculated for $C_{12}H_{15}O$ $[M+H]^+$, 175.1117 found, 175.1117.

Specific rotation: $[\alpha]_D^{22.6}$ −47.6 (c = 0.84 M, CH_2Cl_2).

IR (neat): 3398, 3005, 2989, 2925, 2863, 1462, 1275, 1260, 749 cm^{-1}.

HPLC: Chiralpak IB column (250 mm), detected at 230 nm, hexane/i-propanol = 95/5, flow = 1.0 mL/min, retention time: 14.3 min (major), 15.4 min (minor).

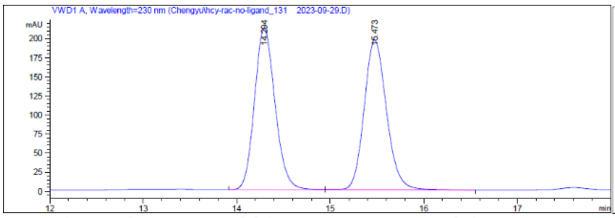

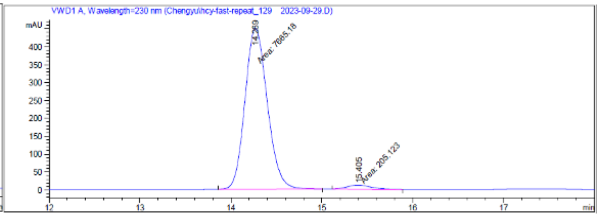

Peak #	RetTime [min]	Type	Width [min]	Area [mAU*s]	Height [mAU]	Area %
1	14.294	BV R	0.2159	3240.01416	211.83820	49.7313
2	15.473	VV R	0.2444	3275.02173	196.41022	50.2687

Peak #	RetTime [min]	Type	Width [min]	Area [mAU*s]	Height [mAU]	Area %
1	14.269	MM	0.2842	7665.17627	449.51666	97.3937
2	15.405	MM	0.3003	205.12344	11.38343	2.6063

(*R*,*E*)-4-(4-aminophenyl)-2-vinylbut-3-en-1-ol (5)

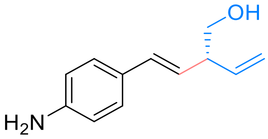

Prepared following general procedure **I**, with a reaction time of 10 min. Purification by flash column chromatography (hexane/ ethyl acetate = 14:86, R_f = 0.3) gave the title compound (25.7 mg, 0.136 mmol, 68% yield, 84% ee) as a yellow oil.

NMR Spectroscopy (<u>*see spectra*</u>):

^{1}H NMR (400 MHz, $CDCl_3$): δ 7.22 – 7.15 (m, 2H), 6.70 – 6.58 (m, 2H), 6.40 (d, *J* = 16.0 Hz, 1H), 5.91 (dd, *J* = 16.0, 7.8 Hz, 1H), 5.82 (ddd, *J* = 16.9, 10.5, 7.3 Hz, 1H) , 5.24 – 5.15 (m, 2H), 3.69 (s, 2H), 3.62 (d, *J* = 6.9 Hz, 2H), 3.14 – 3.04 (m, 1H), 1.58 (s, 1H).

^{13}C NMR (101 MHz, $CDCl_3$): δ 146.1, 137.8, 132.3, 127.9, 127.5, 124.8, 117.1, 115.2, 65.4, 50.2.

HRMS (ESI) m/z calculated for $C_{12}H_{16}NO$ $[M+H]^+$, 190.1226 found, 190.1221.

Specific rotation: Specific rotation: $[\alpha]_D^{22.5}$ −80.0 (c = 0.20 M, CH_2Cl_2).

IR (neat): 3350, 2924, 2868, 1609, 1515, 1276, 1177,1042, 917, 812, 749 cm^{-1}.

HPLC: Chiralpak AD–H column (250 mm), detected at 254 nm, hexane/i-propanol = 90/10, flow = 1.0 mL/min, retention time: 61.4 min (major), 71.2 min (minor).

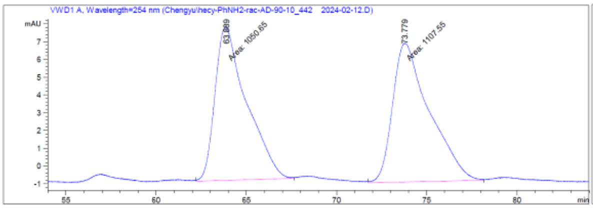

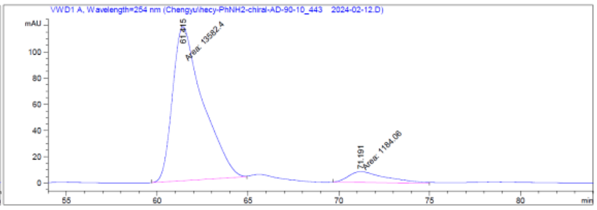

Peak #	RetTime [min]	Type	Width [min]	Area [mAU*s]	Height [mAU]	Area %
1	63.889	MM	2.0262	1050.65173	8.64219	48.6819
2	73.779	MM	2.3580	1107.54761	7.82818	51.3181

Peak #	RetTime [min]	Type	Width [min]	Area [mAU*s]	Height [mAU]	Area %
1	61.415	MM	1.9290	1.35824e4	117.35285	91.9814
2	71.191	MM	2.3637	1184.05774	8.34907	8.0186

(*R*,*E*)-4-(4-methoxyphenyl)-2-vinylbut-3-en-1-ol (6)

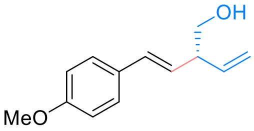

Prepared following general procedure **I** using Cy_2BH*, with a reaction time of 10 min. Purification by flash column chromatography (hexane/CH_2Cl_2 = 50:50, R_f = 0.3) gave the title compound (19.6 mg, 0.096 mmol, 48% yield, 90% ee) as a colourless oil.

*Using 9-BBN a yield of 54% and ee of 81% was obtained.

NMR Spectroscopy (*see spectra*):

^{1}H NMR (400 MHz, $CDCl_3$): δ 7.31 (d, *J* = 8.8 Hz, 2H), 6.85 (d, *J* = 8.7 Hz, 2H), 6.45 (d, *J* = 16.0 Hz, 1H), 5.98 (dd, *J* = 16.0, 7.7 Hz, 1H), 5.83 (ddd, *J* = 18.8, 9.9, 7.4 Hz, 1H), 5.29 – 5.16 (m, 2H), 3.81 (s, 3H), 3.64 (d, *J* = 6.8 Hz, 2H), 3.11 (p, *J* = 7.2 Hz, 1H), 1.59 (s, 1H).

^{13}C NMR (101 MHz, $CDCl_3$): δ 159.3, 137.7, 131.8, 130.0, 127.5, 126.4, 117.3, 114.1, 65.4, 55.4, 50.2 ppm.

HRMS (ESI) m/z calculated for $C_{13}H_{17}O_2$ $[M+H]^+$, 205.1223 found, 205.1225.

Specific rotation: $[\alpha]_D^{21.8}$ −72.7 (c = 0.55 M, CH_2Cl_2).

IR (neat): 3378, 2932, 2835, 1606, 1509, 1245, 1174, 1031, 966, 806 cm^{-1}.

HPLC: Chiralpak IB column (250 mm), detected at 254 nm, hexane/i-propanol = 95/5, flow = 1.0 mL/min, retention time: 12.0 min (minor), 13.7 min (major).

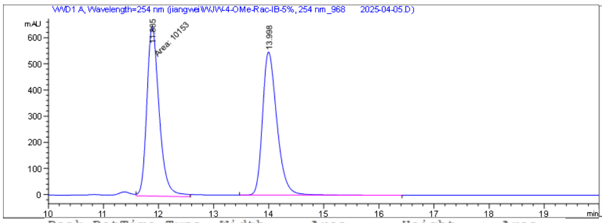

Peak #	RetTime [min]	Type	Width [min]	Area [mAU*s]	Height [mAU]	Area %
1	11.885	MM	0.2616	1.01530e4	646.74097	50.5430
2	13.998	BB	0.2789	9934.81934	546.98541	49.4570

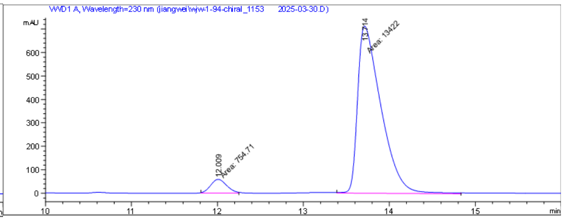

Peak #	RetTime [min]	Type	Width [min]	Area [mAU*s]	Height [mAU]	Area %
1	12.009	MM	0.2155	754.71021	58.36049	5.3236
2	13.714	MM	0.3145	1.34220e4	711.39709	94.6764

(*R*,*E*)-4-(2,3-dihydrobenzofuran-5-yl)-2-vinylbut-3-en-1-ol (7)

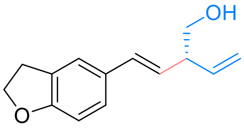

Prepared following general procedure **I** using Cy_2BH*, with a reaction time of 16 h. Purification by flash column chromatography (hexane/CH_2Cl_2 = 50:50, R_f = 0.2) gave the title compound (22.0 mg, 0.10 mmol, 51% yield, 88% ee) as a colourless oil.

*Using 9-BBN a yield of 54% and ee of 81% was obtained.

NMR Spectroscopy (*see spectra*):

^{1}H NMR (400 MHz, $CDCl_3$): δ 7.25 (s, 1H), 7.09 (dd, *J* = 8.3, 2.1 Hz, 1H), 6.71 (d, *J* = 8.3 Hz, 1H), 6.42 (d, *J* = 15.9 Hz, 1H), 5.93 (dd, *J* = 16.0, 7.8 Hz, 1H), 5.81 (ddd, *J* = 17.6, 9.9, 7.3 Hz, 1H), 5.23 – 5.15 (m, 2H), 4.56 (t, *J* = 8.7 Hz, 2H), 3.62 (d, *J* = 6.7 Hz, 2H), 3.18 (t, *J* = 8.7 Hz, 2H), 3.09 (p, *J* = 7.0 Hz, 1H).

^{13}C NMR (101 MHz, $CDCl_3$): δ 159.8, 137.7, 132.2, 130.0, 127.5, 126.7, 125.7, 122.5, 117.1, 109.3, 71.5, 65.4, 50.1, 29.7.

HRMS (EI) m/z calculated for $C_{14}H_{16}O_2$ $[M]^+$, 216.1150 found, 216.1142.

Specific rotation: $[\alpha]_D^{21.8}$ −72.7 (c = 0.55 M, CH_2Cl_2).

IR (neat): 3383, 3005, 2988, 1610, 1490, 1275, 1260, 749 cm^{-1}.

HPLC: Chiralpak IB column (250 mm), detected at 254 nm, hexane/i-propanol = 95/5, flow rate = 1.0 mL/min, 14.1 min (minor), 17.5 min (major).

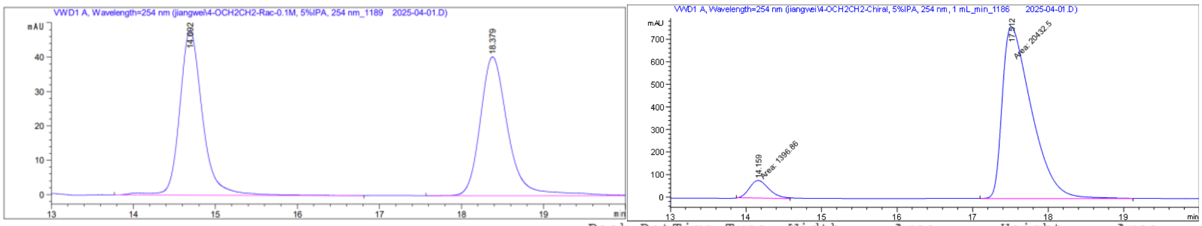

Peak #	RetTime [min]	Type	Width [min]	Area [mAU*s]	Height [mAU]	Area %
1	14.692	VB R	0.2904	927.20966	47.89572	49.1504
2	18.379	BB	0.3615	959.26514	40.38766	50.8496

Peak #	RetTime [min]	Type	Width [min]	Area [mAU*s]	Height [mAU]	Area %
1	14.159	MM	0.2991	1396.86462	77.83090	6.3990
2	17.512	MM	0.4471	2.04325e4	761.75232	93.6010

(*R*,*E*)-4-(p-tolyl)-2-vinylbut-3-en-1-ol (8)

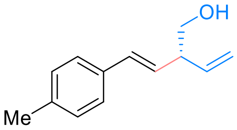

Prepared following general procedure **I** using Cy_2BH*, with a reaction time of 10 min. Purification by flash column chromatography (hexane/ ethyl acetate = 92:8, R_f = 0.2) gave the title compound (23.6 mg, 0.12 mmol, 63% yield, 94% ee) as a colourless oil.

*Using 9-BBN a yield of 70% and ee of 88% was obtained.

NMR Spectroscopy (<u>*see spectra*</u>):

^{1}H NMR (400 MHz, $CDCl_3$): δ 7.27 (d, *J* = 8.0 Hz, 2H), 7.12 (d, *J* = 7.8 Hz, 2H), 6.47 (d, *J* = 15.9 Hz, 1H), 6.07 (dd, *J* = 16.0, 7.8 Hz, 1H,), 5.83 (ddd, *J* = 17.5, 10.0, 7.3 Hz, 1H), 5.31 – 5.11 (m, 2H), 3.64 (d, *J* = 6.9 Hz, 2H), 3.12 (p, *J* = 7.2 Hz, 1H), 2.33 (s, 3H), 1.64 (s, 1H).

^{13}C NMR (101 MHz, $CDCl_3$): δ 137.6, 137.4, 134.4, 132.3, 129.4, 127.6, 126.2, 117.3, 65.4, 50.2, 21.3.

HRMS (ESI) m/z calculated for $C_{13}H_{17}O$ $[M+H]^+$, 189.1274 found, 189.1276.

Specific rotation: $[\alpha]_D^{21.5}$ −73.7 (*c* = 0.95 M, CH_2Cl_2).

IR (neat): 3389, 2921, 2870, 1722, 1607, 1513, 1413, 1273, 1178, 1040, 968, 917, 798 cm^{-1}.

HPLC: Chiralpak AD–H column (250 mm), detected at 254 nm, hexane/*i*-propanol = 90/10, flow = 1.0 mL/min, retention time: 9.25 min (major), 11.2 min (minor).

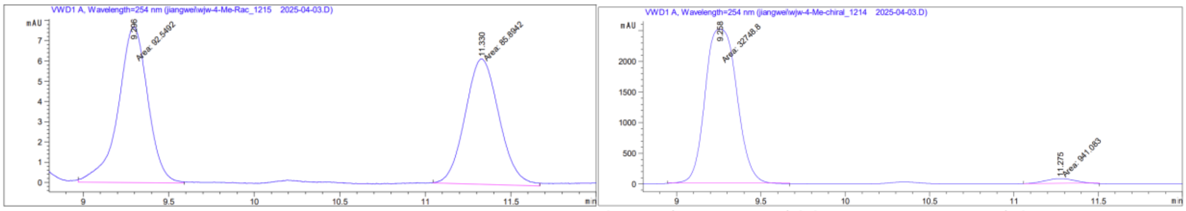

Peak #	RetTime [min]	Type	Width [min]	Area [mAU*s]	Height [mAU]	Area %
1	9.296	MM	0.2003	92.54916	7.69947	51.8647
2	11.330	MM	0.2310	85.89417	6.19616	48.1353

Peak #	RetTime [min]	Type	Width [min]	Area [mAU*s]	Height [mAU]	Area %
1	9.258	MM	0.2171	3.27488e4	2514.35889	97.2066
2	11.275	MM	0.2024	941.08325	77.48492	2.7934

(*R*,*E*)-4-([1,1'-biphenyl]-4-yl)-2-vinylbut-3-en-1-ol (9)

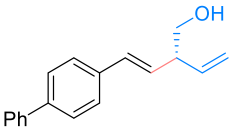

Prepared following general procedure **I**, with a reaction time of 10 min. Purification by flash column chromatography (hexane/ ethyl acetate = 92:8, R_f = 0.2) gave the title compound (44.1 mg, 0.176 mmol, 88% yield, 95% ee) as a white solid.

NMR Spectroscopy (*see spectra*):

^{1}H NMR (400 MHz, $CDCl_3$): δ 7.65 – 7.52 (m, 4H), 7.49 – 7.40 (m, 4H), 7.38 – 7.29 (m, 1H), 6.55 (d, *J* = 15.9 Hz, 1H), 6.19 (dd, *J* = 16.0, 7.7 Hz, 1H), 5.86 (ddd, *J* =17.9, 9.9, 7.3 Hz, 1H), 5.32 – 5.19 (m, 2H), 3.68 (d, *J* = 6.8 Hz, 2H), 3.22 – 3.12 (m, 1H), 1.55 (s, 1H).

^{13}C NMR (101 MHz, $CDCl_3$): δ 140.8, 140.4, 137.4, 136.2, 132.0, 128.9, 128.8, 127.4, 127.4, 127.1, 126.8, 117.5, 65.4, 50.2.

HRMS (EI) m/z calculated for $C_{18}H_{18}O$ $[M]^+$, 250.1358 found, 250.1350.

Specific rotation: $[\alpha]_D^{22.0}$ −80.0 (c = 0.20 M, CH_2Cl_2).

IR (neat): 3385, 2925, 2873, 1697, 1601, 1487, 1407, 1264, 1039, 1006, 761, 694 cm^{-1}.

HPLC: Chiralpak AD–H column (250 mm), detected at 254 nm, hexane/i-propanol = 95/5, flow = 1.0 mL/min, retention time: 29.5 min (major), 32.4 min (minor).

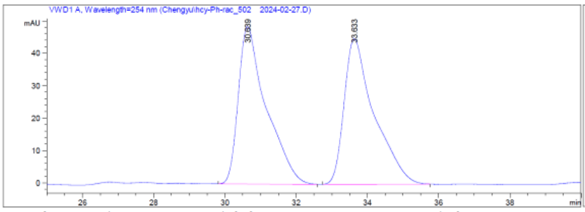

Peak #	RetTime [min]	Type	Width [min]	Area [mAU*s]	Height [mAU]	Area %
1	30.639	BV R	0.6550	2643.63452	48.54761	50.0858
2	33.633	BV R	0.6866	2634.57837	44.93457	49.9142

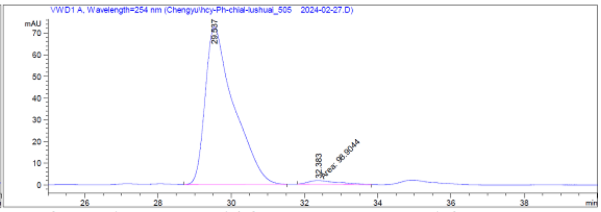

Peak #	RetTime [min]	Type	Width [min]	Area [mAU*s]	Height [mAU]	Area %
1	29.537	BV R	0.6566	3946.66455	72.53477	97.5552
2	32.383	MM	0.9504	98.90435	1.73439	2.4448

Gram-scale synthesis: To an oven-dried scintillation vial in an argon-filled glovebox, was added 4-ethynyl-1,1'-biphenyl (5.0 mmol), followed by 9-borabicyclo[3.3.1]nonane (0.5 M solution in THF, 12.0 mL, 6.0 mmol, 1.0 equiv.) and capped with a rubber septum, and stirred for 2 h at room temperature. Simultaneously, a separate oven-dried 500 mL round-bottom flask was charged with $[Ir(COD)Cl]_2$ (1.0 mol%, 0.05 mmol, 32.5 mg) and **(*S*)-L1** (4.0 mol%, 0.2 mmol, 100 mg) and capped with a rubber septum. The sealed vial and flask were removed from the glove box. The flask containing catalyst and ligand was charged with anhydrous THF (20 mL) and vinyl epoxide **3a** (13.2 mmol, 1.06 mL, 2.2 equiv.) after which the solution was stirred for 25 min. The flask containing catalyst and ligand was then diluted further with anhydrous THF (180 mL). Then, the in situ formed alkenyl borane in THF was completely transferred and added dropwise to the round-bottom flask over 25 min. After vigorous stirring for 6 h at room temperature, sodium perborate tetrahydrate (3.69 g, 24.0 mmol, 4.0 equiv.) dissolved in 300 mL of water was added, and allowed to stir for 2 h at room temperature, the resulting mixture was extracted with dichloromethane (4 × 150 mL). The combined organic layers were dried with $MgSO_4$, filtered, and concentrated in vacuo. The crude product was purified by flash column chromatography on silica gel to afford **9** (0.95 g, 3.8 mmol, 76% yield, 95% ee).

(*R*,*E*)-4-(4-chlorophenyl)-2-vinylbut-3-en-1-ol (10)

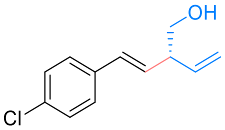

Prepared following general procedure **I**, with a reaction time of 3 h. Purification by flash column chromatography (hexane/ethyl acetate = 92:8, R_f = 0.2) gave the title compound (28.8 mg, 0.138 mmol, 69% yield, 91% ee) as a colourless oil.

NMR Spectroscopy (*see spectra*):

^{1}H NMR (400 MHz, $CDCl_3$): δ 7.38 – 7.15 (m, 4H), 6.43 (d, *J* = 16.0, Hz, 1H), 6.10 (dd, *J* = 16.0, 7.6 Hz, 1H), 5.81 (ddd, *J* = 16.5, 11.0, 7.4 Hz, 1H), 5.26 – 5.15 (m, 2H), 3.64 (d, *J* = 6.7 Hz, 2H), 3.19 – 3.05 (m, 1H), 1.59 (s, 1H).

^{13}C NMR (101 MHz, $CDCl_3$): δ 137.2, 135.6, 133.2, 131.1, 129.5, 128.8, 127.6, 117.6, 65.3, 50.1.

HRMS (EI) m/z calculated for $C_{12}H_{13}OCl$ $[M]^+$, 208.0649 found, 208.0646.

Specific rotation: $[\alpha]_D^{22.2}$ −80.0 (c = 0.75 M, CH_2Cl_2).

IR (neat): 3383, 2927, 2874, 1721, 1490, 1089, 1012, 805, 750 cm^{-1}.

HPLC: Chiralpak IB column (250 mm), detected at 254 nm, hexane/i-propanol = 95/5, flow = 1.0 mL/min, retention time: 9.4 min (minor), 10.6 min (major).

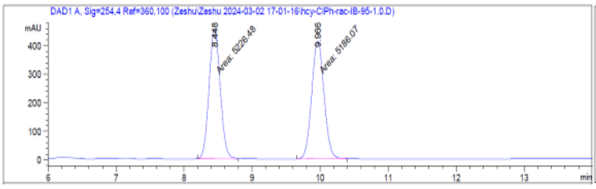

Peak #	RetTime [min]	Type	Width [min]	Area [mAU*s]	Height [mAU]	Area %
1	8.448	MM	0.1911	5226.48389	455.78250	50.1941
2	9.966	MM	0.2095	5186.06689	412.59195	49.8059

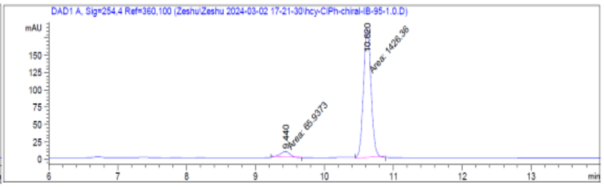

Peak #	RetTime [min]	Type	Width [min]	Area [mAU*s]	Height [mAU]	Area %
1	9.440	MM	0.1450	65.93731	7.58114	4.4185
2	10.620	MM	0.1279	1426.35913	185.91898	95.5815

Methyl (*R*,*E*)-4-(3-(hydroxymethyl)penta-1,4-dien-1-yl)benzoate (11)

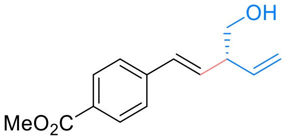

Prepared following general procedure **I**, with a reaction time of 3 h. Purification by flash column chromatography (hexane/ethyl acetate = 91:9, R_f = 0.3) gave the title compound (33.0 mg, 0.142 mmol, 71% yield, 97% ee) as a colourless oil.

NMR Spectroscopy (*see spectra*):

^{1}H NMR (400 MHz, $CDCl_3$): δ 7.96 (d, *J* = 8.6 Hz, 2H), 7.41 (d, *J* = 8.2 Hz, 2H), 6.53 (d, *J* = 15.9 Hz, 1H), 6.28 (dd, *J* = 16.0, 7.6 Hz, 1H), 5.83 (ddd, *J* =16.5, 11.0, 7.4 Hz, 1H), 5.35 – 5.13 (m, 2H), 3.90 (s, 3H), 3.68 (d, *J* = 6.7 Hz, 2H), 3.16 (p, *J* = 7.0 Hz, 1H), 1.76 (s, 1H).

^{13}C NMR (101 MHz, $CDCl_3$): δ 167.0, 141.6, 137.0, 131.7, 131.3, 130.0, 129.0, 126.2, 117.8, 65.2, 52.2, 50.2.

HRMS (EI) m/z calculated for $C_{14}H_{16}O_3$ $[M]^+$, 232.1094 found, 232.1092.

Specific rotation: $[\alpha]_D^{22.2}$ −81.9 (c = 1.05 M, CH_2Cl_2).

IR (neat): 3439, 2952, 1715, 1453, 1275, 1106, 764 cm^{-1}.

HPLC: Chiralpak IB column (250 mm), detected at 210 nm, hexane/i-propanol = 92/8, flow = 1.0 mL/min, retention time: 20.2 min (minor), 25.0 min (major).

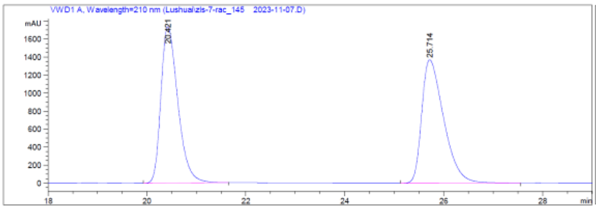

Peak #	RetTime [min]	Type	Width [min]	Area [mAU*s]	Height [mAU]	Area %
1	20.421	BV R	0.3161	4.12688e4	1737.25134	49.4258
2	25.714	BV R	0.3975	4.22277e4	1371.13721	50.5742

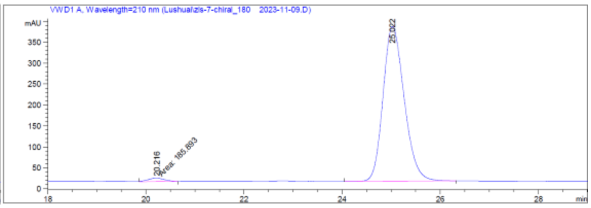

Peak #	RetTime [min]	Type	Width [min]	Area [mAU*s]	Height [mAU]	Area %
1	20.216	MM	0.3786	185.89337	8.18428	1.6525
2	25.022	VV R	0.4001	1.10631e4	369.67084	98.3475

(*R*,*E*)-4-(4-nitrophenyl)-2-vinylbut-3-en-1-ol (12)

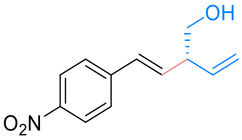

Prepared following general procedure **I**, with a reaction time of 3 h. Purification by flash column chromatography (hexane/ethyl acetate = 91:9, R_f = 0.2) gave the title compound (22.3 mg, 0.1 mmol, 51% yield, 97% ee) as a colourless oil.

NMR Spectroscopy (<u>*see spectra*</u>):

^{1}H NMR (400 MHz, $CDCl_3$): δ 8.16 (d, *J* = 8.6 Hz, 2H), 7.49 (d, *J* = 8.5 Hz, 2H), 6.56 (d, *J* = 16.0 Hz, 1H), 6.38 (dd, *J* = 16.0, 7.5 Hz, 1H), 5.84 (ddd, *J* = 17.5, 10.5, 7.5 Hz, 1H), 5.30 – 5.16 (m, 2H), 3.71 (d, *J* = 6.6 Hz, 2H), 3.19 (p, *J* = 7.1 Hz, 1H), 1.65 (s, 1H).

^{13}C NMR (101 MHz, $CDCl_3$): δ 146.9, 143.7, 136.6, 134.2, 130.2, 126.8, 124.1, 118.2, 65.2, 50.2.

HRMS (EI) m/z calculated for $C_{12}H_{12}NO_2$ $[M-H_2O]^+$, 201.0784 found, 201.0781.

Specific rotation: $[\alpha]_D^{22.4}$ −76.4 (c = 0.55 M, CH_2Cl_2).

IR (neat): 3376, 2928, 2872, 1594, 1510, 1338, 1108, 747 cm^{-1}.

HPLC: Chiralpak IB column (250 mm), detected at 210 nm, hexane/i-propanol = 95/5, flow = 1.0 mL/min, retention time: 20.0 min (major), 21.2 min (minor).

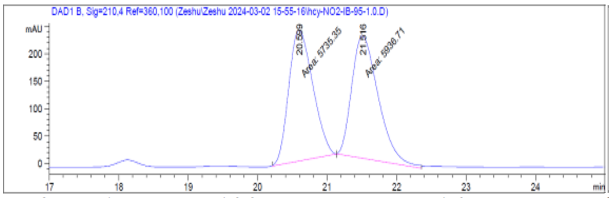

Peak #	RetTime [min]	Type	Width [min]	Area [mAU*s]	Height [mAU]	Area %
1	20.599	MM	0.4007	5735.35107	238.55669	49.1374
2	21.516	MM	0.4434	5936.70605	223.13715	50.8626

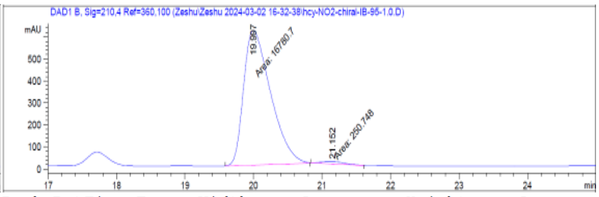

Peak #	RetTime [min]	Type	Width [min]	Area [mAU*s]	Height [mAU]	Area %
1	19.997	MM	0.4567	1.67807e4	612.32843	98.5277
2	21.152	MM	0.3831	250.74829	10.90739	1.4723

(*R*,*E*)-4-(3,5-bis(trifluoromethyl)phenyl)-2-vinylbut-3-en-1-ol (13)

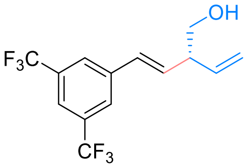

Prepared following general procedure **I**, with a reaction time of16 h. Purification by flash column chromatography (hexane/ethyl acetate = 92:8, R_f = 0.2) gave the title compound (22.3 mg, 0.072 mmol, 36% yield, 91% ee) as a colourless oil.

NMR Spectroscopy (<u>*see spectra*</u>):

^{1}H NMR (400 MHz, $CDCl_3$): δ 7.78 (s, 2H), 7.72 (s, 1H), 6.57 (dd, J = 16.1, 1.2 Hz, 1H), 6.36 (dd, J = 16.0, 7.4 Hz, 1H), 5.84 (ddd, J = 1 .2, 10.4, 7.5 Hz, 1H), 5.36 – 5.18 (m, 2H), 3.72 (d, J = 6.6 Hz, 2H), 3.19 (ttd, J = 7.6, 6.6, 1.2 Hz, 1H), 1.60 (s, 1H).

^{13}C NMR (101 MHz, $CDCl_3$): δ 139.2, 136.5, 133.3, 132.0 (q, J = 33.2 Hz), 129.5 (q, J = 3.9 Hz), 126.2 (q J = 3.9 Hz).123.4 (q, J = 272.6 Hz), 118.2. 65.2, 50.0.

HRMS (EI) m/z calculated for $C_{14}H_{12}OF_6$ $[M]^+$, 310.0787 found, 310.0778.

Specific rotation: $[\alpha]_D^{22.5}$ −47.5 (c = 0.80 M, CH_2Cl_2).

IR (neat): 3350, 3005, 2876, 1379, 1274, 1123, 894, 749, 681 cm^{-1}.

HPLC: Chiralpak IB column (250 mm), detected at 254 nm, hexane/i-propanol = 95/5, flow = 1.0 mL/min, retention time: 5.5 min (major), 6.0 min (minor).

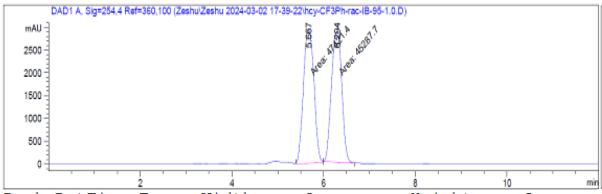

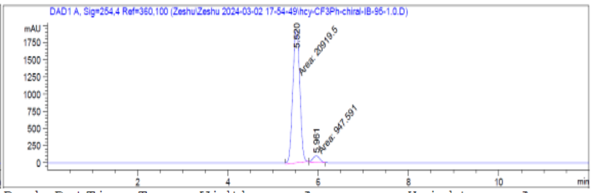

Peak #	RetTime [min]	Type	Width [min]	Area [mAU*s]	Height [mAU]	Area %
1	5.667	MM	0.2683	4.74214e4	2945.68140	51.1507
2	6.294	MM	0.2622	4.52877e4	2878.32910	48.8493

Peak #	RetTime [min]	Type	Width [min]	Area [mAU*s]	Height [mAU]	Area %
1	5.520	MM	0.1804	2.09195e4	1932.23535	95.6666
2	5.961	MM	0.1742	947.59149	90.65105	4.3334

(*R*,*E*)-4-(o-tolyl)-2-vinylbut-3-en-1-ol (14)

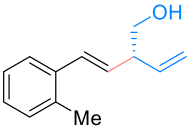

Prepared following general procedure **I**, with a reaction time of 3 h. Purification by flash column chromatography (hexane/CH_2Cl_2 = 50:50, R_f = 0.2) gave the title compound (23.7 mg, 0.126 mmol, 63% yield, 92% ee) as a colourless oil.

NMR Spectroscopy (*see spectra*):

^{1}H NMR (400 MHz, $CDCl_3$): δ 7.51 – 7.39 (m, 1H), 7.22 – 7.10 (m, 3H), 6.72 (d, J = 17.3 Hz, 1H), 6.01 (dd, J = 15.9, 7.8 Hz, 1H), 5.84 (ddd, J =17.0, 10.5,7.3 Hz, 1H), 5.36 – 5.12 (m, 2H), 3.66 (d, J = 6.8 Hz, 2H), 3.23 – 3.09 (m, 1H), 2.34 (s, 3H), 1.59 (s, 1H).

^{13}C NMR (101 MHz, $CDCl_3$): δ 137.6, 136.3, 135.4, 130.4, 130.4, 130.0, 127.6, 126.2, 125.7, 117.4, 65.4, 50.5, 20.0.

HRMS (EI) m/z calculated for $C_{13}H_{16}O$ $[M]^+$, 188.1201 found, 188.1191.

Specific rotation: $[\alpha]_D^{22.4}$ −68.6 (c = 0.35 M, CH_2Cl_2).

IR (neat): 3366, 2988, 2924, 2856, 1460, 1275, 1260, 749 cm^{-1}.

HPLC: Chiralpak IB column (250 mm), detected at 254 nm, hexane/i-propanol = 95/5, flow = 1.0 mL/min, retention time: 9.8 min (minor), 11.2 min (major).

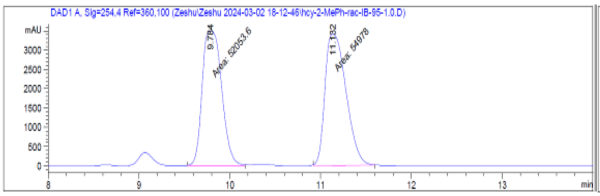

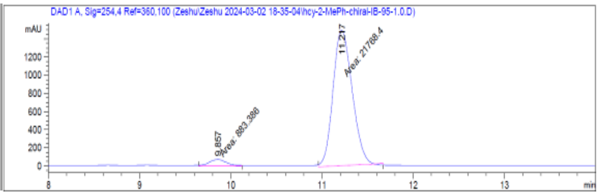

Peak #	RetTime [min]	Type	Width [min]	Area [mAU*s]	Height [mAU]	Area %
1	9.784	MM	0.2483	5.20536e4	3494.00073	48.6339
2	11.132	MM	0.2684	5.49780e4	3414.55444	51.3661

Peak #	RetTime [min]	Type	Width [min]	Area [mAU*s]	Height [mAU]	Area %
1	9.857	MM	0.2117	883.38617	69.54235	3.8999
2	11.217	MM	0.2430	2.17684e4	1493.13513	96.1001

(*R*,*E*)-4-(naphthalen-2-yl)-2-vinylbut-3-en-1-ol (15)

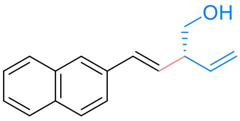

Prepared following general procedure **I**, with a reaction time of 16 h. Purification by flash column chromatography (hexane/ethyl acetate = 92:8, R_f = 0.2) gave the title compound (30.9 mg, 0.138 mmol, 69% yield, 96% ee) as a yellow solid.

NMR Spectroscopy (*see spectra*):

^{1}H NMR (400 MHz, $CDCl_3$): δ 7.82 – 7.76 (m, 3H), 7.72 (d, *J* = 1.8 Hz, 1H), 7.60 (dd, *J* = 8.6, 1.9 Hz, 1H), 7.45 (ddd, *J* = 7.0, 4.9, 1.8 Hz, 2H), 6.68 (d, *J* = 15.9 Hz, 1H), 6.27 (dd, *J* = 15.9, 7.6 Hz, 1H), 5.94 – 5.82 (m, 1H), 5.31 – 5.22 (m, 2H), 3.71 (t, *J* = 6.5 Hz, 2H), 3.20 (p, *J* = 7.3 Hz, 1H), 1.57 (d, *J* = 2.0 Hz, 1H).

^{13}C NMR (101 MHz, $CDCl_3$): δ 137.4, 134.6, 133.7, 133.0, 132.5, 129.1, 128.3, 128.0, 127.8, 126.4, 126.2, 125.9, 123.5, 117.5, 65.4, 50.3.

HRMS (EI) m/z calculated for $C_{16}H_{16}O$ $[M]^+$, 224.1201 found, 224.1192.

Specific rotation: $[\alpha]_D^{24.7}$ −8.0 (c = 0.5 M, CH_2Cl_2).

IR (neat): 3382, 2924, 2855, 1275, 1260, 749 cm^{-1}.

HPLC: Chiral column IB column (250 mm), detected at 254 nm, hexane/i-propanol = 95/5, flow rate = 1.0 mL/min, 17.7 min (major), 14.2 min (minor).

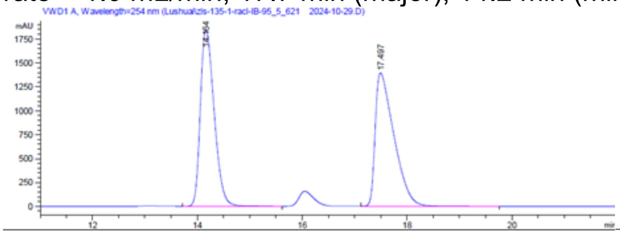

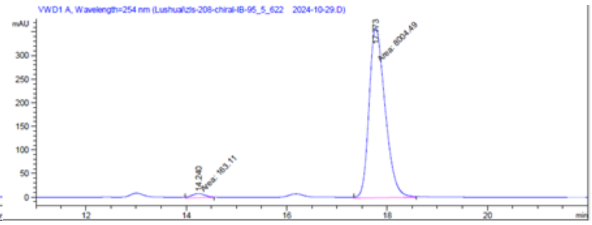

Peak #	RetTime [min]	Type	Width [min]	Area [mAU*s]	Height [mAU]	Area %
1	14.164	BB	0.2836	3.37517e4	1842.93750	49.4173
2	17.497	VB	0.3706	3.45476e4	1394.22546	50.5827

Peak #	RetTime [min]	Type	Width [min]	Area [mAU*s]	Height [mAU]	Area %
1	14.240	MM	0.3135	163.10970	8.67157	1.9970
2	17.773	MM	0.3700	8004.49414	360.52737	98.0030

(*R*,*E*)-4-(pyren-1-yl)-2-vinylbut-3-en-1-ol (16)

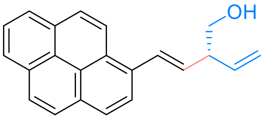

Prepared following general procedure **I**, with a reaction time of 10 min. Purification by flash column chromatography (hexane/ethyl acetate = 92:8, R_f = 0.2) gave the title compound (32.8 mg, 0.11 mmol, 55% yield, 98% ee) as a yellow solid.

NMR Spectroscopy (*see spectra*):

^{1}H NMR (400 MHz, $CDCl_3$): δ 8.35 (d, J = 9.3 Hz, 1H), 8.19 – 8.07 (m, 5H), 8.03 – 7.97 (m, 3H), 7.56 (d, *J* = 15.7 Hz, 1H), 6.37 (dd, *J* = 15.8, 7.6 Hz, 1H), 5.98 (ddd, *J* = 17.6, 10.3, 7.4 Hz, 1H), 5.39 – 5.28 (m, 2H), 3.88 – 3.73 (m, 2H), 3.37 (p, *J* = 7.2 Hz, 1H), 1.72 (s, 1H).

^{13}C NMR (101 MHz, $CDCl_3$): δ 137.5, 132.2, 131.8, 131.6, 131.0, 130.8, 129.6, 128.1, 127.6, 127.5, 127.3, 126.1, 125.4, 125.1, 125.0, 124.0, 123.2, 117.7, 65.5, 50.8.

HRMS (ESI) m/z calculated for $C_{22}H_{19}O$ $[M+H]^+$, 299.1430 found, 299.1428.

Specific rotation: $[\alpha]_D^{22.5}$ −46.0 (c = 1.00 M, CH_2Cl_2).

IR (neat): 3376, 3039, 2924, 2870, 1275, 1042, 838, 751, 713 cm^{-1}.

HPLC: Chiralpak OD–H column (250 mm), detected at 254 nm, hexane/i-propanol = 90/10, flow = 1.0 mL/min, retention time: 24.0 min (major), 30.3 min (minor)

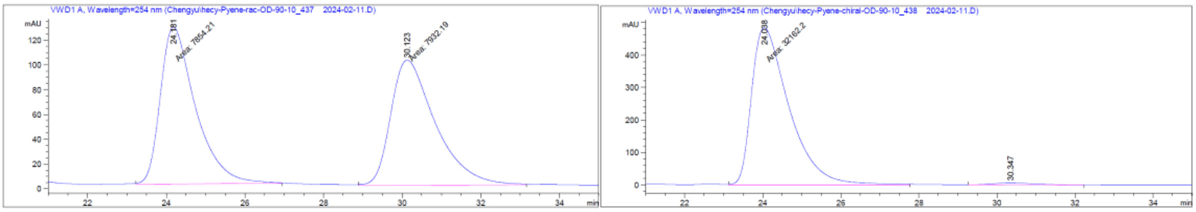

Peak #	RetTime [min]	Type	Width [min]	Area [mAU*s]	Height [mAU]	Area %
1	24.181	MM	1.0330	7854.20654	126.72749	49.7530
2	30.123	MM	1.3093	7932.19482	100.97242	50.2470

Peak #	RetTime [min]	Type	Width [min]	Area [mAU*s]	Height [mAU]	Area %
1	24.038	MM	1.1104	3.21622e4	482.75110	98.7763
2	30.347	VV R	0.8372	398.45206	5.56707	1.2237

(*R*,*E*)-4-(thiophen-3-yl)-2-vinylbut-3-en-1-ol (17)

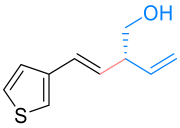

Prepared following general procedure **I**, with a reaction time of 3 h. Purification by flash column chromatography (hexane/ethyl acetate = 92:8, R_f = 0.3) gave the title compound (22.3 mg, 0.124 mmol, 62% yield, 97% ee) as a yellow oil.

NMR Spectroscopy (<u>*see spectra*</u>):

^{1}H NMR (400 MHz, $CDCl_3$): δ 7.28 – 7.24 (m, 1H), 7.20 (dd, J = 5.0, 1.3 Hz, 1H), 7.12 (dd, J = 3.0, 1.3 Hz, 1H), 6.51 (d, J = 16.0 Hz, 1H), 5.97 (dd, J = 15.9, 7.7 Hz, 1H), 5.87 – 5.75 (m, 1H), 5.24-5.16 (m, 2H), 3.63 (d, J = 6.8 Hz, 2H), 3.14 – 3.04 (m, 1H), 1.59 (s, 1H).

^{13}C NMR (101 MHz, $CDCl_3$): δ 139.8, 137.4, 128.5, 126.6, 126.2, 125.0, 121.9, 117.4, 65.3, 50.1.

HRMS (EI) m/z calculated for $C_{10}H_{12}OS$ $[M]^+$, 180.0603 found, 180.0600.

Specific rotation: $[\alpha]_D^{22.6}$ −66.7 (c = 0.60 M, CH_2Cl_2).

IR (neat): 3383, 2989, 2923, 2863, 1275, 1260, 749 cm^{-1}.

HPLC: Chiralpak IB column (250 mm), detected at 254 nm, hexane/i-propanol = 98/2, flow = 1.0 mL/min, retention time: 23.5 min (major), 24.8 min (minor).

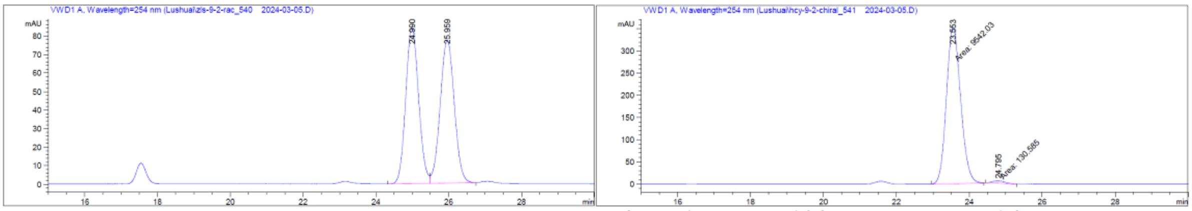

Peak #	RetTime [min]	Type	Width [min]	Area [mAU*s]	Height [mAU]	Area %
1	24.990	BV	0.3811	2074.51880	84.49454	49.9901
2	25.959	VV R	0.4049	2075.34448	77.14361	50.0099

Peak #	RetTime [min]	Type	Width [min]	Area [mAU*s]	Height [mAU]	Area %
1	23.553	MM	0.4511	9542.02637	352.57053	98.6500
2	24.795	MM	0.3978	130.58490	5.47178	1.3500

(*R*,*E*)-5-(phenylthio)-2-vinylpent-3-en-1-ol (18)

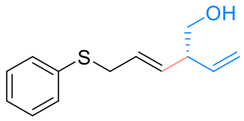

Prepared following general procedure **I**, with a reaction time of 18 h. Purification by flash column chromatography (hexane/ethyl acetate = 91:9, R_f = 0.2) gave the title compound (25.1 mg, 0.114 mmol, 57% yield, 90% ee) as a yellow oil.

NMR Spectroscopy (<u>see spectra</u>):

^{1}H NMR (400 MHz, $CDCl_3$): δ 7.36 – 7.27 (m, 2H), 7.31 – 7.20 (m, 2H), 7.23 – 7.13 (m, 1H), 5.69 – 5.51 (m, 2H), 5.32 (dd, *J* = 15.3, 7.8 Hz, 1H), 5.11 – 4.94 (m, 2H), 3.50 (d, *J* = 6.9 Hz, 2H), 3.46 – 3.26 (m, 2H), 2.90 – 2.80 (m, 1H).

^{13}C NMR (101 MHz, $CDCl_3$): δ 137.1, 135.3, 132.3, 130.9, 128.9, 128.1, 126.7, 117.0, 65.0, 49.4, 36.8.

HRMS (EI) m/z calculated for $C_{13}H_{16}OS$ $[M]^+$, 220.0916 found, 220.0914.

Specific rotation: $[\alpha]_D^{25}$ −20.0 (c = 1.0 M, CH_2Cl_2).

IR (neat): 3384, 2989, 2923, 2863, 1275, 1260, 749 cm^{-1}.

HPLC: Chiralpak IB column (250 mm), detected at 230 nm, hexane/i-propanol = 99/1, flow = 1.0 mL/min, retention time: 18.8 min (major), 17.8 min (minor).

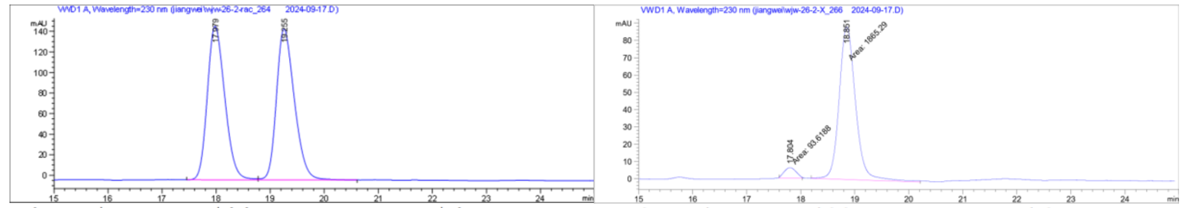

Peak #	RetTime [min]	Type	Width [min]	Area [mAU*s]	Height [mAU]	Area %
1	17.979	BV	0.3476	3353.01270	149.49040	49.7080
2	19.255	VB	0.3536	3392.40698	147.06093	50.2920

Peak #	RetTime [min]	Type	Width [min]	Area [mAU*s]	Height [mAU]	Area %
1	17.803	MM	0.2561	92.53638	6.02173	4.8205
2	18.851	MM	0.3435	1827.12292	88.64051	95.1795

(*R*, 3E,5E)-6-phenyl-2-vinylhexa-3,5-dien-1-ol (19)

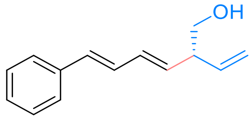

Prepared following general procedure **I**, with a reaction time of 18 h. Purification by flash column chromatography (hexane/ethyl acetate = 91:9, R_f = 0.2) gave the title compound (25.1 mg, 0.12 mmol, 60% yield, 89% ee) as a yellow oil.

NMR Spectroscopy (<u>see spectra</u>):

^{1}H NMR (400 MHz, $CDCl_3$): δ 7.40 – 7.35 (m, 2H), 7.30 (t, *J* = 7.6 Hz, 2H), 7.21 (t, *J* = 7.3 Hz, 1H), 6.76 (dd, *J* = 15.6, 10.4 Hz, 1H), 6.51 (d, *J* = 15.7 Hz, 1H), 6.31 (dd, *J* = 15.4, 10.4 Hz, 1H), 5.84 – 5.66 (m, 2H), 5.24 – 5.11 (m, 2H), 3.60 (t, *J* = 6.4 Hz, 2H), 3.06 (p, *J* = 7.7 Hz, 1H), 1.53 (t, *J* = 6.3 Hz, 1H).

^{13}C NMR (101 MHz, $CDCl_3$): δ 137.3, 132.9, 132.8, 132.0, 128.7, 127.6, 126.4, 117.4, 65.3, 50.0.

HRMS (EI) m/z calculated for $C_{14}H_{16}O$ $[M]^+$, 200.1201 found, 200.1193.

Specific rotation: $[\alpha]_D^{24.2}$ −38.0 (c = 1.0 M, CH_2Cl_2).

IR (neat): 3414, 2989, 1715, 1275, 1260, 749 cm^{-1}.

HPLC: Chiralpak IB column (250 mm), detected at 254 nm, hexane/i-propanol = 95/5, flow rate = 1.0 mL/min, 12.7 min (major), 11.4 min (minor).

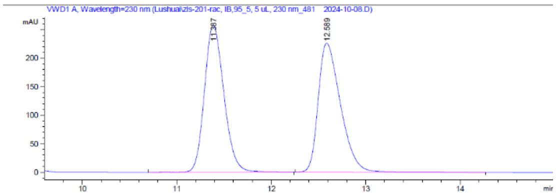

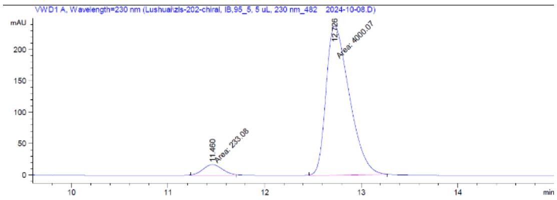

Peak #	RetTime [min]	Type	Width [min]	Area [mAU*s]	Height [mAU]	Area %
1	11.387	BB	0.2223	3681.42065	257.27084	49.9481
2	12.589	BB	0.2516	3689.06982	225.97319	50.0519

Peak #	RetTime [min]	Type	Width [min]	Area [mAU*s]	Height [mAU]	Area %
1	11.460	MM	0.2299	233.08020	16.89762	5.5061
2	12.726	MM	0.2798	4000.06519	238.23503	94.4939

(*R*,*E*)-6-phenyl-2-vinylhex-3-en-1-ol (20)

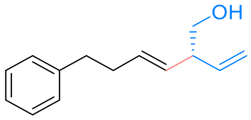

Prepared following general procedure **I**, with a reaction time of 18 h. Purification by flash column chromatography (hexane/ethyl acetate = 92:8, R_f = 0.2) gave the title compound (23.8 mg, 0.118 mmol, 59% yield, 87% ee)* as a yellow oil.

*Using BCy_2 a yield of 50% and ee of 88% was obtained.

NMR Spectroscopy (<u>*see spectra*</u>):

^{1}H NMR (400 MHz, $CDCl_3$): δ 7.31 – 7.24 (m, 2H), 7.21 – 7.13 (m, 3H), 5.70 (ddd, *J* = 17.5, 10.5, 7.3 Hz, 1H), 5.58 (dd, *J* = 15.5, 6.7 Hz, 1H), 5.28 (dt, *J* = 15.4, 7.9 Hz, 1H), 5.13 – 5.03 (m, 2H), 3.51 – 3.36 (m, 2H), 2.88 (m, 1H), 2.68 (t, *J* = 7.6 Hz, 2H), 2.48 – 2.29 (dt, *J* = 7.9, 7.6 Hz, 2H).

^{13}C NMR (101 MHz, $CDCl_3$): δ 141.8, 137.8, 132.8, 129.6, 128.6, 128.5, 126.0, 116.7, 65.2, 49.9, 35.8, 34.6.

HRMS (EI) m/z calculated for $C_{14}H_{16}$ $[M-H_2O]^+$, 184.1247 found, 184.1243.

Specific rotation: $[\alpha]_D^{22.6}$ −10.0 (c = 0.40 M, CH_2Cl_2).

IR (neat): 3380, 3026, 2924, 2856, 1725, 1459, 1453, 1275, 916, 748, 697 cm^{-1}.

HPLC: Chiralpak AD–H column (250 mm), detected at 230 nm, hexane/i-propanol = 95/5, flow = 1.0 mL/min, retention time: 10.5 min (major), 11.6 min (minor).

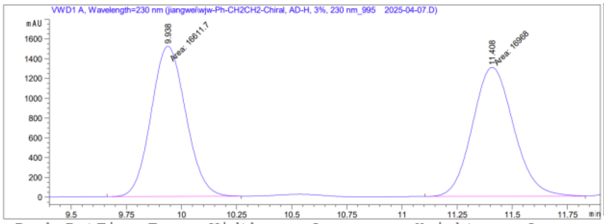

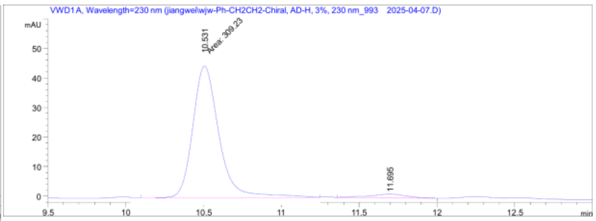

Peak #	RetTime [min]	Type	Width [min]	Area [mAU*s]	Height [mAU]	Area %
1	9.938	MM	0.1824	1.66117e4	1518.16980	49.4695
2	11.408	MM	0.2167	1.69680e4	1304.83606	50.5305

Peak #	RetTime [min]	Type	Width [min]	Area [mAU*s]	Height [mAU]	Area %
1	10.531	MM	0.2809	309.22977	18.34824	93.9758
2	11.695	BB	0.2359	19.82265	1.24082	6.0242

(*R*,*E*)-4-cyclohexyl-2-vinylbut-3-en-1-ol (21)

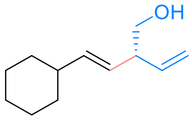

Prepared following general procedure **I**, with a reaction time of 18 h. Purification by flash column chromatography (hexane/ethyl acetate = 92:9, R_f = 0.5) gave the title compound (23.8 mg, 0.122 mmol, 61% yield, 95% ee) as a yellow oil.

NMR Spectroscopy (<u>*see spectra*</u>):

^{1}H NMR (400 MHz, $CDCl_3$): δ 5.73 (ddd, *J* = 17.6, 9.9, 7.3 Hz, 1H), 5.52 (dd, *J* = 15.7, 6.6 Hz, 1H), 5.26 (dd, *J* = 15.7, 7.6, 1.5 Hz, 1H), 5.16 – 5.09 (m, 2H), 3.57 – 3.43 (m, 2H), 2.88 (p, *J* = 7.2 Hz, 1H), 2.02 – 1.88 (m, 1H), 1.77 – 1.59 (m, 6H), 1.51 (t, *J* = 6.3 Hz, 1H), 1.33 – 0.99 (m, 5H).

^{13}C NMR (101 MHz, $CDCl_3$): δ 139.9, 138.2, 125.8, 116.6, 65.3, 49.8, 40.9, 33.2, 33.1, 26.2, 26.1.

HRMS (EI) m/z calculated for $C_{12}H_{18}$ $[M-H_2O]^+$, 162.1408 found 162.1400.

Specific rotation: $[\alpha]_D^{24.7}$ −8.0 (c = 0.50 M, CH_2Cl_2).

IR (neat): 3413, 2922, 2850, 1448, 1275, 1260, 749 cm^{-1}.

HPLC: Chiralpak IA column (250 mm), detected at 210 nm, hexane/i-propanol = 97/3, flow rate = 1.0 mL/min, 7.1 min (major), 7.7 min (minor).

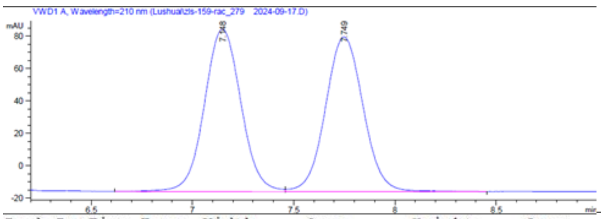

Peak #	RetTime [min]	Type	Width [min]	Area [mAU*s]	Height [mAU]	Area %
1	7.148	BV	0.1960	1257.70996	100.12997	50.3718
2	7.749	VB	0.2038	1239.14270	95.48495	49.6282

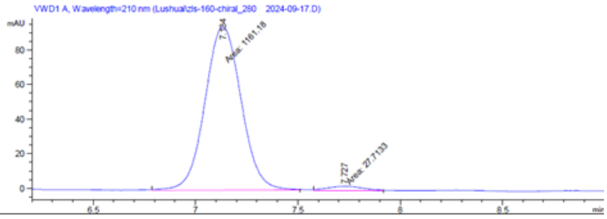

Peak #	RetTime [min]	Type	Width [min]	Area [mAU*s]	Height [mAU]	Area %
1	7.134	MM	0.2051	1161.17810	94.37239	97.6690
2	7.727	MM	0.1966	27.71329	2.34911	2.3310

(*R*,*E*)-6-(hydroxymethyl)octa-4,7-dien-1-yl 2,4,6-triisopropylbenzoate (22)

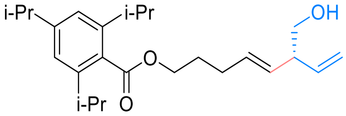

Prepared following general procedure **I**, with a reaction time of 18 h. Purification by flash column chromatography (hexane/ethyl acetate = 91:9, R_f = 0.2) gave the title compound (32.4 mg, 0.084 mmol, 42% yield, 92% ee) as a yellow oil.

NMR Spectroscopy (<u>*see spectra*</u>):

^{1}H NMR (400 MHz, $CDCl_3$): δ 7.00 (s, 2H), 5.73 (ddd, *J* = 16.8, 10.8, 7.4 Hz, 1H), 5.63 – 5.50 (m, 1H), 5.38 (dd, *J* = 15.4, 7.5 Hz, 1H), 5.20 – 5.08 (m, 2H), 4.43 – 4.19 (m, 2H), 3.53 (t, *J* = 6.3 Hz, 2H), 2.98 – 2.75 (m, 4H), 2.18 (m, 2H), 1.82 (dq, *J* = 8.2, 6.5 Hz, 2H), 1.24 (d, *J* = 6.9 Hz, 18H).

^{13}C NMR (101 MHz, $CDCl_3$): δ 171.1, 150.2, 144.7, 137.8, 131.9, 130.6, 129.9, 120.9, 116.9, 65.2, 64.2, 49.8, 34.5, 31.6, 29.1, 28.3, 24.2, 24.0.

HRMS (ESI) m/z calculated for $C_{25}H_{38}O_3Na$ $[M+Na]^+$, 409.2713 found, 409.2710.

Specific rotation: $[\alpha]_D^{22.5}$ −20.0 (c = 1.0 M, CH_2Cl_2).

IR (neat): 3447, 2989, 2868, 1724, 1462, 1275, 1260, 749 cm^{-1}.

HPLC: Chiralpak IB column (250 mm), detected at 230 nm, hexane/i-propanol = 95/5, flow = 1.0 mL/min, retention time: 6.1 min (major), 8.0 min (minor).

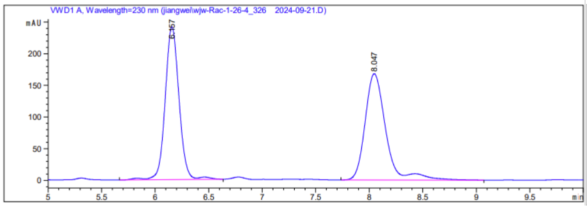

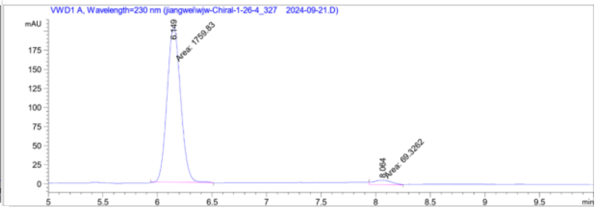

Peak #	RetTime [min]	Type	Width [min]	Area [mAU*s]	Height [mAU]	Area %
1	6.157	VV R	0.1367	2176.22144	241.19261	49.1158
2	8.047	BV R	0.1951	2254.57568	167.59633	50.8842

Peak #	RetTime [min]	Type	Width [min]	Area [mAU*s]	Height [mAU]	Area %
1	6.149	MM	0.1444	1759.82507	203.14188	96.2099
2	8.064	MM	0.1943	69.32618	5.94610	3.7901

(*R*,*E*)-6-bromo-2-vinylhex-3-en-1-ol (23)

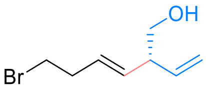

Prepared following general procedure **I**, with a reaction time of 18 h. Purification by flash column chromatography (hexane/ethyl acetate = 91:9, R_f = 0.5) gave the title compound (32.4 mg, 0.14 mmol, 70% yield, 94% ee) as a yellow oil.

NMR Spectroscopy (*see spectra*):

^{1}H NMR (400 MHz, $CDCl_3$): δ 5.80 – 5.69 (m, 1H), 5.60 – 5.43 (m, 2H), 5.20 – 5.12 (m, 2H), 3.61 – 3.49 (m, 2H), 3.48 – 3.36 (m, 2H), 2.96 (p, *J* = 7.0 Hz, 1H), 2.61 (p, *J* = 6.6 Hz, 2H), 1.57 (d, *J* = 2.4 Hz, 1H).

^{13}C NMR (101 MHz, $CDCl_3$): δ 137.4, 132.3, 129.8, 117.1, 65.1, 49.8, 35.9, 32.8.

HRMS (ESI) m/z calculated for $C_8H_{14}O_{Br}$ [M+H]$^+$, 205.0223 found, 205.0223.

Specific rotation: $[\alpha]_D^{23.3}$ −6.7 (c = 0.3 M, CH_2Cl_2).

IR (neat): 3367, 2988, 2924, 2870, 1275, 1260, 750 cm^{-1}.

HPLC: Chiralpak IA column (250 mm), detected at 210 nm, hexane/ i-propanol = 96/4, flow rate = 1.0 mL/min, 10.4 min (major), 11.3 min (minor).

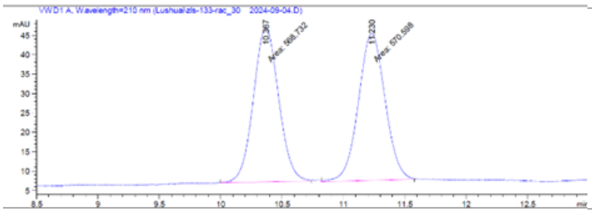

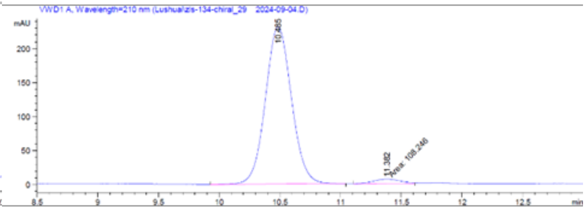

Peak #	RetTime [min]	Type	Width [min]	Area [mAU*s]	Height [mAU]	Area %
1	10.367	MM	0.2386	568.73218	39.73508	49.9181
2	11.230	MM	0.2518	570.59814	37.76176	50.0819

Peak #	RetTime [min]	Type	Width [min]	Area [mAU*s]	Height [mAU]	Area %
1	10.485	BB	0.2329	3463.45654	231.40767	96.9693
2	11.382	MM	0.2620	108.24613	6.88613	3.0307

(8*R*,9S,13S,14S,17R)-17-((*R*,E)-3-(hydroxymethyl)penta-1,4-dien-1-yl)-3-methoxy-13-methyl-7,8,9,11,12,13,14,15,16,17-decahydro-6H-cyclopenta[a]phenanthren-17-ol (24)

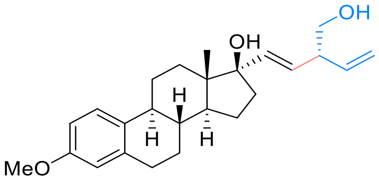

Prepared following general procedure **I**, 2.0 equiv. of 9H-BBN was used with a reaction time of 18 h. Purification by flash column chromatography (hexane/ethyl acetate = 86:14, R_f = 0.2) gave the title compound (52.1 mg, 0.136 mmol, 68% yield, >20:1 d.r.) as a yellow oil. The d.r. was determined by ^{1}H-NMR analysis.

NMR Spectroscopy (*see spectra*):

^{1}H NMR (400 MHz, $CDCl_3$): δ 7.18 (d, *J* = 8.6 Hz, 1H), 6.70 (dd, *J* = 8.7, 2.8 Hz, 1H), 6.63 (d, *J* = 2.8 Hz, 1H), 5.93 – 5.68 (m, 2H), 5.56 (dd, *J* = 15.7, 7.5 Hz, 1H), 5.27 – 5.10 (m, 2H), 3.77 (s, 3H), 3.59 (d, *J* = 7.0 Hz, 2H), 3.03 (quin, *J* = 7.1 Hz, 1H), 2.96 – 2.77 (m, 2H), 2.31 – 2.26 (m, 1H), 2.14 – 2.08 (m, 1H), 2.04 – 1.96 (m, 1H), 1.92 – 1.85 (m, 2H), 1.77 – 1.68 (m, 3H), 1.64 – 1.55 (m, 1H), 1.54 – 1.29 (m, 6H), 0.94 (s, 3H).

^{13}C NMR (101 MHz, $CDCl_3$): δ 157.5, 138.1, 137.8, 132.7, 126.7, 126.4, 117.1, 113.9, 111.6, 83.9, 65.5, 55.3, 49.6, 49.4, 47.0, 43.9, 39.6, 37.2, 32.6, 30.0, 27.6, 26.4, 23.4, 14.2.

HRMS (ESI) m/z calculated for $C_{25}H_{35}O_3$ $[M+H]^+$, 383.2581 found, 383.2588.

Specific rotation: $[\alpha]_D^{22.7}$ +15.0 (c = 0.80 M, CH_2Cl_2).

IR (neat): 3379, 2928, 2869, 1608, 1498, 1275, 1260, 749 cm^{-1}.

(*R*,*E*)-4-(trimethylsilyl)-2-vinylbut-3-en-1-ol (25)

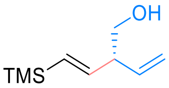

Prepared following a modified general procedure **I**, 3.0 equiv. of alkynes was used, first step 4 days, second step with a reaction time of 12 h. Purification by flash column chromatography (hexane/ethyl acetate = 95:5, R_f = 0.6) gave the title compound (13.9 mg, 0.082 mmol, 41% yield, 90% ee) as a white oil.

NMR Spectroscopy (*see spectra*):

^{1}H NMR (400 MHz, $CDCl_3$): δ 5.92 (dd, *J* = 18.7, 6.5 Hz, 1H), 5.83 – 5.68 (m, 2H), 5.34 – 5.00 (m, 2H), 3.70 – 3.47 (m, 2H), 3.08 – 2.90 (m, 1H), 0.06 (s, 9H).

^{13}C NMR (101 MHz, $CDCl_3$): δ 144.5, 137.4, 133.4, 117.3, 64.7, 53.3, -1.1.

HRMS (ESI) m/z calculated for $C_9H_{19}OSi$ $[M+H]^+$, 171.1200 found, 171.1196.

Specific rotation: $[\alpha]_D^{25.0}$ −1.3 (c = 1.5 M, CH_2Cl_2).

IR (neat): 3749, 2989, 2862, 2318, 1462, 1275, 1260, 749 cm^{-1}.

HPLC: Chiralpak AD–H column (250 mm), detected at 210 nm, hexane/i-propanol = 99.5/0.5, flow = 1.0 mL/min, retention time: 9.2 min (major), 9.4 min (minor).

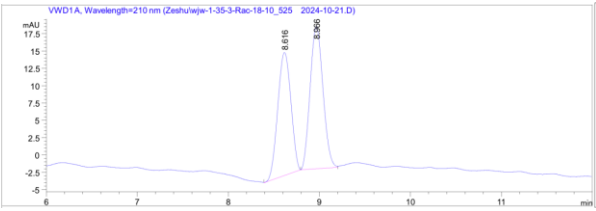

Peak #	RetTime [min]	Type	Width [min]	Area [mAU*s]	Height [mAU]	Area %
1	8.616	BV	0.1686	201.69856	18.83381	43.8913
2	8.966	VV	0.1733	257.84204	22.69747	56.1087

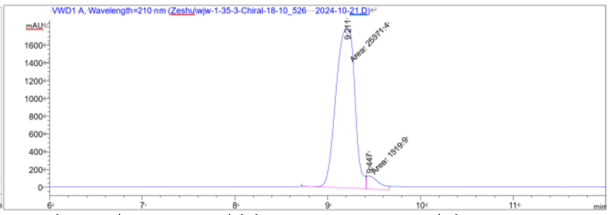

Peak #	RetTime [min]	Type	Width [min]	Area [mAU*s]	Height [mAU]	Area %
1	9.211	MM	0.2341	2.53714e4	1806.61438	95.0549
2	9.447	MM	0.1504	1319.89636	146.31082	4.9451

(*R*,*E*)-4-(dimethyl(phenyl)silyl)-2-vinylbut-3-en-1-ol (26)

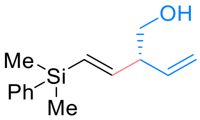

Prepared following a modified general procedure **I**, 3.0 equiv. of alkynes was used, first step 4 days, second step with a reaction time of 12 h. Purification by flash column chromatography

(hexane/ethyl acetate = 91:9, R_f = 0.6) gave the title compound (20.0 mg, 0.086 mmol, 43% yield, 94% ee) as a white oil.

NMR Spectroscopy (<u>*see spectra*</u>):

^{1}H NMR (400 MHz, $CDCl_3$): δ 7.55 – 7.47 (m, 2H), 7.35 (dd, *J* = 4.4, 2.2 Hz, 3H), 6.06 – 5.86 (m, 2H), 5.76 (ddd, *J* = 17.2, 10.4, 7.7 Hz, 1H), 5.30 – 5.02 (m, 2H), 3.58 (td, *J* = 10.0, 6.9 Hz, 2H), 3.13 – 2.91 (m, 1H), 0.34 (s, 6H).

^{13}C NMR (101 MHz, $CDCl_3$): δ 146.4, 138.6, 137.2, 133.8, 133.1, 131.0, 129.1, 127.9, 117.5, 64.8, 53.3, -2.4.

HRMS (ESI) m/z calculated for $C_{14}H_{21}OSi$ $[M+H]^+$, 233.1356 found, 233.1354.

Specific rotation: $[\alpha]_D^{25}$ −1.6 (c = 2.5 M, CH_2Cl_2).

IR (neat): 3351, 2954, 2890, 1600, 1492, 1260, 830, 750, 697 cm^{-1}.

HPLC: Chiralpak AD–H column (250 mm), detected at 230 nm, hexane/i-propanol = 99/1, flow = 1.0 mL/min, retention time: 14.2 min (major), 15.3 min (minor).

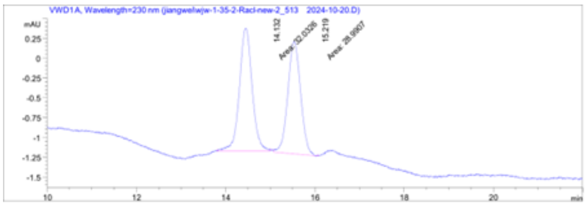

Peak #	RetTime [min]	Type	Width [min]	Area [mAU*s]	Height [mAU]	Area %
1	14.132	MM	0.3403	32.03259	1.56878	52.4924
2	15.219	MM	0.3391	28.99066	1.42477	47.5076

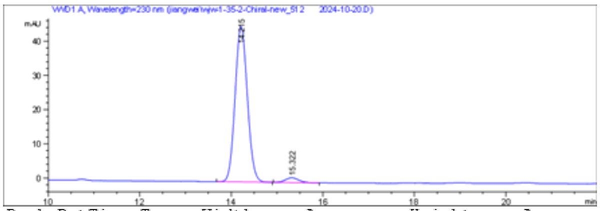

Peak #	RetTime [min]	Type	Width [min]	Area [mAU*s]	Height [mAU]	Area %
1	14.215	BB	0.2908	849.33972	45.45535	96.7317
2	15.322	BB	0.3259	28.69670	1.38673	3.2683

(*R*,*E*)-4-(benzyldimethylsilyl)-2-vinylbut-3-en-1-ol (27)

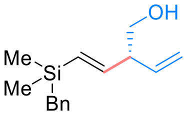

Prepared following a modified general procedure **I**, 3.0 equiv. of alkynes was used, with a reaction time of 12 h. Purification by flash column chromatography (hexane/ethyl acetate = 91:9, R_f = 0.6) gave the title compound (20.0 mg, 0.082 mmol, 41% yield, 90% ee, *Z*/*E* = 1:4) as a white oil.

NMR Spectroscopy (<u>*see spectra*</u>):

^{1}H NMR (400 MHz, $CDCl_3$): δ 7.26 – 7.15 (m, 2H), 7.13 – 7.02 (m, 2H), 6.97 (d, *J* = 6.7 Hz, 2H), 5.84 (dd, *J* = 18.8, 6.5 Hz, 1H), 5.78 – 5.65 (m, 2H), 5.20 – 5.05 (m, 2H), 3.51 (d, *J* = 6.9 Hz, 2H), 3.01 – 2.90 (m, 1H), 2.17 (s, 0.5H), 2.12 (s, 2H), 0.13 (s, 1.5H), 0.06 (s, 6H).

^{13}C NMR (101 MHz, $CDCl_3$): δ 146.0, 140.0, 137.1, 131.1, 128.5, 128.3, 128.2, 128.2, 124.4, 124.1, 117.3, 64.7, 53.4, 28.1, 26.1, -0.5, -3.1, -3.3.

Specific rotation: $[\alpha]_D^{19.3}$ −16.7 (c = 3.0 M, CH_2Cl_2).

IR (neat): 3350, 2955, 2890, 1600, 1492, 1259, 829, 750, 697 cm^{-1}.

HRMS (EI) m/z calculated for $C_{14}H_{19}SiO$ $[M-Me]^+$, 231.1200 found, 231.1197.

HPLC: Chiralpak IB column (250 mm), detected at 230 nm, hexane/i-propanol = 95/5, flow = 1.0 mL/min, retention time: 5.9 min (major), 6.3 min (minor).

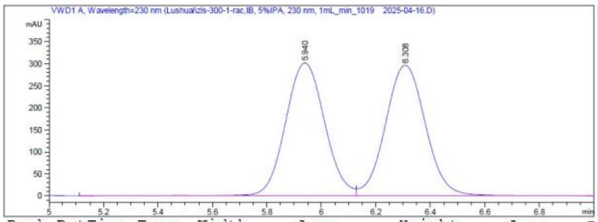 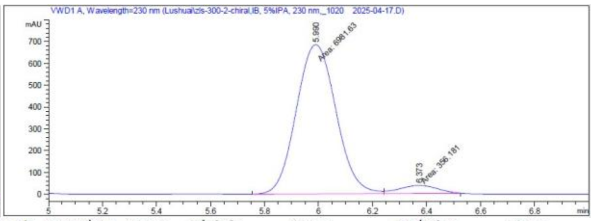

Peak #	RetTime [min]	Type	Width [min]	Area [mAU*s]	Height [mAU]	Area %
1	5.940	VV R	0.1572	3014.89966	301.29437	49.8014
2	6.308	VB	0.1605	3038.94482	296.09378	50.1986

Peak #	RetTime [min]	Type	Width [min]	Area [mAU*s]	Height [mAU]	Area %
1	5.990	MF	0.1699	6981.62842	684.71924	95.1459
2	6.373	FM	0.1627	356.18103	36.48477	4.8541

(*R*, Z)-3,4-bis(trimethylsilyl)-2-vinylbut-3-en-1-ol (28)

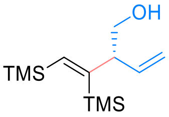

Prepared following a modified general procedure **I**, 3.0 equiv. of alkynes was used, first step 4 days, second step with a reaction time of 12 h. Purification by flash column chromatography (hexane/ethyl acetate = 95:5, R_f = 0.6) gave the title compound (19.4 mg, 0.08 mmol, 40% yield, 96% ee) as a white oil.

NMR Spectroscopy (*see spectra*):

^{1}H NMR (400 MHz, $CDCl_3$): δ 6.35 (d, *J* = 9.8 Hz, 1H), 5.72 (ddd, *J* = 17.4, 10.5, 7.0 Hz, 1H), 5.18 – 5.02 (m, 2H), 3.53 (d, *J* = 6.9 Hz, 2H), 3.39 – 3.27 (m, 1H), 0.18 (s, 9H), 0.09 (s, 9H).

^{13}C NMR (101 MHz, $CDCl_3$): δ 153.9, 137.1, 116.9, 65.0, 52.2, 2.1, 0.4.

HRMS (EI) m/z calculated for $C_{11}H_{23}OSi_2$ [M−Me]$^+$, 227.1282 found, 227.1278.

Specific rotation: $[\alpha]_D^{25}$ −1.9 (c = 1.0 M, CH_2Cl_2).

IR (neat): 3749, 2989, 2926, 2853, 2318, 1463, 1275, 1262, 749 cm^{-1}.

HPLC: Chiralpak IB column (250 mm), detected at 210 nm, hexane/i-propanol = 99.6/0.4, flow = 1.0 mL/min, retention time: 4.4 min (major), 3.1 min (minor).

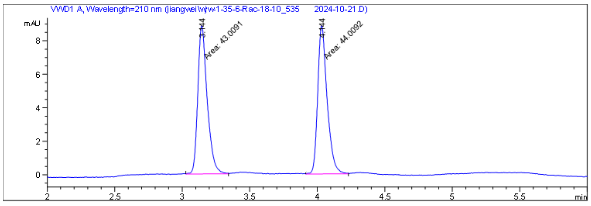 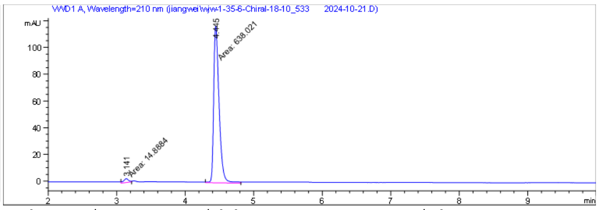

Peak #	RetTime [min]	Type	Width [min]	Area [mAU*s]	Height [mAU]	Area %
1	3.144	MM	0.0806	43.00909	8.89624	49.6626
2	4.144	MM	0.0840	44.00915	8.89802	50.3374

Peak #	RetTime [min]	Type	Width [min]	Area [mAU*s]	Height [mAU]	Area %
1	3.141	MM	0.0846	14.88836	2.93215	2.2803
2	4.445	MM	0.0902	638.02142	117.94233	97.7197

(*R*)-2-(phenylethynyl)but-3-en-1-ol (29a)

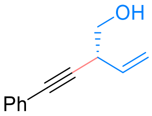

Prepared following a modified general procedure **I**, with a reaction time of 12 h, 0.2 mmol scale and 0.001 M concentration. Purification by flash column chromatography (hexane/ethyl acetate = 91:9, R_f = 0.35) gave the title compound (15.3 mg, 0.178 mmol, 89% yield, 80% ee) as a white oil.

NMR Spectroscopy (*see spectra*):

^{1}H NMR (400 MHz, $CDCl_3$): δ 7.46 – 7.40 (m, 2H), 7.31 – 7.27 (m, 3H), 5.85 (ddd, *J* = 16.6, 10.1, 6.2 Hz, 1H), 5.48 (d, *J* = 17.0 Hz, 1H), 3.78 – 3.67 (m, 2H), 3.52 (q, *J* = 6.2 Hz, 1H), 1.86 (t, *J* = 6.7 Hz, 1H).

^{13}C NMR (101 MHz, $CDCl_3$): δ 134.5, 132.2, 128.7, 128.6, 123.4, 118.4, 87.4, 85.5, 65.8, 40.3, 30.1.

HRMS (ESI) m/z calculated for $C_{12}H_{13}O$ $[M+H]^+$, 173.0961 found, 173.0959.

Specific rotation: $[\alpha]_D^{23.4}$ −64.6 (c = 5.26 M, CH_2Cl_2).

IR (neat): 3376, 2987, 2874, 1597, 1489, 1275, 1260, 1050, 921, 751, 690 cm^{-1}.

HPLC: Chiralpak AD-H column (250 mm), detected at 254 nm, hexane/i-propanol = 97/3, flow = 1.0 mL/min, retention time: 13.5 min (major), 15.4 min (minor).

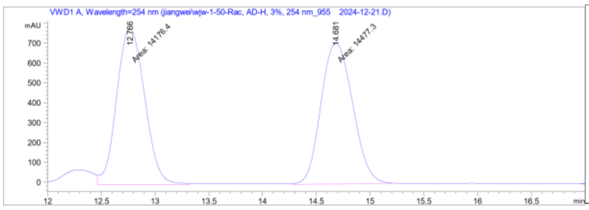

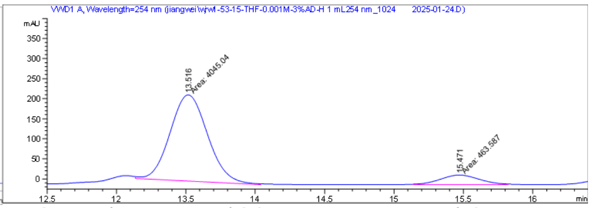

Peak #	RetTime [min]	Type	Width [min]	Area [mAU*s]	Height [mAU]	Area %
1	12.766	MM	0.3021	1.41764e4	782.15790	49.4749
2	14.681	MM	0.3410	1.44773e4	707.60938	50.5251

Peak #	RetTime [min]	Type	Width [min]	Area [mAU*s]	Height [mAU]	Area %
1	13.516	MM	0.3138	4045.03564	214.87332	89.7178
2	15.471	MM	0.3276	463.58667	23.58449	10.2822

(R)-2-(thiophen-3-ylethynyl)but-3-en-1-ol (29b)

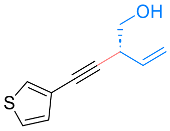

Prepared following a modified general procedure **I**, with a reaction time of 12 h, 0.2 mmol scale and 0.001 M concentration. Purification by flash column chromatography (hexane/ethyl acetate = 80:20, R_f = 0.6) gave the title compound (23.1 mg, 0.13 mmol, 65% yield, 81% ee) as a white oil.

NMR Spectroscopy (*see spectra*):

^{1}H NMR (500 MHz, $CDCl_3$) δ 7.44 (m, 1H), 7.27 (m, 1H), 7.12 (m, 1H), 5.86 (ddd, *J* = 16.9, 10.1, 6.3 Hz, 1H), 5.48 (dt, *J* = 16.9, 1.4 Hz, 1H), 5.29 (dt, *J* = 10.1, 1.4 Hz, 1H), 3.80 – 3.68 (m, 2H), 3.52 (qt, *J* = 6.1, 1.4 Hz, 1H), 1.92 –1.82 (m, 1H).

^{13}C NMR (126 MHz, $CDCl_3$) δ 134.2, 130.1, 128.7, 125.3, 122.1, 118.1, 86.7, 80.2, 65.4, 40.0.

HRMS (ESI) m/z calculated for $C_{10}H_{11}OS$ $[M+H]^+$, 179.0525 found, 179.0519.

Specific rotation: $[\alpha]_D^{22.1}$ −7.5 (c = 0.03 M, CH_2Cl_2).

IR (neat): 3399, 3006, 1358, 1275, 1260, 1053, 926, 764, 750 cm^{-1}.

HPLC: Chiralpak AD-H column (250 mm), detected at 254 nm, hexane/i-propanol = 95/5, flow = 1.0 mL/min, retention time: 11.5 min (major), 12.6 min (minor).

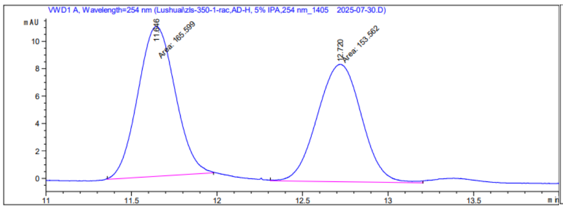

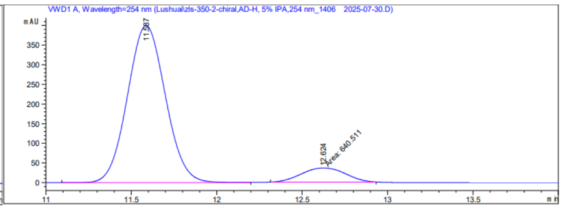

Peak #	RetTime [min]	Type	Width [min]	Area [mAU*s]	Height [mAU]	Area %
1	11.646	MM	0.2521	165.59880	10.94696	51.8856
2	12.720	MM	0.2989	153.56230	8.56286	48.1144

Peak #	RetTime [min]	Type	Width [min]	Area [mAU*s]	Height [mAU]	Area %
1	11.587	BV	0.2445	6171.96631	398.98184	90.5980
2	12.624	MM	0.2985	640.51086	35.76078	9.4020

(R)-2-vinyloct-3-yn-1-ol (29c)

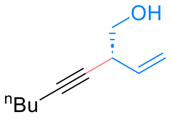

Prepared following a modified general procedure **I**, with a reaction time of 12 h, 0.2 mmol scale and 0.001 M concentration. Purification by flash column chromatography (hexane/ethyl acetate = 90:10, R_f = 0.4) gave the title compound (18.2 mg, 0.12 mmol, 60% yield, 34% ee) as a white oil.

NMR Spectroscopy (see spectra):

^{1}H NMR (500 MHz, $CDCl_3$) δ 5.83 – 5.71 (m, 1H), 5.39 (dt, *J* = 17.1, 1.6 Hz, 1H), 5.21 (dt, *J* = 10.1, 1.4 Hz, 1H), 3.67 – 3.52 (m, 2H), 3.32 – 3.24 (m, 1H), 2.23 (td, *J* = 7.0, 2.2 Hz, 2H), 1.80 (t, *J* = 6.8 Hz, 1H), 1.58 (s, 1H), 1.55 – 1.36 (m, 4H), 0.92 (t, *J* = 7.3 Hz, 3H).

^{13}C NMR (126 MHz, $CDCl_3$) δ 135.0, 117.5, 85.7, 65.6, 39.5, 31.1, 22.1, 18.6, 13.7.

HRMS (ESI) m/z calculated for $C_{10}H_{15}O$ $[M-H]^+$, 151.1117 found, 151.1116.

Specific rotation: $[\alpha]_D^{21.8}$ −30 (c = 0.02 M, CH_2Cl_2).

IR (neat): 3446, 2928, 1464, 1275, 1260, 1104, 923, 764, 750 cm^{-1}.

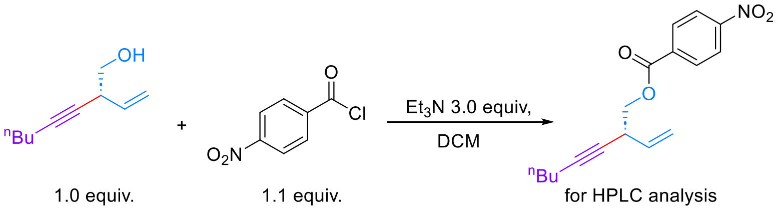

HPLC: Chiralpak AD-H column (250 mm), detected at 254 nm, hexane/i-propanol = 96/4, flow = 1.0 mL/min, retention time: 5.9 min (major), 5.6 min (minor).

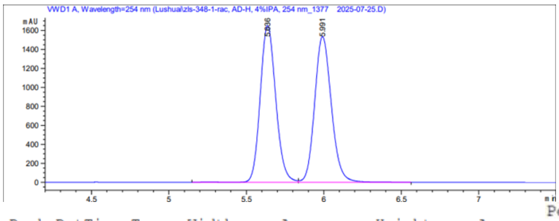

Peak #	RetTime [min]	Type	Width [min]	Area [mAU*s]	Height [mAU]	Area %
1	5.636	VV R	0.1089	1.15026e4	1649.51904	50.0138
2	5.991	VB	0.1165	1.14963e4	1534.58838	49.9862

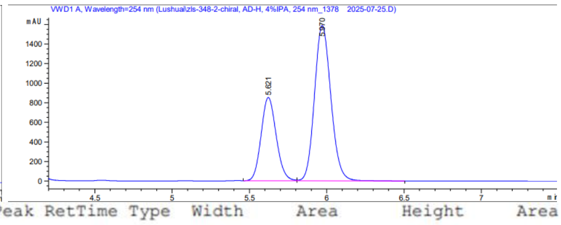

Peak #	RetTime [min]	Type	Width [min]	Area [mAU*s]	Height [mAU]	Area %
1	5.621	BV	0.1054	5771.09619	850.27295	32.9965
2	5.970	VB	0.1153	1.17190e4	1586.55908	67.0035

(*R*)-2-phenylbut-3-en-1-ol (30)

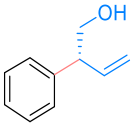

Prepared following general procedure **II**, with a reaction time of 36 h. Purification by flash column chromatography (hexane/ethyl acetate = 91:9, R_f = 0.3) gave the title compound (21.9 mg, 0.148 mmol, 74% yield, 99% ee) as a white oil. The analytical data of the compound was in complete agreement with the literature.[8]

NMR Spectroscopy (see spectra):

^{1}**H NMR** (400 MHz, $CDCl_3$): δ 7.36 – 7.30 (m, 2H), 7.26 – 7.19 (m, 3H), 6.00 (ddd, *J* = 17.1, 10.4, 7.7 Hz, 1H), 5.28 – 4.89 (m, 2H), 3.80 (dd, *J* = 7.1, 1.2 Hz, 2H), 3.52 (q, *J* = 7.3 Hz, 1H).

^{13}**C NMR** (101 MHz, $CDCl_3$): δ 140.7, 138.3, 128.8, 128.0, 127.0, 117.1, 66.1, 52.6.

Specific rotation: $[\alpha]_D^{25.0}$ −40.0 (c = 1.0 M, CH_2Cl_2).

IR (neat): 3358, 2988, 2874, 1637, 1601, 1492, 1452, 1275, 1260, 916, 750, 698 cm^{-1}.

HPLC: Chiralpak AD-H column (250 mm), detected at 210 nm, hexane/i-propanol = 99.8/0.2, flow = 1.0 mL/min, retention time: 31.6 min (major), 35.5 min (minor).

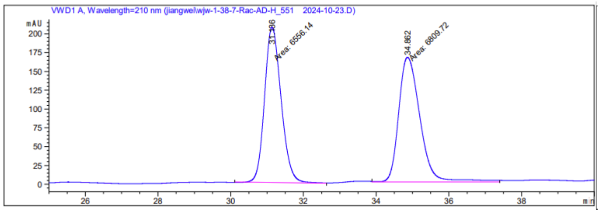

Peak #	RetTime [min]	Type	Width [min]	Area [mAU*s]	Height [mAU]	Area %
1	31.136	MM	0.5286	6556.13525	206.69797	49.0514
2	34.862	MM	0.6840	6809.71826	165.93478	50.9486

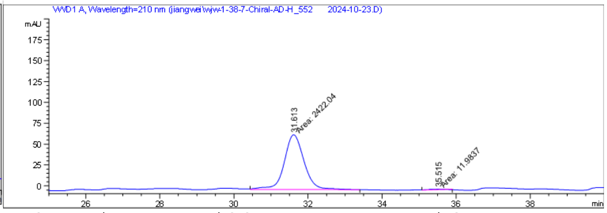

Peak #	RetTime [min]	Type	Width [min]	Area [mAU*s]	Height [mAU]	Area %
1	31.613	MM	0.6171	2422.04150	65.40961	99.5077
2	35.515	MM	0.5495	11.98369	3.63498e-1	0.4923

(*R*)-2-(p-tolyl)but-3-en-1-ol (31)

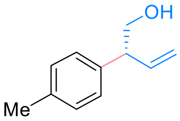

Prepared following general procedure **II**, with a reaction time of 36 h. Purification by flash column chromatography (hexane/ethyl acetate = 91:9, R_f = 0.3) gave the title compound (24.6 mg, 0.152 mmol, 76% yield, 98% ee) as a white oil. The analytical data of the compound was in complete agreement with the literature.[8]

NMR Spectroscopy (<u>*see spectra*</u>):

^{1}**H NMR** (400 MHz, $CDCl_3$): δ 7.14 (d, *J* = 3.9 Hz, 4H), 5.99 (ddd, *J* = 17.1, 10.5, 7.8 Hz, 1H), 5.25 – 5.13 (m, 2H), 3.80 (d, *J* = 6.7 Hz, 2H), 3.50 (q, *J* = 7.5 Hz, 1H), 2.33 (s, 3H).

^{13}**C NMR** (101 MHz, $CDCl_3$): δ 138.4, 137.5, 136.6, 129.5, 127.9, 117.0, 66.1, 52.2, 21.1.

Specific rotation: $[\alpha]_D^{23.3}$ −28.0 (c = 1.0 M, CH_2Cl_2).

IR (neat): 3377, 2988, 2923, 2868, 1513, 1275, 1260, 749 cm^{-1}.

HPLC: Chiralpak AD-H column (250 mm), detected at 210 nm, hexane/i-propanol = 98/2, flow = 1.0 mL/min, retention time: 18.8 min (major), 21.8 min (minor).

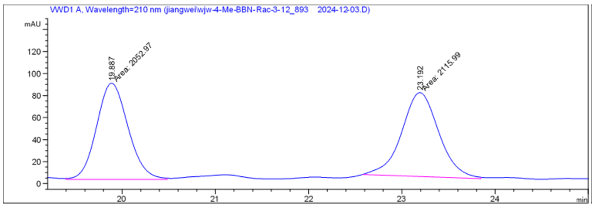

Peak #	RetTime [min]	Type	Width [min]	Area [mAU*s]	Height [mAU]	Area %
1	19.887	MM	0.3916	2052.97217	87.38331	49.2442
2	23.192	MM	0.4631	2115.98730	76.15588	50.7558

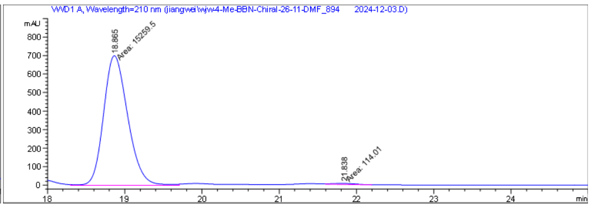

Peak #	RetTime [min]	Type	Width [min]	Area [mAU*s]	Height [mAU]	Area %
1	18.865	MM	0.3633	1.52595e4	700.05505	99.2584
2	21.838	MM	0.3504	114.00999	5.42277	0.7416

(*R*)-2-(4-chlorophenyl)but-3-en-1-ol (32)

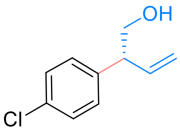

Prepared following general procedure **II**, with a reaction time of 36 h. Purification by flash column chromatography (hexane/ethyl acetate = 91:9, R_f = 0.3) gave the title compound (24.6 mg, 0.144 mmol, 72% yield, 97% ee) as a white oil. The analytical data of the compound was in complete agreement with the literature.[8]

NMR Spectroscopy (<u>*see spectra*</u>):

^{1}H NMR (400 MHz, $CDCl_3$): δ 7.31 (d, *J* = 8.4 Hz, 2H), 7.18 (d, *J* = 8.4 Hz, 2H), 5.97 (ddd, *J* = 17.8, 10.4, 7.7 Hz, 1H), 5.27 – 5.12 (m, 2H), 3.87 – 3.75 (m, 2H), 3.54 – 3.47 (m, 1H).

^{13}C NMR (101 MHz, $CDCl_3$): δ 137.7, 129.4, 128.9, 117.6, 65.9, 51.8, 29.8.

Specific rotation: $[\alpha]_D^{23.3}$ −49.6 (c = 0.8 M, CH_2Cl_2).

IR (neat): 3414, 2989, 2924, 2857, 1462, 1275, 1260, 749 cm^{-1}.

HPLC: Chiralpak AD-H column (250 mm), detected at 210 nm, hexane/i-propanol = 98/2, flow = 1.0 mL/min, retention time: 22.9 min (major), 26.3 min (minor).

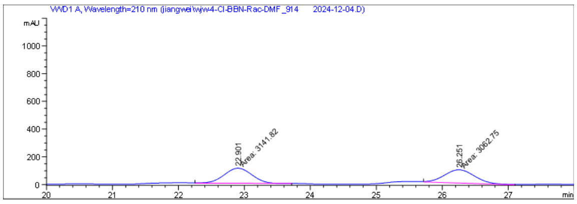

Peak #	RetTime [min]	Type	Width [min]	Area [mAU*s]	Height [mAU]	Area %
1	22.901	MM	0.4850	3141.81665	107.95889	50.6372
2	26.251	MM	0.5353	3062.74854	95.35518	49.3628

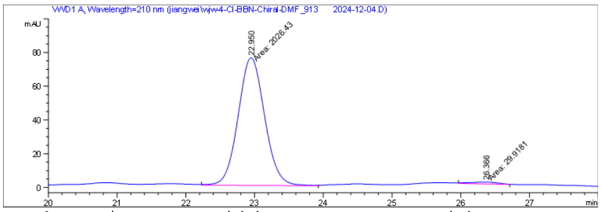

Peak #	RetTime [min]	Type	Width [min]	Area [mAU*s]	Height [mAU]	Area %
1	22.950	MM	0.4465	2026.43396	75.64936	98.5451
2	26.366	MM	0.4550	29.91806	1.09586	1.4549

(*R*)-2-(4-fluorophenyl)but-3-en-1-ol (33)

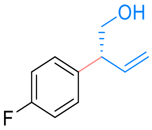

Prepared following general procedure **II**, with a reaction time of 36 h. Purification by flash column chromatography (hexane/ethyl acetate = 91:9, R_f = 0.3) gave the title compound (22.9 mg, 0.138 mmol, 69% yield, 99% ee) as a white oil. The analytical data of the compound was in complete agreement with the literature.[8]

NMR Spectroscopy (<u>*see spectra*</u>):

^{1}H NMR (400 MHz, $CDCl_3$): δ 7.20 (dd, *J* = 8.5, 5.5 Hz, 2H), 7.03 (t, *J* = 8.7 Hz, 2H), 5.97 (ddd, *J* = 17.5, 10.3, 7.5 Hz, 1H), 5.27 – 5.10 (m, 2H), 3.81 (t, *J* = 6.6 Hz, 2H), 3.52 (q, *J* = 7.5 Hz, 1H). 1.46 (t, *J* = 6.3 Hz, 1H).

^{13}C NMR (101 MHz, $CDCl_3$): δ 138.1, 129.5 (d, *J* = 7.8 Hz), 117.3, 115.6 (d, *J* = 21.1 Hz), 66.1, 51.7.

^{19}F NMR (376 MHz, $CDCl_3$): δ −115.8.

Specific rotation: $[\alpha]_D^{23.3}$ −44.0 (c = 0.45 M, CH_2Cl_2).

IR (neat): 3401, 2989, 2925, 2859, 1509, 1275, 1260, 749 cm^{-1}.

HPLC: Chiralpak IB column (250 mm), detected at 210 nm, hexane/i-propanol = 98/2, flow = 1.0 mL/min, retention time: 8.9 min (major), 13.1 min (minor).

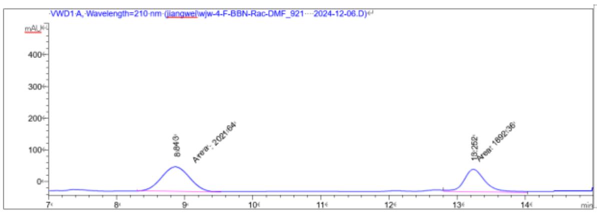

Peak #	RetTime [min]	Type	Width [min]	Area [mAU*s]	Height [mAU]	Area %
1	8.843	MM	0.1883	2021.64221	178.90379	51.6516
2	13.252	MM	0.3567	1892.35791	88.41318	48.3484

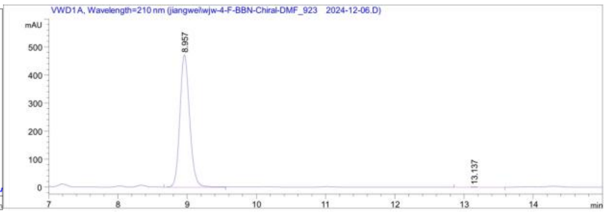

Peak #	RetTime [min]	Type	Width [min]	Area [mAU*s]	Height [mAU]	Area %
1	8.957	BV	0.1522	4621.45068	471.70670	99.6794
2	13.137	BB	0.3121	14.86610	6.75339e-1	0.3206

(*R*)-2-(o-tolyl)but-3-en-1-ol (34)

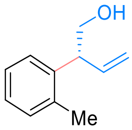

Prepared following general procedure **II**, with a reaction time of 36 h. Purification by flash column chromatography (hexane/ethyl acetate = 91:9, R_f = 0.3) gave the title compound (22.0 mg, 0.136 mmol, 68% yield, 96% ee) as a white oil. The analytical data of the compound was in complete agreement with the literature.[8]

NMR Spectroscopy (*see spectra*):

^{1}H NMR (400 MHz, $CDCl_3$): δ 7.25 – 7.11 (m, 4H), 5.96 (ddd, *J* = 17.1, 10.3, 6.9 Hz, 1H), 5.29 – 5.03 (m, 2H), 3.88 – 3.75 (m, 3H), 2.37 (s, 3H).

^{13}C NMR (101 MHz, $CDCl_3$): δ 138.6, 138.2, 136.7, 130.8, 126.7, 126.5, 126.4, 65.4, 47.9, 19.7.

Specific rotation: $[\alpha]_D^{23.5}$ −16.0 (c = 1.0 M, CH_2Cl_2).

IR (neat): 3392, 2989, 2926, 2863, 1275, 1260, 750 cm^{-1}.

HPLC: Chiralpak OD-H column (250 mm), detected at 210 nm, hexane/i-propanol = 98/2, flow = 1.0 mL/min, retention time: 18.2 min (minor), 20.4 min (major).

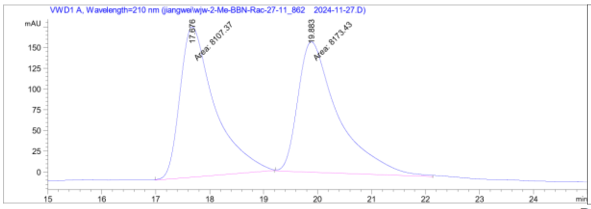

Peak #	RetTime [min]	Type	Width [min]	Area [mAU*s]	Height [mAU]	Area %
1	17.676	MM	0.7456	8107.36621	181.21587	49.7971
2	19.883	MM	0.8659	8173.43213	157.31786	50.2029

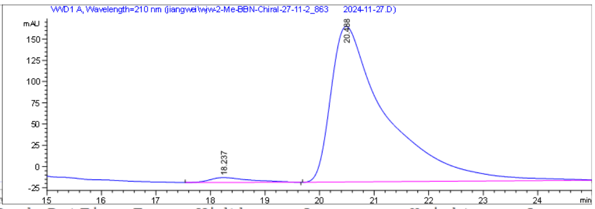

Peak #	RetTime [min]	Type	Width [min]	Area [mAU*s]	Height [mAU]	Area %
1	18.237	BB	0.7341	296.97592	5.77289	2.2316
2	20.488	BBA	0.9914	1.30107e4	183.25810	97.7684

(*R*)-2-(perfluorophenyl)but-3-en-1-ol (35)

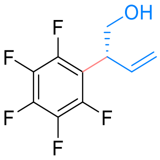

Prepared following general procedure **II**, with a reaction time of 36 h. Purification by flash column chromatography (hexane/ethyl acetate = 83:17, R_f = 0.2) gave the title compound (21.4 mg, 0.09 mmol, 45% yield, 80% ee) as a white oil.

NMR Spectroscopy (<u>*see spectra*</u>):

^{1}H NMR (400 MHz, $CDCl_3$): δ 5.83 – 5.49 (m, 1H), 5.47 – 5.00 (m, 2H), 4.18 – 3.85 (m, 1H), 3.72 – 3.30 (m, 2H).

^{13}C NMR (101 MHz, $CDCl_3$): δ 134.6-134.8 (m), 134.3, 118.2-118.6 (m), 116.3, 116.2, 65.4, 36.5.

^{19}F NMR (376 MHz, $CDCl_3$): δ −133.3 – −137.8 (m), −157.8 (t, *J* = 20.6 Hz), −161.5 – −167.4 (m).

HRMS (ESI) m/z calculated for $C_{10}H_7F_5OK$ $[M+K]^+$, 277.0049 found, 277.0048.

Specific rotation: $[\alpha]_D^{25.0}$ −16.0 (c = 1.0 M, CH_2Cl_2).

IR (neat): 3402, 2989, 2926, 2860, 1509, 1275, 1260, 749 cm^{-1}.

HPLC: Chiralpak IA column (250 mm), detected at 254 nm, hexane/i-propanol = 98/2, flow = 1.0 mL/min, retention time: 14.8 min (major), 15.6 min (minor).

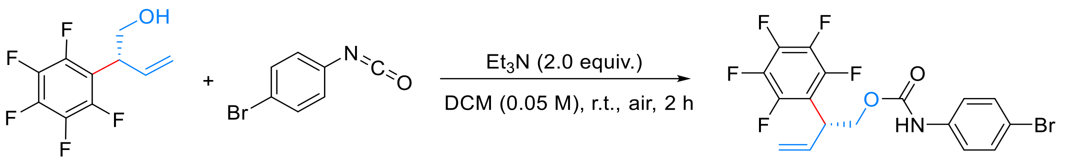

33: 0.1 mmol (1.0 equiv.) **33a** (2.0 equiv.) **33b**, 91% yield

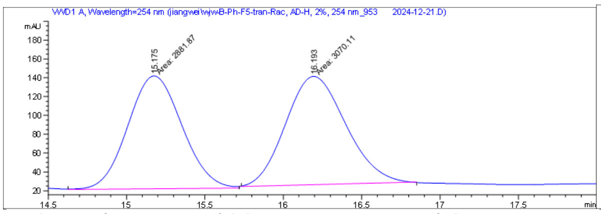

Peak #	RetTime [min]	Type	Width [min]	Area [mAU*s]	Height [mAU]	Area %
1	15.175	MM	0.4011	2881.86841	119.74569	48.4186
2	16.193	MM	0.4449	3070.11304	114.99850	51.5814

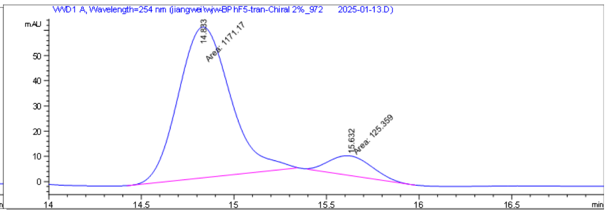

Peak #	RetTime [min]	Type	Width [min]	Area [mAU*s]	Height [mAU]	Area %
1	14.833	MM	0.3256	1171.16711	59.94985	90.3312
2	15.632	MM	0.2681	125.35862	7.79300	9.6688

(*R*,*E*)-4-methyl-N-(4-(o-tolyl)-2-vinylbut-3-en-1-yl)benzenesulfonamide (36)

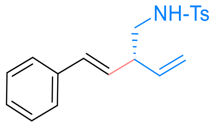

Prepared following general procedure **I**, with a reaction time of 18 h. Purification by flash column chromatography (Et_2O/hexane = 33:67, R_f = 0.3) gave the title compound (39.2 mg, 0.12 mmol, 60% yield, 89% ee).

NMR Spectroscopy (<u>*see spectra*</u>):

^{1}H NMR (400 MHz, $CDCl_3$): δ 7.71 (d, *J* = 8.4 Hz, 2H), 7.27 (s, 2H), 7.24 – 7.18 (m, 1H), 6.33 (d, *J* = 16.0 Hz, 1H), 5.90 (dd, *J* = 16.0, 7.3 Hz, 1H), 5.64 (ddd, *J* = 17.3, 10.3, 6.8 Hz, 1H), 5.17 – 5.04 (m, 2H), 4.50 (t, *J* = 6.0 Hz, 1H), 3.07 – 2.95 (m, 3H), 2.40 (s, 3H).

^{13}C NMR (101 MHz, $CDCl_3$): δ 143.6, 137.1, 137.0, 136.7, 132.6, 129.8, 128.7, 128.1, 127.8, 127.2, 126.3, 117.7, 46.9, 46.4, 21.6.

HRMS (ESI) m/z calculated for $C_{19}H_{22}NO_2S$ $[M+H]^+$, 328.1293 found, 328.1363.

Specific rotation: $[\alpha]_D^{24.8}$ = −18.0 (c = 2.0 M, CH_2Cl_2).

IR (neat): 3280, 2988, 2924, 2865, 1323, 1275, 1260, 1155, 1092, 749 cm^{-1}.

HPLC: Chiralpak IB column (250 mm), detected at 230 nm, hexane/i-propanol = 90/10, flow rate = 1.0 mL/min, 11.9 min (major), 9.7 min (minor).

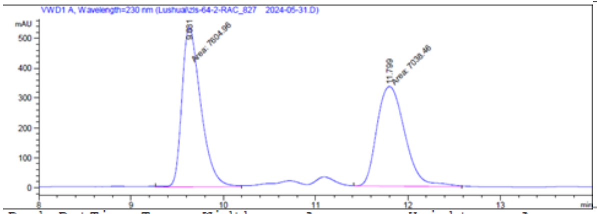

Peak #	RetTime [min]	Type	Width [min]	Area [mAU*s]	Height [mAU]	Area %
1	9.631	MM	0.2371	7604.95557	534.62213	51.9343
2	11.799	MM	0.3514	7038.46143	333.82394	48.0657

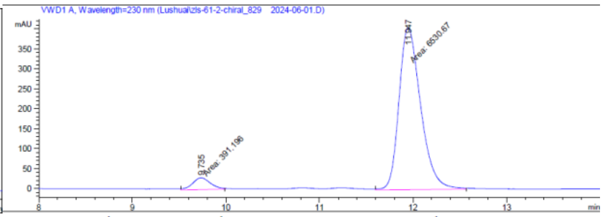

Peak #	RetTime [min]	Type	Width [min]	Area [mAU*s]	Height [mAU]	Area %
1	9.735	MM	0.2219	391.19598	29.38805	5.6516
2	11.947	MM	0.2681	6530.67480	406.05121	94.3484

(*R*,*E*)-4-methyl-N-(4-(p-tolyl)-2-vinylbut-3-en-1-yl)benzenesulfonamide (37)

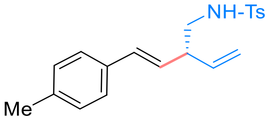

Prepared following general procedure **I**, with a reaction time of 18 h. Purification by flash column chromatography (Et_2O/hexane= 33:67, R_f = 0.3) gave the title compound (42.9 mg, 0.126 mmol, 63% yield, 93% ee).

NMR Spectroscopy (<u>*see spectra*</u>):

^{1}H NMR (400 MHz, $CDCl_3$): δ 7.74 (d, *J* = 8.4 Hz, 2H), 7.30 (d, *J* = 8.3 Hz, 2H), 7.19 (d, *J* = 8.3 Hz, 2H), 7.10 (d, *J* = 8.1 Hz, 2H), 6.33 (d, *J* = 15.9 Hz, 1H), 5.86 (dd, *J* = 16.0, 7.3 Hz, 1H), 5.67 (ddd, *J* = 17.1, 10.4, 6.9 Hz, 1H), 5.18 – 5.06 (m, 2H), 4.47 (t, *J* = 6.0 Hz, 1H), 3.09 – 2.95 (m, 3H), 2.43 (s, 3H), 2.33 (s, 3H).

^{13}C NMR (101 MHz, $CDCl_3$): δ 143.6, 137.7, 137.2, 137.0, 133.9, 132.5, 129.8, 129.3, 127.2, 127.0, 126.2, 117.6, 46.9, 46.4, 21.6, 21.3.

HRMS (ESI) m/z calculated for $C_{20}H_{24}NO_2S$ $[M+H]^+$, 342.1449 found, 342.1518.

Specific rotation: $[\alpha]_D^{24.1}$ = −18.2 (c = 1.1 M, CH_2Cl_2).

IR (neat): 3280, 2988, 2857, 1741, 1325, 1275, 1260, 1157, 1092, 750 cm^{-1}.

HPLC: Chiralpak IB column (250 mm), detected at 254 nm, hexane/i-propanol = 93/7, flow rate = 1.0 mL/min, 15.0 min (major), 14.0 min (minor).

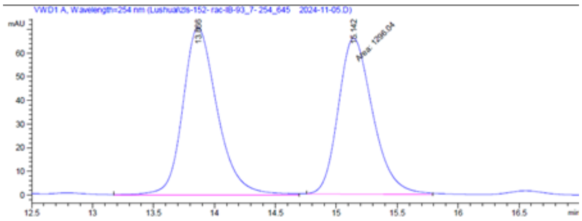

Peak #	RetTime [min]	Type	Width [min]	Area [mAU*s]	Height [mAU]	Area %
1	13.866	BV	0.3005	1387.18762	70.62064	51.6985
2	15.142	MM	0.3281	1296.03931	65.84500	48.3015

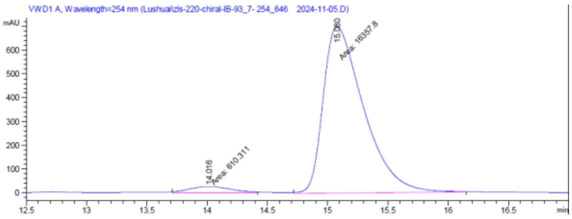

Peak #	RetTime [min]	Type	Width [min]	Area [mAU*s]	Height [mAU]	Area %
1	14.016	MM	0.4064	610.31140	25.02877	3.5968
2	15.080	MM	0.3881	1.63578e4	702.39478	96.4032

(*R*,*E*)-N-(4-(4-methoxyphenyl)-2-vinylbut-3-en-1-yl)-4-methylbenzenesulfonamide (38)

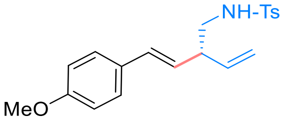

Prepared following general procedure **I**, with a reaction time of 18 h. Purification by flash column chromatography (Et_2O/hexane= 33:67, R_f = 0.2) gave the title compound (32.1 mg, 0.06 mmol, 45% yield, 94% ee).

NMR Spectroscopy (*see spectra*):

^{1}H NMR (400 MHz, $CDCl_3$): δ 7.74 (d, *J* = 8.4 Hz, 2H), 7.30 (d, *J* = 8.3 Hz, 2H), 7.23 (d, *J* = 8.7 Hz, 2H), 6.83 (d, *J* = 8.9 Hz, 2H), 6.30 (d, *J* = 15.7 Hz, 1H), 5.77 (dd, *J* = 15.9, 7.3 Hz, 1H), 5.66 (ddd, *J* = 17.1, 10.4, 6.9 Hz, 1H), 5.18 – 5.05 (m, 2H), 4.48 (t, *J* = 6.0 Hz, 1H), 3.81 (s, 3H), 3.03 (dq, *J* = 11.9, 6.0 Hz, 2H), 3.00 – 2.93 (m, 1H), 2.43 (s, 3H).

^{13}C NMR (101 MHz, $CDCl_3$): δ 159.4, 143.6, 137.3, 137.0, 132.1, 129.8, 129.4, 127.5, 127.2, 125.8, 117.5, 114.1, 55.4, 46.9, 46.4, 21.6.

HRMS (ESI) m/z calculated for $C_{20}H_{24}NO_3S[M+H]^+$, 358.139 found, 358.1469.

Specific rotation: $[\alpha]_D^{23.4}$ = −18.5 (c = 1.3 M, CH_2Cl_2).

IR (neat): 3283, 2989, 2926, 1606, 1510, 1275, 1259, 1157, 750 cm^{-1}.

HPLC: Chiralpak IA column (250 mm), detected at 254 nm, hexane/i-propanol = 90/10, flow rate = 1.0 mL/min, 20.9 min (major), 23.6 min (minor).

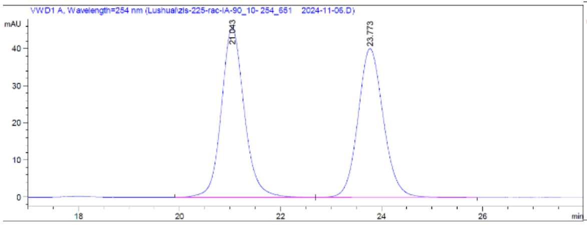

Peak #	RetTime [min]	Type	Width [min]	Area [mAU*s]	Height [mAU]	Area %
1	21.043	BB	0.4859	1438.93054	45.62827	50.6408
2	23.773	BB	0.5361	1402.51196	40.10682	49.3592

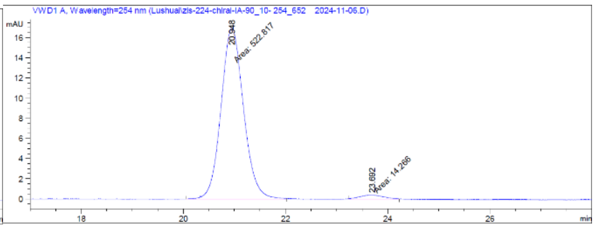

Peak #	RetTime [min]	Type	Width [min]	Area [mAU*s]	Height [mAU]	Area %
1	20.948	MM	0.5132	522.81677	16.97889	97.3438
2	23.692	MM	0.5553	14.26596	4.28189e-1	2.6562

Methyl (R,E)-4-(3-(((4-methylphenyl)sulfonamido)methyl)penta-1,4-dien-1-yl)benzoate (39)

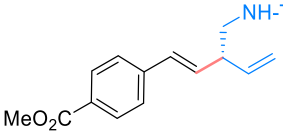

Prepared following general procedure **I**, with a reaction time of 18 h. Purification by flash column chromatography (Et_2O/hexane = 33:67, R_f = 0.2) gave the title compound (30.8 mg, 0.08 mmol, 40% yield, 98% ee).

NMR Spectroscopy (*see spectra*):

^{1}H NMR (400 MHz, $CDCl_3$): δ 7.96 (d, *J* = 8.4 Hz, 2H), 7.74 (d, *J* = 8.4 Hz, 2H), 7.31 (dd, *J* = 19.4, 8.6 Hz, 4H), 6.40 (d, *J* = 16.0 Hz, 1H), 6.07 (dd, *J* = 15.9, 7.2 Hz, 1H), 5.67 (ddd, *J* = 17.3, 10.3, 7.0 Hz, 1H), 5.21 – 5.08 (m, 2H), 4.57 (t, *J* = 6.1 Hz, 1H), 3.91 (s, 3H), 3.11 – 3.01 (m, 3H), 2.42 (s, 3H).

^{13}C NMR (101 MHz, $CDCl_3$): δ 166.9, 143.7, 141.1, 137.0, 136.6, 131.7, 131.0, 130.0, 129.9, 129.2, 127.2, 126.2, 118.1, 52.2, 47.1, 46.3, 21.6.

HRMS (ESI) m/z calculated for $C_{21}H_{24}NO_4S$ $[M+H]^+$, 386.1348 found, 386.1418.

Specific rotation: $[\alpha]_D^{23.6}$ = −20.0 (c = 1.2 M, CH_2Cl_2).

IR (neat): 3280, 2989, 1716, 1606, 1434, 1276, 1158, 749 cm^{-1}.

HPLC: Chiralpak IB column (250 mm), detected at 254 nm, hexane/i-propanol = 90/10, flow rate = 1.0 mL/min, 26.9 min (major), 23.7 min (minor).

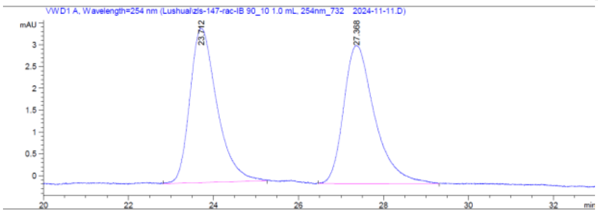

Peak #	RetTime [min]	Type	Width [min]	Area [mAU*s]	Height [mAU]	Area %
1	23.712	BB	0.5721	156.05150	3.53270	49.2360
2	27.368	BB	0.6139	160.89452	3.16244	50.7640

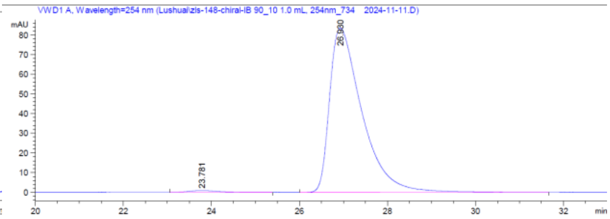

Peak #	RetTime [min]	Type	Width [min]	Area [mAU*s]	Height [mAU]	Area %
1	23.781	BB	0.5432	40.75573	9.04380e-1	0.9446
2	26.930	BB	0.7539	4274.01855	83.57241	99.0554

(*R*,*E*)-N-(4-(3,5-bis(trifluoromethyl)phenyl)-2-vinylbut-3-en-1-yl)-4-methylbenzenesulfonamide (40)

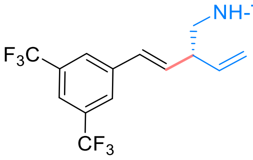

Prepared following general procedure **I**, with a reaction time of 18 h. Purification by flash column chromatography (Et_2O/hexane = 33:67, R_f = 0.1) gave the title compound (54.6 mg, 0.118 mmol, 59% yield, 94% ee).

NMR Spectroscopy (*see spectra*):

^{1}H NMR (400 MHz, $CDCl_3$): δ 7.76 – 7.67 (m, 5H), 7.28 (d, *J* = 8.4 Hz, 2H), 6.42 (d, *J* = 16.0 Hz, 1H), 6.19 – 6.10 (m, 1H), 5.67 (ddd, *J* = 17.3, 10.3, 7.0 Hz, 1H), 5.23 – 5.09 (m, 2H), 4.83 (t, *J* = 5.7 Hz, 1H), 3.09 (d, *J* = 3.5 Hz, 3H), 2.41 (s, 3H).

^{13}C NMR (101 MHz, $CDCl_3$): δ 143.7, 138.8, 136.9, 136.2, 132.7, 132.1, 131.8, 129.8, 127.2, 126.2, 124.7, 122.0, 121.0, 118.4, 47.0, 46.2, 21.6.

HRMS (ESI) m/z calculated for $C_{21}H_{20}F_6NO_2S$ $[M+H]^+$, 464.1041 found, 464.1126.

Specific rotation: $[\alpha]_D^{23.9}$ = −16.3 (c = 1.6 M, CH_2Cl_2).

IR (neat): 3291, 2988, 2924, 2863, 1324, 1275, 1260, 1156, 1092, 749 cm^{-1}.

HPLC: Chiralpak AD-H column (250 mm), detected at 230 nm, hexane/i-propanol = 97/3, flow rate = 1.0 mL/min, 15.5 min (major), 16.1 min(minor).

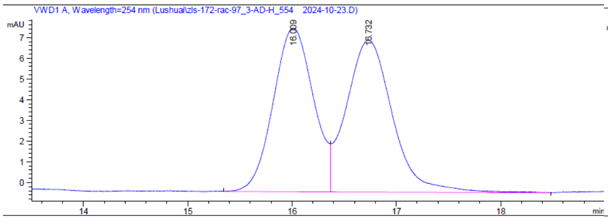

Peak #	RetTime [min]	Type	Width [min]	Area [mAU*s]	Height [mAU]	Area %
1	16.009	BV	0.4035	211.94185	7.89518	48.5463
2	16.732	VB	0.4461	224.63478	7.33447	51.4537

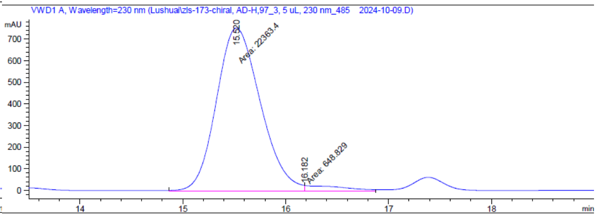

Peak #	RetTime [min]	Type	Width [min]	Area [mAU*s]	Height [mAU]	Area %
1	15.520	MF	0.4948	2.23634e4	753.30359	97.1805
2	16.182	FM	0.4241	648.82910	25.49933	2.8195

(*R*,*E*)-4-methyl-N-(4-(o-tolyl)-2-vinylbut-3-en-1-yl)benzenesulfonamide (41)

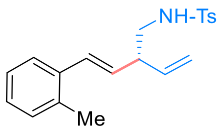

Prepared following general procedure **I**, with a reaction time of 18 h. Purification by flash column chromatography (Et_2O/hexane = 33:67, R_f = 0.2) gave the title compound (47.7 mg, 0.14 mmol, 70% yield, 97% ee).

NMR Spectroscopy (*see spectra*):

^{1}H NMR (400 MHz, $CDCl_3$): δ 7.75 (d, *J* = 8.4 Hz, 2H), 7.36 – 7.27 (m, 3H), 7.19 – 7.09 (m, 3H), 6.59 (d, *J* = 15.7 Hz, 1H), 5.88 – 5.61 (m, 2H), 5.22 – 5.06 (m, 2H), 4.53 (t, *J* = 6.0 Hz, 1H), 3.13 – 2.94 (m, 3H), 2.42 (s, 3H), 2.31 (s, 3H).

^{13}C NMR (101 MHz, $CDCl_3$): δ 143.6, 137.2, 137.0, 135.8, 135.4, 130.6, 130.4, 129.8, 129.5, 127.7, 127.2, 126.1, 125.6, 117.7, 47.2, 46.4, 21.6, 19.9.

HRMS (ESI) m/z calculated for $C_{20}H_{24}NO_2S$ $[M+H]^+$, 342.1449 found, 342.1519.

Specific rotation: $[\alpha]_D^{24.8}$ = −18.7 (c = 1.5 M, CH_2Cl_2).

IR (neat): 3281, 2988, 2924, 2863, 1324, 1275, 1260, 1156, 1092, 749 cm^{-1}.

HPLC: Chiralpak IA column (250 mm), detected at 254 nm, hexane/i-propanol = 97/3, flow rate = 1.0 mL/min, 35.2 min (major), 38.1 min (minor).

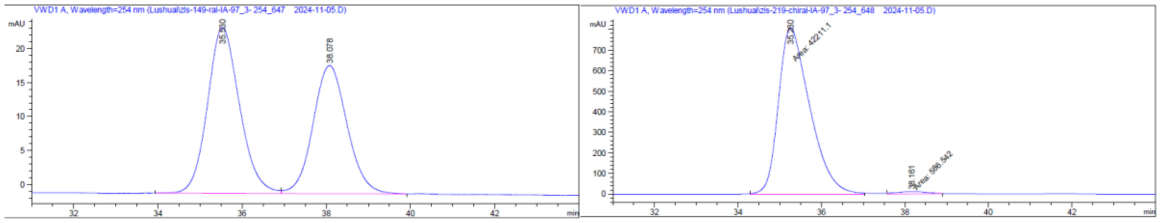

Peak #	RetTime [min]	Type	Width [min]	Area [mAU*s]	Height [mAU]	Area %
1	35.530	BV	0.7638	1295.07715	24.43990	54.8533
2	38.078	VB	0.8368	1065.90479	18.90603	45.1467

Peak #	RetTime [min]	Type	Width [min]	Area [mAU*s]	Height [mAU]	Area %
1	35.260	MM	0.8707	4.22111e4	807.98608	98.6295
2	38.161	MM	0.5976	586.54193	11.50210	1.3705

(*R*,*E*)-4-methyl-N-(4-(naphthalen-2-yl)-2-vinylbut-3-en-1-yl)benzenesulfonamide (42)

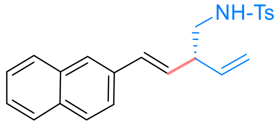

Prepared following general procedure **I**, with a reaction time of 18 h. Purification by flash column chromatography (Et_2O/hexane =33:67, R_f = 0.2) gave the title compound (39.2 mg, 0.104 mmol, 52% yield, 88% ee).

NMR Spectroscopy (*see spectra*):

^{1}H NMR (400 MHz, $CDCl_3$): δ 7.83 – 7.71 (m, 5H), 7.65 (d, *J* = 1.8 Hz, 1H), 7.53 – 7.40 (m, 3H), 7.29 (d, *J* = 8.3 Hz, 2H), 6.53 (d, *J* = 16.0 Hz, 1H), 6.06 (dd, *J* = 15.9, 7.3 Hz, 1H), 5.71 (ddd, *J* = 17.2, 10.4, 6.7 Hz, 1H), 5.22 – 5.10 (m, 2H), 3.10 (t, *J* = 5.6 Hz, 3H), 2.41 (s, 3H).

^{13}C NMR (101 MHz, $CDCl_3$): δ 143.6, 137.1, 137.0, 134.1, 133.6, 133.1, 132.7, 129.9, 128.5, 128.3, 128.0, 127.8, 127.2, 126.4, 126.3, 126.0, 123.4, 117.8, 47.1, 46.4, 21.6.

HRMS (ESI) m/z calculated for $C_{23}H_{24}NO_2S$ $[M+H]^+$, 378.1449 found, 378.1518.

Specific rotation: $[\alpha]_D^{23.4}$ = −28.9 (c = 0.9 M, CH_2Cl_2).

IR (neat): 3280, 2989, 2924, 2864, 1592, 1275, 1260, 1158, 749 cm^{-1}.

HPLC: Chiralpak IA column (250 mm), detected at 254 nm, hexane/i-propanol = 94/6, flow rate = 1.0 mL/min, 49.0 min (major), 52.4 min (minor).

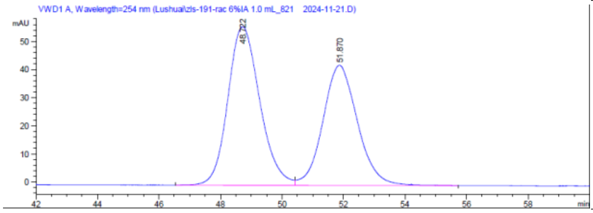

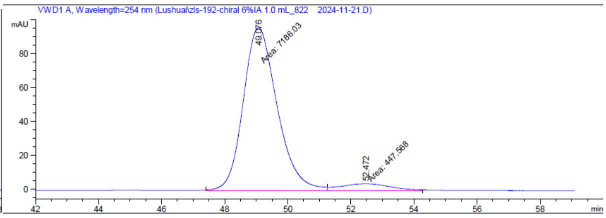

Peak #	RetTime [min]	Type	Width [min]	Area [mAU*s]	Height [mAU]	Area %
1	49.076	MF	1.2477	7186.02783	95.98770	94.1369
2	52.472	FM	1.8217	447.56827	4.09475	5.8631

Peak #	RetTime [min]	Type	Width [min]	Area [mAU*s]	Height [mAU]	Area %
1	48.722	BV	1.1163	4073.54443	56.40759	54.8897
2	51.870	VB	1.2071	3347.78467	42.77922	45.1103

(*R*)-4-methyl-N-(2-(phenylethynyl)but-3-en-1-yl)benzenesulfonamide (43)

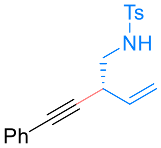

Prepared following general procedure **II**, with a reaction time of 12 h. 0.2 mmol scale, 0.1M, Purification by flash column chromatography (hexane/ethyl acetate = 83:17, R_f = 0.2) gave the title compound (52.6 mg, 0.162 mmol, 81% yield, 99% ee) as a white oil.

NMR Spectroscopy (<u>*see spectra*</u>):

^{1}H NMR (400 MHz, $CDCl_3$): δ 7.77 (d, *J* = 8.3 Hz, 2H), 7.43 – 7.34 (m, 2H), 7.33 – 7.28 (m, 5H), 5.71 (ddd, *J* = 16.9, 10.0, 6.1 Hz, 1H), 5.40 (dt, *J* = 17.0, 1.4 Hz, 1H), 5.23 (dt, *J* = 10.0, 1.3 Hz, 1H), 4.72 (t, *J* = 6.5 Hz, 1H), 3.43 (dtt, *J* = 7.5, 6.0, 1.4 Hz, 1H), 3.21 (ddd, *J* = 12.9, 7.1, 6.0 Hz, 1H), 3.10 (ddd, *J* = 12.6, 7.4, 5.7 Hz, 1H), 2.42 (s, 3H).

^{13}C NMR (101 MHz, $CDCl_3$): δ 143.6, 137.0, 133.9, 131.8, 129.8, 128.4, 128.4, 127.2, 122.6, 118.5, 86.4, 85.5, 46.7, 36.8, 21.6.

HRMS (ESI) m/z calculated for $C_{19}H_{20}NO_2S$ $[M+H]^+$, 326.1209 found, 326.1208.

Specific rotation: $[\alpha]_D^{23.4}$ −26.7 (c = 0.33 M, CH_2Cl_2).

IR (neat): 3281, 2988, 2865, 1597, 1490, 1326, 1275, 1260, 1157, 1092, 750 cm^{-1}.

HPLC: Chiralpak IB column (250 mm), detected at 254 nm, hexane/i-propanol = 97/3, flow = 1.0 mL/min, retention time: 16.0 min (major), 18.6 min (minor).

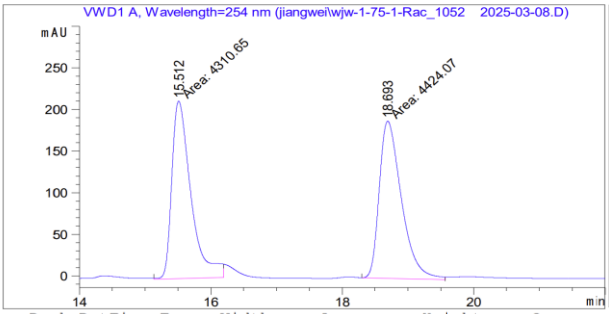

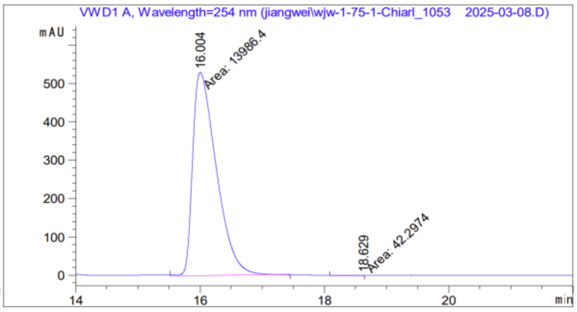

Peak #	RetTime [min]	Type	Width [min]	Area [mAU*s]	Height [mAU]	Area %
1	15.512	MM	0.3370	4310.65430	213.20689	49.3508
2	18.693	MM	0.3906	4424.06592	188.75565	50.6492

Peak #	RetTime [min]	Type	Width [min]	Area [mAU*s]	Height [mAU]	Area %
1	16.004	MM	0.4398	1.39864e4	530.07495	99.6985
2	18.629	MM	0.4900	42.29736	1.43876	0.3015

(*R*,*E*)-1-(1-phenylpenta-1,4-dien-3-yl)cyclohexan-1-ol (44)

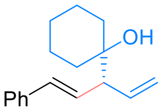

Prepared following general procedure **I**, with a reaction time of 18 h. Purification by flash column chromatography (Et_2O/hexane = 33:67, R_f = 0.2) gave the title compound (26.1 mg, 0.108 mmol, 54% yield, 30% ee).

NMR Spectroscopy (see spectra):

^{1}H NMR (400 MHz, $CDCl_3$): δ 7.38 (d, *J* = 7.0 Hz, 2H), 7.32 (d, *J* = 7.3 Hz, 2H), 7.23 (d, *J* = 7.3 Hz, 1H), 6.44 (d, *J* = 15.9 Hz, 1H), 6.31 (dd, *J* = 16.0, 8.6 Hz, 1H), 6.07 – 5.92 (m, 1H), 5.23 – 5.10 (m, 2H), 2.89 (t, *J* = 8.6 Hz, 1H), 1.69 – 1.42 (m, 10H).

^{13}C NMR (101 MHz, $CDCl_3$): δ 137.4, 137.0, 132.3, 128.6, 127.4, 126.3, 117.5, 72.9, 58.9, 35.4, 25.8, 21.9.

HRMS (EI) m/z calculated for $C_{17}H_{19}$ $[M-H_2O]^+$, 224.1560 found, 224.1557.

IR (neat): 3460, 2930, 2855, 1447, 1275, 1260, 965, 912, 750, 692 cm^{-1}.

HPLC: Chiralpak AD-H column (250 mm), detected at 254 nm, hexane/i-propanol = 96/4, flow = 1.0 mL/min, retention time: 10.0 min (major), 11.0 min (minor).

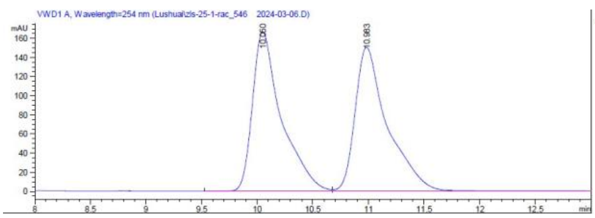

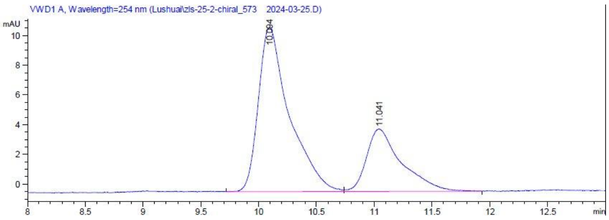

Peak #	RetTime [min]	Type	Width [min]	Area [mAU*s]	Height [mAU]	Area %
1	10.050	BV	0.2603	3012.16846	166.74873	49.7022
2	10.983	VB	0.2918	3048.26709	150.40744	50.2978

Peak #	RetTime [min]	Type	Width [min]	Area [mAU*s]	Height [mAU]	Area %
1	10.094	BV	0.2704	206.17990	11.03312	69.9439
2	11.041	VB	0.3019	88.59908	4.19183	30.0561

(*E*)-2-methyl-4-phenyl-2-vinylbut-3-en-1-ol (45)

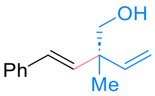

45

Prepared following general procedure **I** (reaction performed at 100 °C), with a reaction time of 18 h. Purification by flash column chromatography (Et_2O/hexane = 33:67, R_f = 0.4) gave the title compound (11.3 mg, 0.06 mmol, 30% yield, 60% ee)*.

*Reaction performed at room temperature: 15% yield, 16% ee.

NMR Spectroscopy (see spectra):

^{1}H NMR (400 MHz, $CDCl_3$): δ 7.39 (d, *J* = 7.0 Hz, 2H), 7.31 (t, *J* = 7.5 Hz, 2H), 7.25 – 7.19 (m, 1H), 6.46 (d, *J* = 16.3 Hz, 1H), 6.22 (d, *J* = 16.3 Hz, 1H), 5.92 (dd, *J* = 17.6, 10.7 Hz, 1H), 5.15 (d, *J* = 1.2 Hz, 2H), 3.55 (d, *J* = 6.2 Hz, 2H), 1.26 (s, 3H).

^{13}C NMR (101 MHz, $CDCl_3$): δ 142.4, 137.3, 134.0, 129.8, 127.5, 126.3, 115.1, 70.0, 45.5, 21.0.

HRMS (EI) m/z calculated for $C_{13}H_{16}O$ $[M]^+$, 188.1196 found, 188.1192.

IR (neat): 3391, 2988, 2927, 2869, 1447, 1275, 1260, 749 cm^{-1}.

HPLC: Chiralpak IN column (150 mm), detected at 254 nm, water/acetonitrile = 65/35, flow = 1.0 mL/min, retention time: 14.6 min (major), 13.8 min (minor).

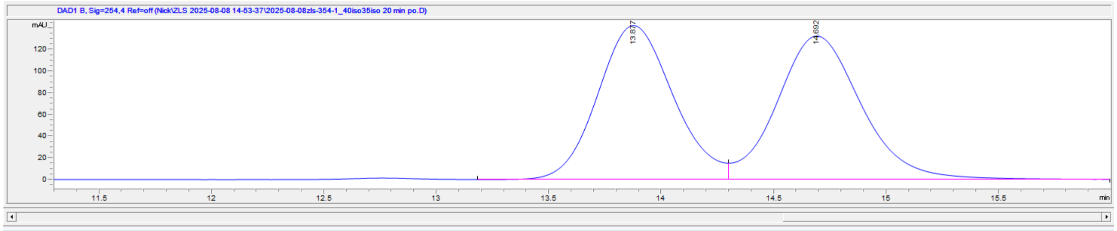

File Information

LC-File	2025-08-08zls-354-1_40iso35iso 20 min po.D
File Path	C:\Users\Public\Documents\ChemStation\1\Data\Nick\ZLS 2025-08-
Date	08-Aug-25, 16:10:20
Sample	zls-354-1_40iso
Sample Info	
Barcode	
Operator	SYSTEM
Method	35iso 20 min pos 2.M
Reference	C:\Users\Public\Documents\ChemStation\1\Data\Nick\ZLS 2025-08-
Analysis Time	15.993 min
Sampling Rate	0.0067 min (0.402 sec), 2400 datapoints

#	Time	Type	Area	Height	Width	Area%	Symmetry
1	13.877	BV	3314.4	141.9	0.3604	49.111	0.87
2	14.692	VBA	3434.3	132.2	0.3971	50.889	0.852

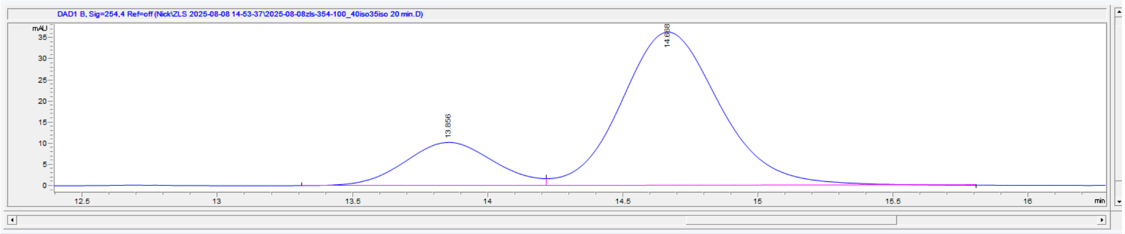

LC-File	2025-08-08zls-354-100_40iso35iso 20 min.D
File Path	C:\Users\Public\Documents\ChemStation\1\Data\Nick\ZLS 2025-08-
Date	08-Aug-25, 16:27:19
Sample	zls-354-100_40iso
Sample Info	
Barcode	
Operator	SYSTEM
Method	35iso 20 min pos 2.M
Reference	C:\Users\Public\Documents\ChemStation\1\Data\Nick\ZLS 2025-08-
Analysis Time	19.987 min
Sampling Rate	0.0067 min (0.402 sec), 2999 datapoints

#	Time	Type	Area	Height	Width	Area%	Symmetry
1	13.856	BV	234.5	10.2	0.3543	20.280	0.953
2	14.668	VB	922	36.2	0.3931	79.720	0.859

(*S*,*E*)-3-methyl-4-phenyl-2-((*E*)-styryl)but-3-en-1-ol (46)

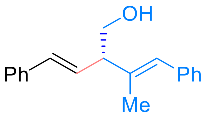

Prepared following general procedure **I**, with a reaction time of 18 h. Purification by flash column chromatography (Et_2O/hexane = 33:67, R_f = 0.2) gave the title compound (25.3 mg, 0.096 mmol, 48% yield, 17% ee).

NMR Spectroscopy (<u>*see spectra*</u>):

^{1}H NMR (500 MHz, $CDCl_3$): δ 7.40 (d, *J* = 7.1 Hz, 2H), 7.33 (q, *J* = 7.4 Hz, 4H), 7.28 (d, *J* = 6.4 Hz, 2H), 7.27 – 7.19 (m, 2H), 3.88 (dd, *J* = 10.7, 7.3 Hz, 1H), 3.80 (dd, *J* = 10.7, 6.9 Hz, 1H), 3.23 (q, *J* = 7.1 Hz, 1H), 1.92 (d, *J* = 1.4 Hz, 3H).

^{13}C NMR (126 MHz, $CDCl_3$): δ 137.5, 137.2, 136.9, 128.8, 128.4, 128.3, 128.0, 127.4, 126.3, 126.1, 63.7, 55.1, 16.0.

IR (neat): 3348, 2970, 2885, 1463, 1275, 1260, 950, 749 cm^{-1}.

HRMS (EI) m/z calculated for $C_{19}H_{20}O$ $[M]^+$, 264.1514 found, 264.1511.

HPLC: Chiralpak AD-H column (250 mm), detected at 254 nm, hexane/i-propanol = 94/6, flow = 1.0 mL/min, retention time: 14.9 min (major), 11.7 min (minor).

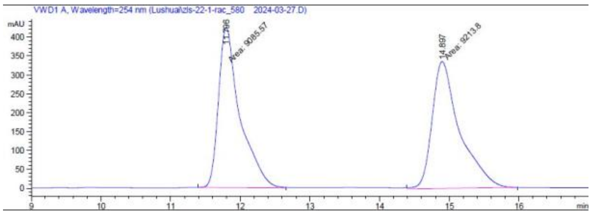

Peak #	RetTime [min]	Type	Width [min]	Area [mAU*s]	Height [mAU]	Area %
1	11.796	MM	0.3572	9085.56738	423.94693	49.6496
2	14.897	MM	0.4574	9213.80078	335.70206	50.3504

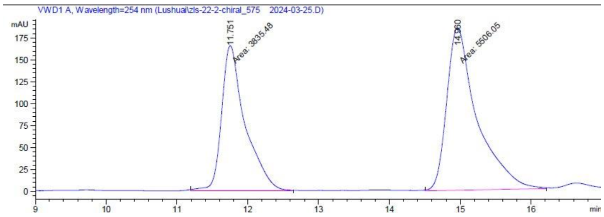

Peak #	RetTime [min]	Type	Width [min]	Area [mAU*s]	Height [mAU]	Area %
1	11.751	MM	0.3860	3835.48242	165.58853	41.0584
2	14.960	MM	0.4956	5506.04834	185.15735	58.9416

(*S*,*E*)-4-phenyl-2-((*E*)-2-(thiophen-3-yl)vinyl)but-3-en-1-ol (47)

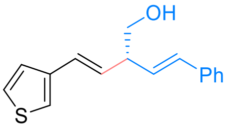

Prepared following general procedure **I**, with a reaction time of 18 h. Purification by flash column chromatography (Et_2O/hexane = 33:67, R_f = 0.2) gave the title compound (30.2 mg, 0.118 mmol, 59% yield, 3% ee).

NMR Spectroscopy (*see spectra*):

^{1}H NMR (400 MHz, $CDCl_3$): δ 7.44 – 7.40 (m, 2H), 7.37 – 7.31 (m, 3H), 7.31 – 7.23 (m, 3H), 7.17 (dd, *J* = 3.0, 1.3 Hz, 1H), 6.58 (dd, *J* = 16.0, 7.9 Hz, 2H), 6.22 (dd, *J* = 15.9, 7.6 Hz, 1H), 6.09 (dd, *J* = 15.9, 7.6 Hz, 1H), 3.76 (t, *J* = 6.3 Hz, 2H), 3.29 (p, *J* = 7.1 Hz, 1H).

^{13}C NMR (101 MHz, $CDCl_3$): δ 139.7, 137.0, 132.5, 128.7, 128.7, 128.6, 127.6, 126.7, 126.3, 126.2, 124.9, 121.9, 65.6, 49.4.

HRMS (MS-EI) m/z calculated for $C_{16}H_{16}OS$ $[M]^+$, 256.0916 found, 256.0913.

IR (neat): 3352, 2989, 1275, 1260, 749 cm^{-1}.

HPLC: Chiralpak IB column (250 mm), detected at 254 nm, hexane/i-propanol = 95/5, flow = 1.0 mL/min, retention time: 18.5 min (major), 20.3 min (minor).

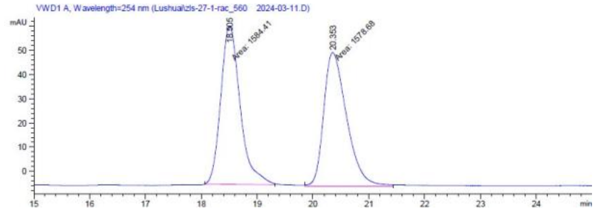

Peak #	RetTime [min]	Type	Width [min]	Area [mAU*s]	Height [mAU]	Area %
1	18.505	MM	0.4019	1584.40710	65.69708	50.0906
2	20.353	MM	0.4752	1578.67834	55.37277	49.9094

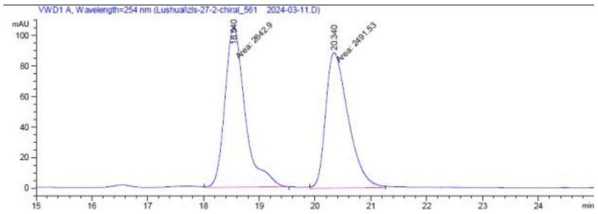

Peak #	RetTime [min]	Type	Width [min]	Area [mAU*s]	Height [mAU]	Area %
1	18.540	MM	0.4200	2642.89648	104.86731	51.4741
2	20.340	MM	0.4675	2491.52808	88.83176	48.5259

(*S*,*E*)-4-phenyl-2-(1-phenylvinyl)but-3-en-1-ol (48)

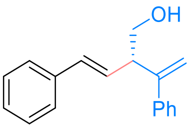

Prepared following general procedure **I**, with a reaction time of 18 h. Purification by flash column chromatography (Et_2O/hexane = 33:67, R_f = 0.2) gave the title compound (25.5 mg, 0.102 mmol, 51% yield, 5% ee).

NMR Spectroscopy (*see spectra*):

^{1}H NMR (400 MHz, CDCl$_3$): δ 7.45 – 7.37 (m, 4H), 7.36 – 7.29 (m, 4H), 7.26 – 7.20 (m, 1H), 6.59 (d, *J* = 15.9 Hz, 1H), 6.28 (dd, *J* = 16.0, 8.0 Hz, 1H), 5.46 (s, 1H), 5.23 (s, 1H), 3.85 (ddd, *J* = 10.6, 7.2, 5.8 Hz, 1H), 3.74 (ddd, *J* = 10.7, 7.1, 5.3 Hz, 1H), 3.69 – 3.61 (m, 1H), 1.65 (dd, *J* = 7.2, 5.4 Hz, 1H).

^{13}C NMR (101 MHz, CDCl$_3$): δ 148.5, 141.6, 136.9, 132.5, 129.3, 128.5, 128.4, 127.6, 127.5, 126.5, 126.3, 114.3, 64.8, 50.6.

HRMS (MS-EI) m/z calculated for $C_{18}H_{18}O$ [M]$^+$, 250.1352 found, 250.1349.

HPLC: Chiralpak IB column (250 mm), detected at 254 nm, hexane/i-propanol = 95/5, flow = 1.0 mL/min, retention time: 12.8 min (major), 15.4 min (minor).

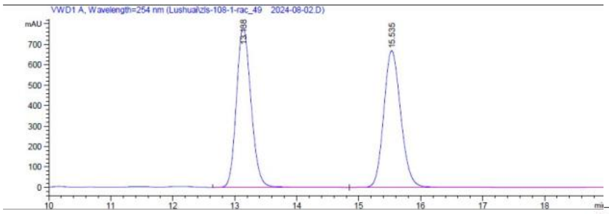

Peak #	RetTime [min]	Type	Width [min]	Area [mAU*s]	Height [mAU]	Area %
1	13.138	BV R	0.2551	1.28331e4	782.94104	49.9491
2	15.535	VV R	0.2965	1.28592e4	669.80042	50.0509

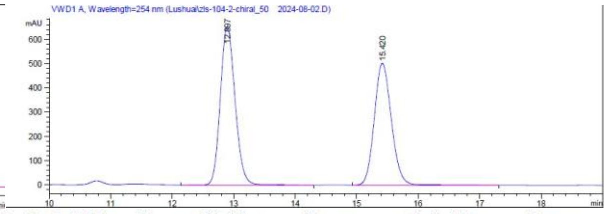

Peak #	RetTime [min]	Type	Width [min]	Area [mAU*s]	Height [mAU]	Area %
1	12.897	BV R	0.2588	1.09086e4	651.86658	52.7925
2	15.420	BV R	0.3003	9754.58301	501.64209	47.2075

2.7 Synthetic Applications

(2*S*,3*R*,4*S*)-2-([1,1'-biphenyl]-4-yl)-3-iodo-4-vinyltetrahydrofuran (49)

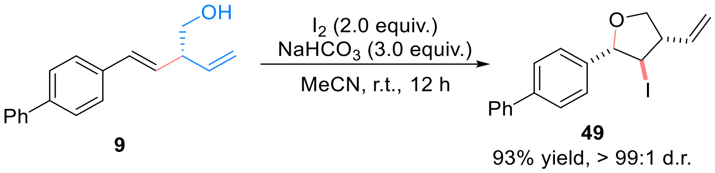

9 **49**

93% yield, > 99:1 d.r.

Procedure: An oven-dried flask was charged with compound **9** (25 mg, 0.10 mmol, 1.0 equiv.), iodine (50.8 mg, 0.200 mmol, 2.00 equiv.), sodium bicarbonate (25.2 mg, 0.300 mmol, 3.00 equiv.), and acetonitrile (2.0 mL). The mixture was stirred at room temperature for 12 hours. Upon completion of the reaction, the resulting solution was concentrated under reduced pressure and subsequently purified by column chromatography (hexane/ethyl acetate = 91:9 R_f = 0.45). This procedure afforded the target product **49** (34.9 mg, 0.093 mmol, 93%) as a white crystalline solid with a >99:1 diastereomeric ratio (d.r.).

NMR Spectroscopy (see spectra):

^{1}H NMR (400 MHz, CDCl$_3$): δ 7.61 (d, *J* = 8.3 Hz, 4H), 7.54 (d, *J* = 8.3 Hz, 2H), 7.45 (t, *J* = 7.5 Hz, 2H), 7.41 – 7.31 (m, 1H), 5.73 (ddd, *J* = 17.0, 10.2, 8.4 Hz, 1H), 5.37 – 5.24 (m, 2H), 5.11 (d, *J* = 9.3 Hz, 1H), 4.21 (t, *J* = 8.5 Hz, 1H), 3.92 (t, *J* = 8.6 Hz, 1H), 3.75 (t, *J* = 9.6 Hz, 1H), 3.38 – 3.24 (m, 1H).

^{13}C NMR (101 MHz, CDCl$_3$): δ 141.4, 140.8, 137.6, 134.7, 128.8, 127.5, 127.4, 127.2, 127.1, 118.9, 89.2, 71.9, 55.8, 34.3.

IR (neat): 2862, 1487, 1275, 1260, 1047, 918, 829, 749 cm^{-1}.

HRMS (EI-MS) m/z calculated for $C_{18}H_{17}IO$ [M]$^+$, 376.0319 found, 376.0314.

(*R*,*E*)-2-(2-([1,1'-biphenyl]-4-yl)vinyl)butane-1,4-diol (50)

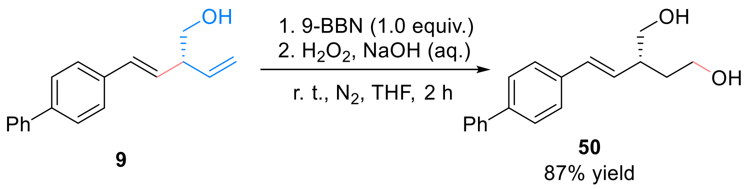

9 **50** 87% yield

Procedure: An oven-dried flask, compound **9** (25.0 mg, 0.10 mmol, 1.0 equiv.) was combined with 9-borabicyclo[3.3.1]nonane (9H-BBN) (0.5 M in THF, 1.0 equiv.) in anhydrous tetrahydrofuran. The reaction mixture was stirred at ambient temperature for 12 hours. Subsequently, an aqueous NaOH solution (3.0 M, 1.0 mL) and aq. H_2O_2 (30% wt, 0.1 mL) were sequentially added, and the resulting mixture was stirred for an additional 2 hours at room temperature. After confirmation of reaction completion by TLC analysis, the mixture was extracted with dichloromethane (3 × 3.0 mL). The combined organic layers were dried over anhydrous $MgSO_4$, filtered, and concentrated in vacuo. Purification of the crude material by flash column chromatography (hexane/ethyl acetate = 50:50, R_f = 0.35) afforded the desired product **50** as a white crystalline solid (23.3 mg, 0.087 mmol, 87%).

NMR Spectroscopy (*see spectra*):

^{1}H NMR (400 MHz, DMSO-*D*$_6$): δ 7.66 (d, *J* = 7.0 Hz, 2H), 7.62 (d, *J* = 8.4 Hz, 2H), 7.50 – 7.41 (m, 4H), 7.34 (t, *J* = 7.3 Hz, 1H), 6.43 (d, *J* = 15.9 Hz, 1H), 6.19 (dd, *J* = 16.0, 8.5 Hz, 1H), 3.62 – 3.44 (m, 4H), 2.41 (td, *J* = 8.9, 4.7 Hz, 1H), 1.78 – 1.63 (m, 2H).

^{13}C NMR (101 MHz, DMSO-*D*$_6$): δ 140.2, 139.0, 137.0, 133.6, 129.9, 129.4, 127.8, 127.2, 126.9, 126.9, 65.3, 59.4, 42.8, 34.7.

IR (neat): 3341, 2927, 2857, 1275, 1260, 1006, 749 cm^{-1}.

HRMS (EI-MS) m/z calculated for $C_{18}H_{20}O_2$ [M]$^+$, 268.1463 found, 268.1462.

(*R*,*E*)-5-(2-([1,1'-biphenyl]-4-yl)vinyl)tetrahydro-2H-pyran-2-one (51)

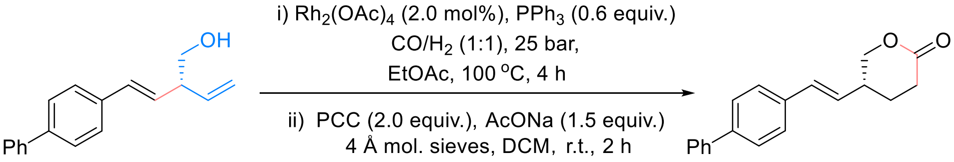

9 **51** 76% yield (2 steps)

Procedure: In the main reactor of the autoclave, $Rh_2(OAc)_4$ (1.4 mg, 0.004 mmol, 2.0 mol%) and PPh_3 (31.4 mg, 0.12 mmol, 0.6 equiv.) were dissolved in anhydrous ethyl acetate (5.0 mL) and subjected to CO/H_2 (1:1, 10 bar) at 100 °C for 10 min. Then, a solution of compound **9** (50.0 mg, 0.2 mmol, 1.0 equiv.) in anhydrous ethyl acetate (2.0 mL) was added to the secondary autoclave. The system was evacuated three times with CO/H_2, and the pressure was maintained at 20 bar. The substrate was pushed into the autoclave under pressure, and the secondary valve was closed. The reaction mixture was stirred at 100 °C for 4 hours. After completion, the mixture was allowed to cool to room temperature. The reaction solution was concentrated and subjected to column chromatography (hexane/ethyl acetate = 75:25, R_f = 0.2) to afford the hemiacetal intermediate (45.6 mg, 0.163 mmol, 81% yield). Next, the hemiacetal intermediate (28.0 mg, 0.1 mmol, 1.0 equiv.), PCC (43.1 mg, 0.20 mmol, 2.0 equiv.), AcONa (12.3 mg, 0.15 mmol, 1.5 equiv.), and 4 Å molecular sieves (20 mg) were combined and stirred at room temperature for 2 hours. Upon completion of the reaction, the solution was concentrated and subjected to column chromatography (hexane/ethyl acetate = 75:25, R_f = 0.2) to obtain **51** (26.1 mg, 0.090 mmol, 94%).

NMR Spectroscopy (*see spectra*):

¹H NMR (400 MHz, $CDCl_3$): δ 7.61 – 7.55 (m, 4H), 7.49 – 7.40 (m, 4H), 7.40 – 7.29 (m, 1H), 6.58 (d, *J* = 15.9 Hz, 1H), 6.10 (dd, *J* = 16.0, 7.4 Hz, 1H), 4.44 (ddd, *J* = 11.2, 4.7, 1.9 Hz, 1H), 4.19 (dd, *J* = 11.2, 9.8 Hz, 1H), 2.90 – 2.79 (m, 1H), 2.74 (ddd, *J* = 18.0, 6.9, 4.7 Hz, 1H), 2.60 (ddd, *J* = 18.0, 9.6, 7.2 Hz, 1H), 2.22 – 2.09 (m, 1H), 1.97 – 1.83 (m, 1H).

¹³C NMR (101 MHz, $CDCl_3$): δ 170.4, 140.5, 140.3, 135.2, 131.8, 128.6, 127.2, 127.1, 126.9, 126.7, 126.4, 72.5, 36.4, 28.7, 25.6.

IR (neat): 2989, 1462, 1275, 1260, 750 cm^{-1}.

HRMS (ESI) m/z calculated for $C_{19}H_{19}O_2$ $[M+H]^+$, 279.1380 found, 279.1379.

(*R*,*E*)-4-([1,1'-biphenyl]-4-yl)-2-((*E*)-2-(4,4,5,5-tetramethyl-1,3,2-dioxaborolan-2-yl)vinyl)but-3-en-1-ol (52)

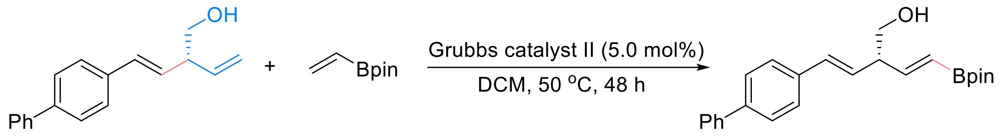

Procedure: In a dry reaction flask, compound **9** (0.1 mmol, 25.0 mg), 4,4,5,5-tetramethyl-2-vinyl-1,3,2-dioxaborolane (61.6 mg, 0.40 mmol, 4.0 equiv.), and Grubbs catalyst (**II**) (4.3 mg, 0.005 mmol, 5.0 mol%) were dissolved in anhydrous DCM (2.0 mL). The mixture was then purged with nitrogen and stirred at 50 °C for 48 hours. Upon completion of the reaction, the solution was concentrated and subjected to column chromatography (hexane/ethyl acetate = 83:17, R_f = 0.4) to yield **52** (36.1 mg, 0.096 mmol, 96%).

NMR Spectroscopy (<u>*see spectra*</u>):

¹H NMR (400 MHz, $CDCl_3$): δ 7.63 – 7.48 (m, 4H), 7.46 – 7.41 (m, 4H), 7.37 – 7.31 (m, 1H), 6.76 – 6.46 (m, 2H), 6.17 (dd, *J* = 15.9, 8.2 Hz, 1H), 5.65 (dd, *J* = 18.2, 1.3 Hz, 1H), 3.71 (qd, *J* = 10.7, 6.8 Hz, 2H), 3.30 – 3.19 (m, 1H), 1.28 (s, 12H).

¹³C NMR (101 MHz, $CDCl_3$): δ 151.7, 140.7, 140.3, 136.0, 132.4, 128.8, 128.1, 127.3, 127.3, 127.0, 126.7, 83.4, 65.0, 52.0, 24.8.

IR (neat): 3447, 3003, 2979, 2867, 1736, 1634, 1485, 1275, 1260, 1142, 967, 848, 750 cm^{-1}.

HRMS (MS-ES) m/z calculated for $C_{24}H_{29}BNaO_3$ $[M+Na]^+$, 399.2107 found, 399.2106.

Table S8. Optimization of the synthesis of 54 [a]

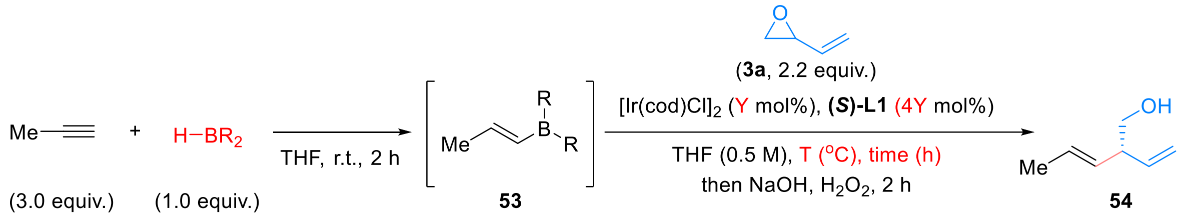

Entry	H−BR$_2$	T (°C)	time (h)	Y	Yield of **54** (%)	ee (%)
1	9-BBN	0	5	1.0 mol%	11	78
2	Cy_2BH	0	2	1.0 mol%	40	90
3	Cy_2BH	0	4	1.0 mol%	45	90
4	Cy_2BH	0	5	1.0 mol%	50	90
5	Cy_2BH	0	8	1.0 mol%	50	90
6	Cy_2BH	0	5	10 mol%	46	-
7	Cy_2BH	20	5	0.1 mol%	58	92
8	Cy_2BH	20	5	0.1 mol%	63[b]	92

[a] Reaction conditions: 9H-BBN (0.5 M in THF, 1.0 mmol, 1.0 equiv.) or Cy_2BH (1.0 mmol, 1.0 equiv.), propyne (1.0 M in THF, 3.0 equiv.), $[Ir(cod)Cl]_2$ (Y mol%), **(S)-L1** (4Y mol%), **3a** (2.2 equiv.), THF (0.5 M) for the time and temperature stated. Work up with aq. NaOH (3.0 M, 1.0 mL), aq. H_2O_2 (30% wt, 0.3 mL), 2 h. NMR yield with CH_2Br_2 as internal standard, ee determined by chiral HPLC.
[b] 10.0 mmol scale, isolated yield determined after Kugelrohr distillation.

A study was performed to establish the optimal reaction conditions for the allylation of propyne to access **54** (Table S8). Initial experiments employing 9H-BBN as the hydroboration reagent at 0 °C for 5 h resulted in a modest yield of 11% with 78% ee (entry 1). Switching to Cy_2BH significantly improved both the reaction efficiency and stereoselectivity, affording product **54** in 40% yield with 90% ee (entry 2). Further optimization of reaction parameters revealed that the reaction time played a crucial role in this transformation. When the reaction time was extended from 2 h to 4 h at 0 °C, the yield increased to 45% while maintaining excellent enantioselectivity (90% ee, entry 3). A slight extension of the reaction time to 5 h further improved the yield to 50% (entry 4). Interestingly, extending the reaction time to 8 h did not provide additional improvement in yield (entry 5), suggesting that the reaction had reached completion. We next examined the influence of catalyst loading on the reaction outcome. Increasing the catalyst loading from 1.0 mol% to 10 mol% resulted in a slight decrease in yield to 46% (entry 6), indicating that higher catalyst loadings may promote undesired side reactions. Remarkably, reducing the catalyst loading to 0.1 mol% while simultaneously increasing the reaction temperature to 20 °C and extending the reaction time to 5 h, led to a significant improvement in yield and enantioselectivity (58%, 92% ee, entry 7). To verify the reproducibility of these promising results, we conducted two additional experiments under identical conditions (entries 8 and 9). The consistent yields (58% and 63%, respectively) and excellent enantioselectivities (92% ee) confirmed the robustness of our optimized protocol.

Procedure for optimised conditions: In an argon-filled glovebox, Cy_2BH (1.78 g, 10.0 mmol, 1.0 equiv.) was added to an oven-dried scintillation vial and sealed with a rubber septum. The sealed vial was then transferred out of the glovebox, and a solution of propyne (30.0 mL, 0.5 M in THF, 3.0 equiv.) was rapidly introduced via syringe. The reaction mixture was stirred at room temperature for 2 h, after which time the excess propyne and THF were removed under reduced pressure. Concurrently, in a separate 10 mL oven-dried flask inside the glovebox, was added $[Ir(COD)Cl]_2$ (6.5 mg, 0.1 mmol, 0.1 mol%), **(*S*)-L1** (20.0 mg, 0.4 mmol, 0.4 mol%), and vinyl

epoxide **3a** (1.75 mL, 22 mmol, 2.2 equiv.) and sealed with a rubber septum. The in situ generated borane solution was then transferred into the flask containing the catalyst and ligand, rinsing with THF (15 mL) to ensure complete transfer. The resulting mixture was stirred at 20 °C for 5 h. Upon completion of the reaction, the mixture was treated with aq. NaOH (3.0 M, 10 mL) and aq. H_2O_2 (30% wt, 3.0 mL). After vigorous stirring for 2 h at room temperature, the excess H_2O_2 was quenched with sat. aq. $Na_2S_2O_3$ (10 mL). The product was extracted with dichloromethane (3 × 10 mL), and the combined organic layers were dried over anhydrous $MgSO_4$, filtered, and concentrated under reduced pressure. The crude product was purified by Kugelrohr distillation (55 °C, 0.1 Torr, collection flask at −78 °C) to yield **54** (0.705 g, 6.30 mmol, 63% yield, 92% ee).

(*R*,*E*)-2-vinylpent-3-en-1-ol (54)

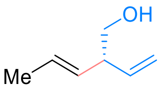

NMR Spectroscopy (<u>*see spectra*</u>):

^{1}H NMR (400 MHz, $CDCl_3$): δ 5.80 – 5.65 (m, 1H), 5.58 (dqd, *J* = 15.3, 6.4, 1.0 Hz, 1H), 5.34 (ddq, *J* = 15.3, 7.7, 1.6 Hz, 1H), 5.14 (q, *J* = 1.5 Hz, 1H), 5.14 – 5.06 (m, 1H), 3.57 – 3.44 (m, 2H), 2.96 – 2.83 (m, 1H), 1.70 (1.70 (dd, *J* = 6.4, 0.9 Hz, 3H), 1.61 (t, *J* = 6.2 Hz, 1H).

^{13}C NMR (101 MHz, $CDCl_3$): δ 138.0, 129.8, 128.2, 116.6, 65.2, 49.9, 18.2.

HRMS (MS-ES) m/z calculated for $C_7H_{13}O$ $[M+H]^+$, 113.0961 found, 113.0960.

Specific rotation: $[\alpha]_D^{20.5}$ −4.0 (c = 2.0 M, CH_2Cl_2).

IR (neat): 3364, 2989, 2931, 2856, 1453, 1275, 1260, 749 cm^{-1}.

HPLC: Chiralpak AD-H column (250 mm), detected at 210 nm, hexane/i-propanol = 98/2, flow = 1.0 mL/min, retention time: 10.2 min (major), 10.7 min (minor).

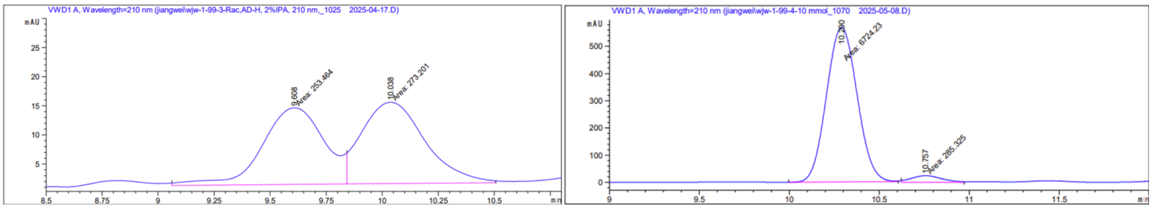

Peak #	RetTime [min]	Type	Width [min]	Area [mAU*s]	Height [mAU]	Area %
1	9.608	MF	0.3211	253.46410	13.15590	48.1263
2	10.038	FM	0.3260	273.20074	13.96642	51.8737

Peak #	RetTime [min]	Type	Width [min]	Area [mAU*s]	Height [mAU]	Area %
1	10.290	MM	0.1970	6724.22998	568.85358	95.9295
2	10.757	MM	0.1948	285.32474	24.41019	4.0705

2-hydroxy-6-((S,1E,4E)-3-(hydroxymethyl)hexa-1,4-dien-1-yl)benzaldehyde (56)

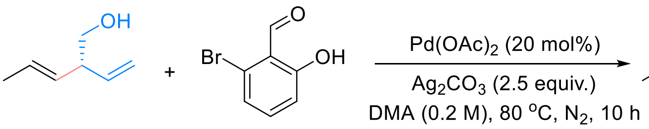

54 **55** **56**

Procedure: In an oven-dried flask, $Pd(OAc)_2$ (8.8 mg, 0.04 mmol, 20 mol%), Ag_2CO_3 (138 mg, 0.50 mmol, 2.5 equiv.), and 2-bromo-6-hydroxybenzaldehyde **55** (40.2 mg, 0.20 mmol) were combined in anhydrous DMA (1.0 mL). The mixture was stirred under vacuum and exchanged with nitrogen to remove air. Then, (*R*,*E*)-2-vinylpent-3-en-1-ol **54** (33.6 mg, 0.3 mmol, 1.5 equiv.) was carefully added by micro-syringe. The reaction mixture was stirred at 80 °C for 10 hours. After confirmation of reaction completion by TLC analysis, the mixture was extracted with

dichloromethane (3 × 5.0 mL) and H_2O (5.0 mL). The combined organic layers were dried over anhydrous $MgSO_4$, filtered, and concentrated in vacuo. Purification of the crude material by flash column chromatography (hexane/ethyl acetate/toluene = 70:15:15, R_f = 0.20) afforded the desired product **56** as a colourless oil (25.6 mg, 0.112 mmol, 56%, 90% ee). (*R*,*E*)-2-vinylpent-3-en-1-ol **54** was simultaneously recovered (4.2 mg, 0.0375 mmol, 13%).

NMR Spectroscopy (<u>*see spectra*</u>):

^{1}H NMR (500 MHz, $CDCl_3$): δ 11.86 (s, 1H), 10.31 (s, 1H), 7.43 (t, *J* = 8.0 Hz, 1H), 6.99 – 6.89 (m, 2H), 6.86 (d, *J* = 8.3 Hz, 1H), 6.04 (dd, *J* = 15.7, 7.4 Hz, 1H), 5.67 (ddd, *J* = 15.3, 6.5, 1.0 Hz, 1H), 5.48 – 5.39 (m, 1H), 3.70 – 3.60 (m, 2H), 3.14 (p, *J* = 7.2 Hz, 1H), 1.75 (d, *J* = 6.4 Hz, 3H).

^{13}C NMR (126 MHz, $CDCl_3$): δ 195.5, 162.9, 143.0, 137.6, 137.2, 129.3, 129.2, 126.2, 118.9, 117.4, 116.9, 65.5, 49.4, 18.3.

Specific rotation: $[\alpha]_D^{19.5}$ −20.0 (c = 0.5 M, CH_2Cl_2) {lit.[9] $[\alpha]_D^{24}$ −22.2 (c = 0.105, $CHCl_3$)}.

IR (neat): 3391, 3005, 2989, 1643, 1452, 1275, 1260, 750 cm^{-1}.

HRMS (MS-ES) m/z calculated for $C_{14}H_{16}NaO_3$ $[M+Na]^+$, 255.0997 found, 255.0994.

HPLC: Chiralpak AD-H column (250 mm), detected at 254 nm, hexane/i-propanol = 95/5, flow = 1.0 mL/min, retention time: 21.5 min (major), 25.7 min (minor).

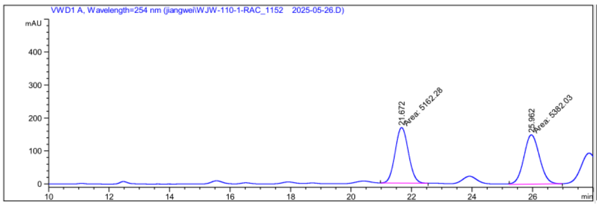

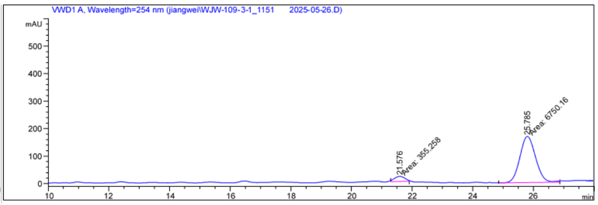

Peak #	RetTime [min]	Type	Width [min]	Area [mAU*s]	Height [mAU]	Area %
1	21.672	MM	0.5107	5162.28320	168.45961	48.9580
2	25.962	MM	0.6005	5382.03027	149.37050	51.0420

Peak #	RetTime [min]	Type	Width [min]	Area [mAU*s]	Height [mAU]	Area %
1	21.576	MM	0.3672	355.25839	16.12598	4.9998
2	25.785	MM	0.6489	6750.16357	173.36189	95.0002

3. REFERENCES

1. (a) Q. Zhang, H. M. Nguyen, *Chem. Sci.*, **2014**, 5, 291-296; (b) S. Pandit, A. S. Adhikari, N. Majumdar, *Org. Lett*. **2022**, *24*, 7388-7393.

2. J. R. Jagannathan, Y. Ma, B. J. Curole, S. M. Grayson, O. S. Fenton, F. A. Leibfarth, *J. Am. Chem. Soc.* **2024**, *146*, 15264-15274.

3. L. Hunter, D. O'Hagan, A. M. Z. Slawin, *J. Am. Chem. Soc.* **2006**, *128*, 16422-16423.

4. F. Pünnerand, G. Hilt, *Chem. Commun*. **2012**, 48, 3617–3619

5. S. Faßbender, J. Molloy, C. Mück-Lichtenfeld, R. Gilmour, *Angew. Chem. Int. Ed.* **2019**, 58, 18619 – 18626

6. G. Y. Fang, O. A. Wallner; N. Di Blasio; X. Ginesta, J. N. Harvey, V. K. Aggarwal, *J. Am. Chem. Soc*. **2007**, *129*, 14632-14639.

7. Y. Cheng, C.K. Klein, I. A. Tonks, *Chem. Sci.*, **2020**, *11*, 10236-10242.

8. V. Chintalapudi, E. A. Galvin, R. L. Greenaway, E. A. Anderson, *Chem. Comm.* **2016**, 693-696.

9. K. Tanaka, Y. Nakamura, A. Sasaki, R. Ueda, Y. Suzuki, S. Kuwahara, H. Kiyota, *Tetrahedron* **2009**, *65*, 6115-6122.

10. (a) C. Lee, W. Yang, R. G. Parr, *Phys. Rev. B* **1988**, *37*, 785–789; (b) A. D. Becke, *J. Chem. Phys.* **1993**, *98*, 5648-5652.

11. P. J. Hay, W. R. Wadt, *J. Chem. Phys.*, **1985**, *82*, 270-283.

12. (a) W. J. Hehre, R. Ditchfield, J. A. Pople, *J. Chem. Phys.* **1972**, *56*, 2257-2261; (b) P. C. Hariharan, J. A. Pople, *Theor. Chim. Acta.* **1973**, *28*, 213-222; (c) R. Krishnan, J. S. Binkley, R. Seeger, J. A. Pople, *J. Chem. Phys.* **1980**, *72*, 650-654; (d) A. D. McLean, G. S. Chandler, *J. Chem. Phys.* **1980**, *72*, 5639-5648; (e) M. M. Francl, W. J. Pietro, W. J. Hehre, J. S. Binkley, M. S. Gordon, D. J. DeFrees, J. A., Pople, *J. Chem. Phys.* **1982**, *77*, 3654-3665.

13. Y. Zhao, D. G. Truhlar, *Theor. Chem. Acc.* **2008**, *120*, 215-241.

14. L. E. Roy, P. J. Hay, R. L. Martin, *J. Chem. Theory Comput.* **2008**, *4*, 1029-1031.

15. A. V. Marenich, C. J. Cramer, D. G. Truhlar, *J. Phys. Chem. B* **2009**, *113*, 6378-6396.

16. C. R. Davis, Y. Fu, P. Liu, J. M. Ready, *J. Am. Chem. Soc.* **2022**, *144*, 16118-16130.

17. K. Fukui, *Acc. Chem. Res.* **1981**, *14*, 363-368.

18. Gaussian 16, Revision C.01, M. J. Frisch, G. W. Trucks, H. B. Schlegel, G. E. Scuseria, M. A. Robb, J. R. Cheeseman, G. Scalmani, V. Barone, G. A. Petersson, H. Nakatsuji, X. Li, M. Caricato, A. V. Marenich, J. Bloino, B. G. Janesko, R. Gomperts, B. Mennucci, H. P. Hratchian, J. V. Ortiz, A. F. Izmaylov, J. L. Sonnenberg, D. Williams-Young, F. Ding, F. Lipparini, F. Egidi, J. Goings, B. Peng, A. Petrone, T. Henderson, D. Ranasinghe, V. G. Zakrzewski, J. Gao, N. Rega, G. Zheng, W. Liang, M. Hada, M. Ehara, K. Toyota, R. Fukuda, J. Hasegawa, M. Ishida, T. Nakajima, Y. Honda, O. Kitao, H. Nakai, T. Vreven, K. Throssell, J. A. Jr.

Montgomery, J. E. Peralta, F. Ogliaro, M. J. Bearpark, J. J. Heyd, E. N. Brothers, K. N. Kudin, V. N. Staroverov, T. A. Keith, R. Kobayashi, J. Normand, K. Raghavachari, A. P. Rendell, J. C. Burant, S. S. Iyengar, J. Tomasi, M. Cossi, J. M. Millam, M. Klene, C. Adamo, R. Cammi, J. W. Ochterski, R. L. Martin, K. Morokuma, O. Farkas, J. B. Foresman, D. J. Fox, Gaussian, Inc., Wallingford CT, **2016**.

19. AQME, v1.4, J. V. Alegre-Requena, S. Sowndarya, T. Alturaifi, R. Perez-Soto, R. S. Paton, WIREs *Comput. Mol. Sci.* **2023**,17590884.

20. CYLview, 1.0b; Legault, C. Y., Université de Sherbrooke, **2009** (http://www.cylview.org).

21. S. Grimme, *Chem. Eur. J.* **2012**, *18*, 9955-9964.

22. G. Luchini, J. V. Alegre-Requena, I. Funes-Ardoiz, R. S. Paton, *F1000Research* **2020**, *9*, 291.

23. V. S. Bryantsev, M. S. Diallo, W. A. Goddard III, *J. Phys. Chem. B* **2008**, *112*, 9709-9719.

4. NMR SPECTRA

^{1}H NMR (400 MHz, $CDCl_3$) of **3b** (*see procedure*)

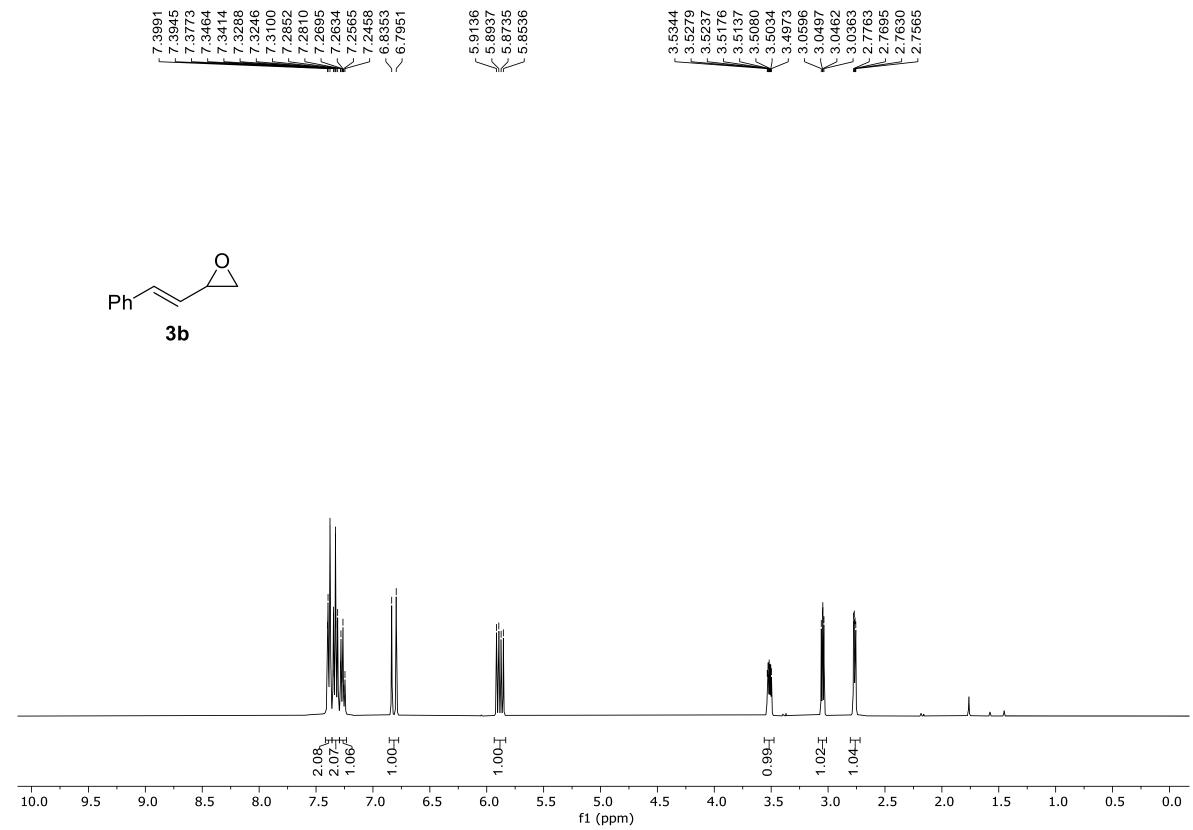

^{13}C NMR (101 MHz, $CDCl_3$) of **3b**

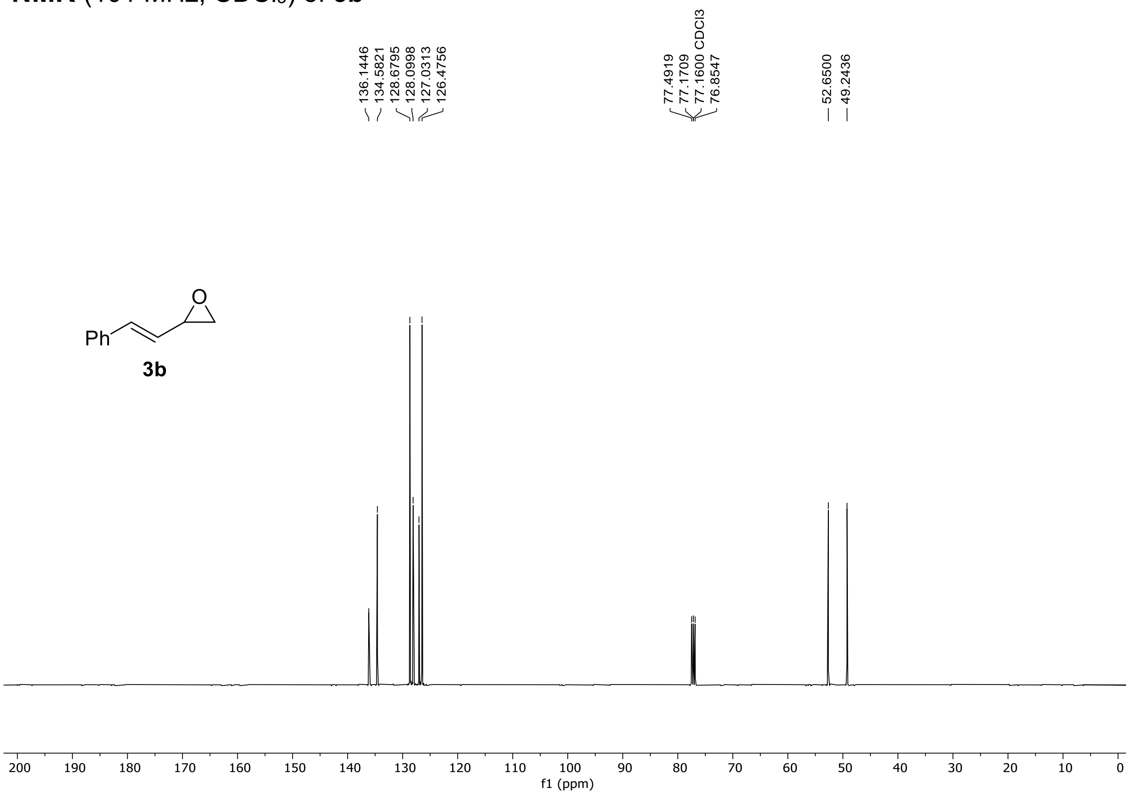

^{1}H NMR (400 MHz, $CDCl_3$) of **3c** (*see procedure*)

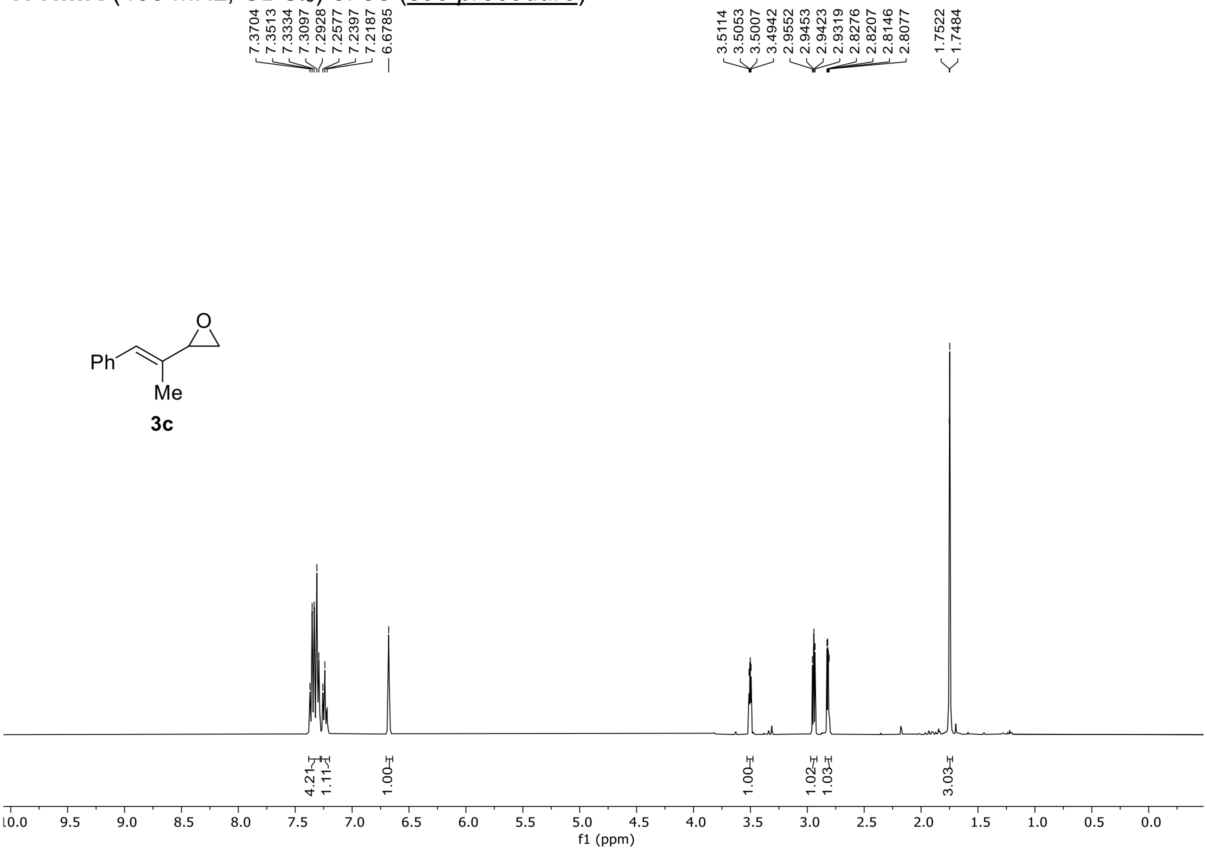

^{13}C NMR (101 MHz, $CDCl_3$) of **3c**

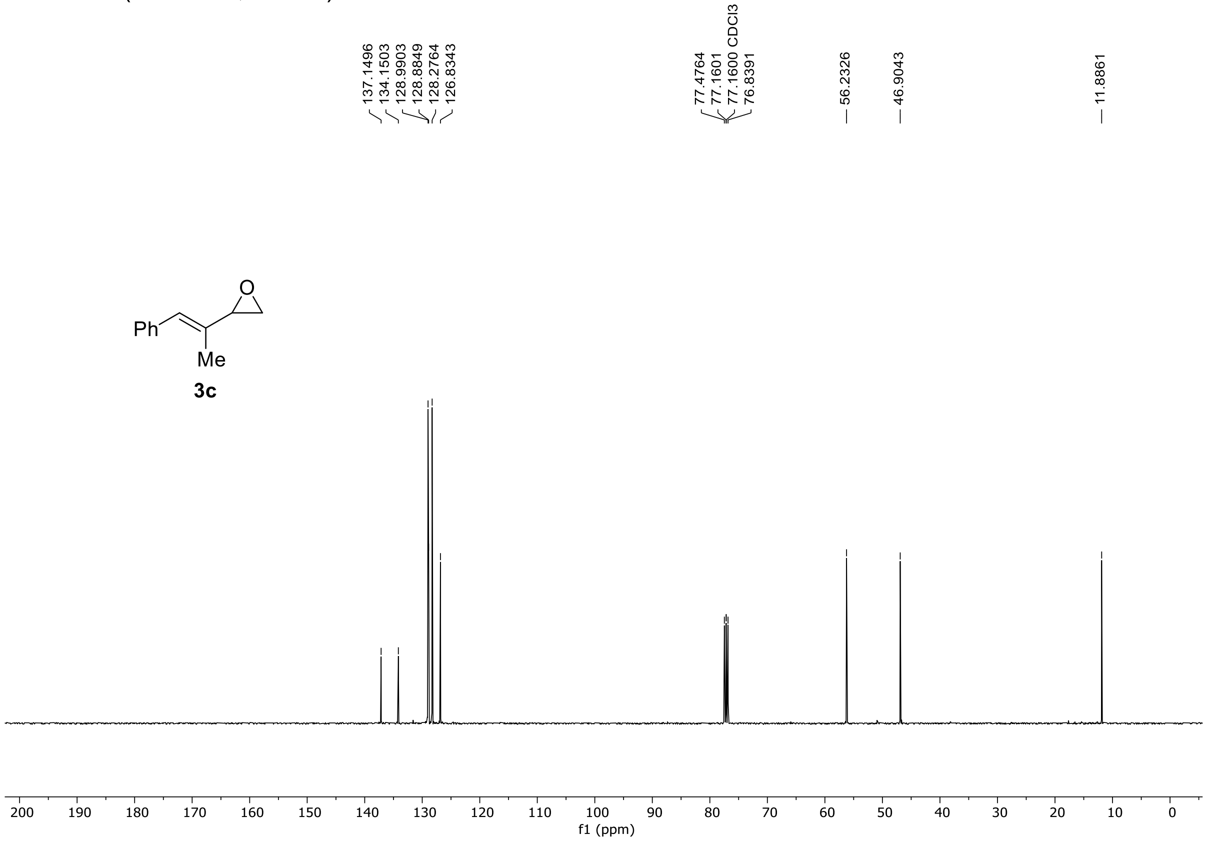

^{1}H NMR (400 MHz, $CDCl_3$) of **3d** (*see procedure*)

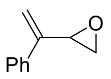

3d

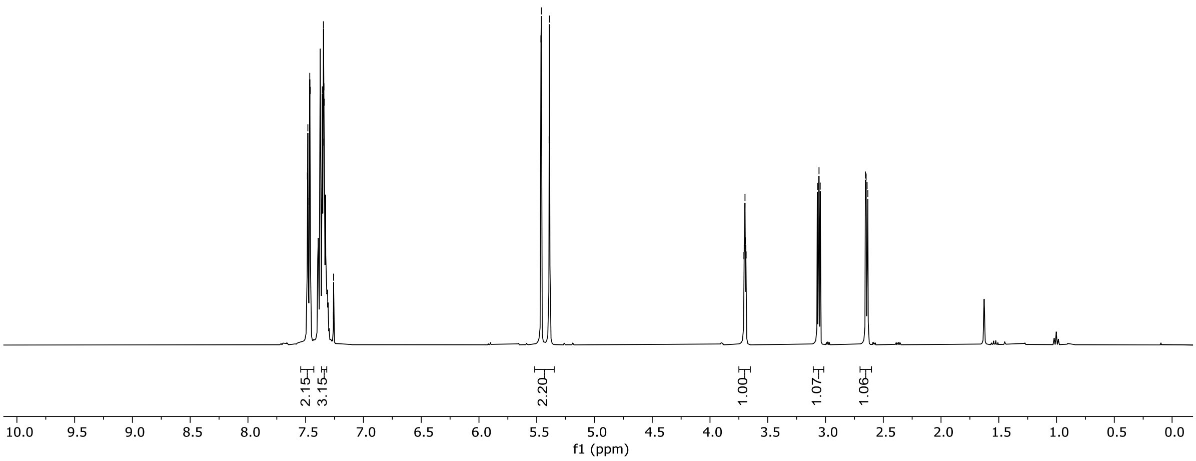

^{13}C NMR (101 MHz, $CDCl_3$) of **3d**

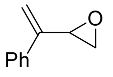

3d

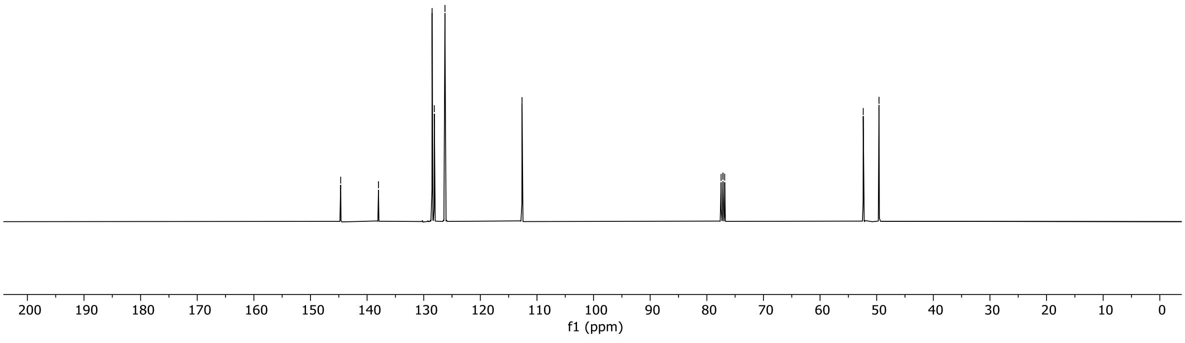

^{1}H NMR (400MHz, $CDCl_3$) of **3e** (*see procedure*)

3e

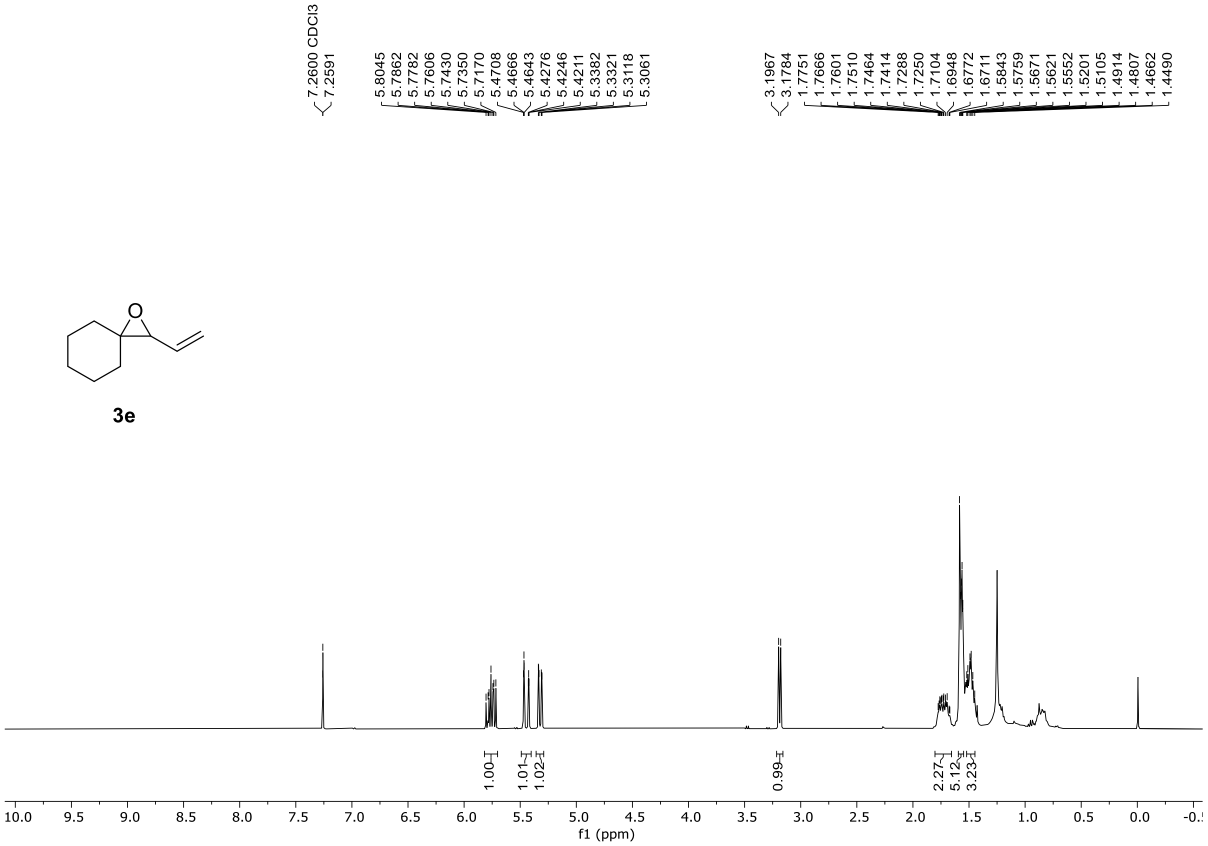

^{13}C NMR (101 MHz, $CDCl_3$) of **3e**

3e

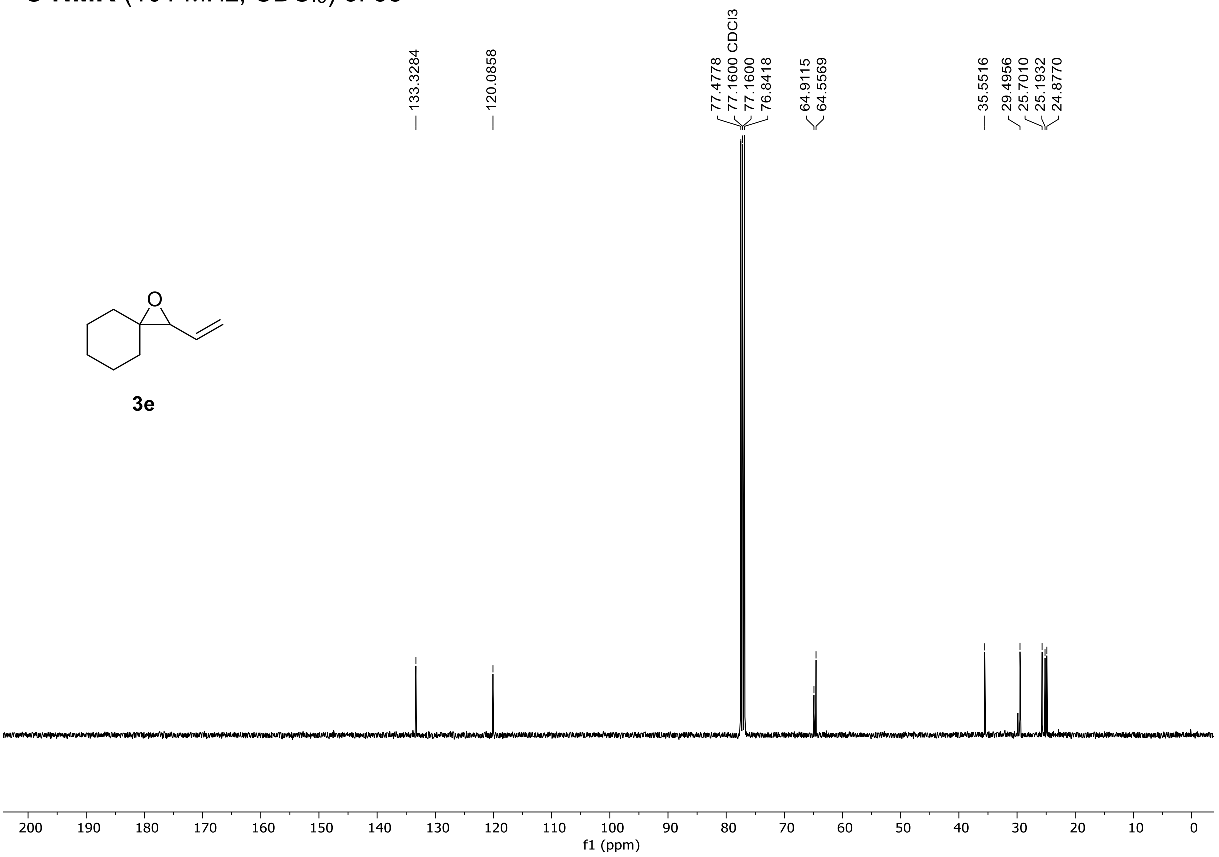

^{1}H NMR (400 MHz, $CDCl_3$) of **3f** (*see procedure*)

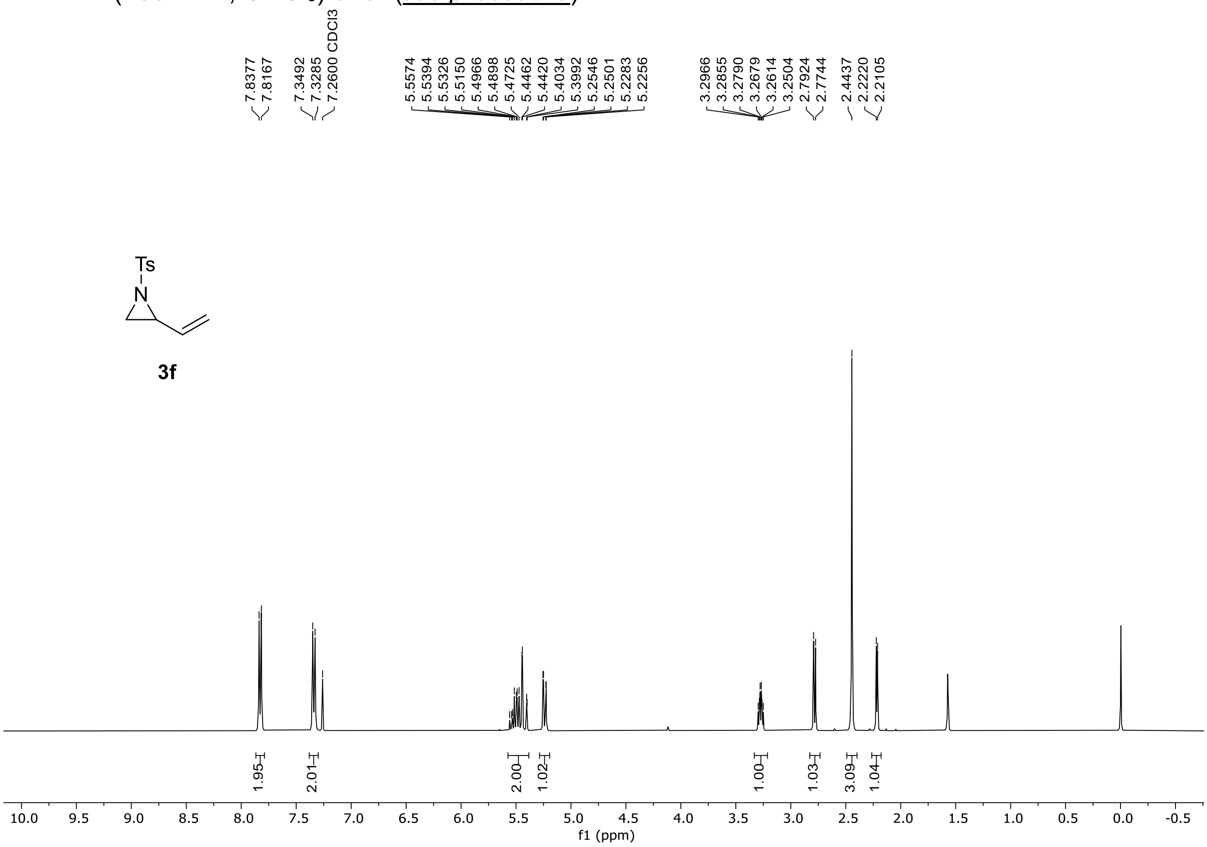

^{13}C NMR (101 MHz, $CDCl_3$) of **3f**

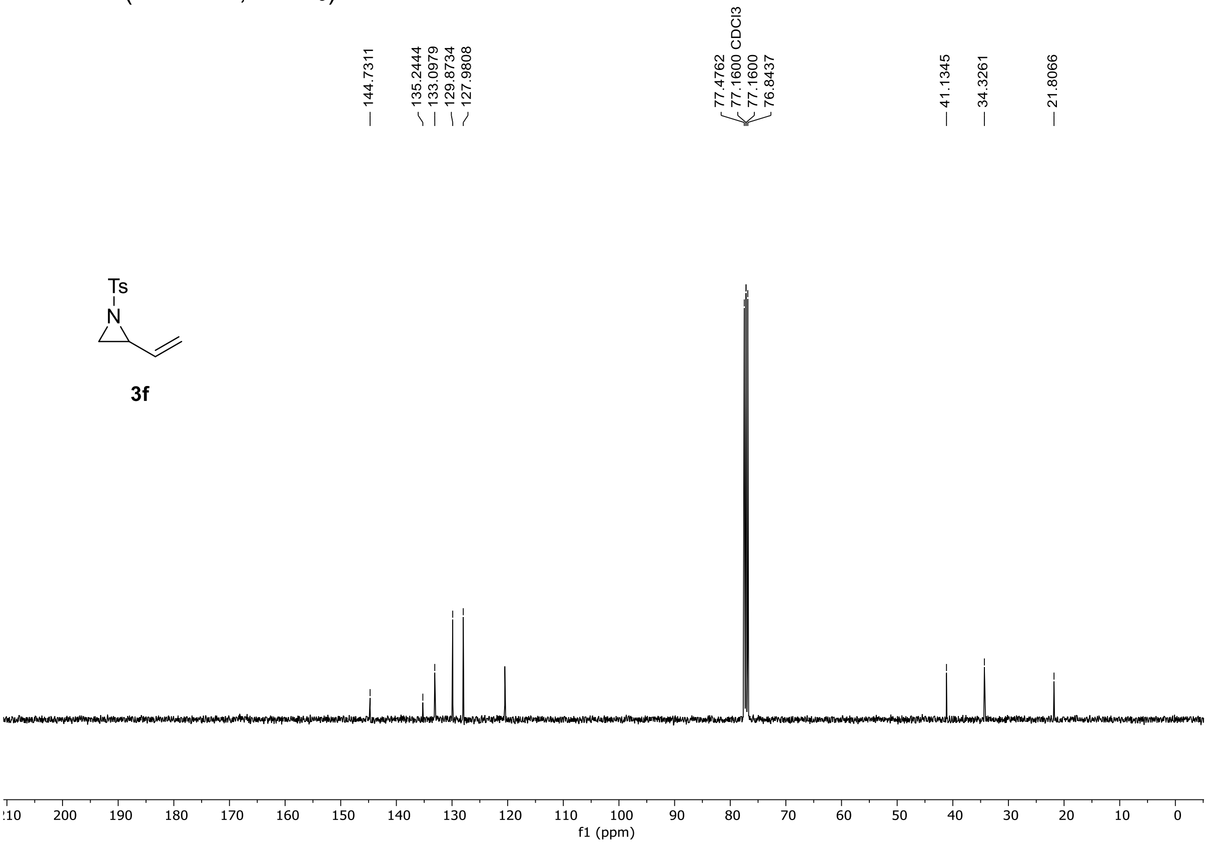

^{1}H NMR (400 MHz, $CDCl_3$) of **1-chloro-4-ethynylbenzene** (*see procedure*)

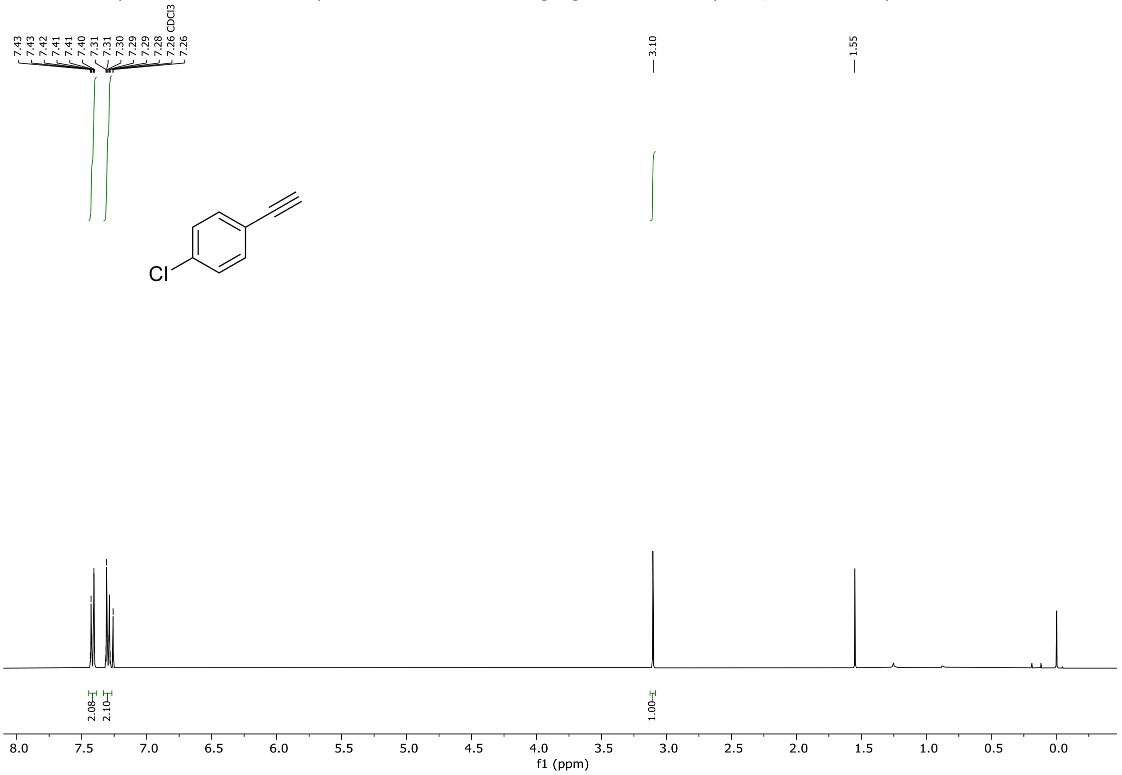

^{13}C NMR (101 MHz, $CDCl_3$) of **1-chloro-4-ethynylbenzene**

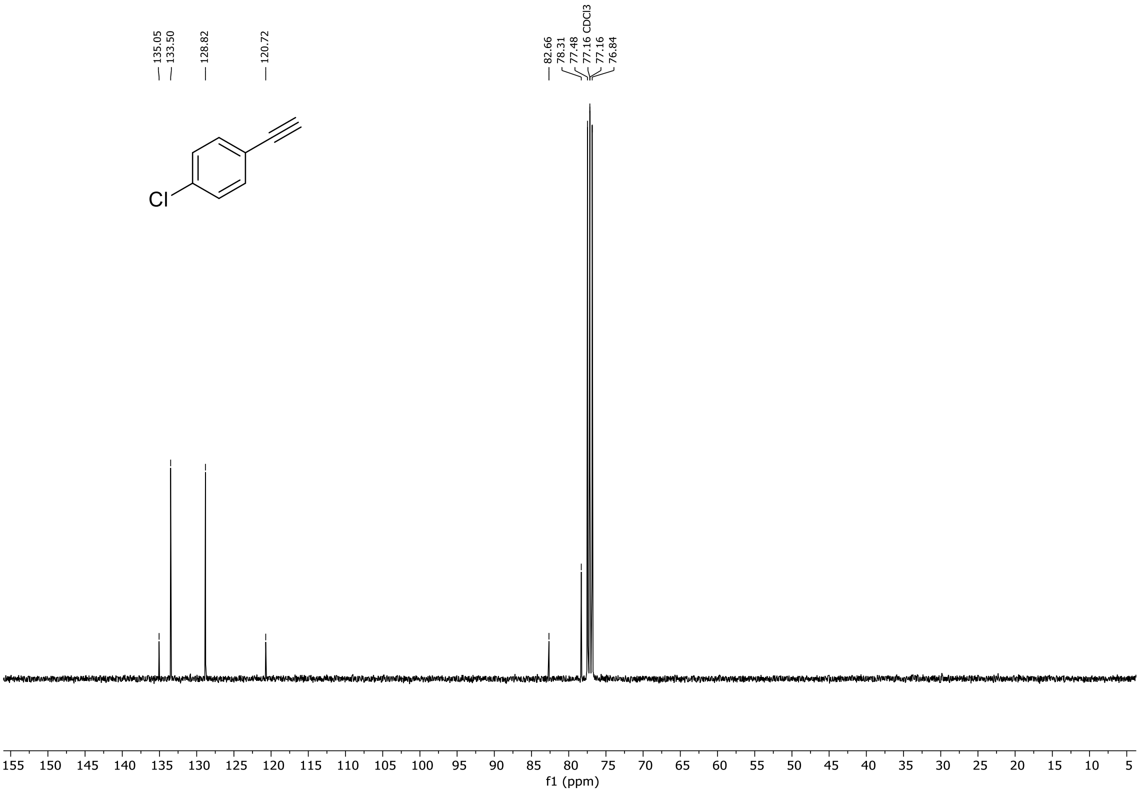

^{1}H NMR (400 MHz, $CDCl_3$) of **5-ethynyl-2,3-dihydrobenzofuran** (*see procedure*)

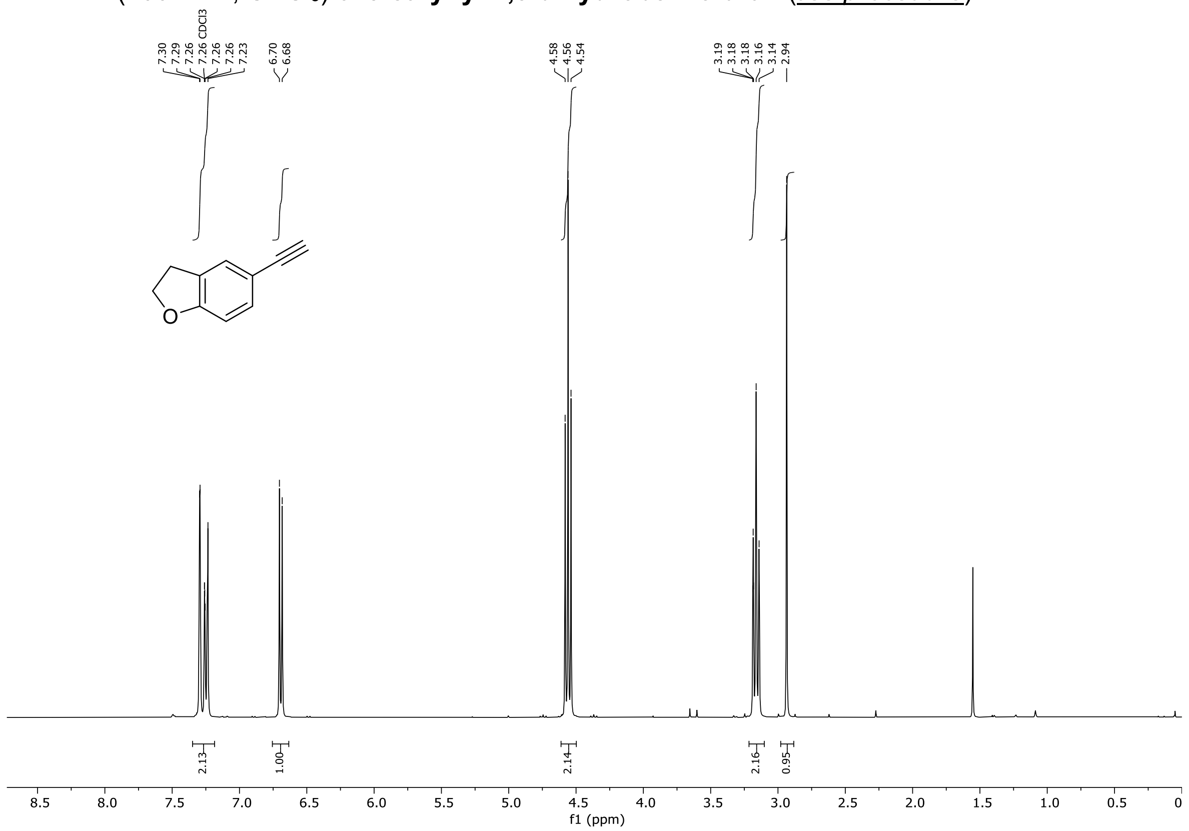

^{13}C NMR (126 MHz, $CDCl_3$) of **5-ethynyl-2,3-dihydrobenzofuran**

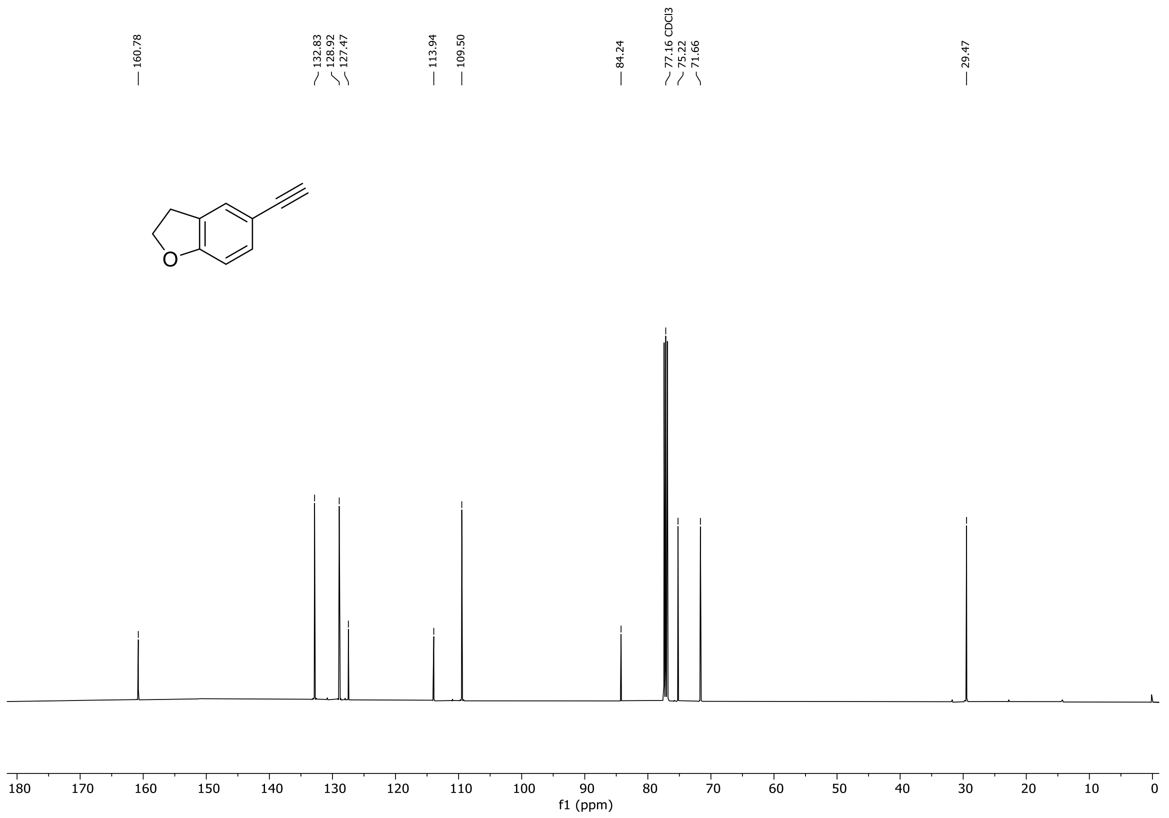

^{1}H NMR (400 MHz, $CDCl_3$) of **benzyl(ethynyl)dimethylsilane** (*see procedure*)

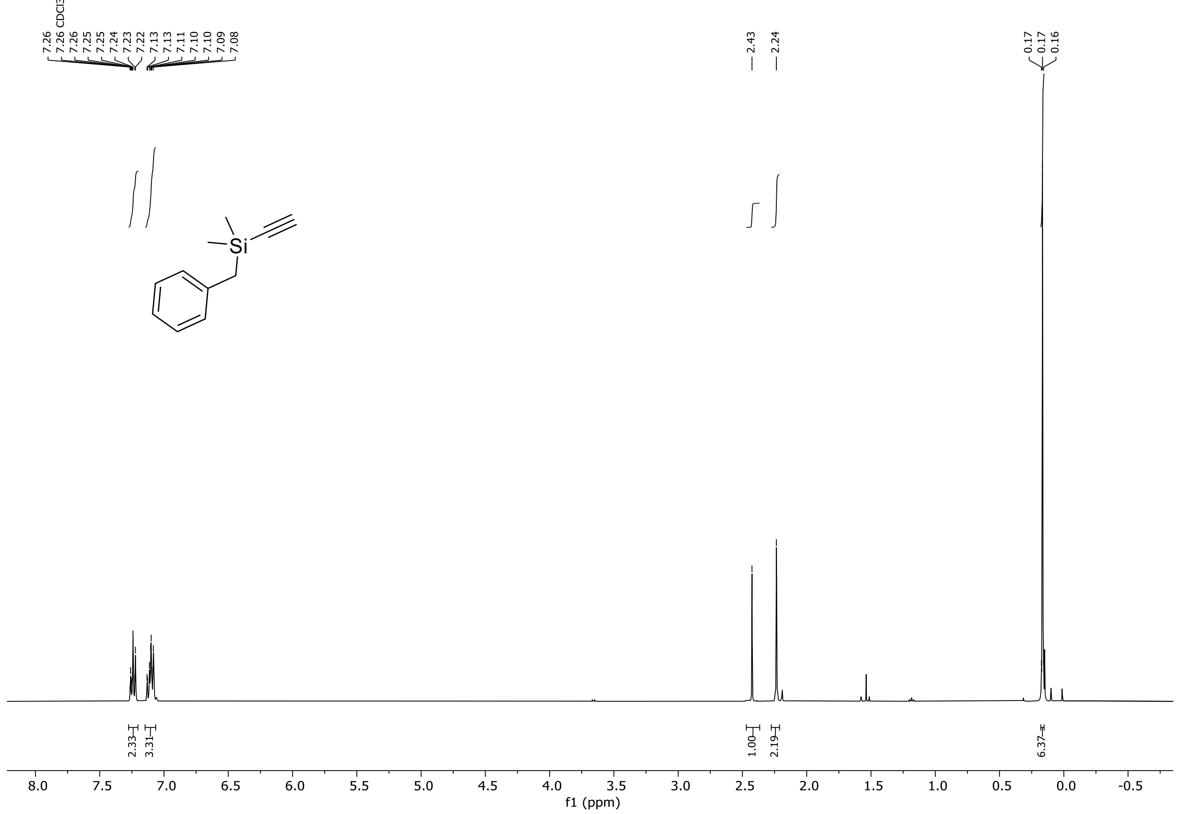

^{13}C NMR (126 MHz, $CDCl_3$) of **benzyl(ethynyl)dimethylsilane**

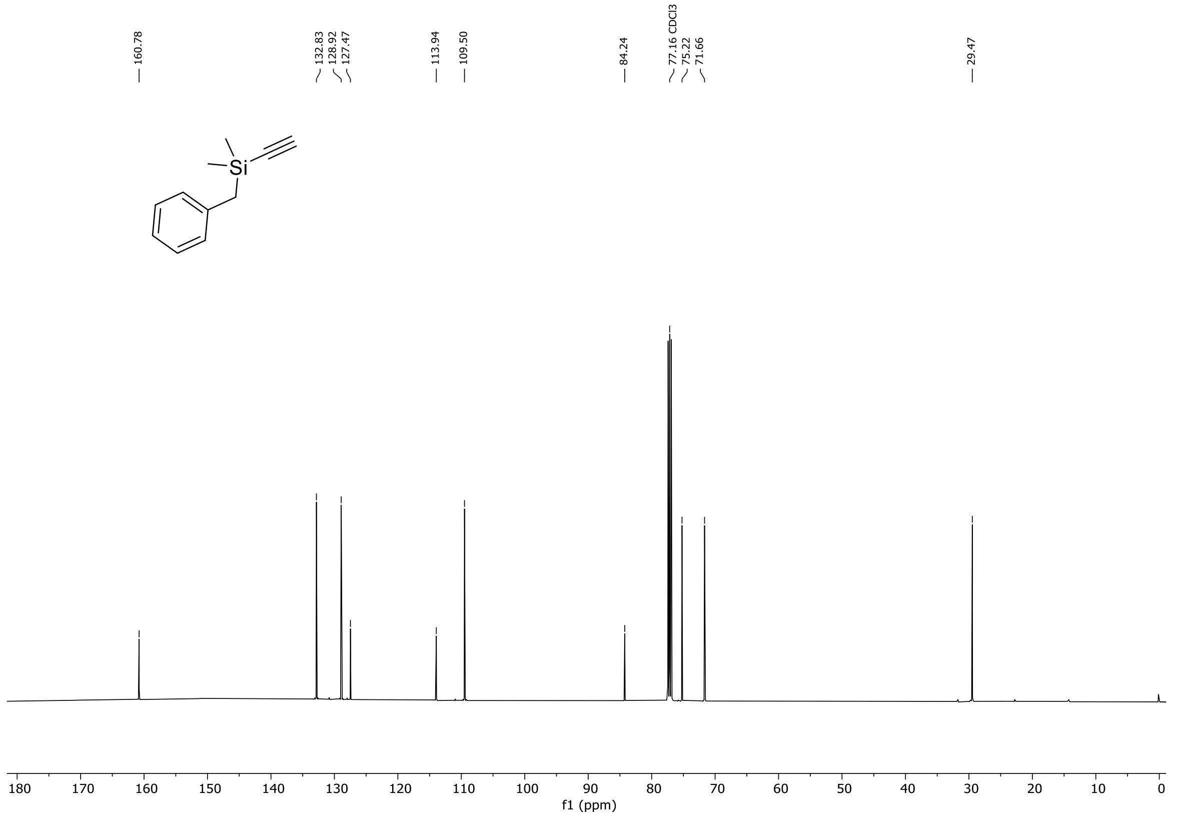

^{1}H NMR (400 MHz, $CDCl_3$) of **5a** (*see NMR data*)

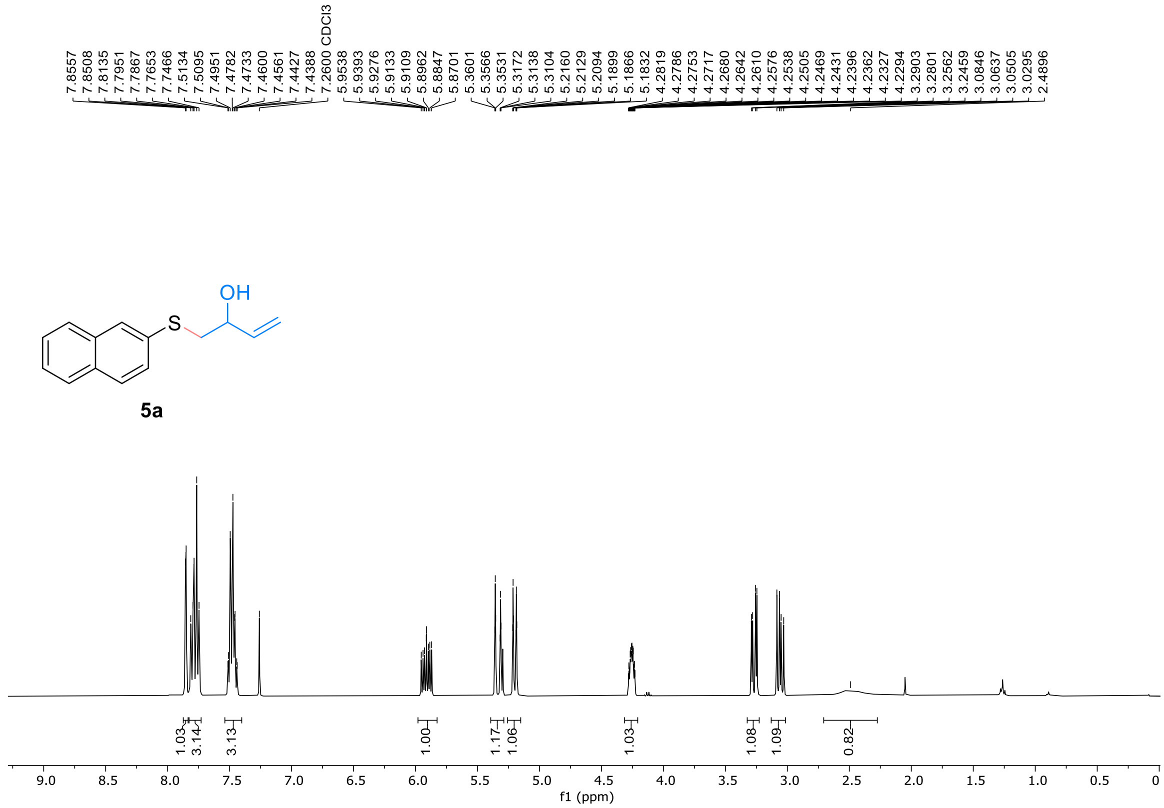

^{13}C NMR (101 MHz, $CDCl_3$) of **5a**

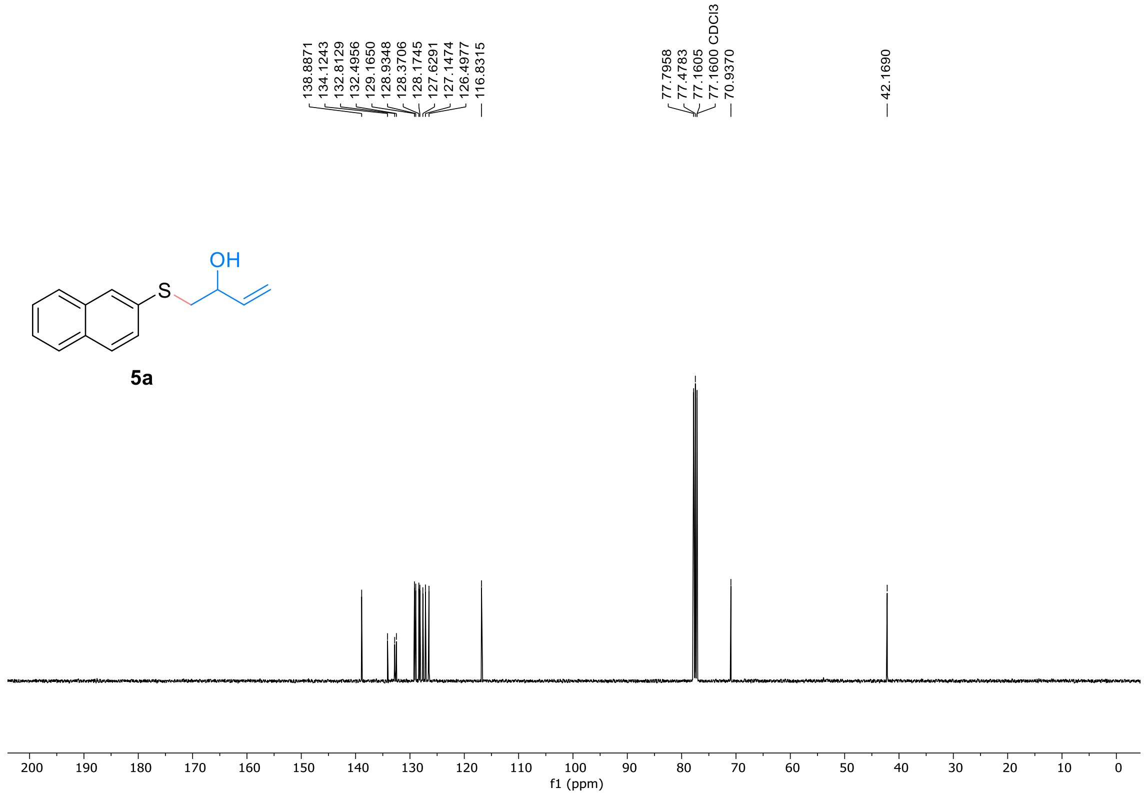

^{1}H NMR (400 MHz, $CDCl_3$) of **6a** (*see NMR data*)

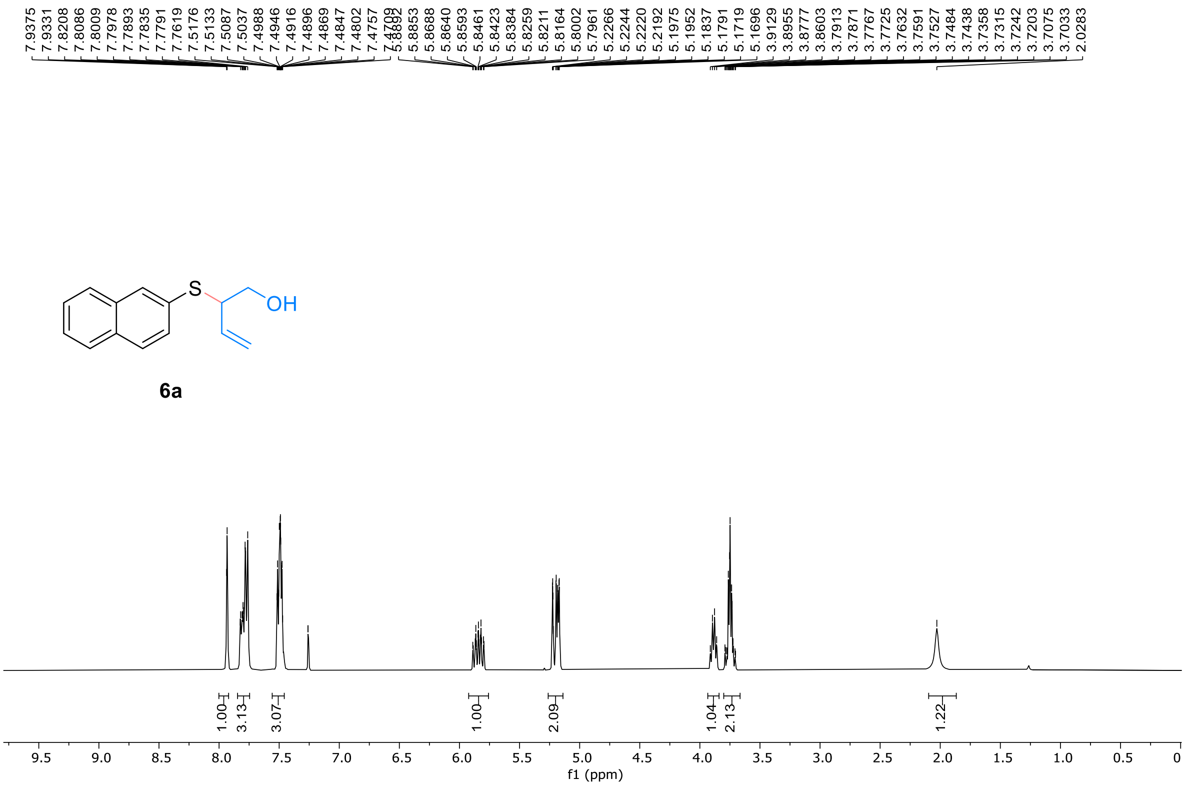

^{13}C NMR (101 MHz, $CDCl_3$) of **6a**

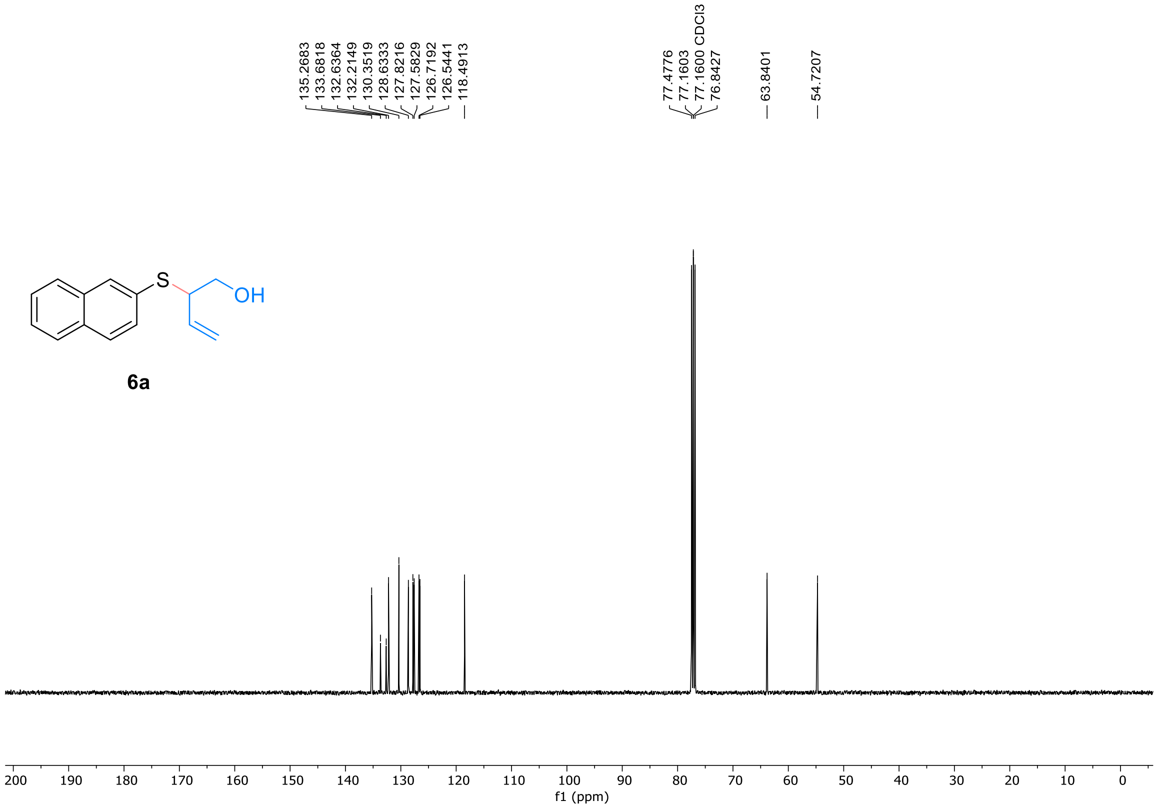

^{1}H NMR (400 MHz, $CDCl_3$) of **4** (*see NMR data*)

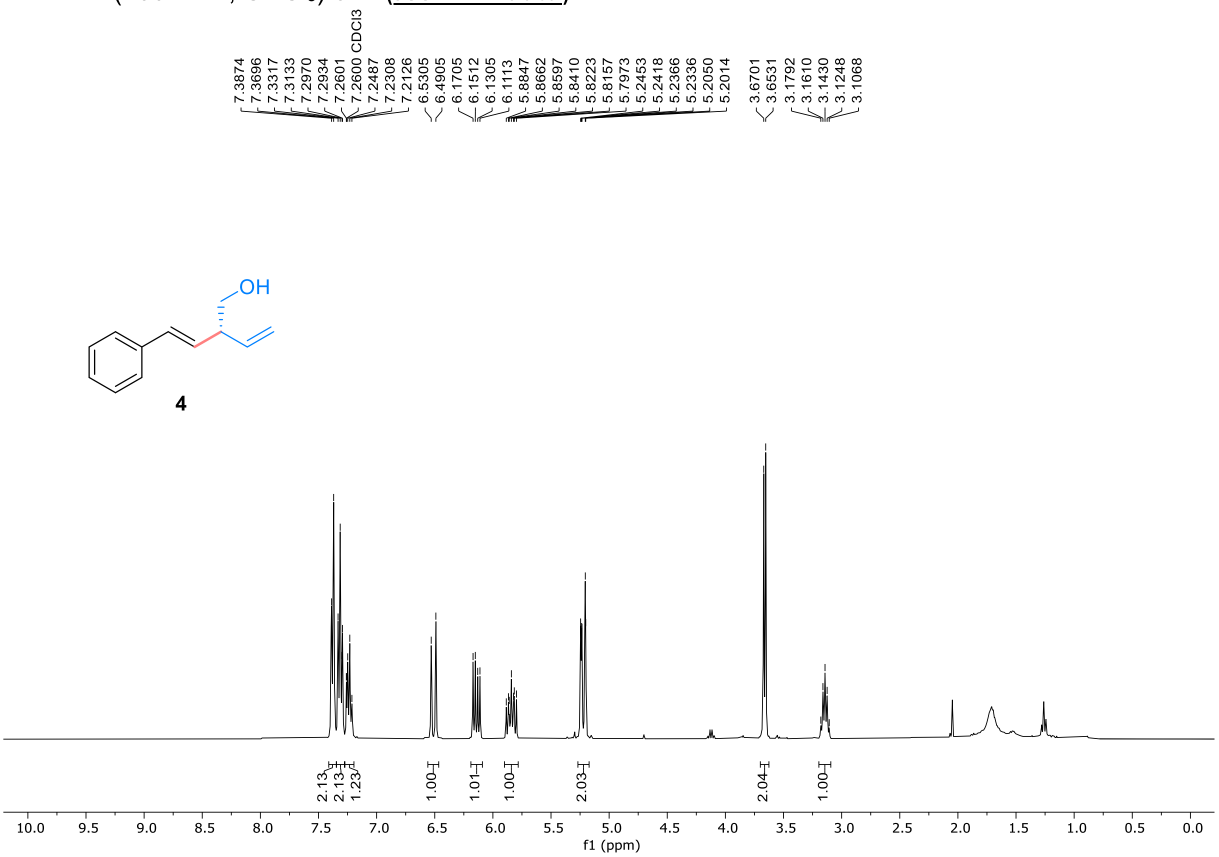

^{13}CNMR (101 MHz, $CDCl_3$) of **4**

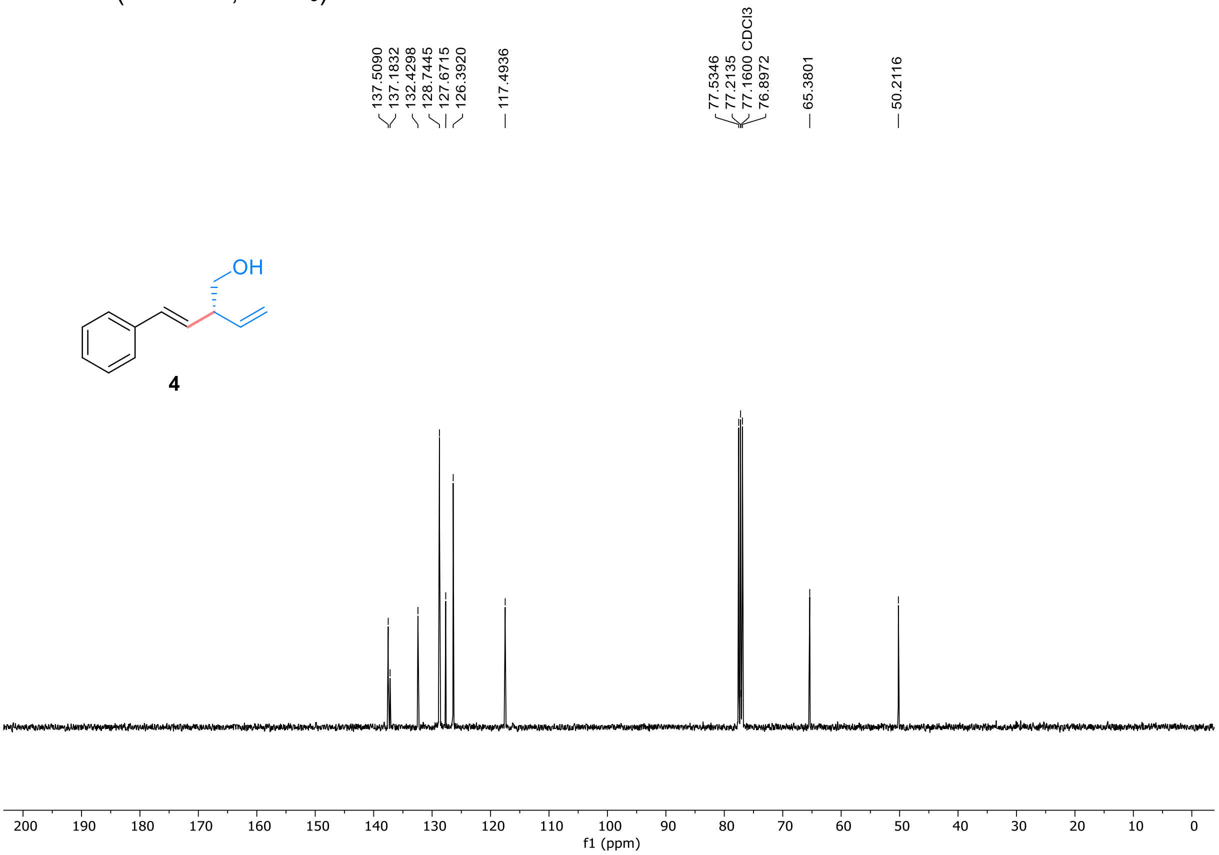

^{1}H NMR (400 MHz, $CDCl_3$) of **5** (*see NMR data*)

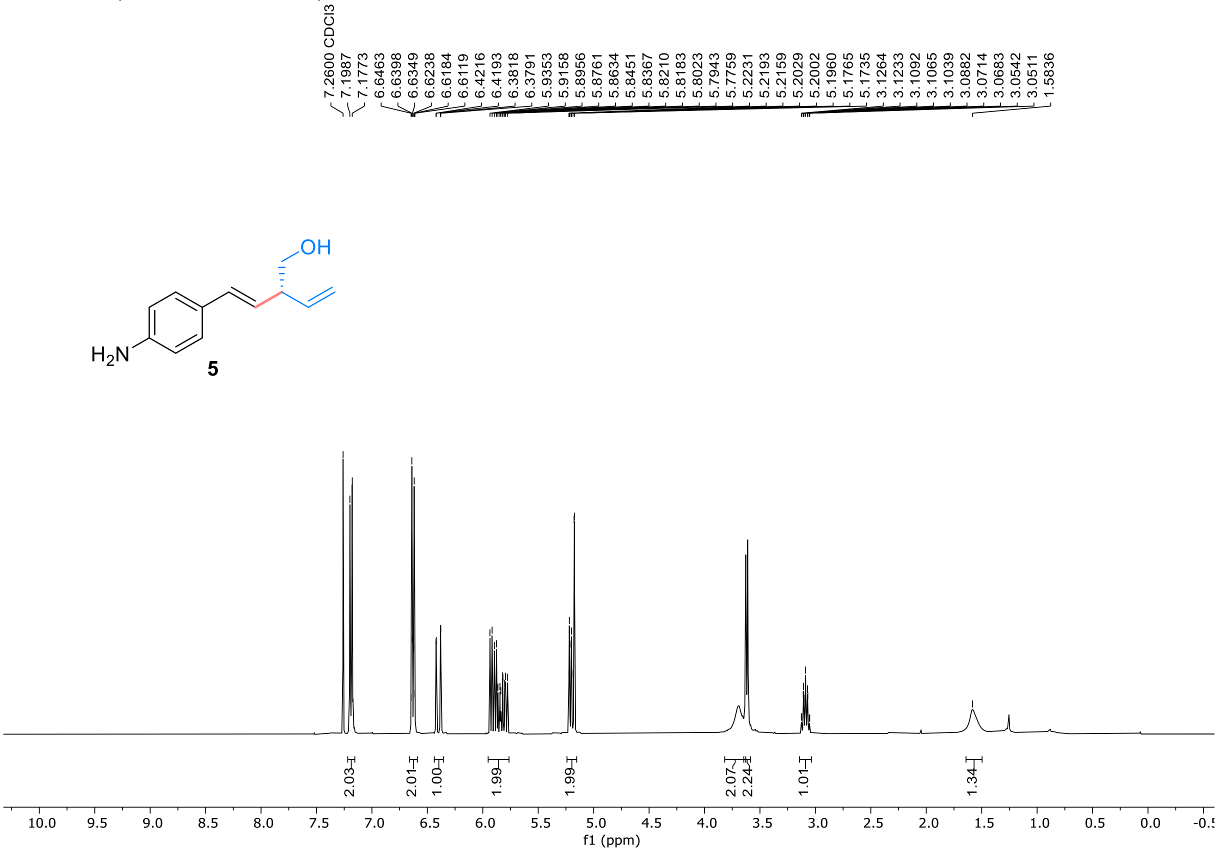

^{13}C NMR (101 MHz, $CDCl_3$) of **5**

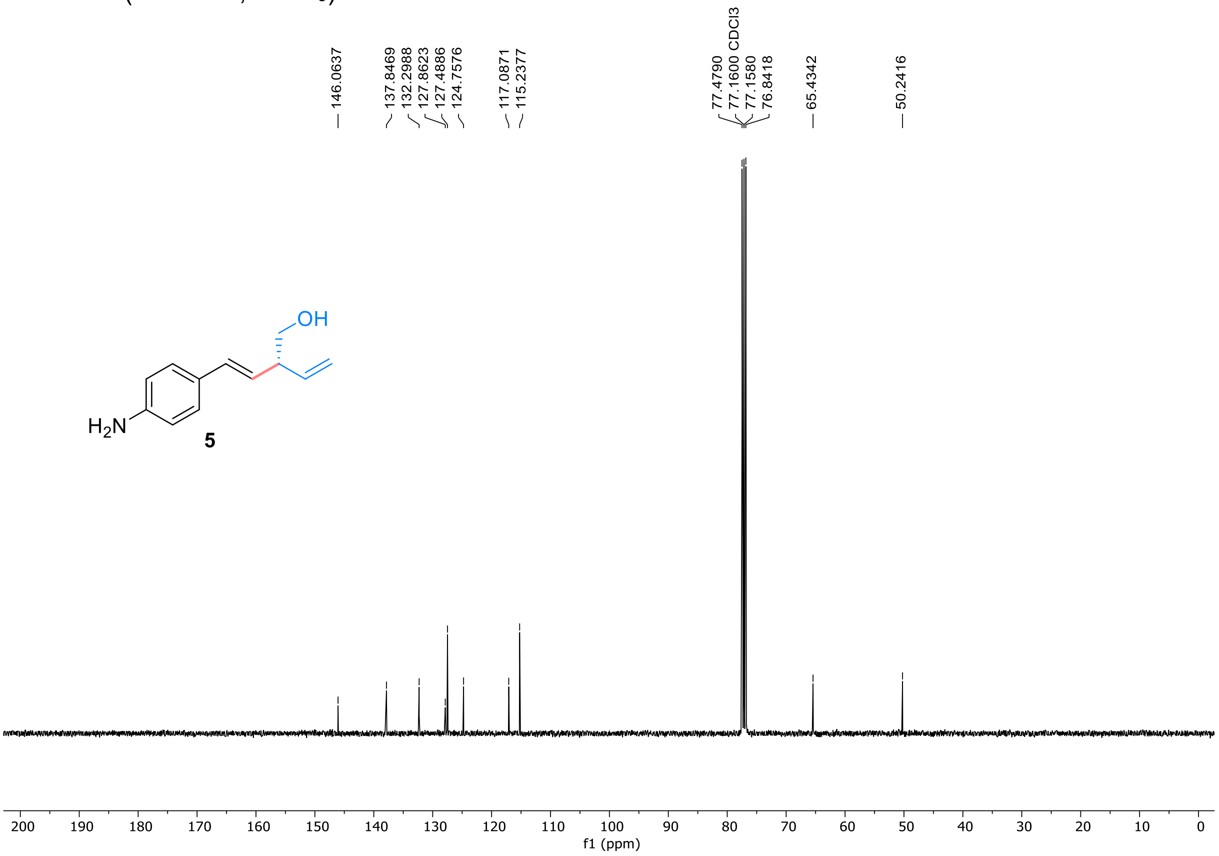

^{1}H NMR (400 MHz, $CDCl_3$) of **6** (*see NMR data*)

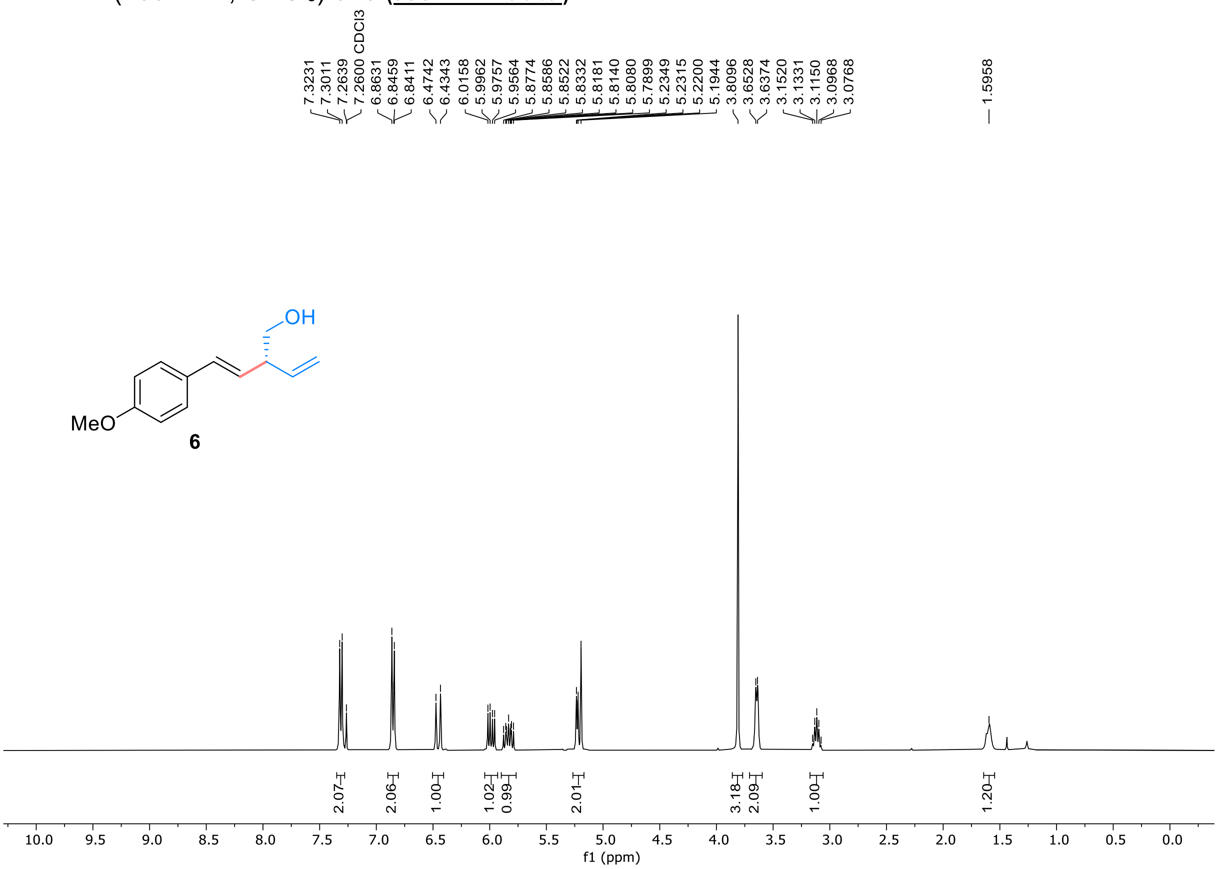

^{13}C NMR (101 MHz, $CDCl_3$) of **6**

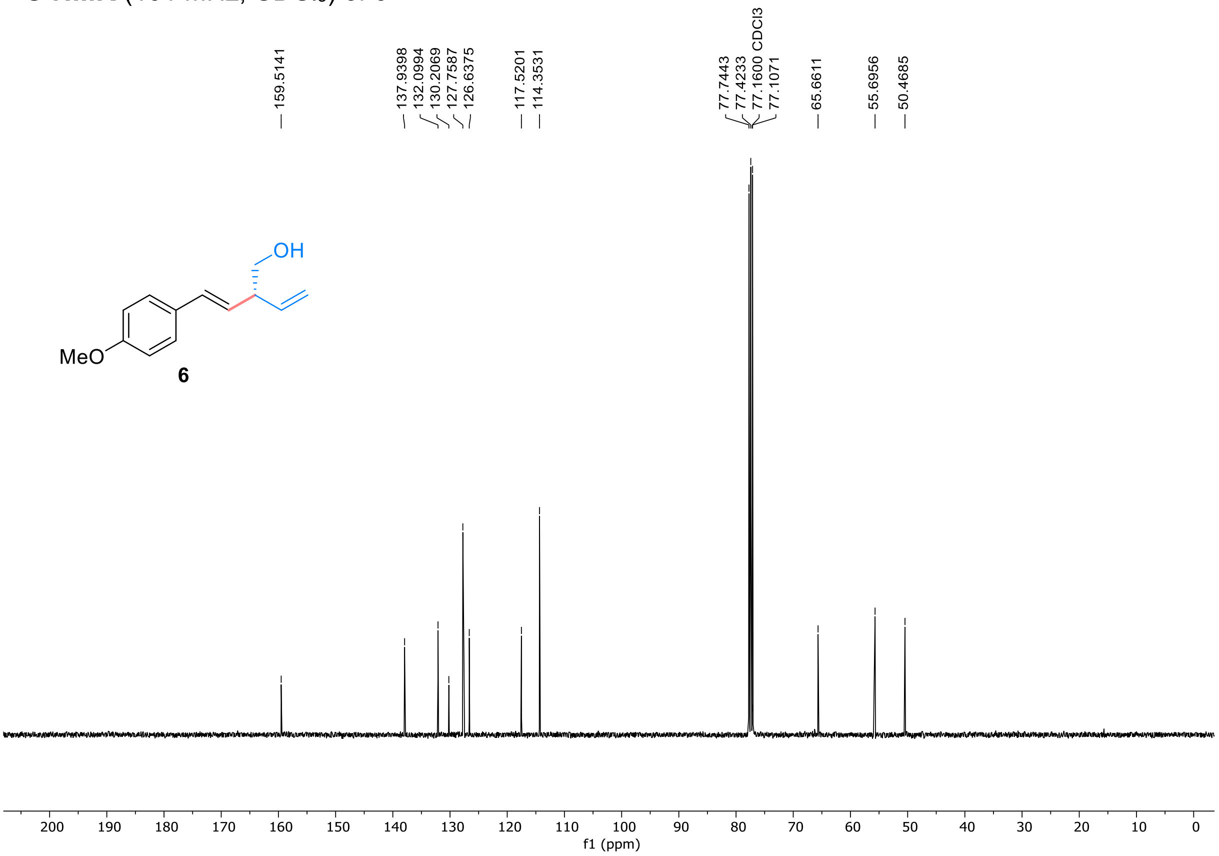

^{1}H NMR (400 MHz, $CDCl_3$) of **7** (*see NMR data*)

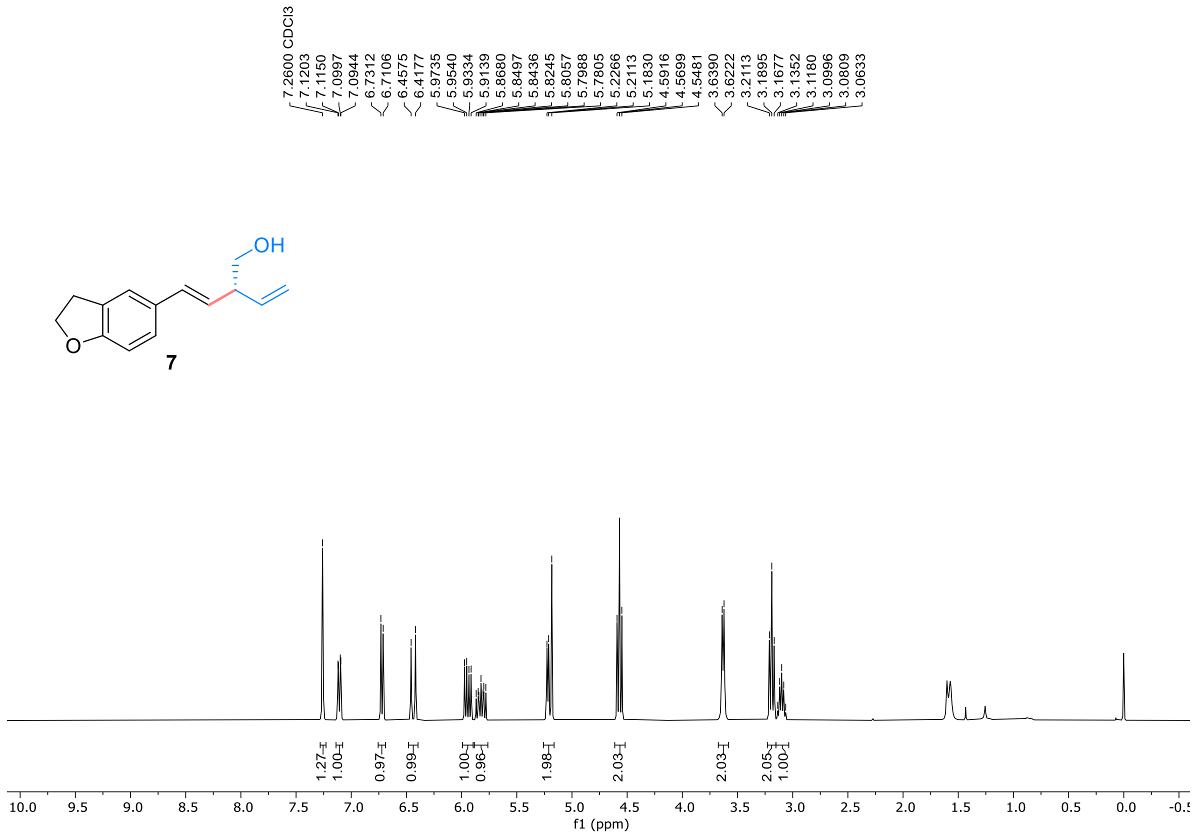

^{13}C NMR (101 MHz, $CDCl_3$) of **7**

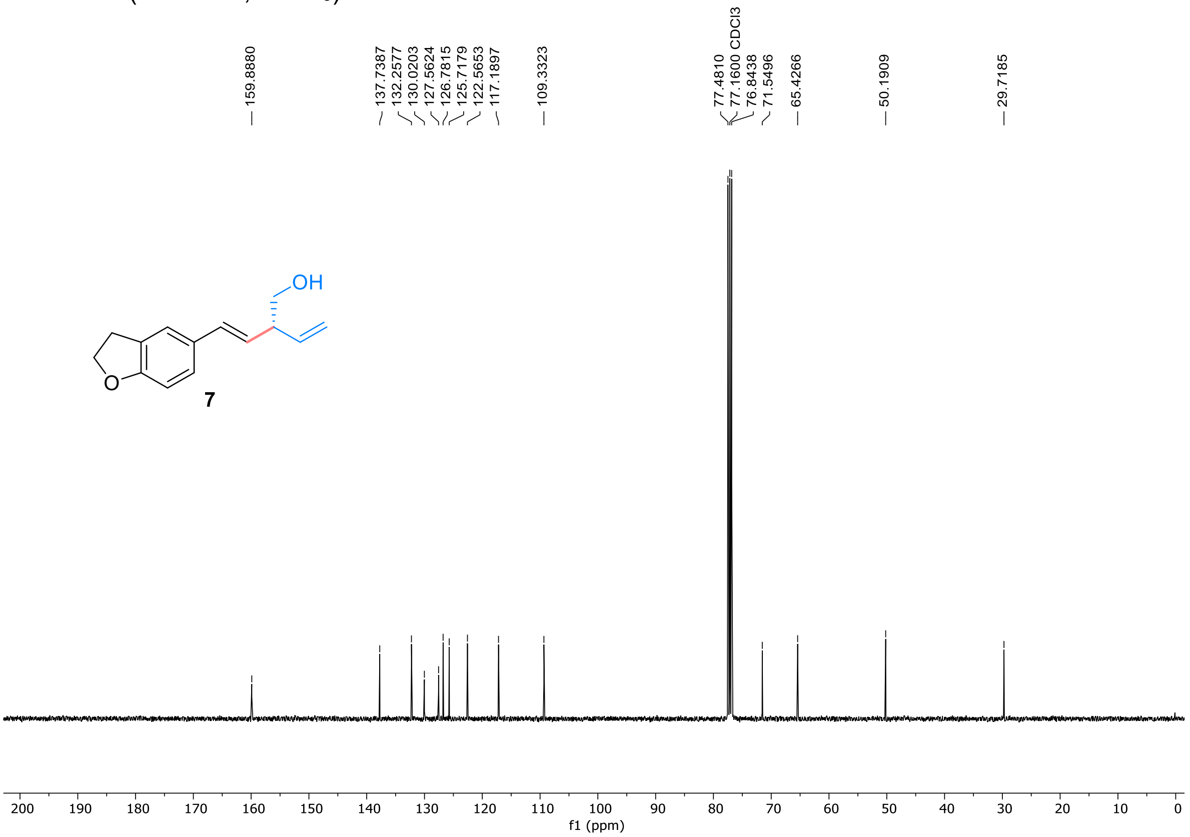

^{1}H NMR (400 MHz, $CDCl_3$) of **8** (*see NMR data*)

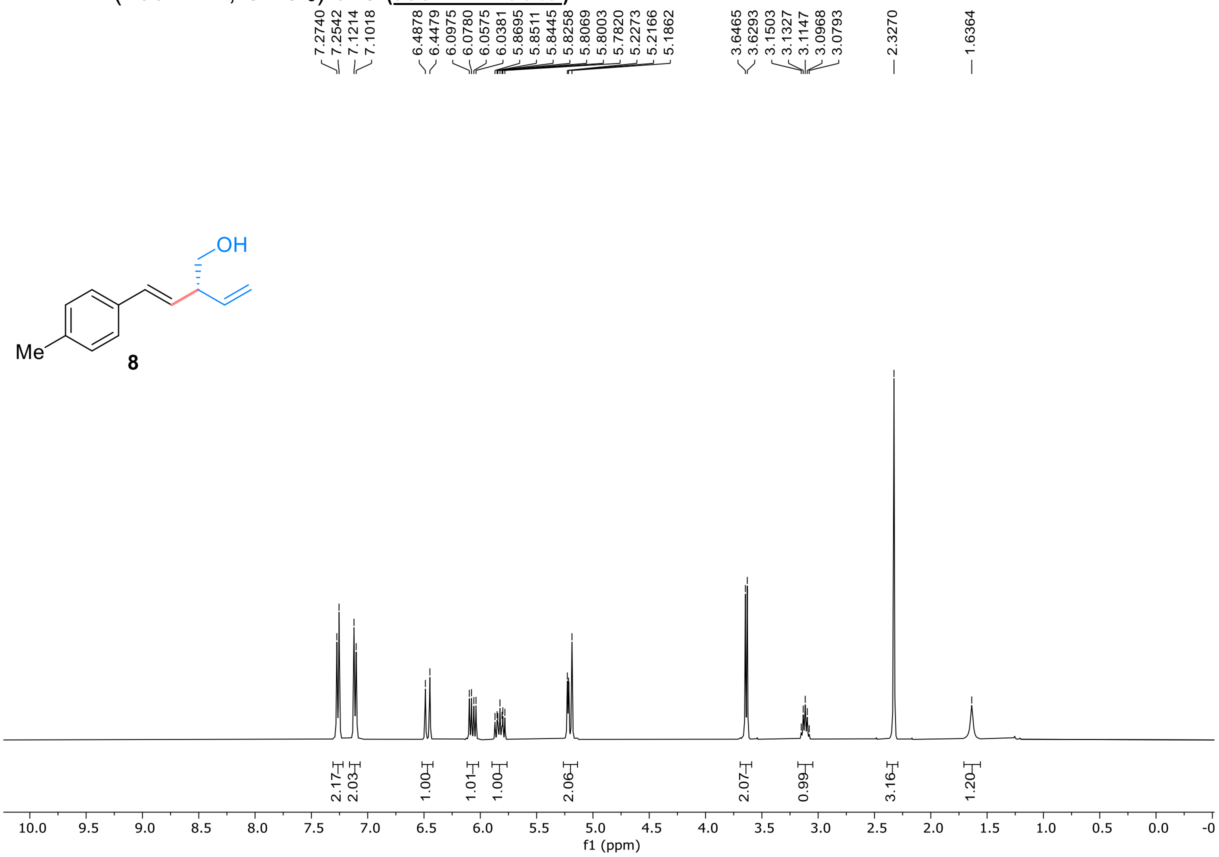

^{13}C NMR (101 MHz, $CDCl_3$) of **8**

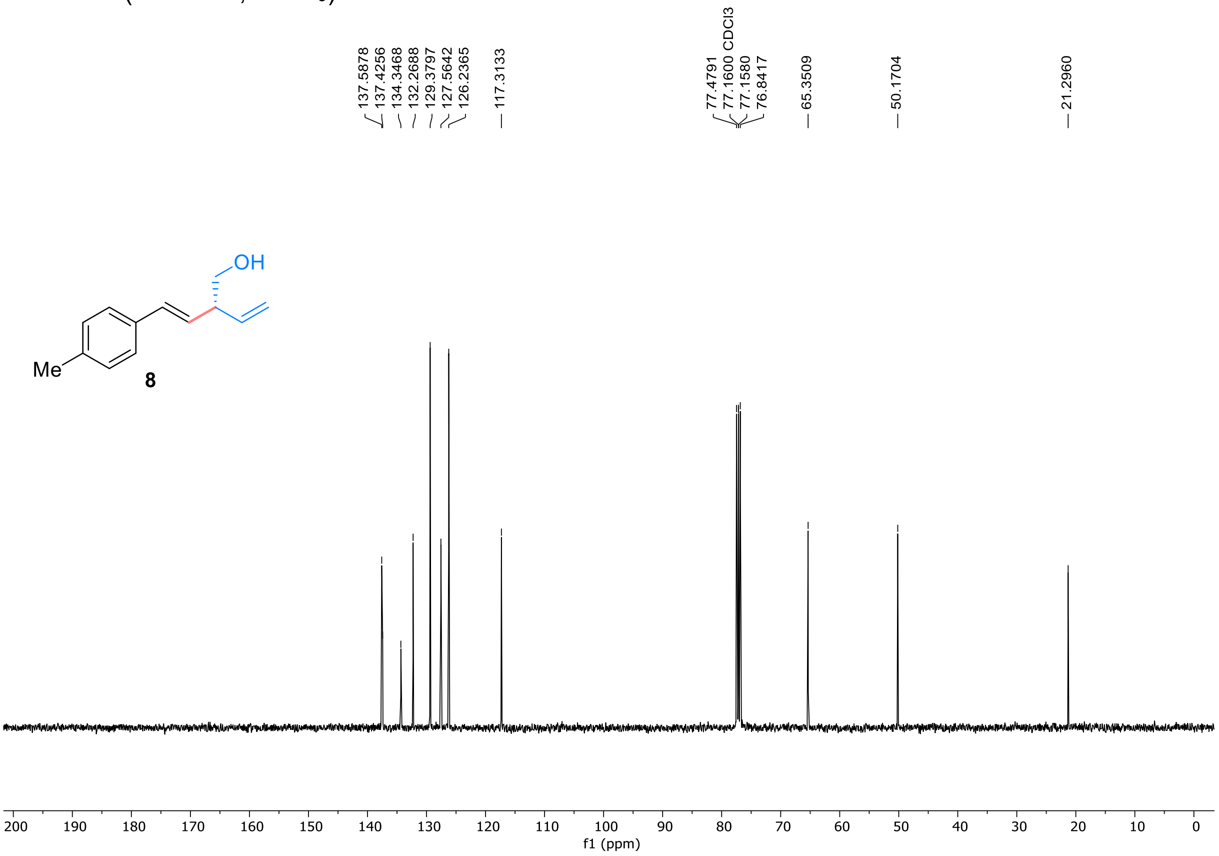

^{1}H NMR (400 MHz, $CDCl_3$) of **9** (*see NMR data*)

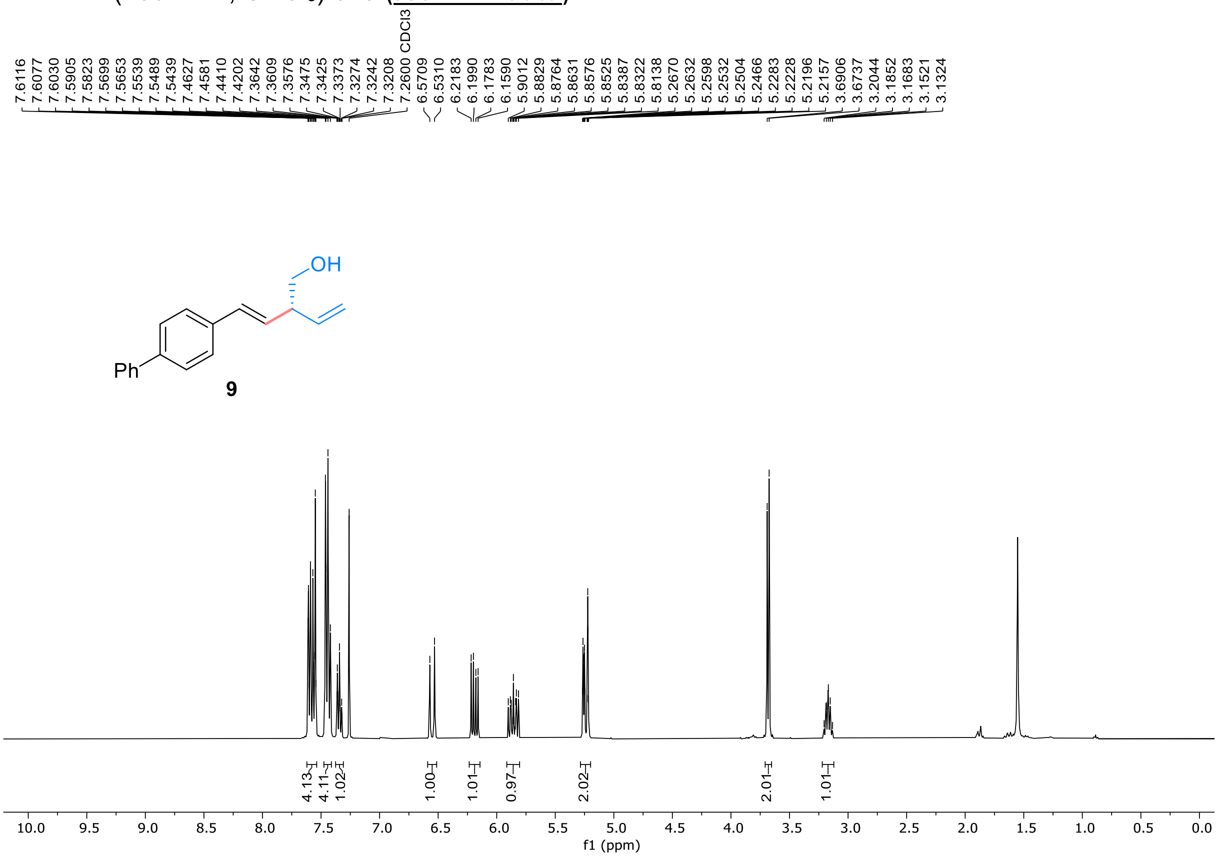

^{13}C NMR (101 MHz, $CDCl_3$) of **9**

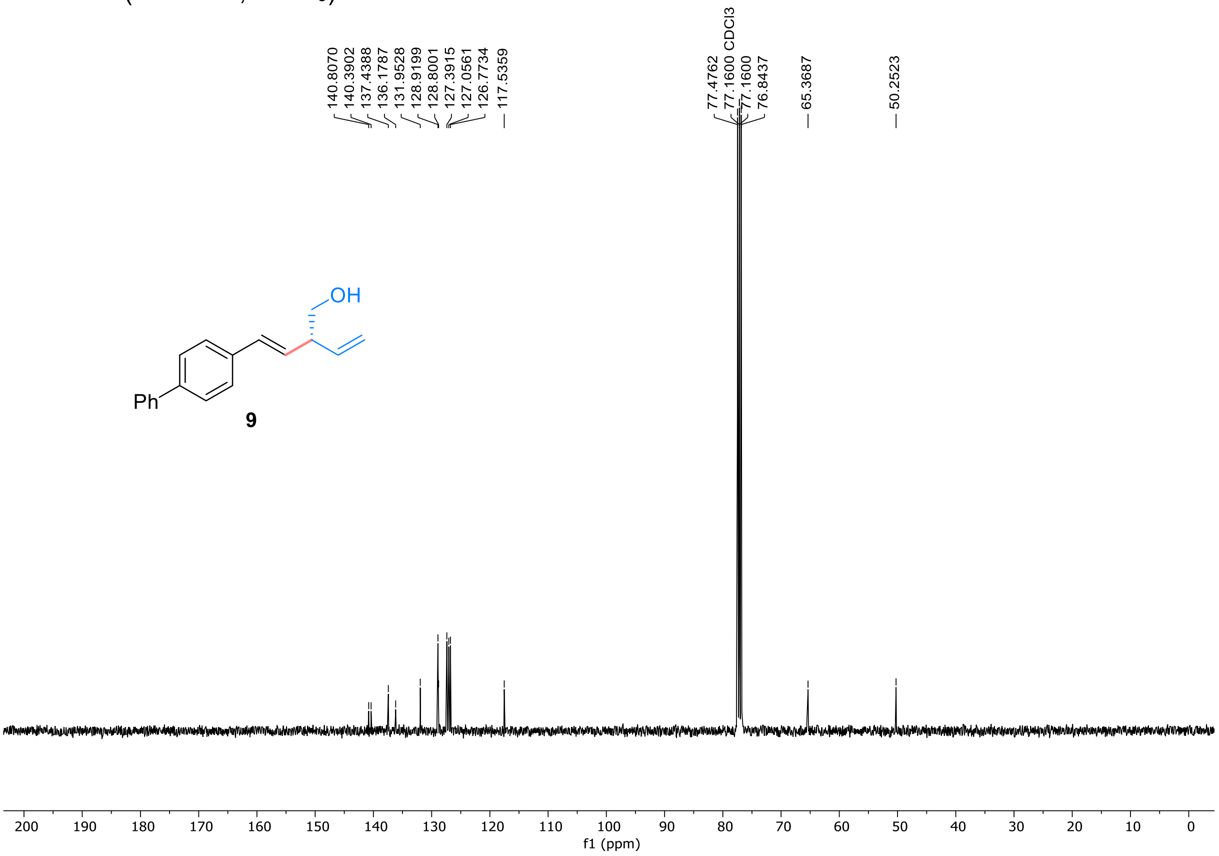

^{1}H NMR (400 MHz, $CDCl_3$) of **10** (*see NMR data*)

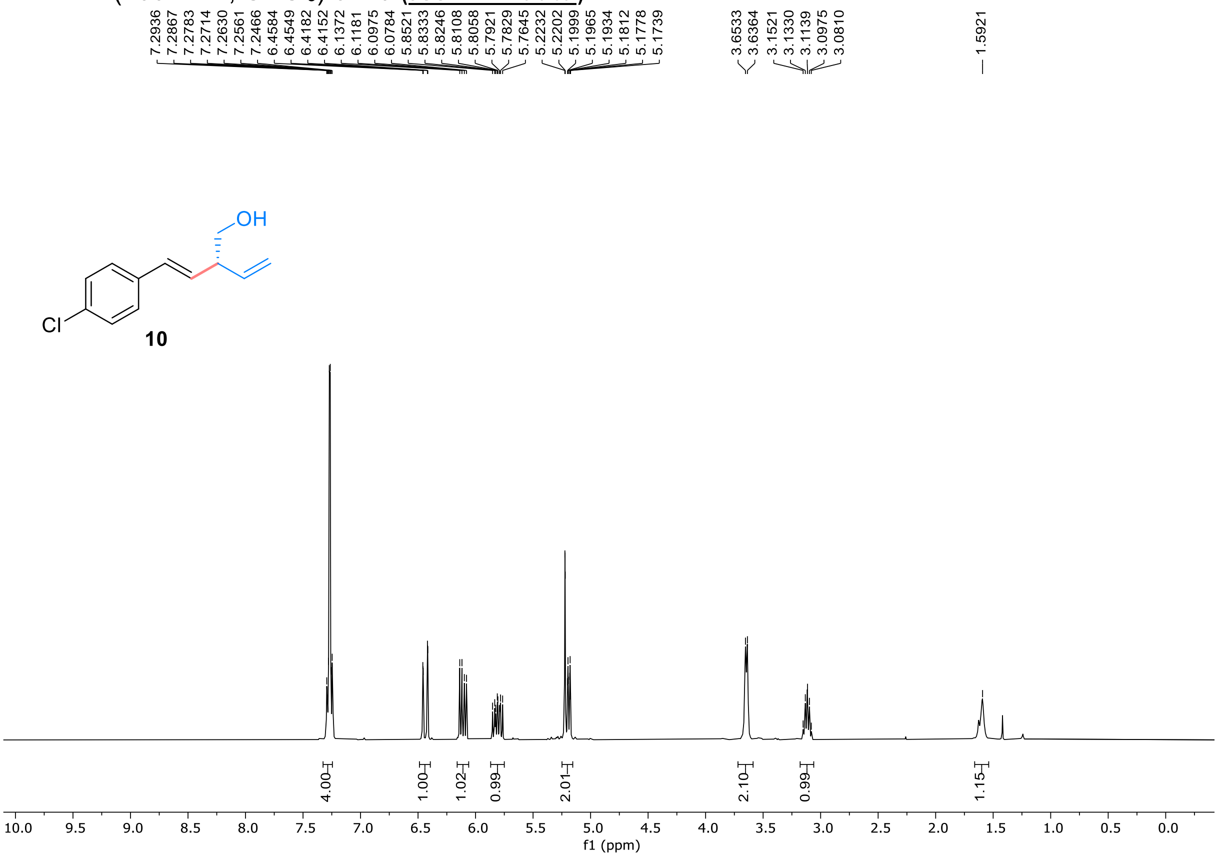

^{13}C NMR (101 MHz, $CDCl_3$) of **10**

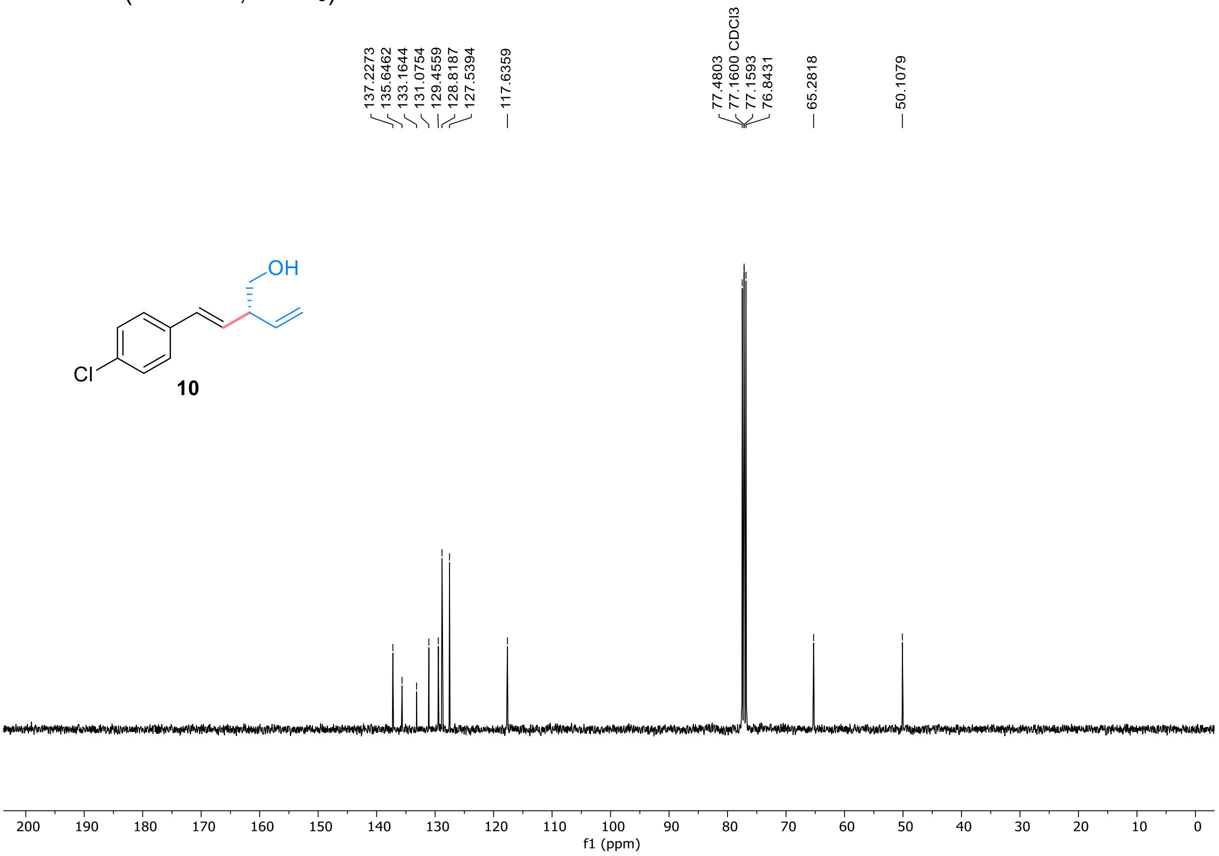

^{1}H NMR (400 MHz, $CDCl_3$) of **11** (*see NMR data*)

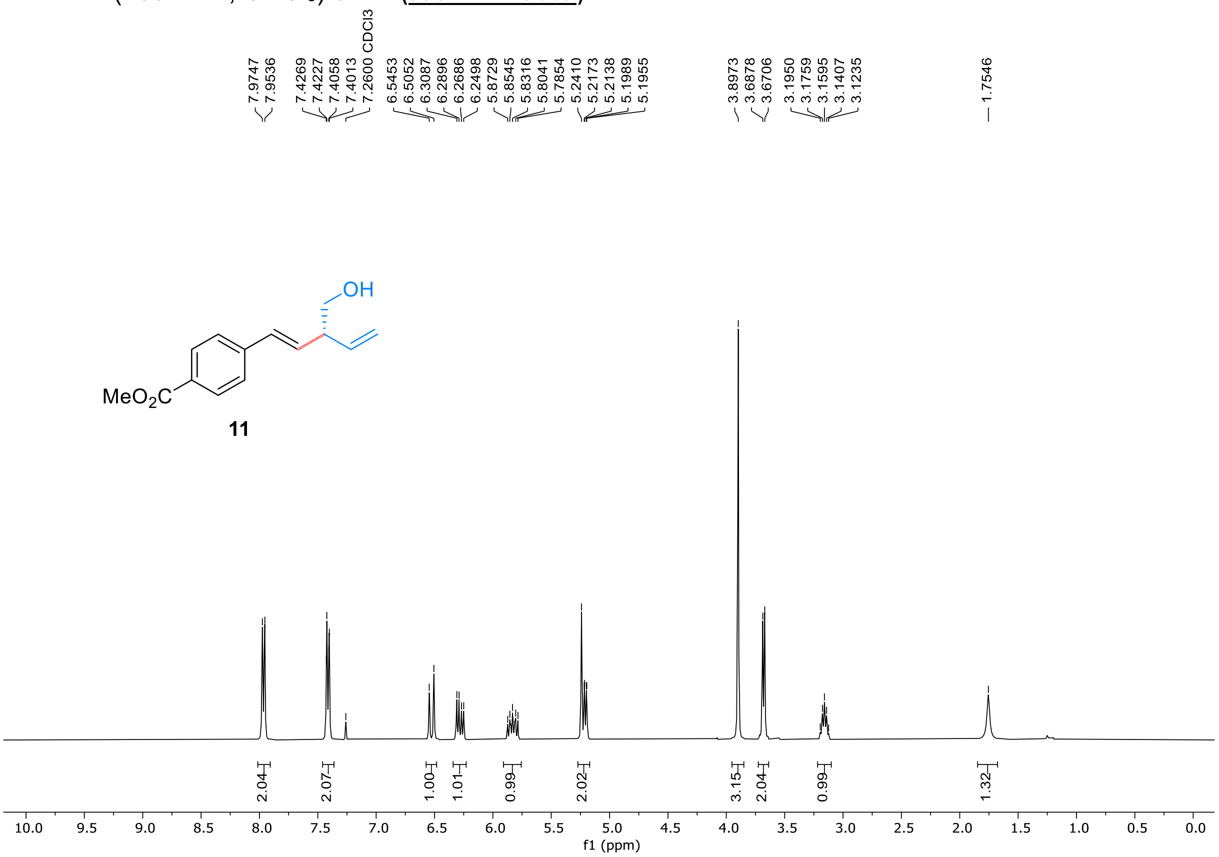

^{13}C NMR (101 MHz, $CDCl_3$) of **11**

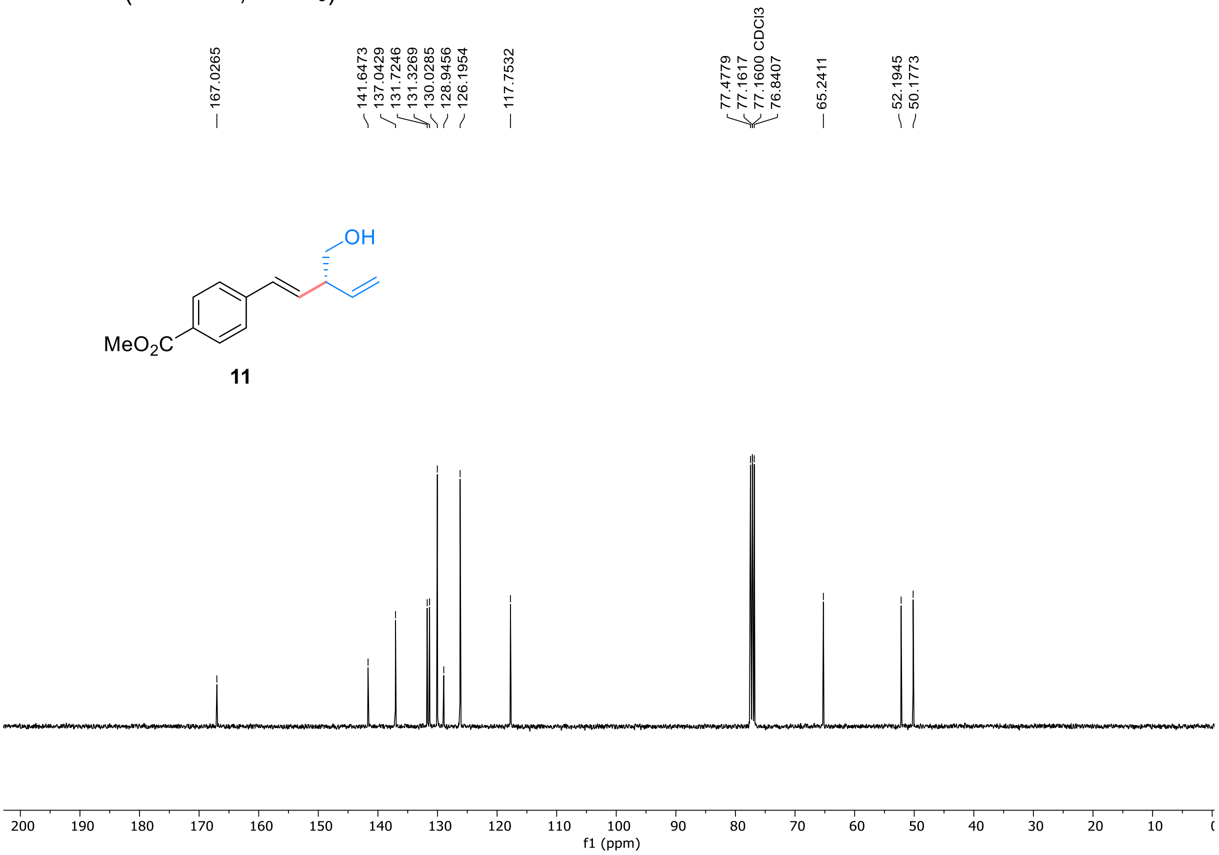

^{1}H NMR (400 MHz, $CDCl_3$) of **12** (*see NMR data*)

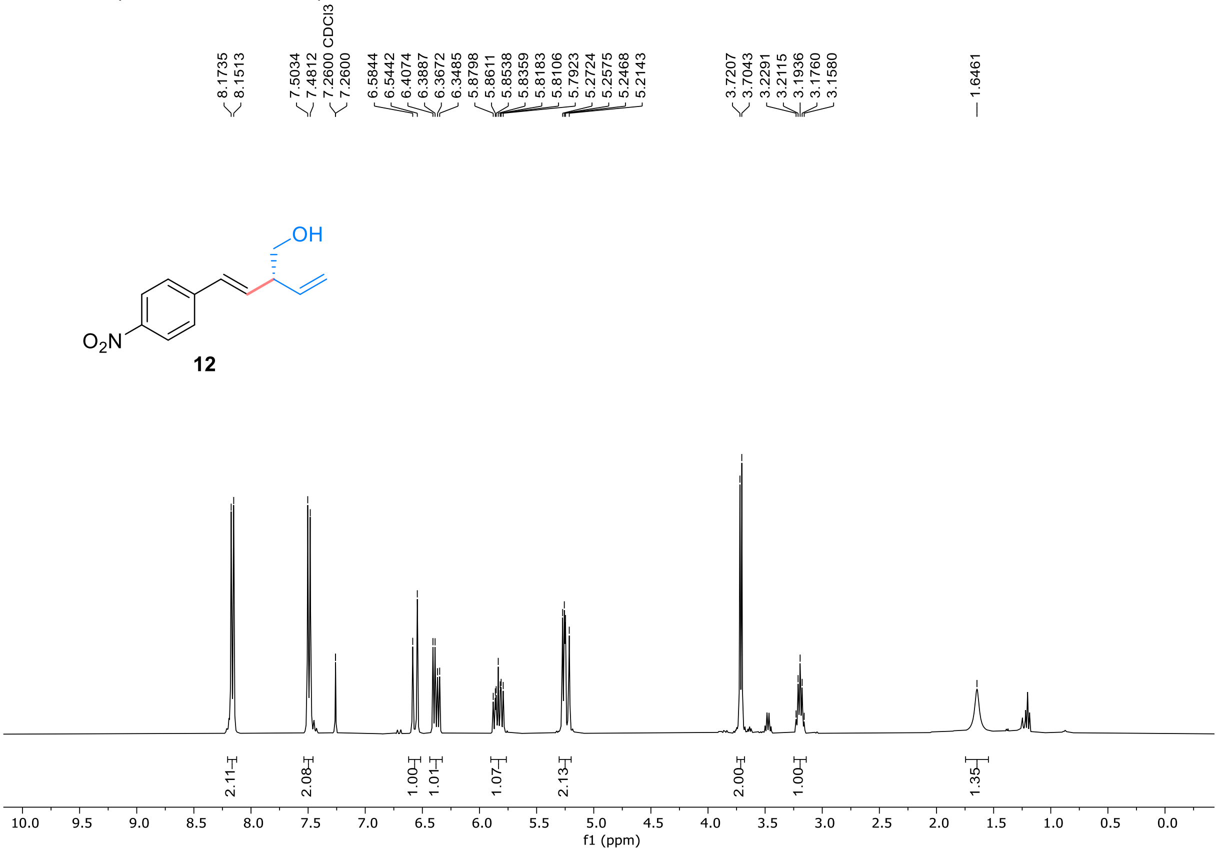

^{13}C NMR (101 MHz, $CDCl_3$) of **12**

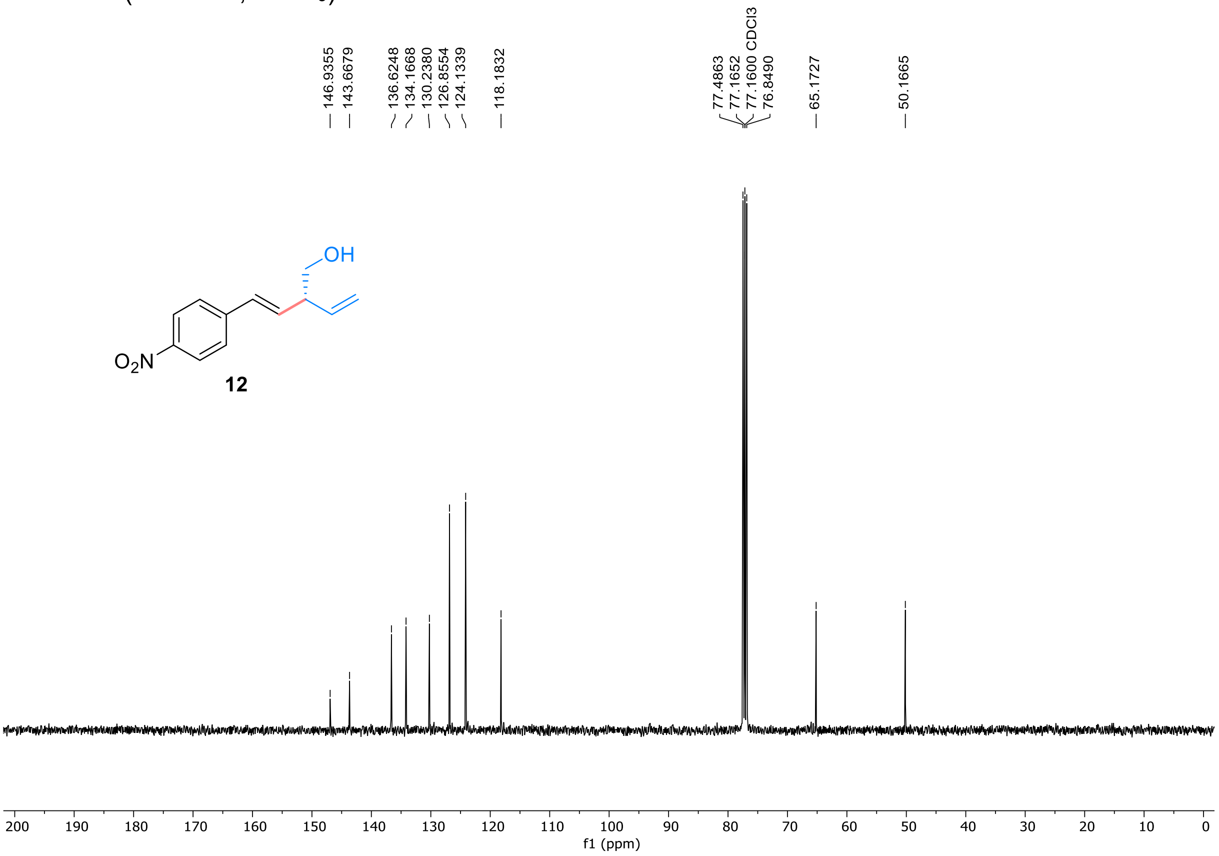

^{1}H NMR (400 MHz, $CDCl_3$) of **13** (*see NMR data*)

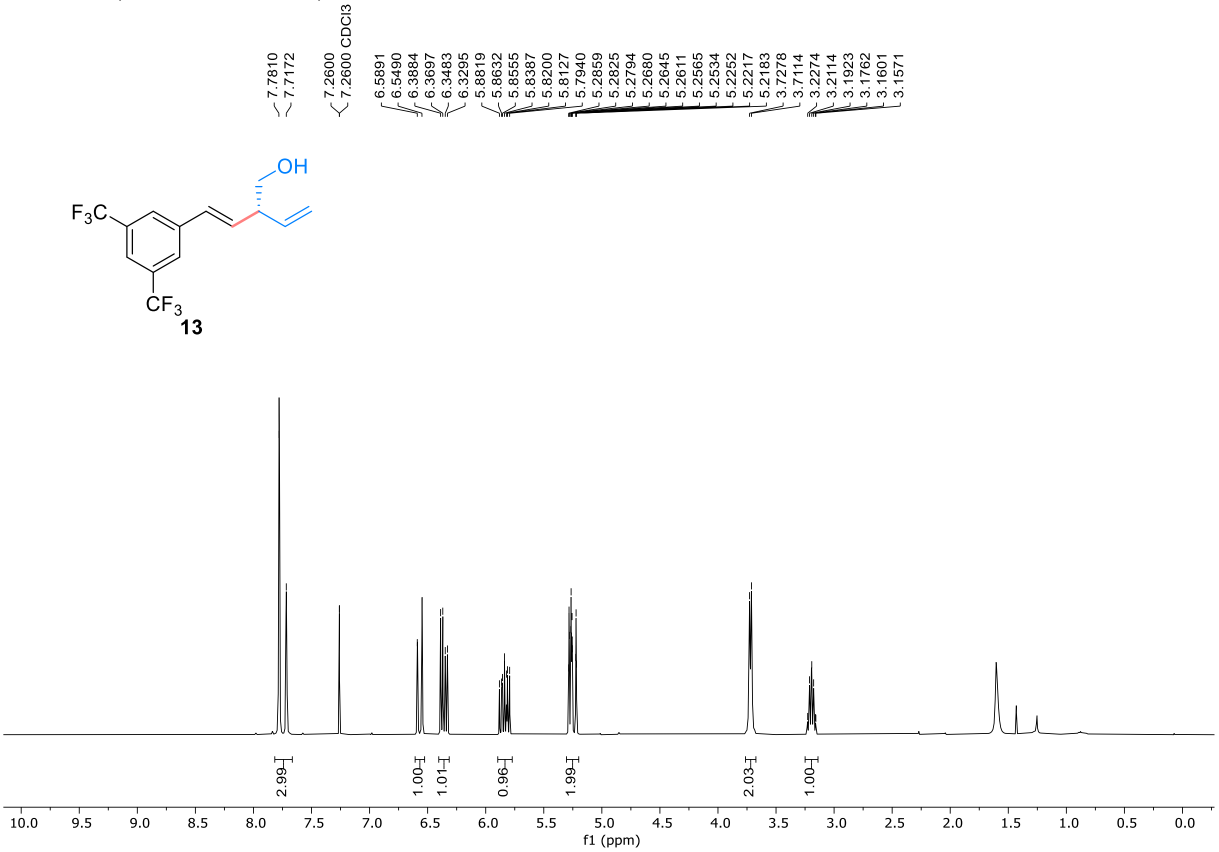

^{13}C NMR (101 MHz, $CDCl_3$) of **13**

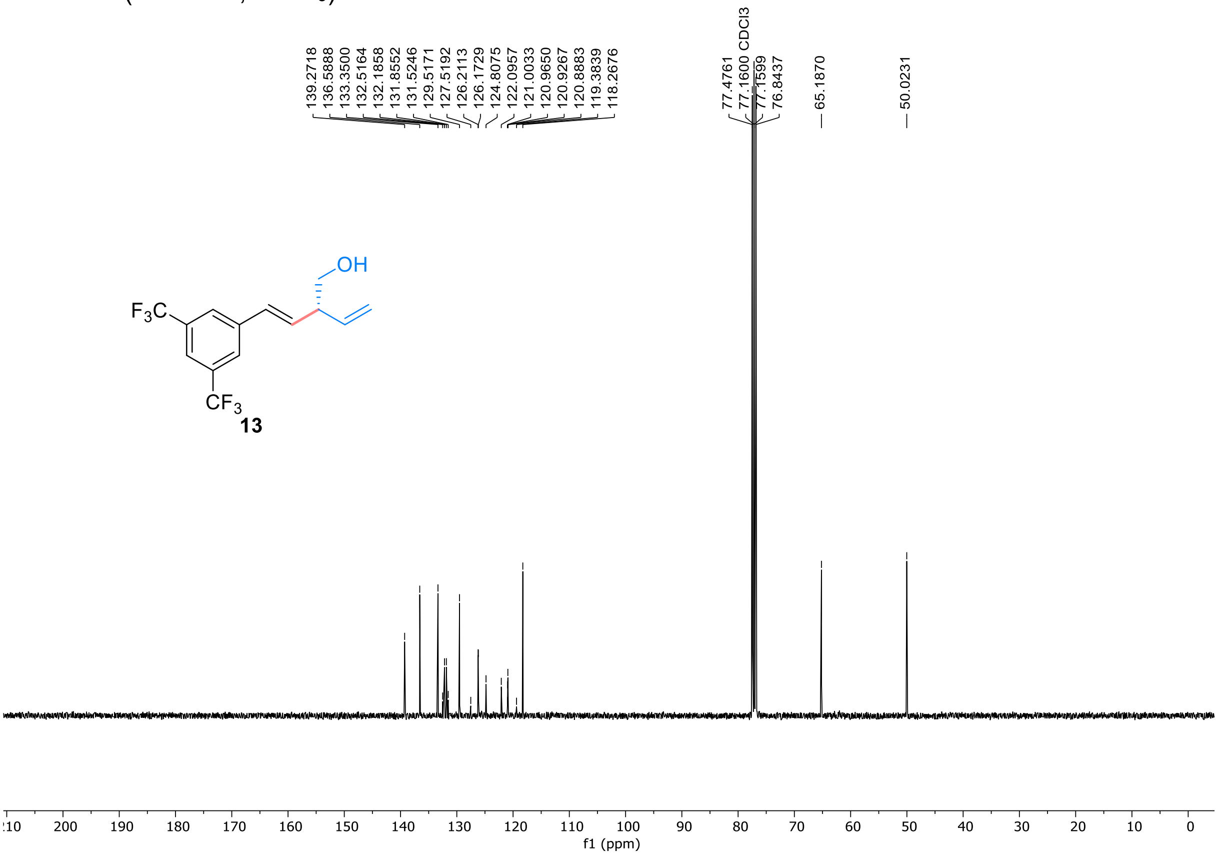

^{1}H NMR (400 MHz, $CDCl_3$) of **14** (*see NMR data*)

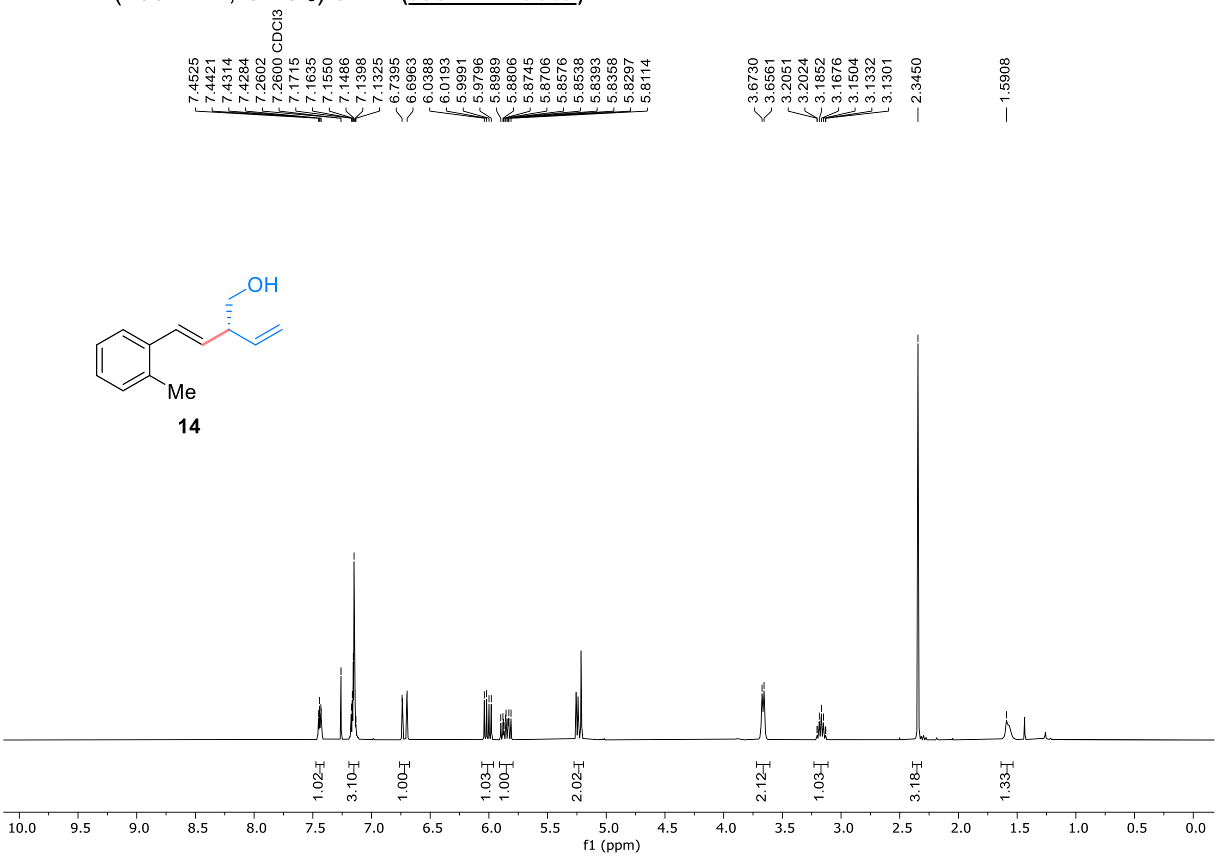

^{13}C NMR (101 MHz, $CDCl_3$) of **14**

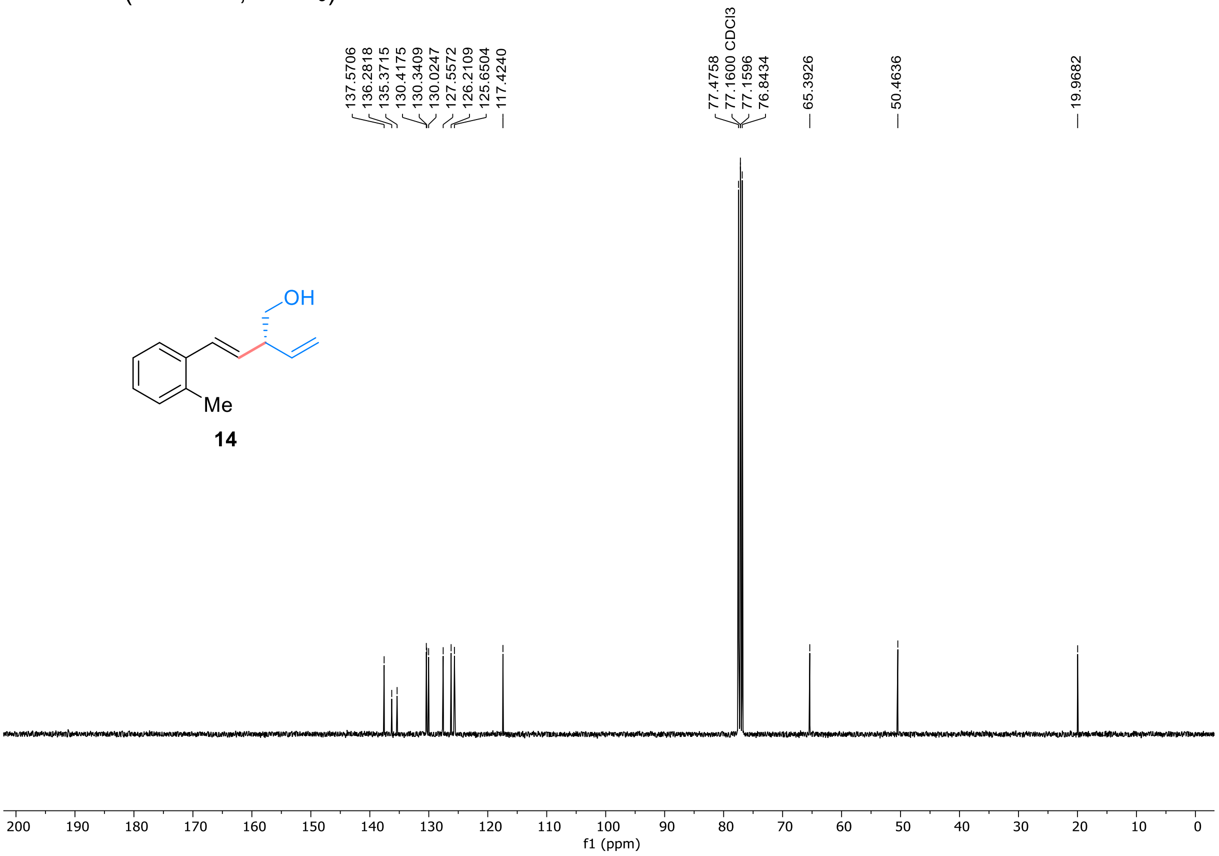

^{1}H NMR (400 MHz, CDCl$_3$) of **15**(*see NMR data*)

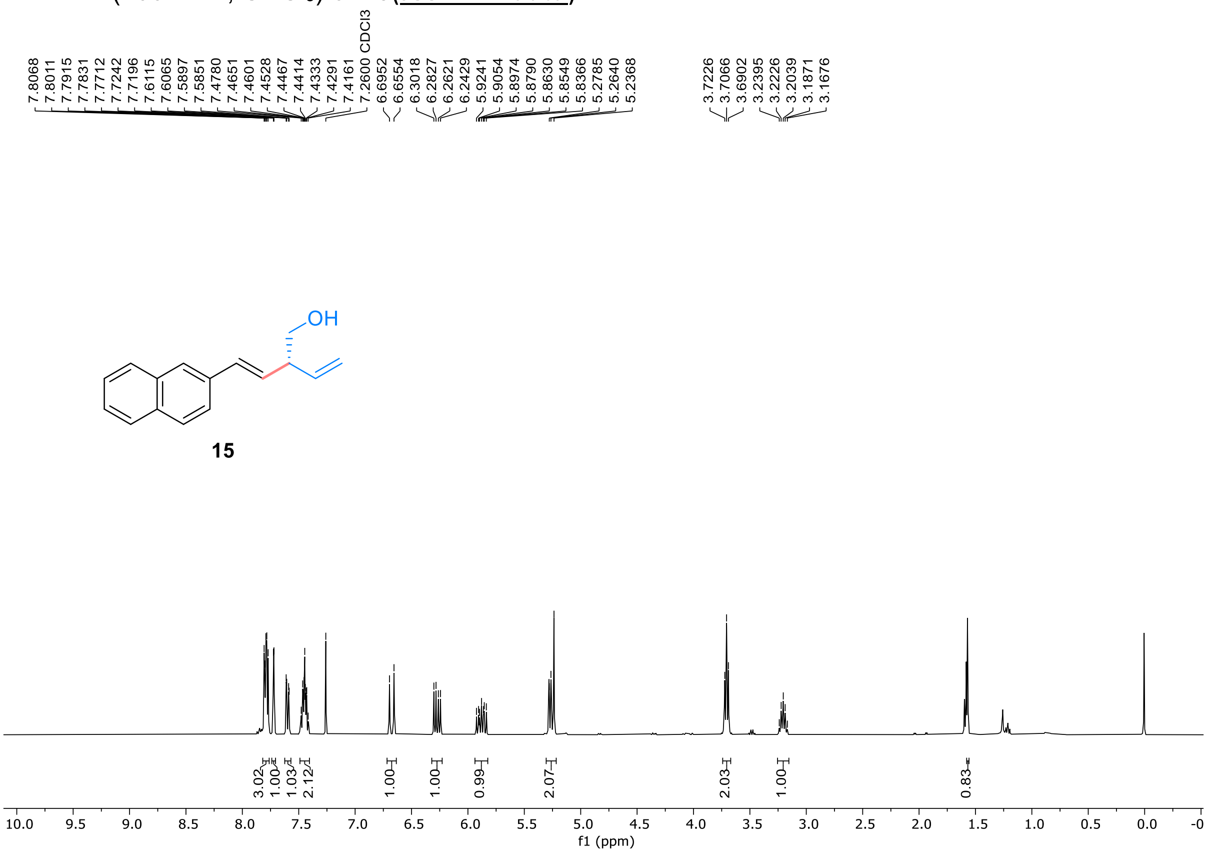

^{13}C NMR (101 MHz, CDCl$_3$) of **15**

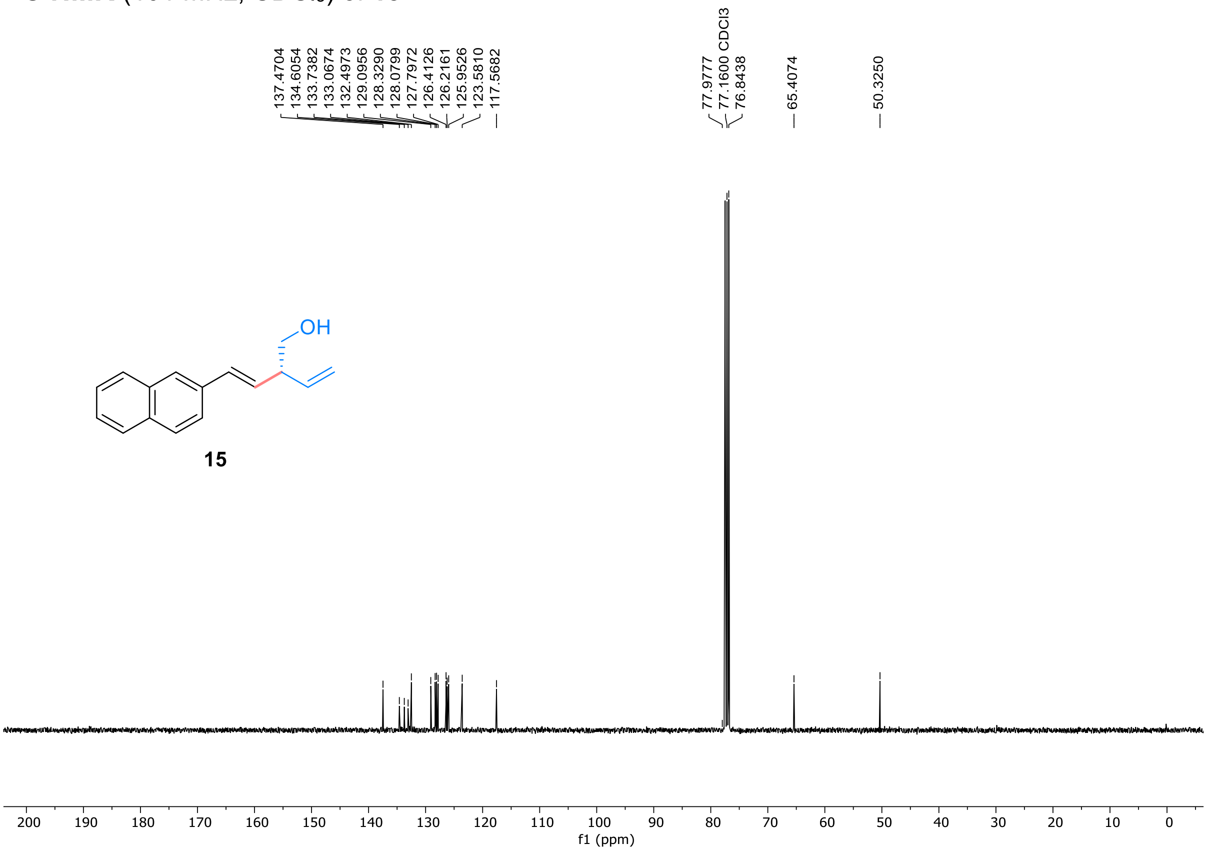

^{1}H NMR (400 MHz, CDCl$_3$) of **16**(*see NMR data*)

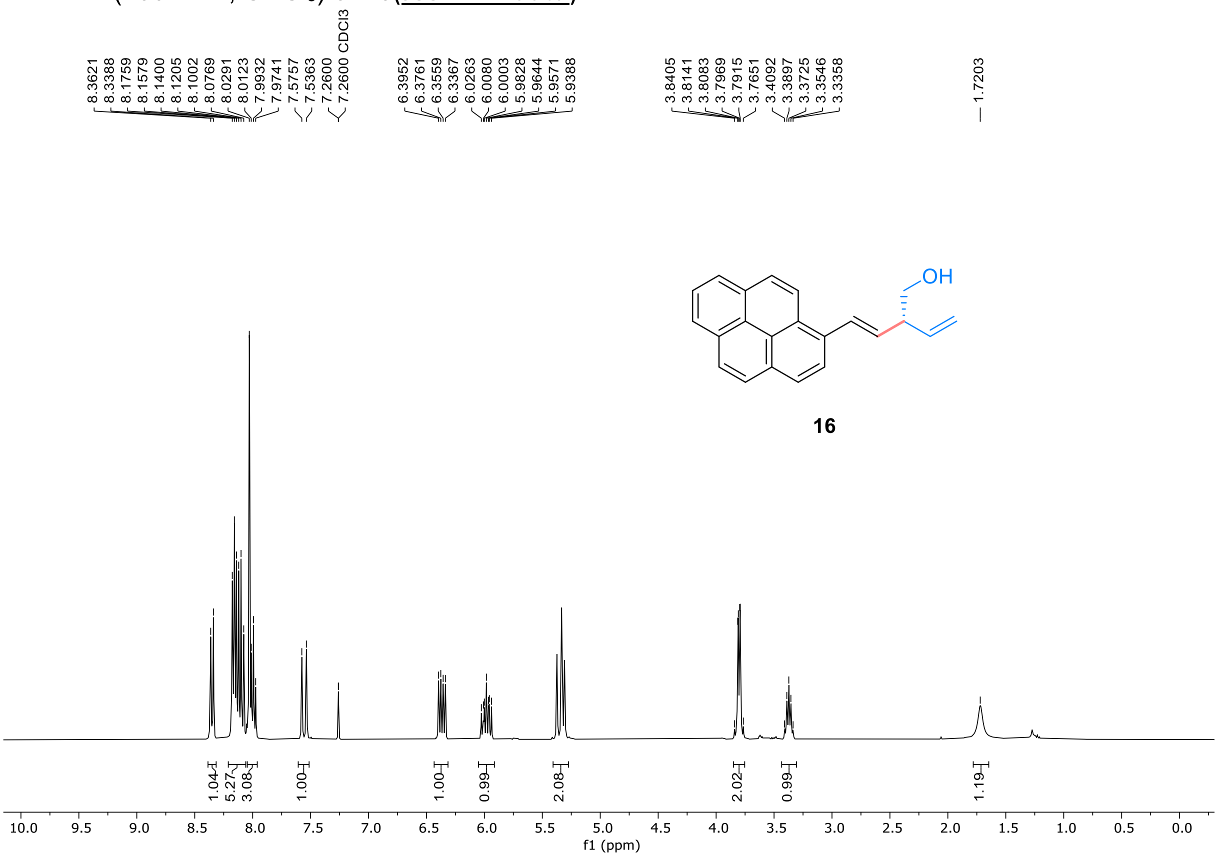

^{13}C NMR (101 MHz, CDCl$_3$) of **16**

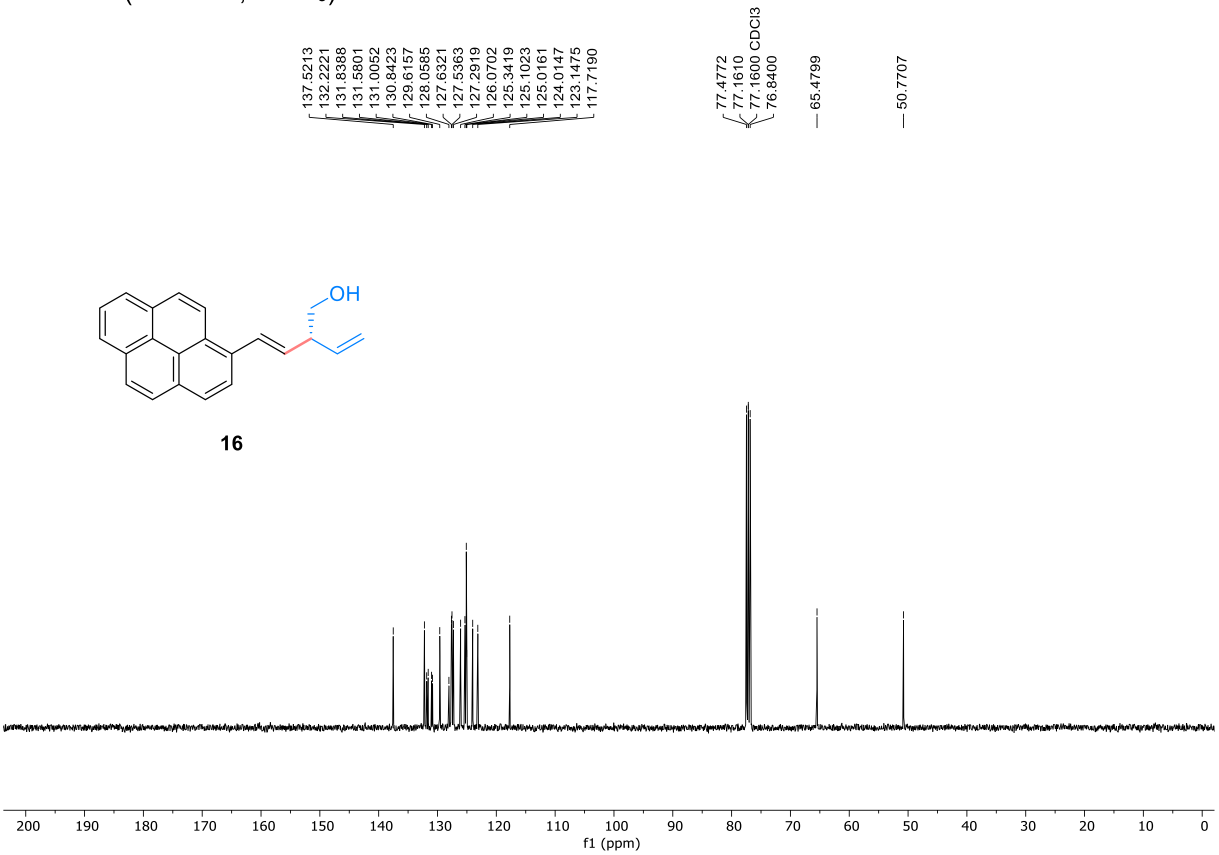

^{1}H NMR (400 MHz, $CDCl_3$) of **17** (*see NMR data*)

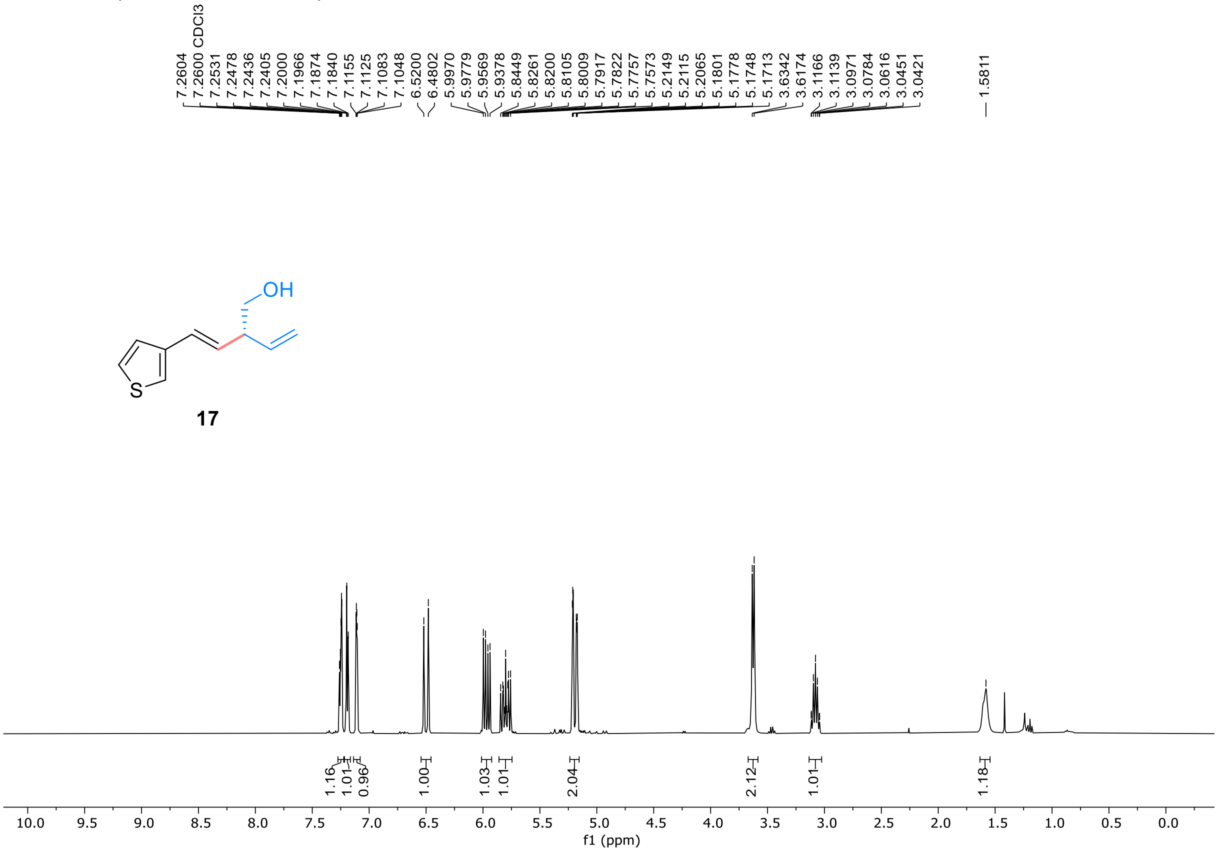

^{13}C NMR (101 MHz, $CDCl_3$) of **17**

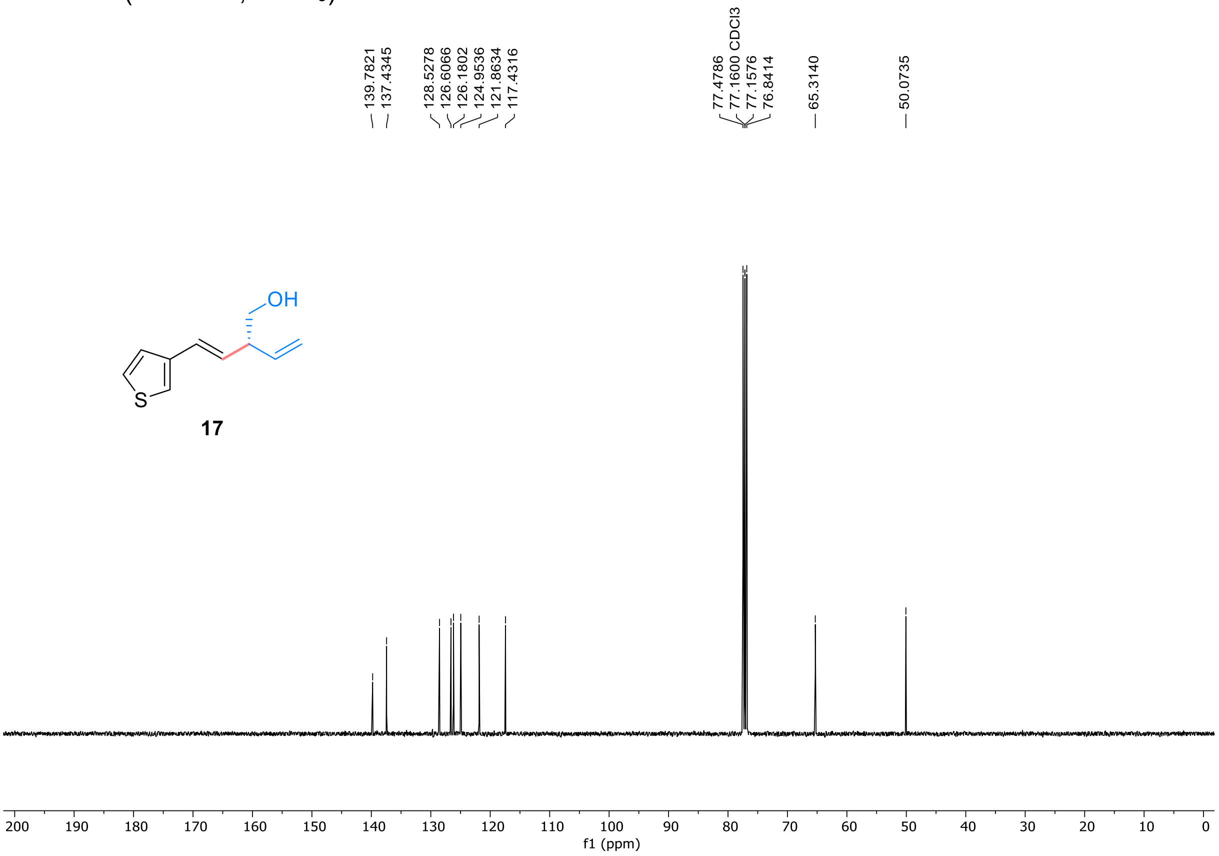

^{1}H NMR (400 MHz, $CDCl_3$) of **18** (*see NMR data*)

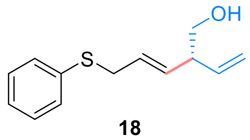

18

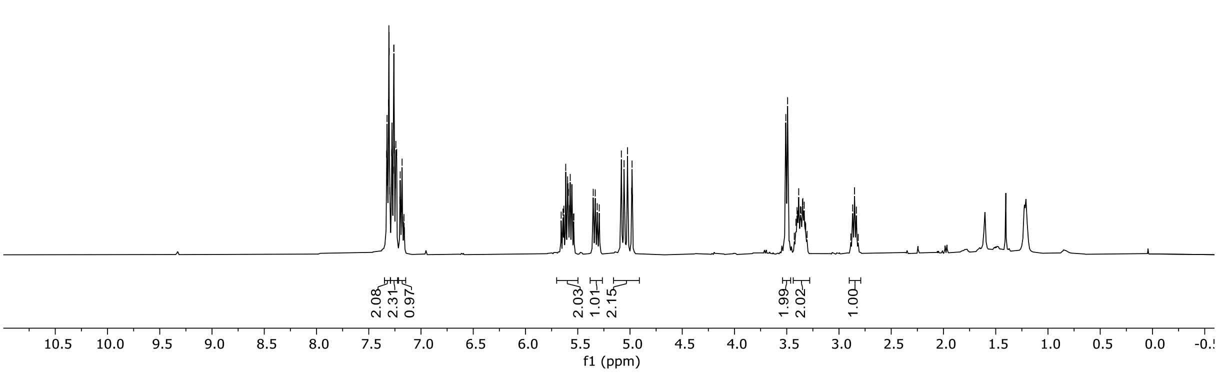

^{13}C NMR (101 MHz, $CDCl_3$) of **18**

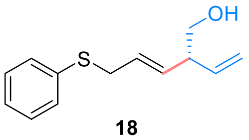

18

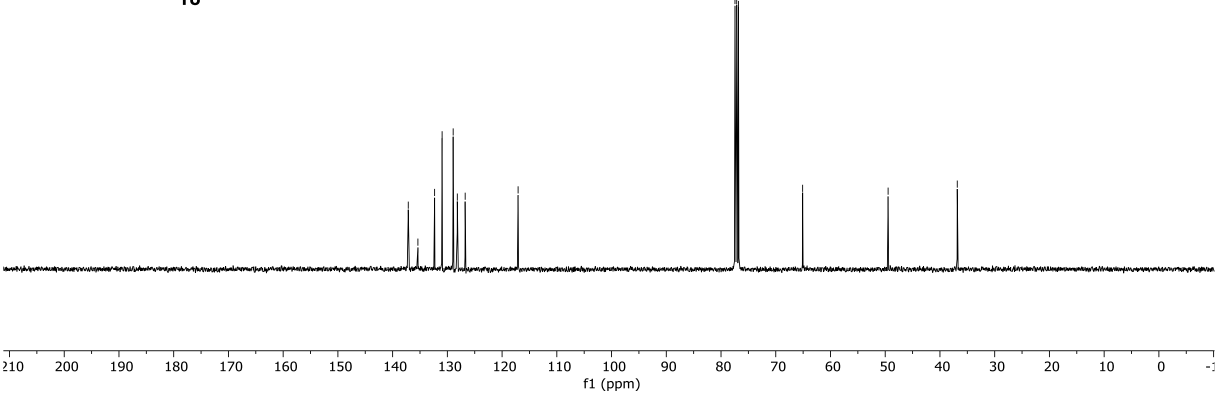

^{1}H NMR (400 MHz, $CDCl_3$) of **19** (*see NMR data*)

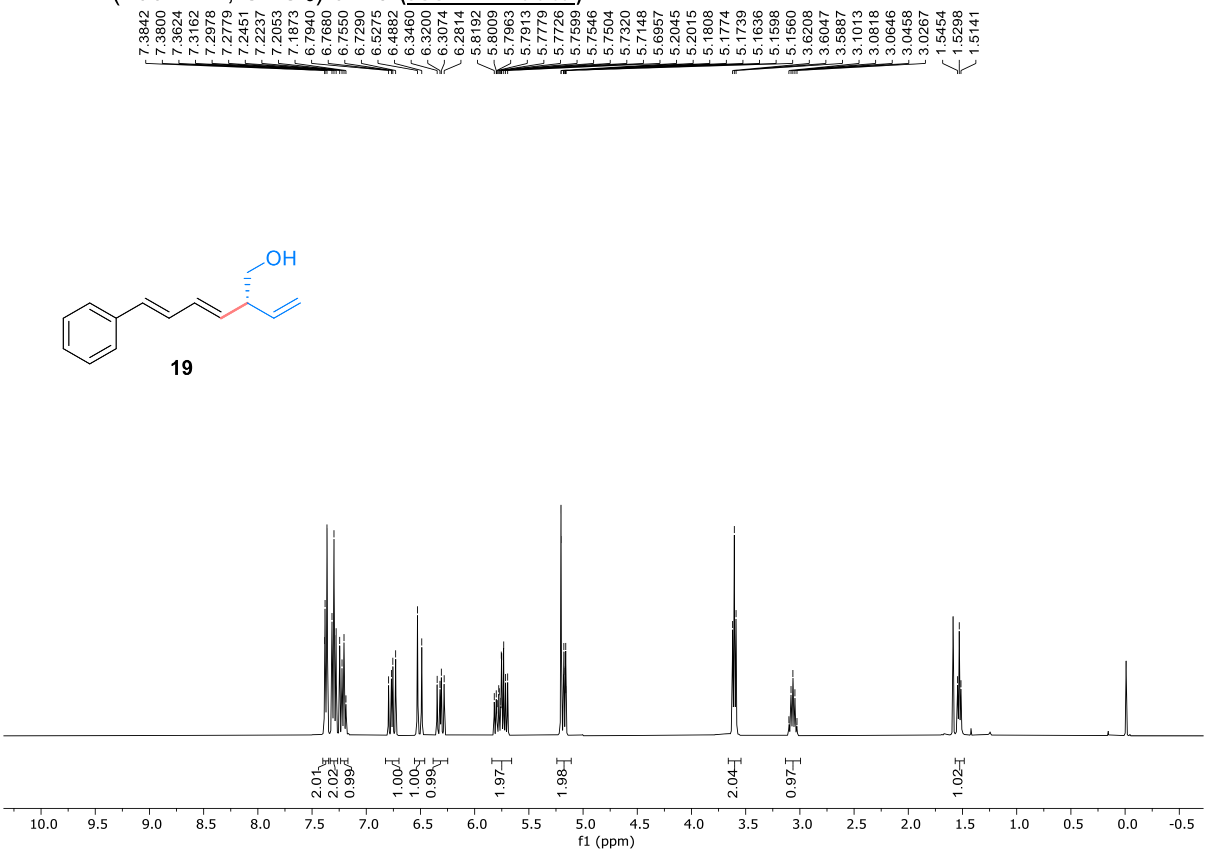

^{13}C NMR (101 MHz, $CDCl_3$) of **19**

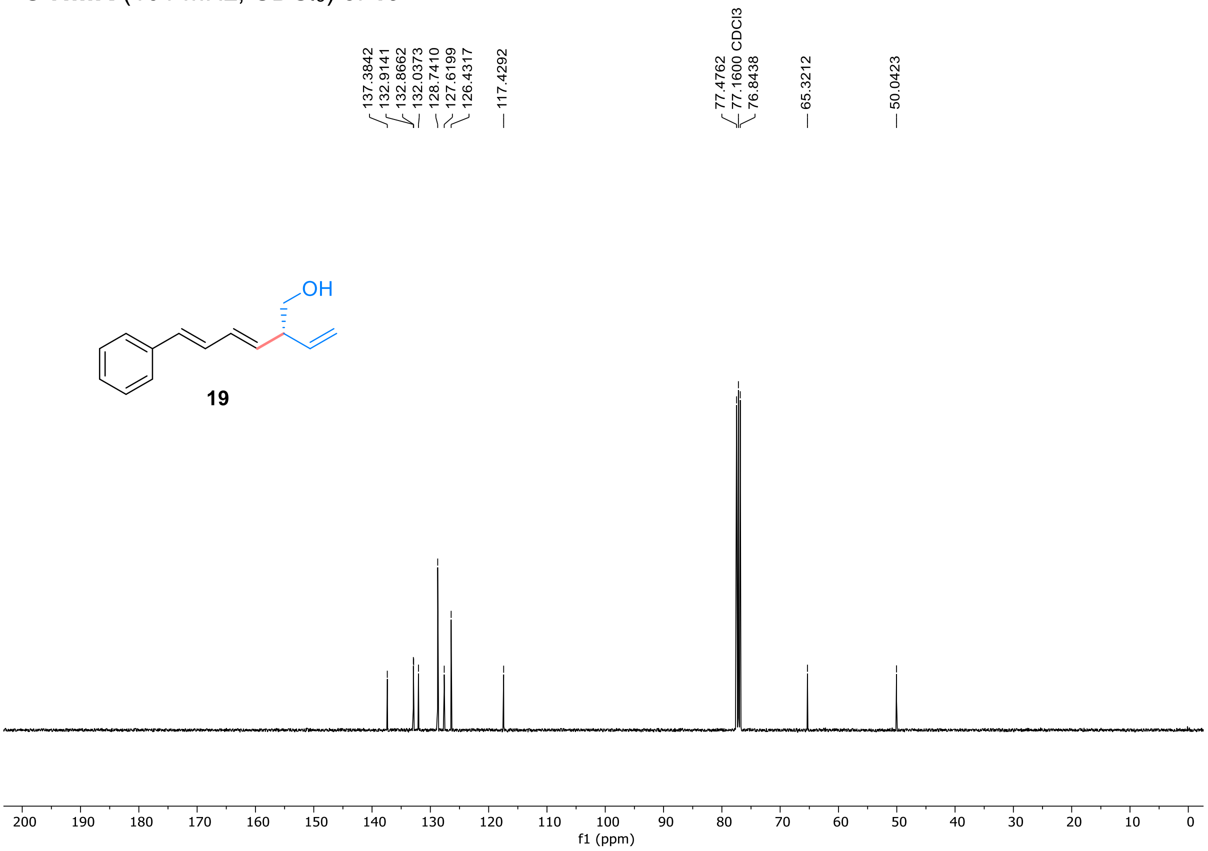

^{1}H NMR (400 MHz, $CDCl_3$) of **20** (*see NMR data*)

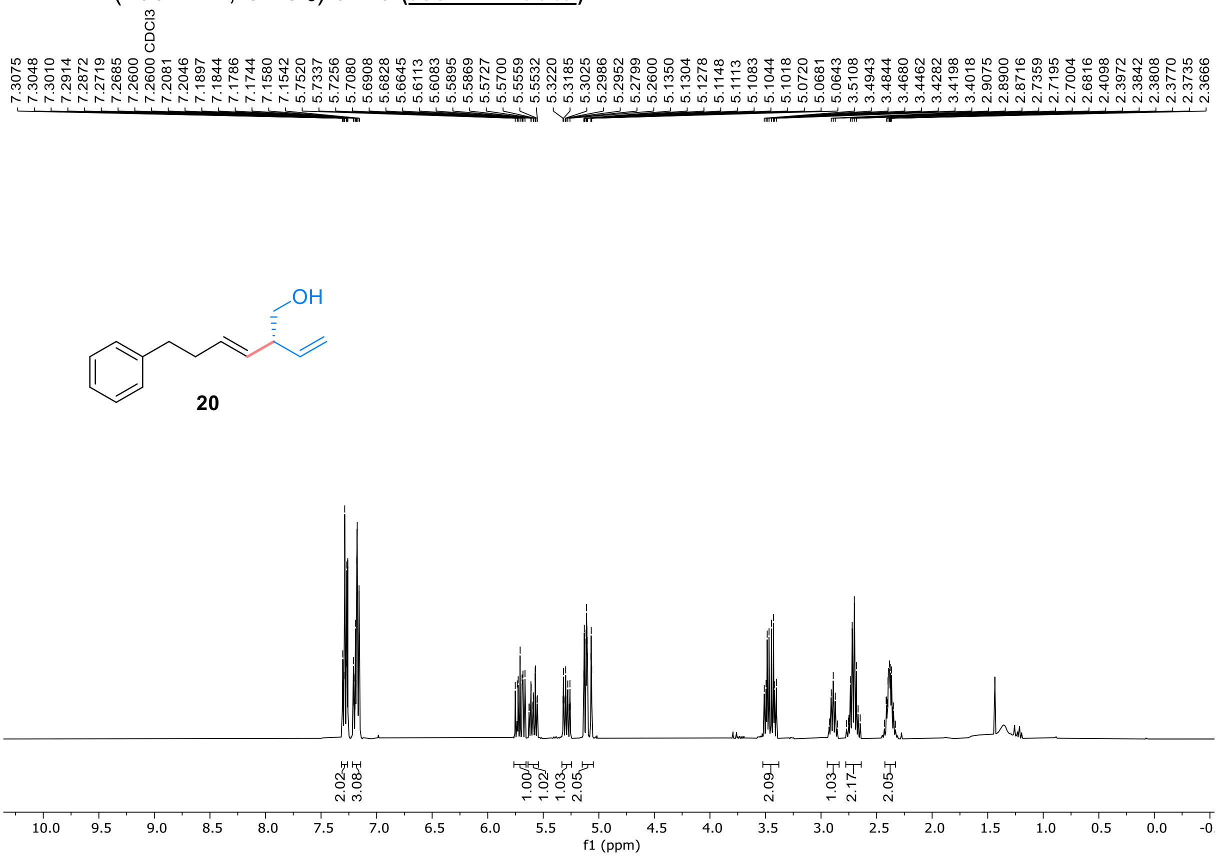

^{13}C NMR (101 MHz, $CDCl_3$) of **20**

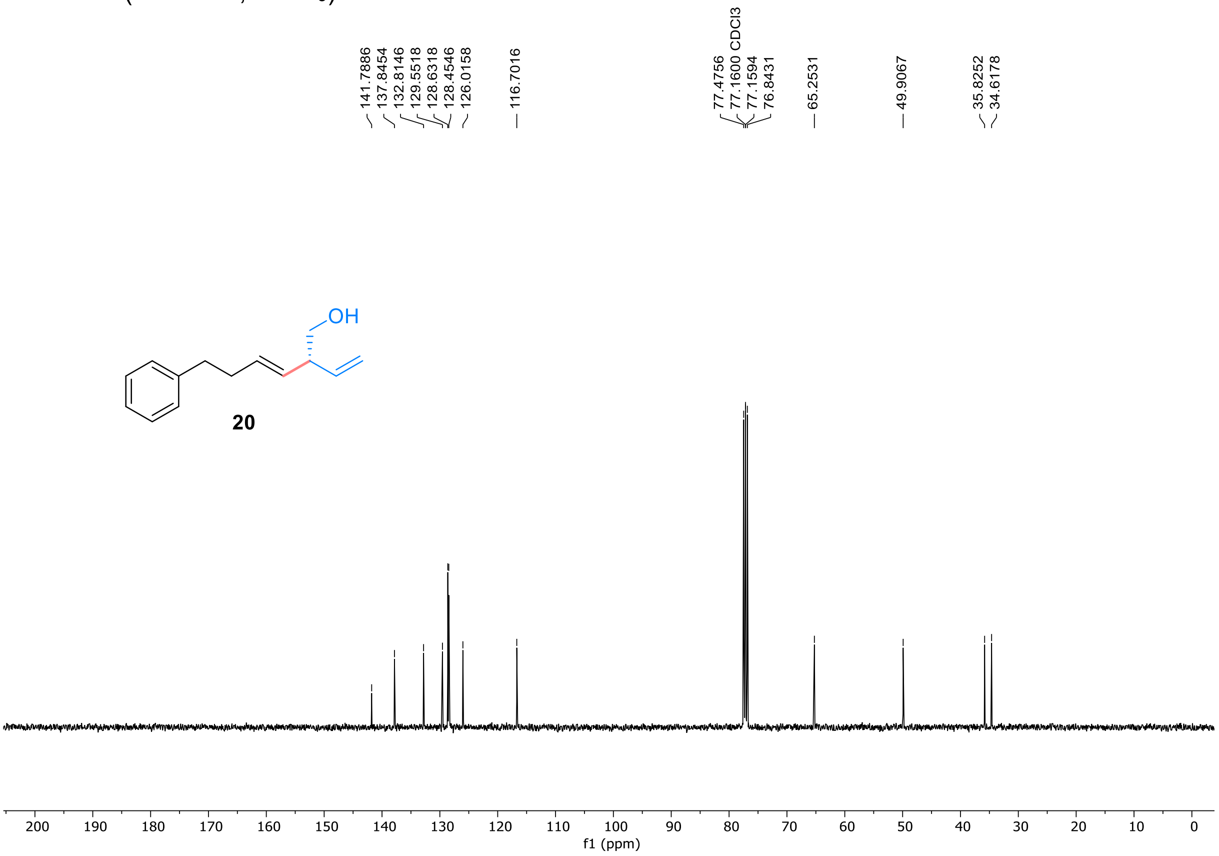

¹H NMR (400 MHz, CDCl₃) of **21** (*see NMR data*)

21

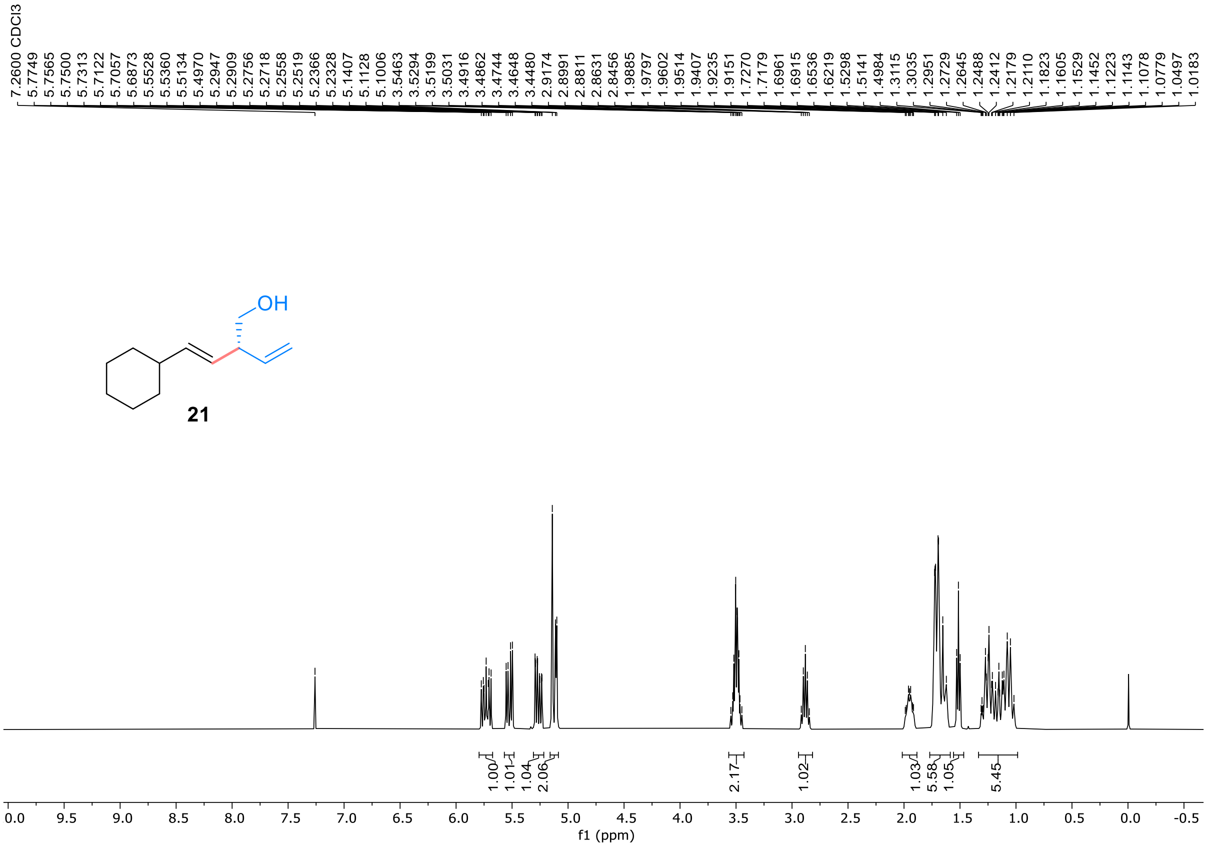

¹³C NMR (101 MHz, CDCl₃) of **21**

21

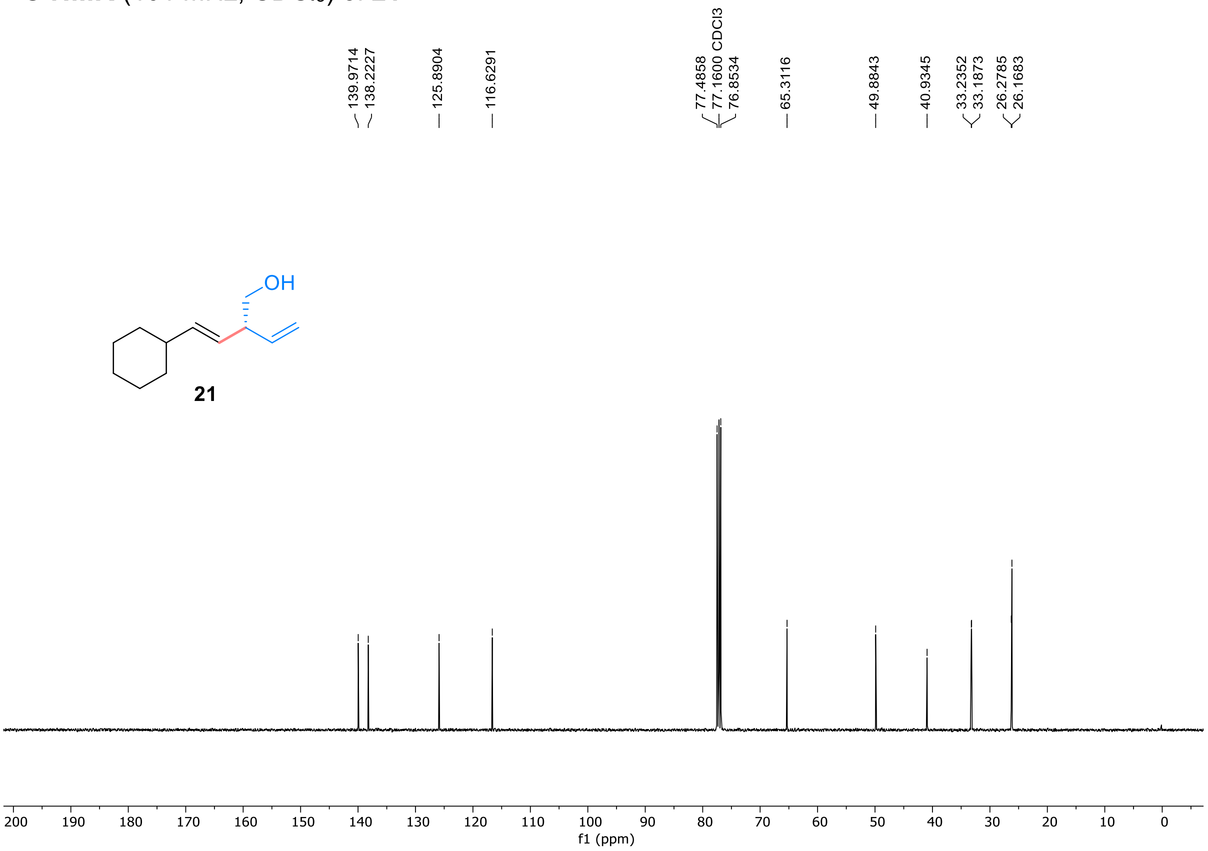

^{1}H NMR (400 MHz, $CDCl_3$) of **22** (*see NMR data*)

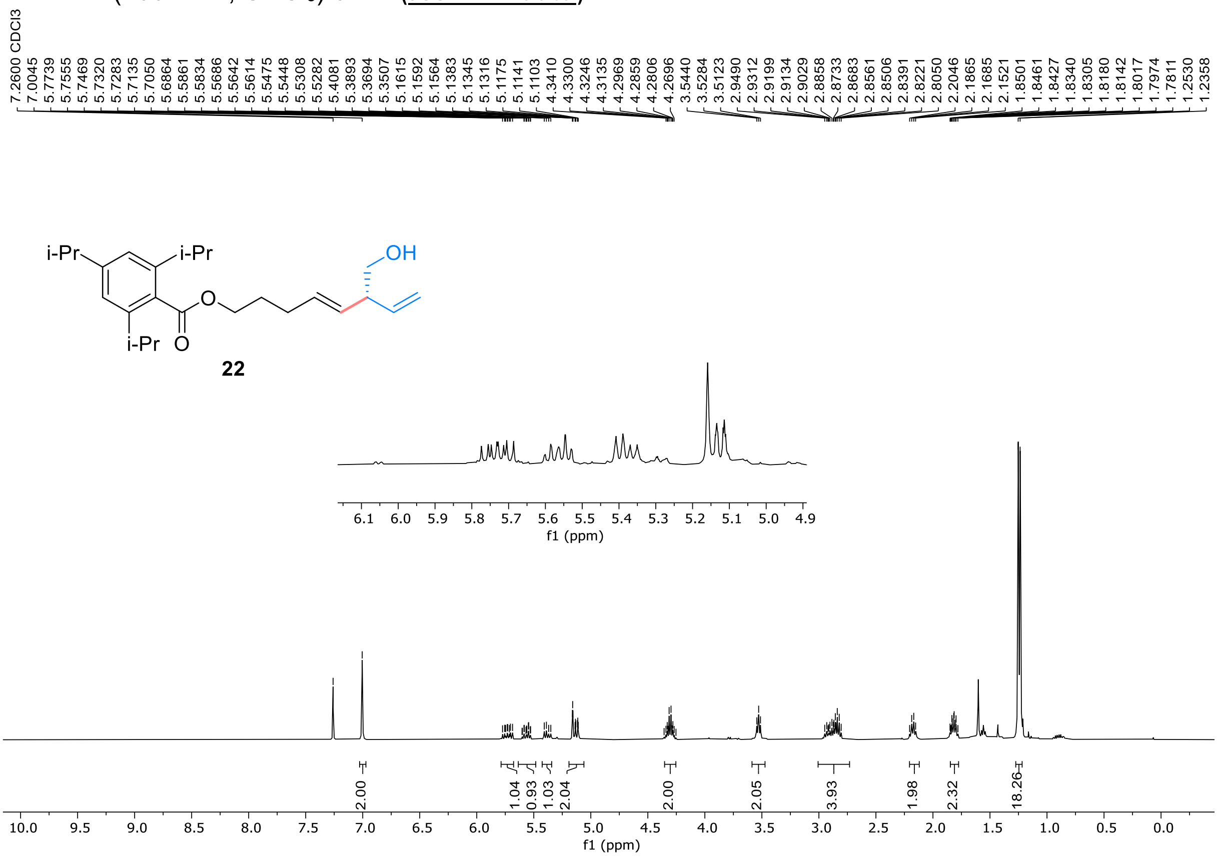

^{13}C NMR (101 MHz, $CDCl_3$) of **22**

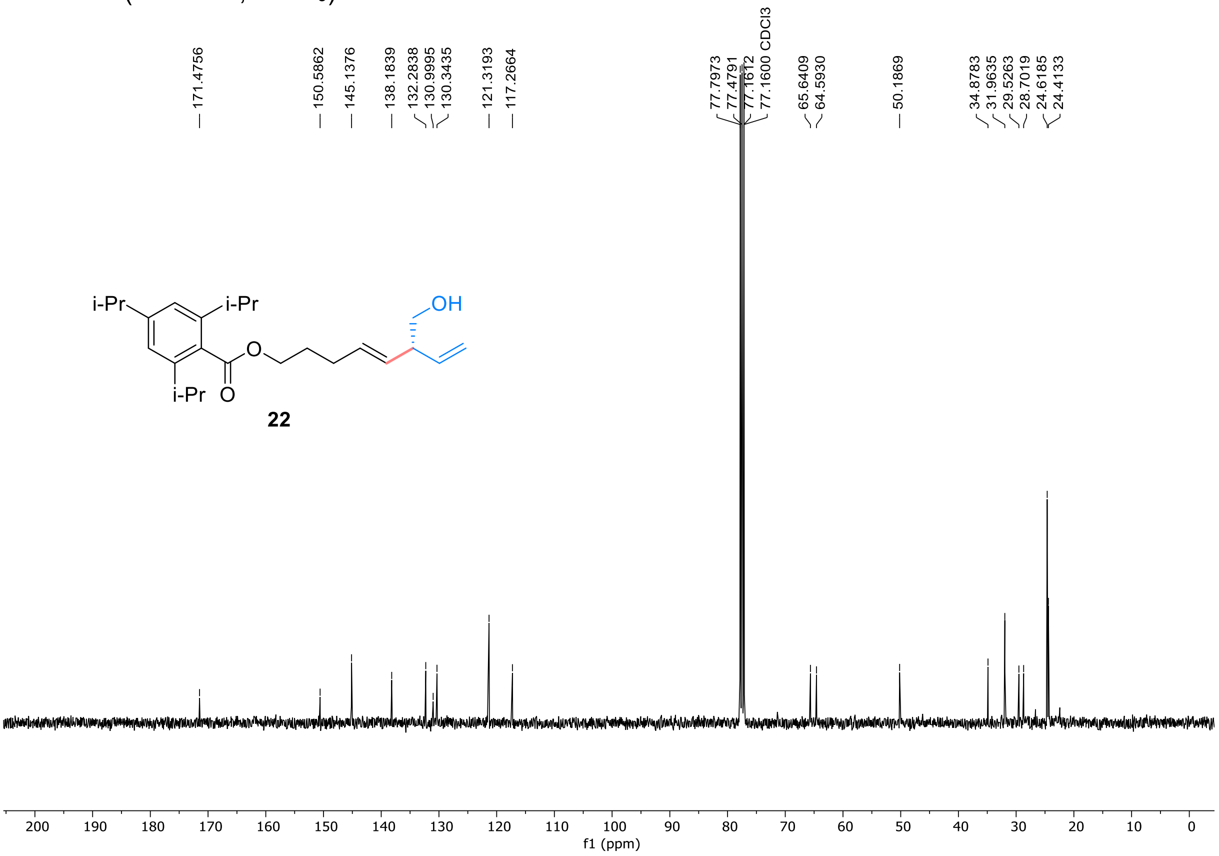

¹H NMR (400 MHz, CDCl₃) of **23** (*see NMR data*)

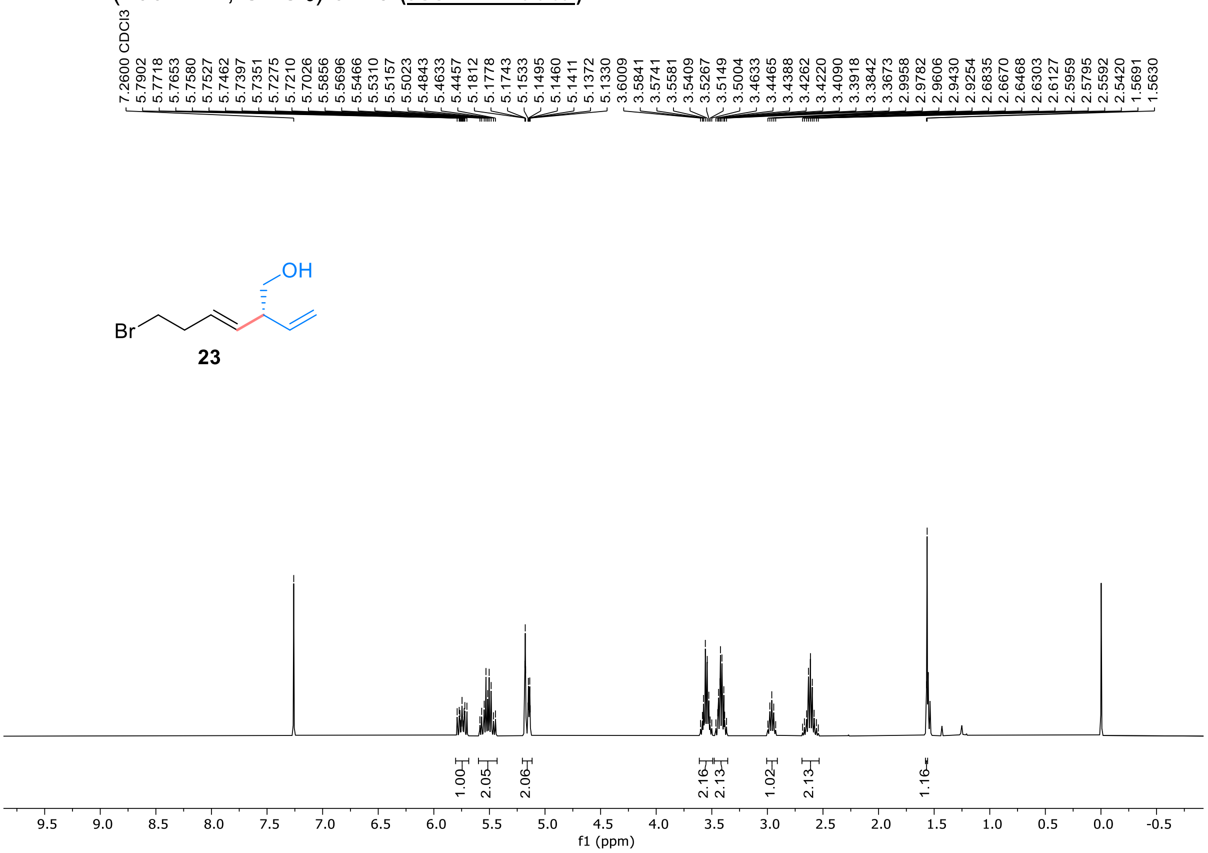

¹³C NMR (101 MHz, CDCl₃) of **23**

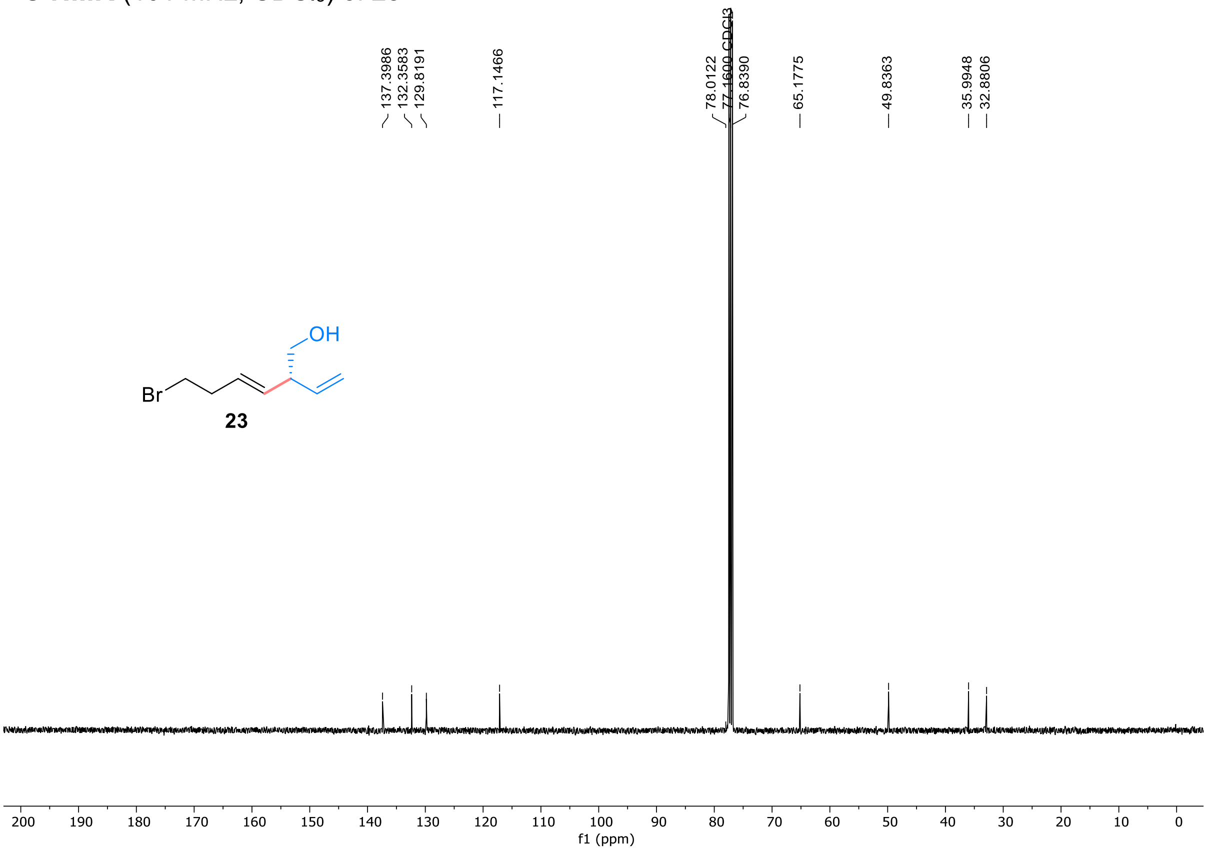

^{1}H NMR (400 MHz, $CDCl_3$) of **24** (*see NMR data*)

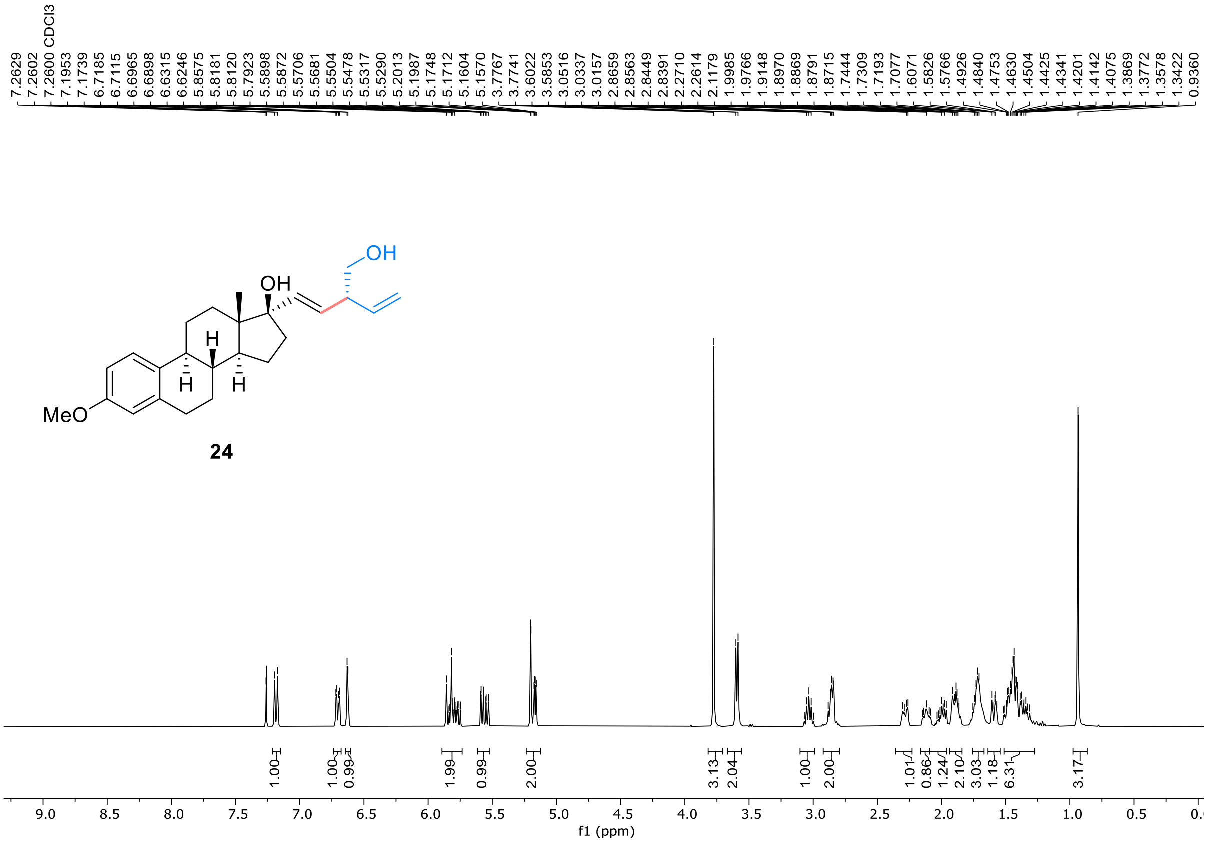

^{13}C NMR (101 MHz, $CDCl_3$) of **24**

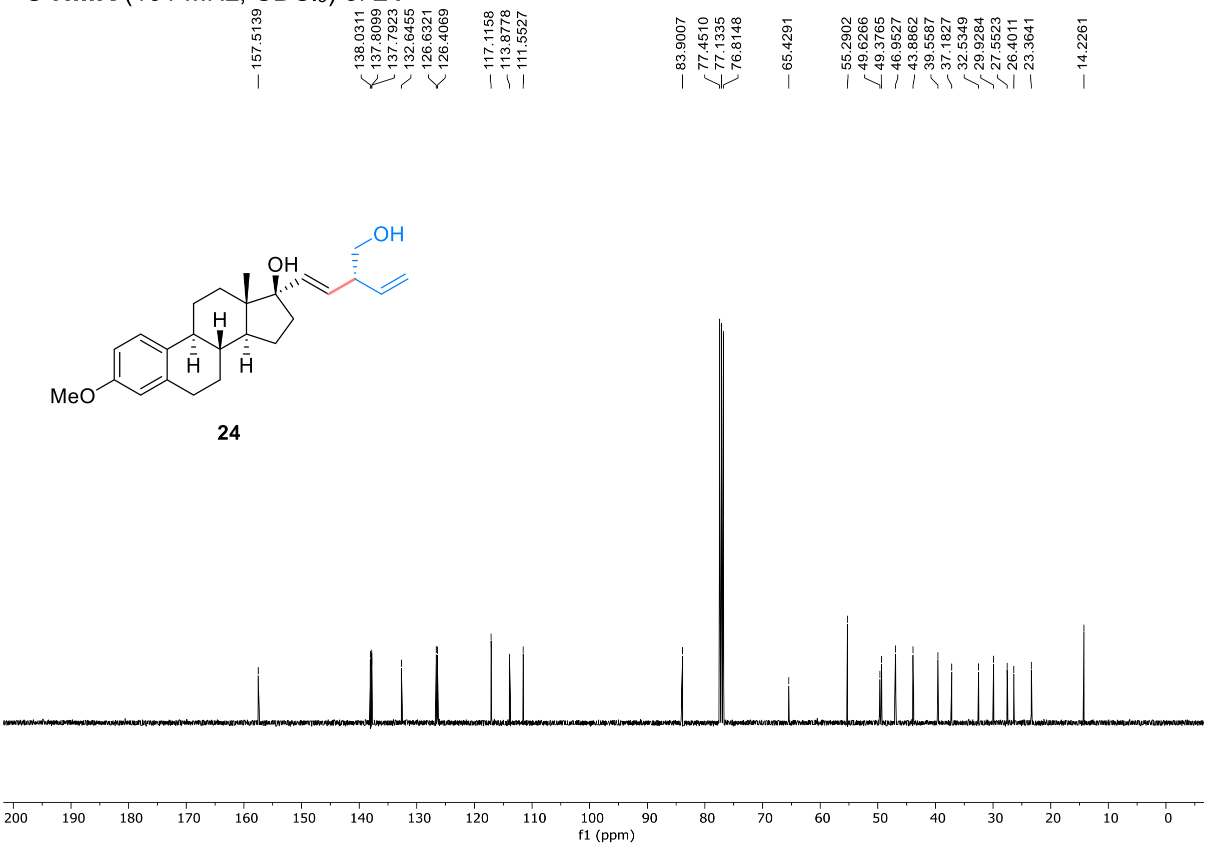

^{1}H NMR (400 MHz, $CDCl_3$) of **25** (*see NMR data*)

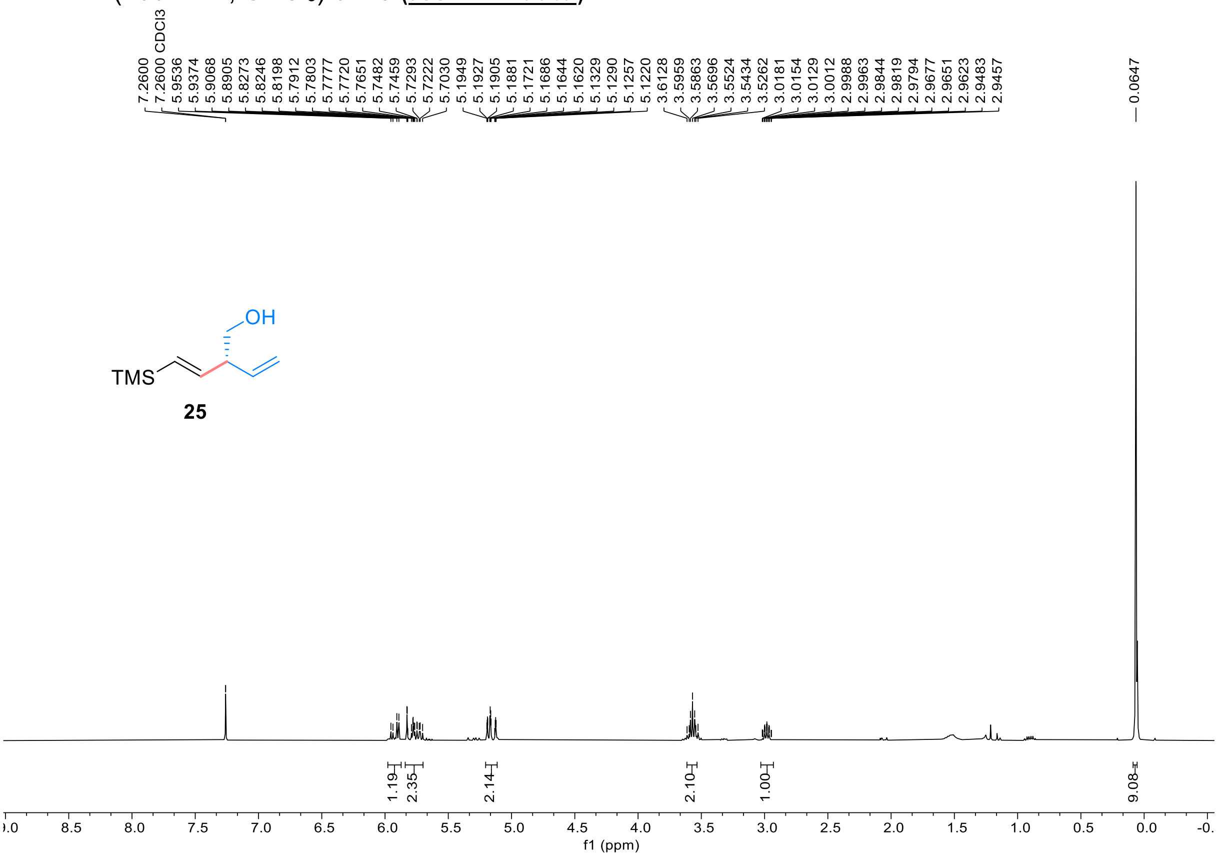

^{13}C NMR (101 MHz, $CDCl_3$) of **25**

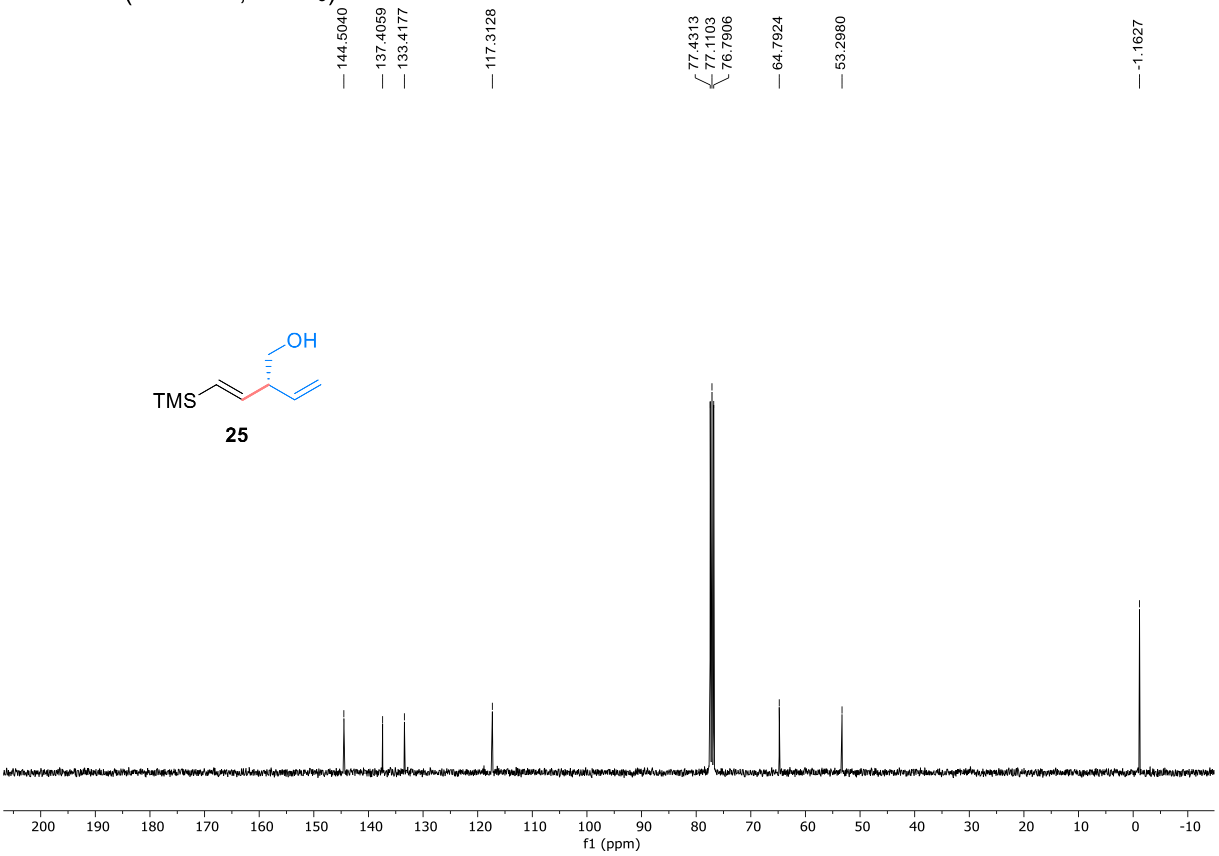

^{1}H NMR (400 MHz, $CDCl_3$) of **26** (*see NMR data*)

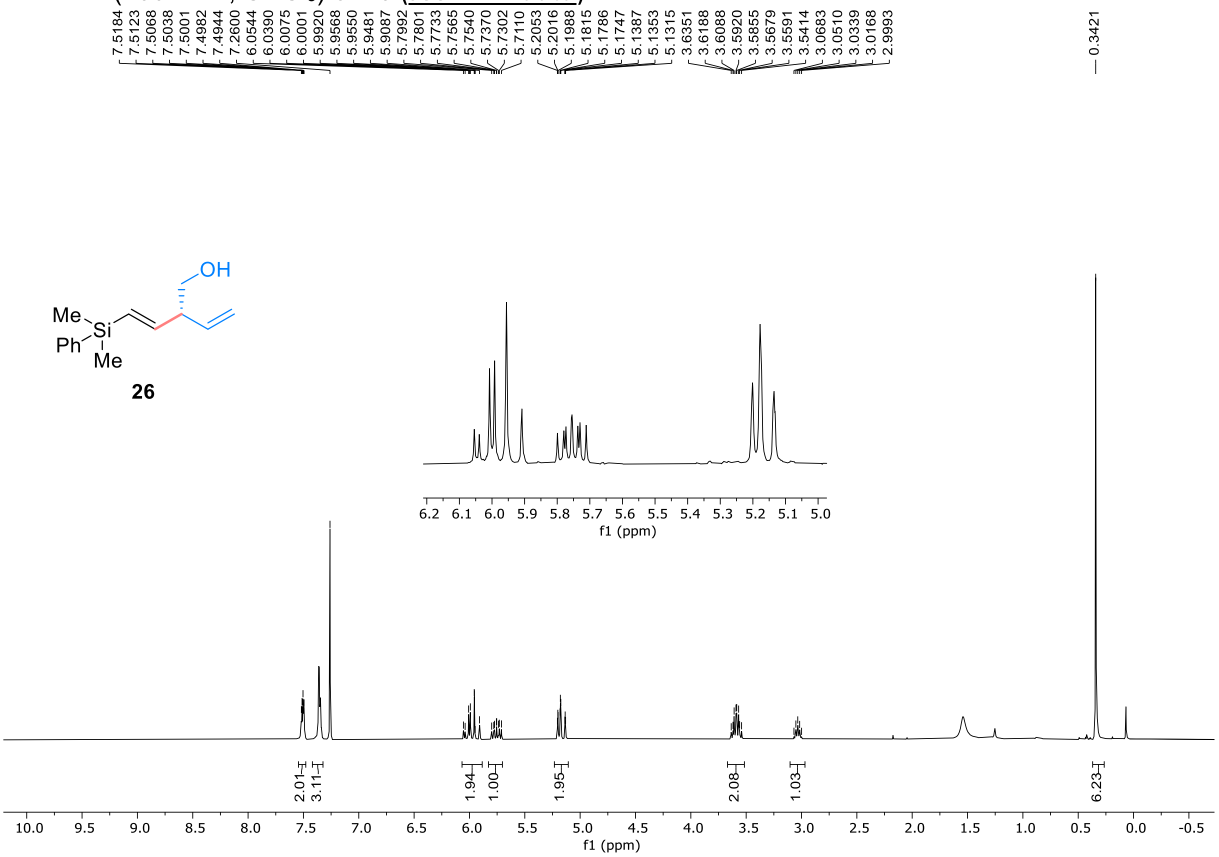

^{13}C NMR (101 MHz, $CDCl_3$) of **26**

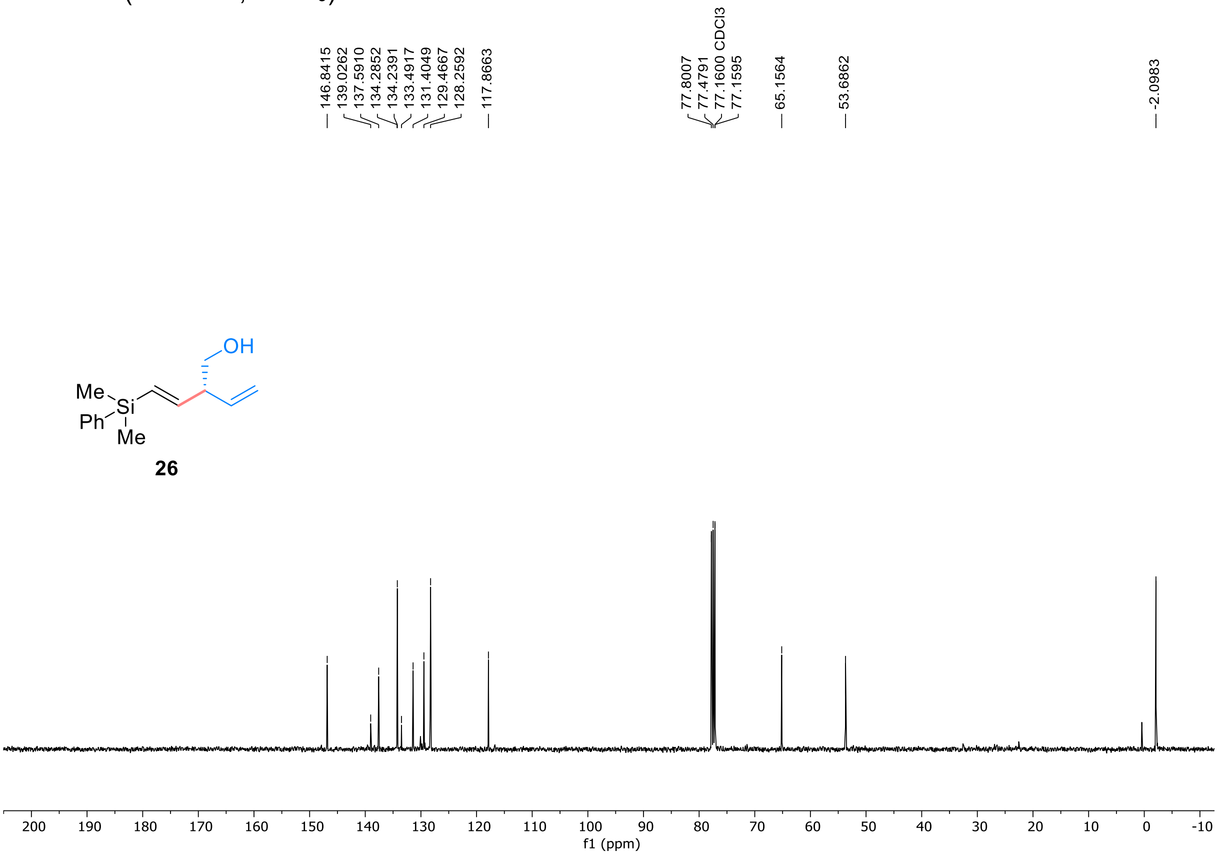

^{1}H NMR (400 MHz, $CDCl_3$) of **27** (*see NMR data*)

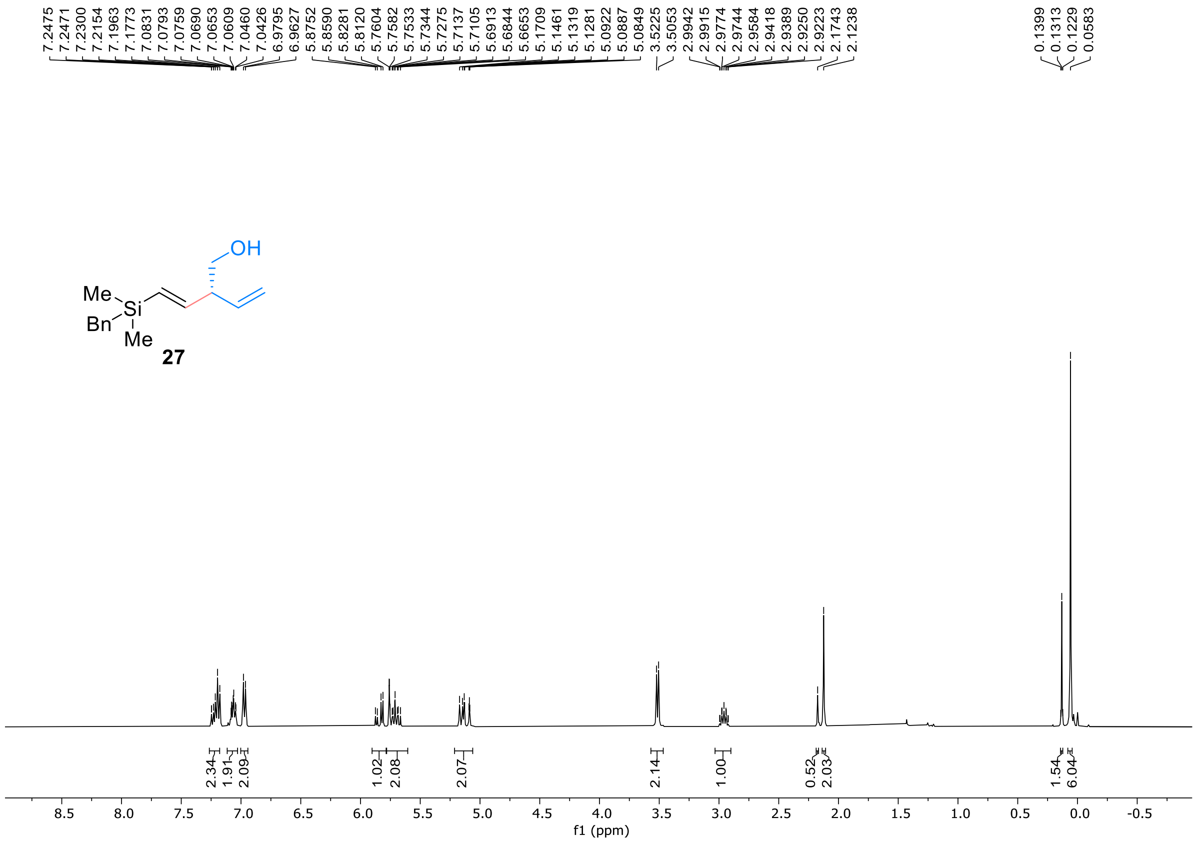

^{13}C NMR (101 MHz, $CDCl_3$) of **27**

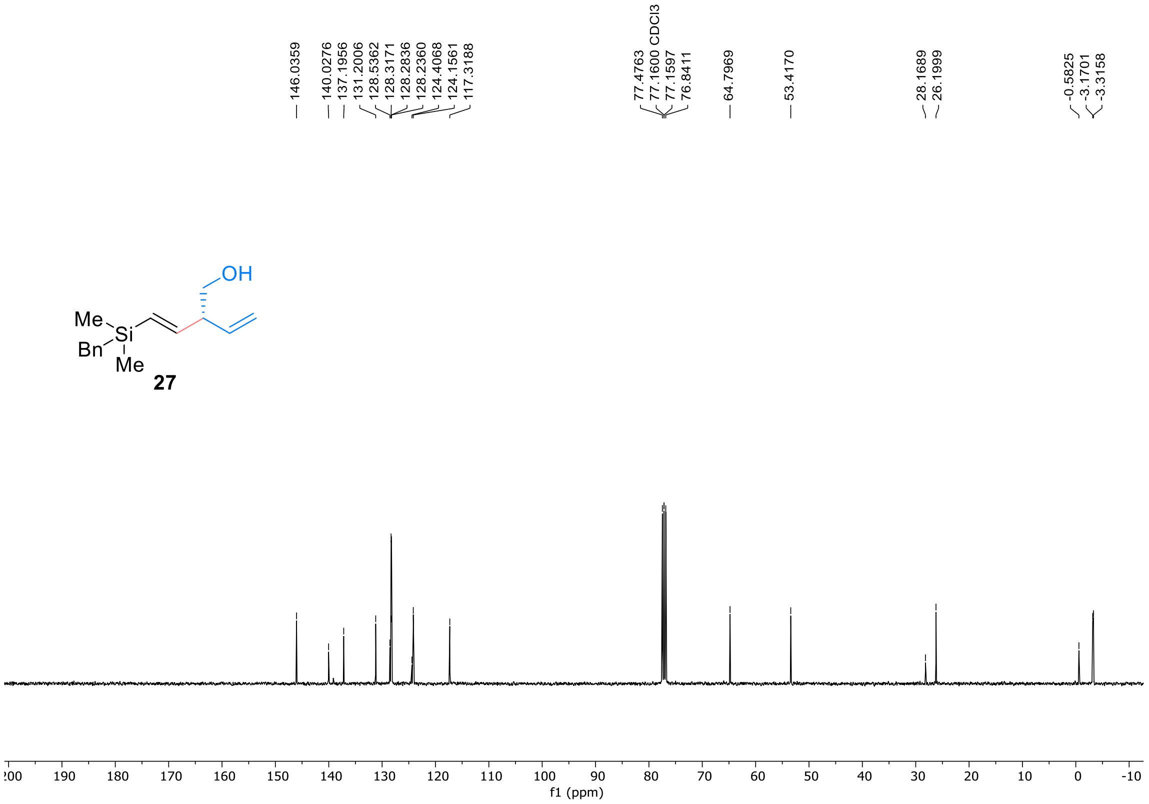

^{1}H NMR (400 MHz, $CDCl_3$) of **28** (*see NMR data*)

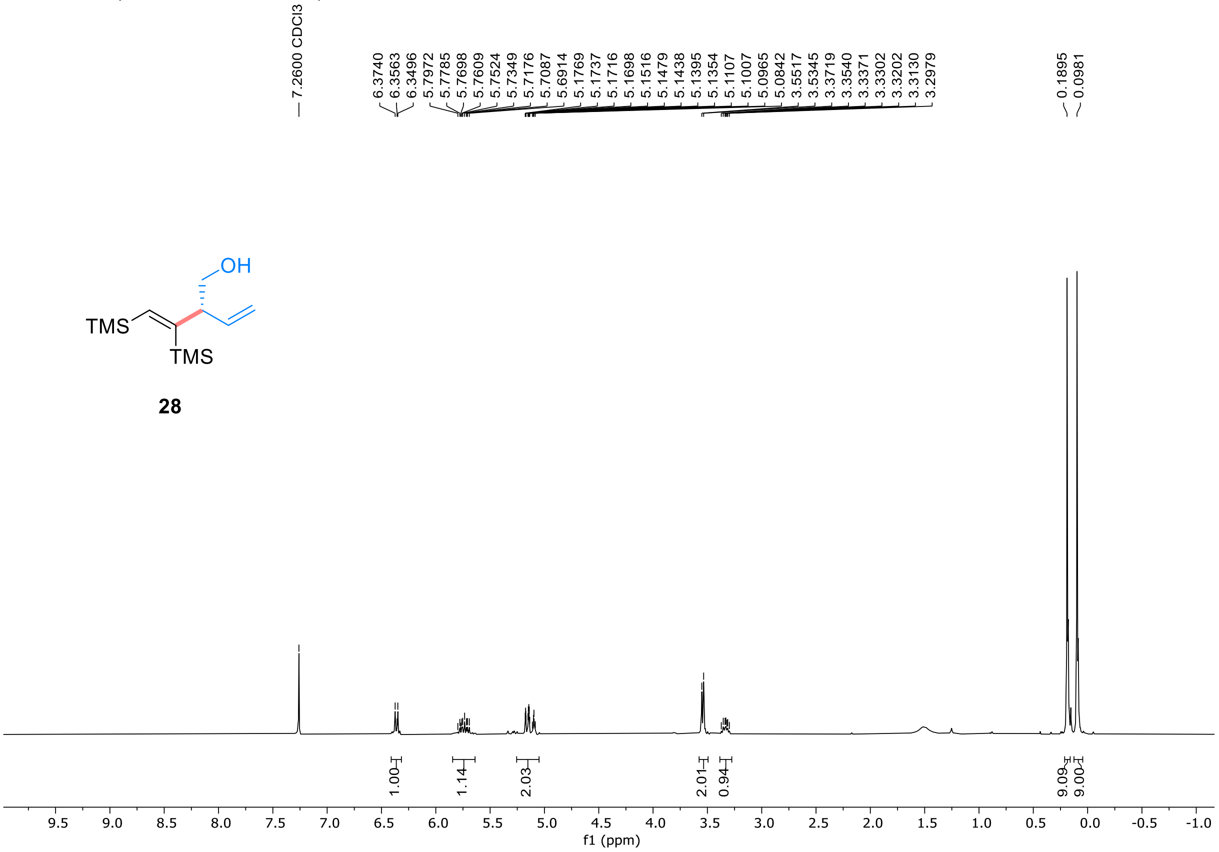

^{13}C NMR (101MHz, $CDCl_3$) of **28**

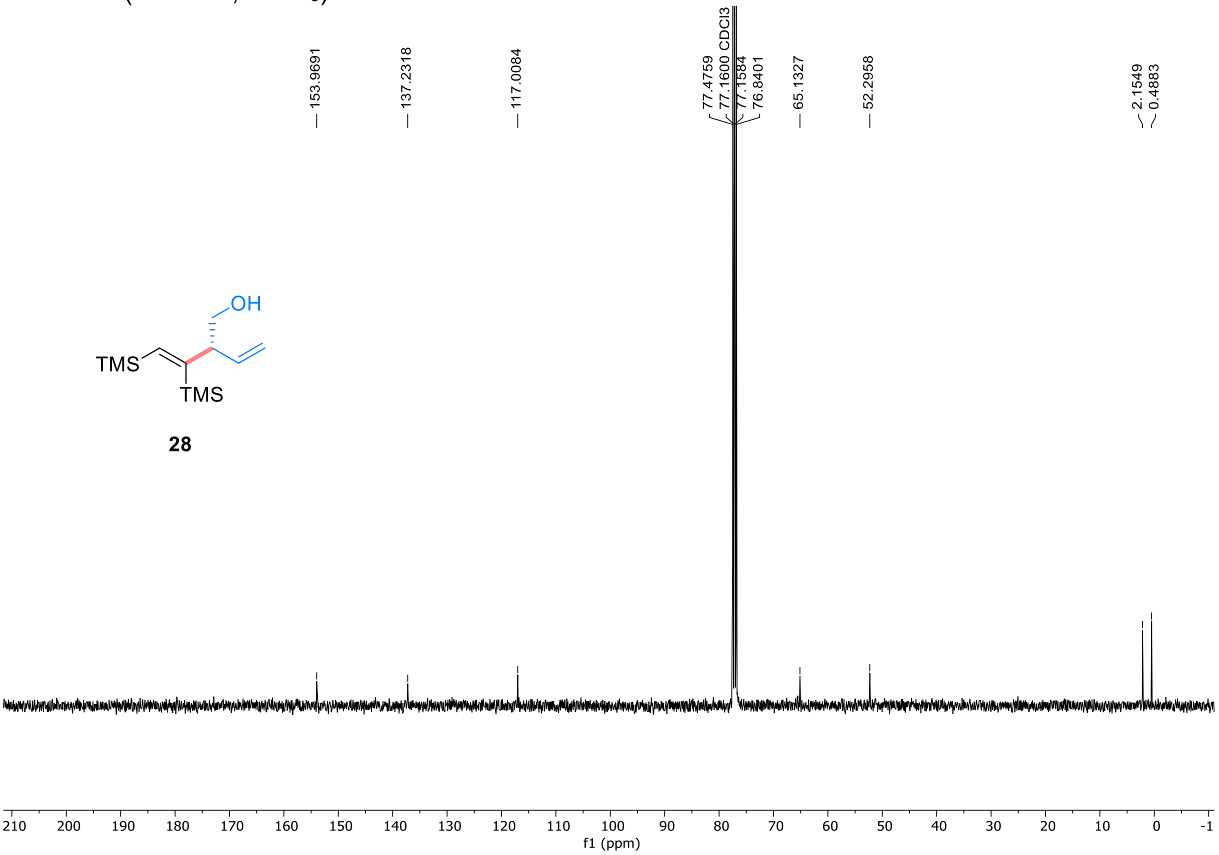

^{1}H NMR (400 MHz, $CDCl_3$) of **29a** (*see NMR data*)

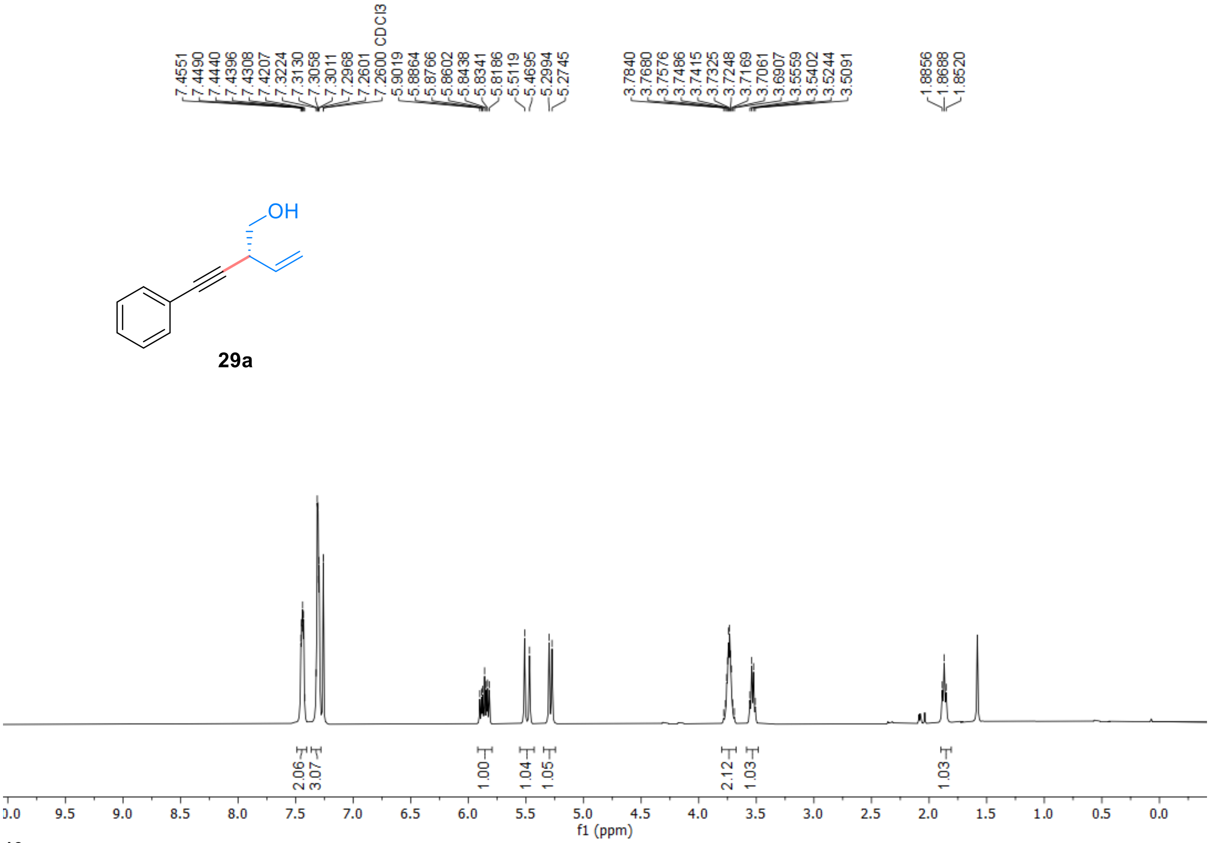

^{13}C NMR (101 MHz, $CDCl_3$) of **29a**

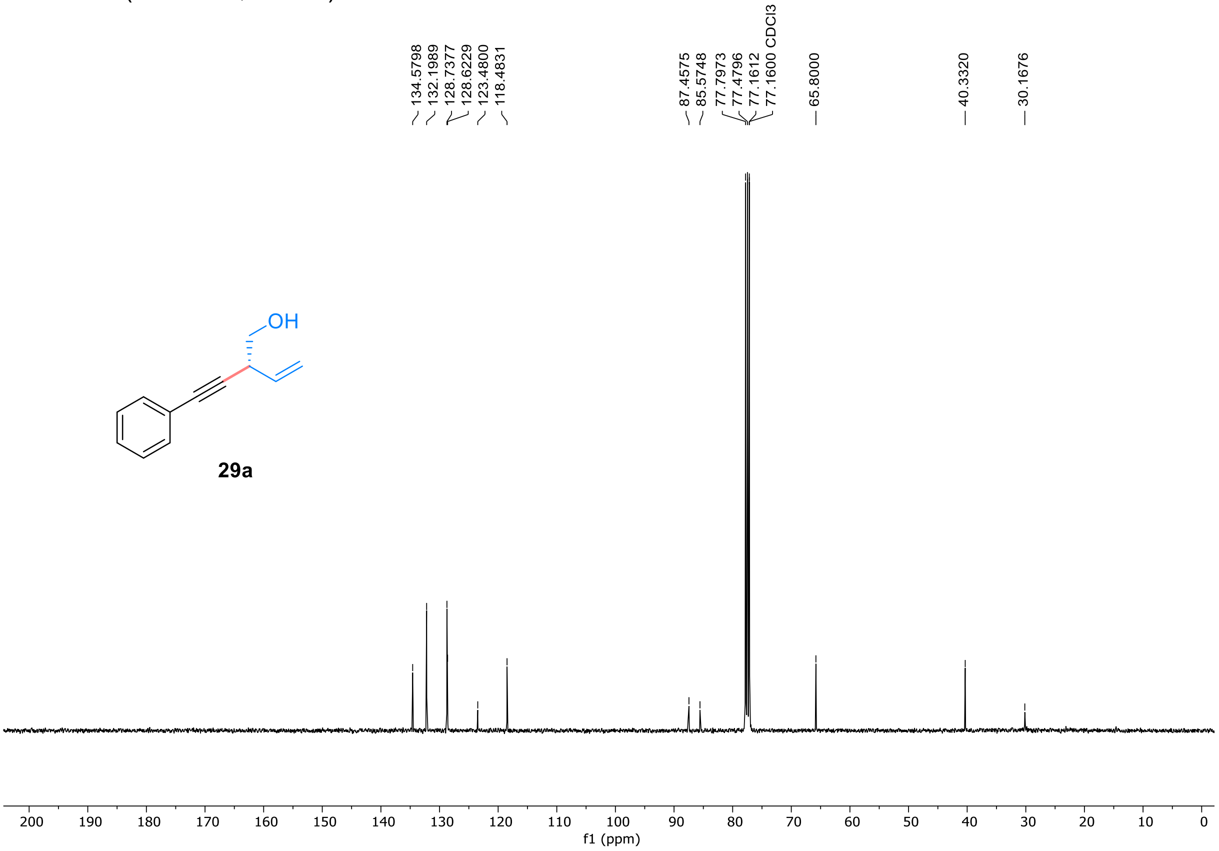

^{1}H NMR (500 MHz, $CDCl_3$) of **29b** (<u>*see NMR data*</u>)

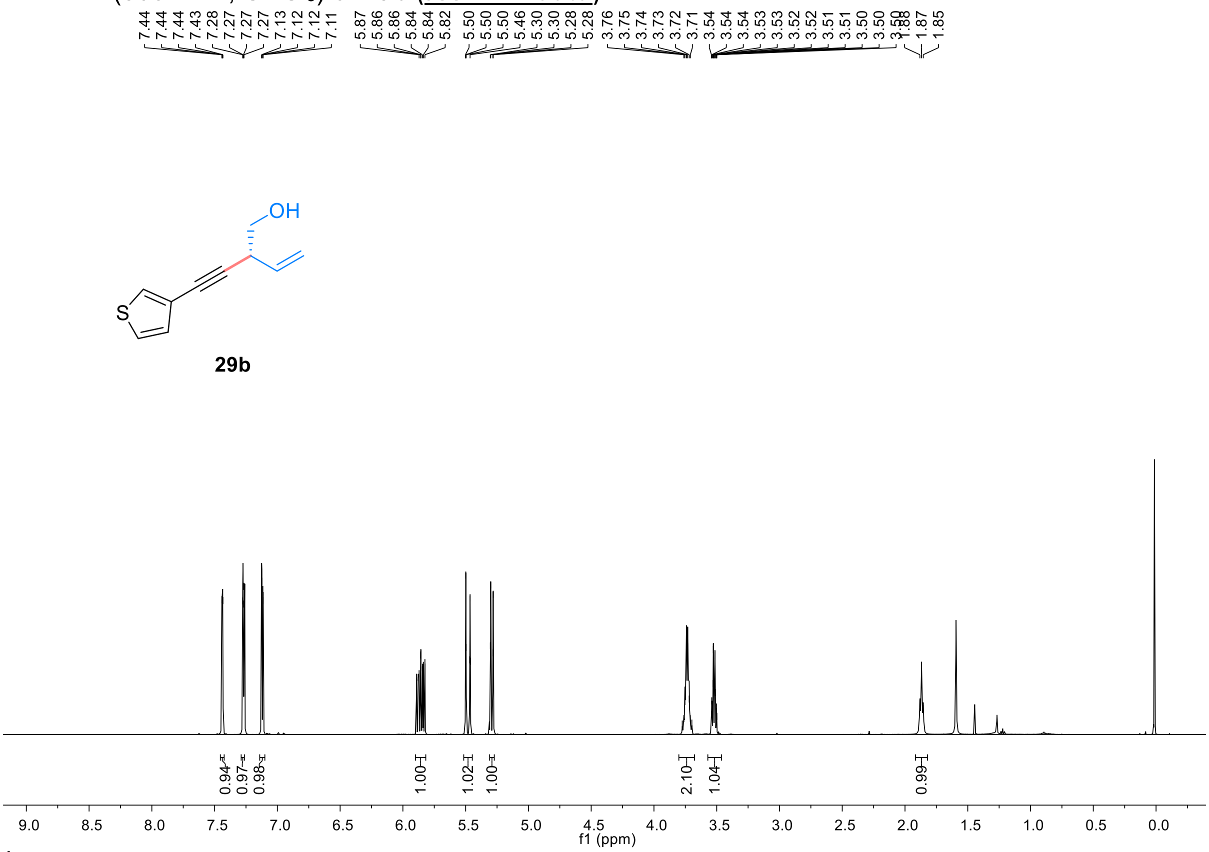

^{1}H NMR (126 MHz, $CDCl_3$) of **29b**

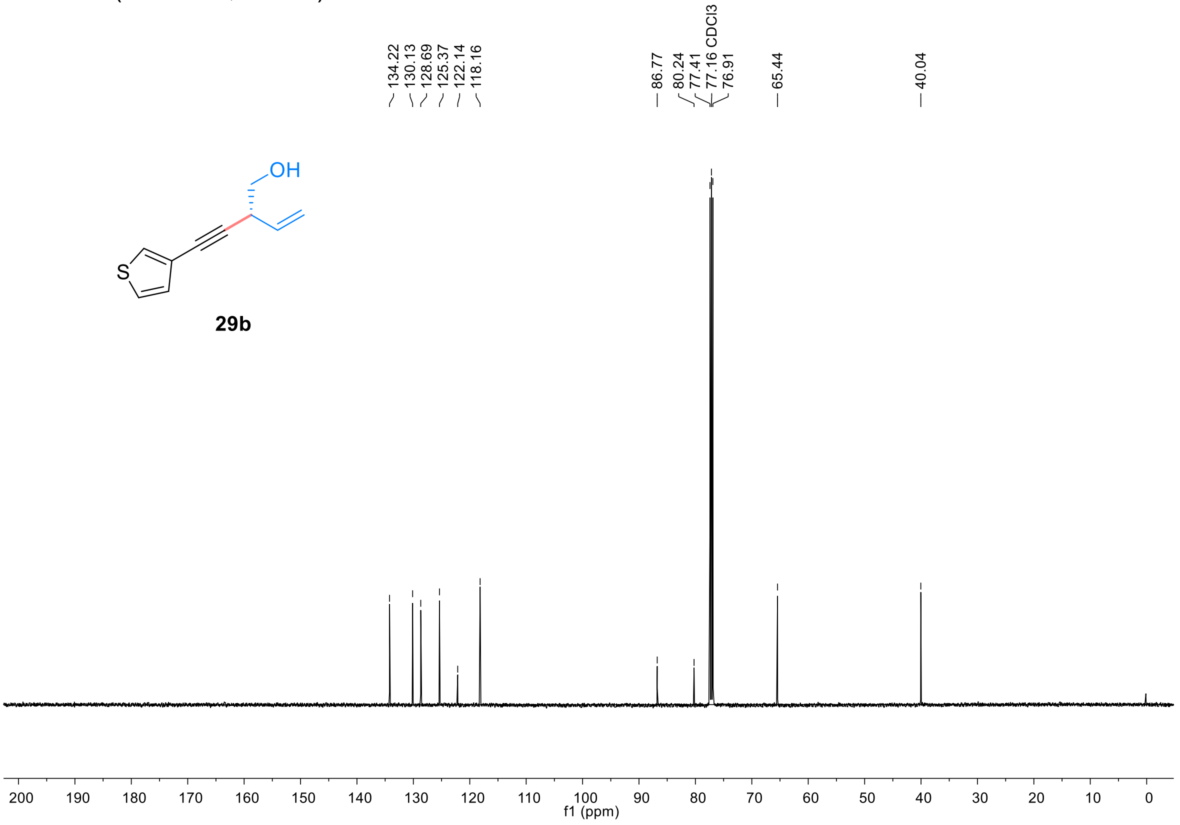

^{1}H NMR (500 MHz, $CDCl_3$) of **29c** (*see NMR data*)

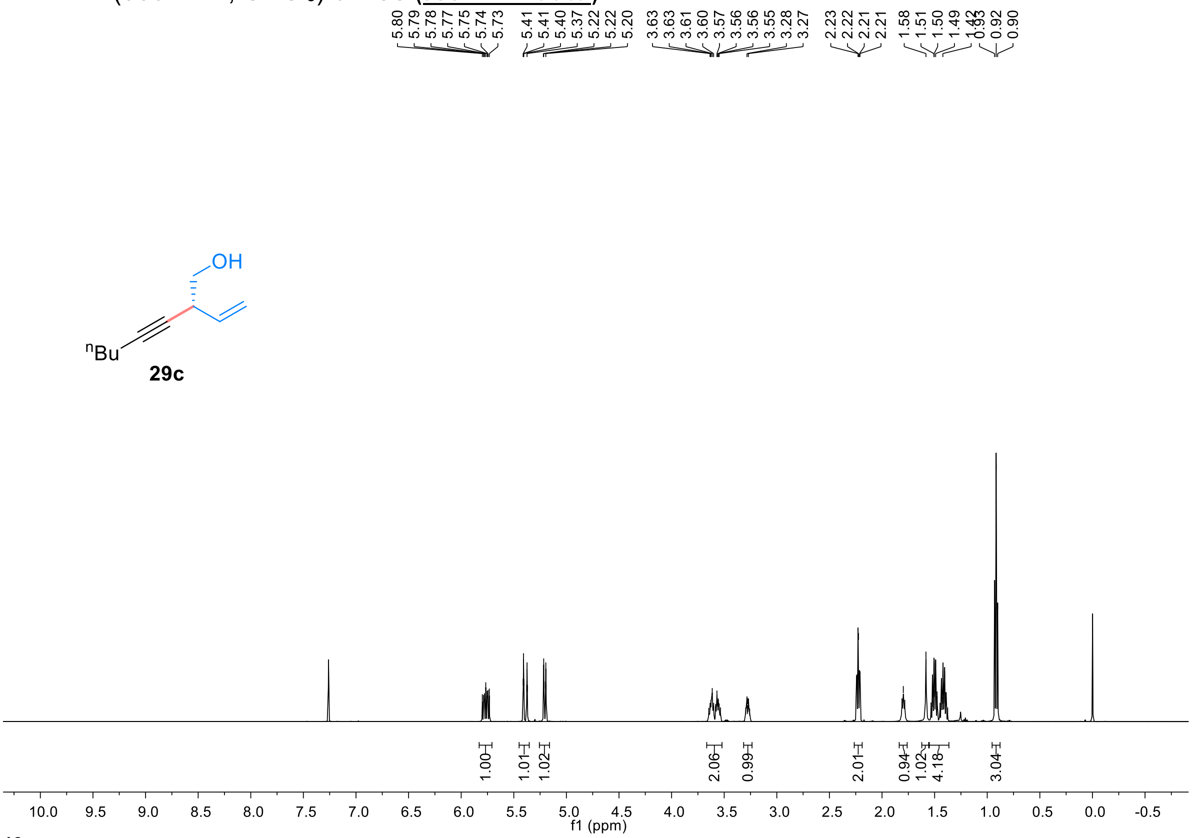

^{13}C NMR (126 MHz, $CDCl_3$) of **29c**

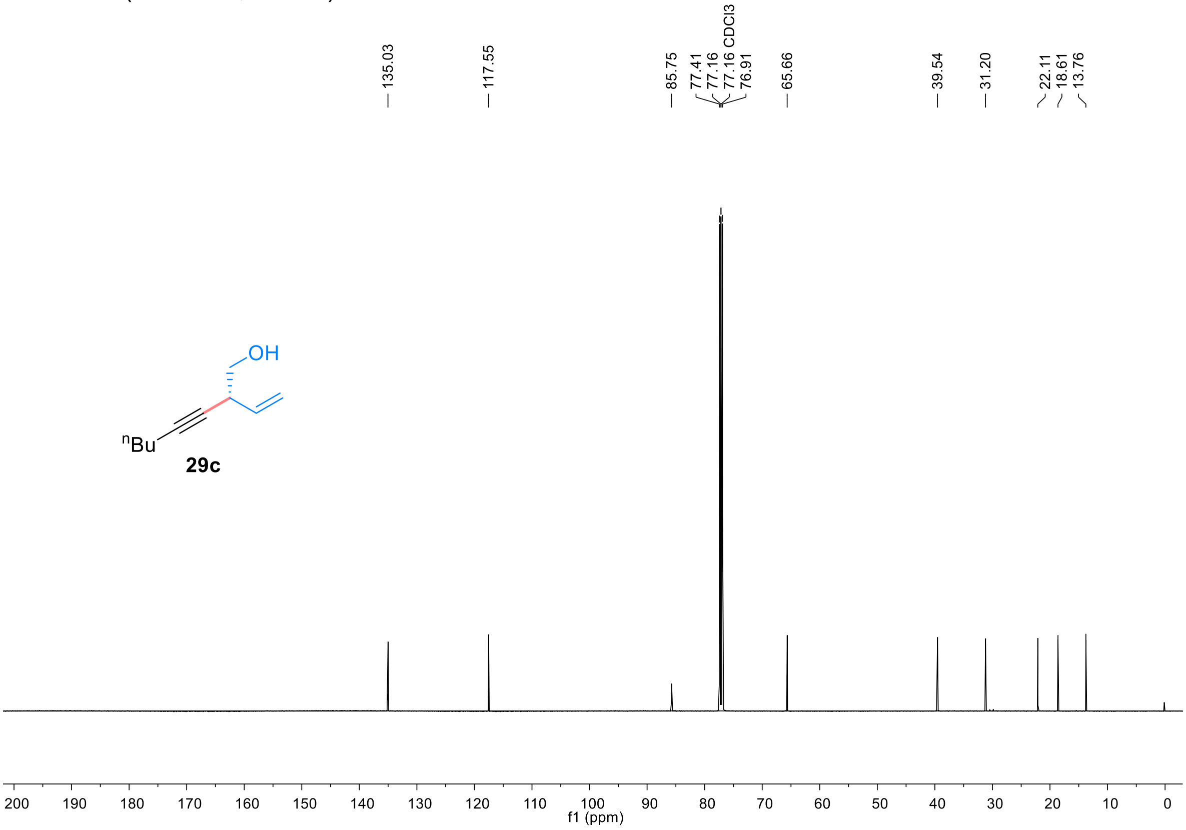

^{1}H NMR (400 MHz, $CDCl_3$) of **30** (*see NMR data*)

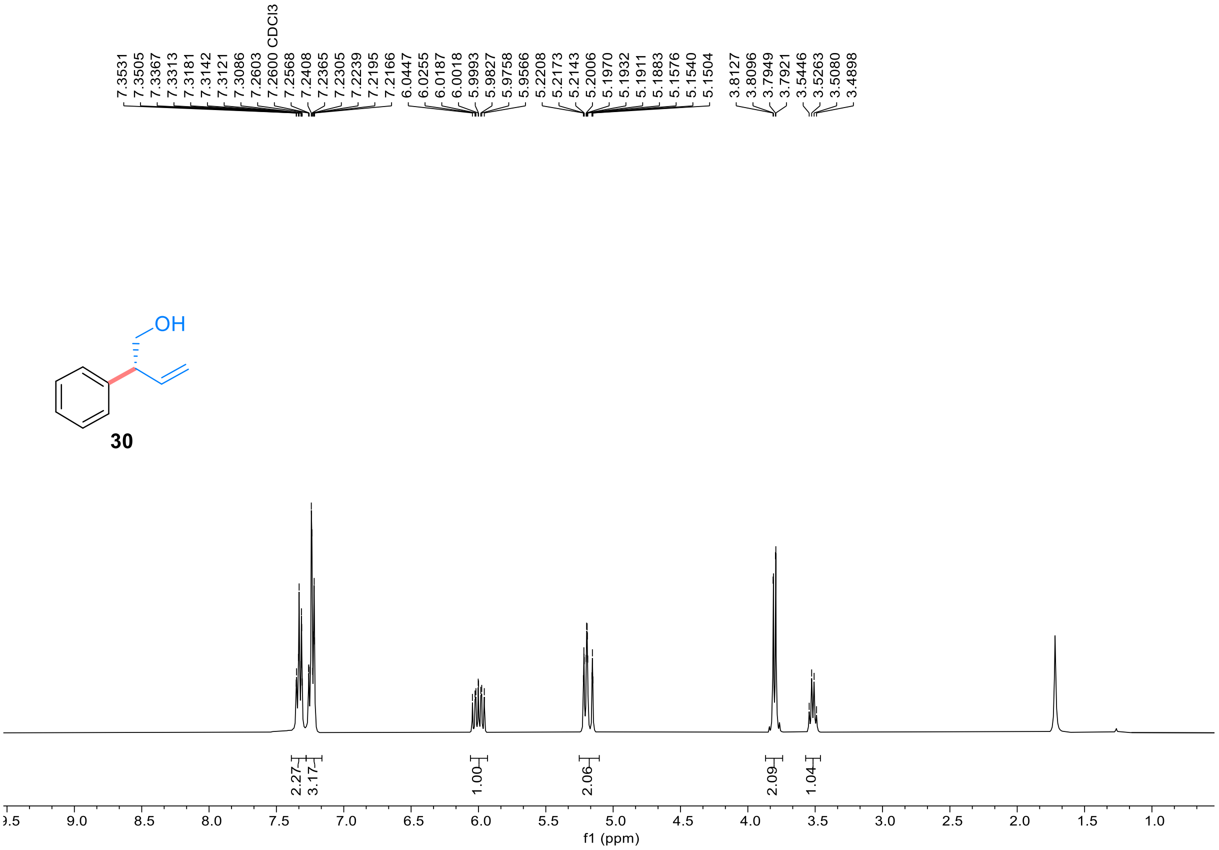

^{13}C NMR (101 MHz, $CDCl_3$) of **30**

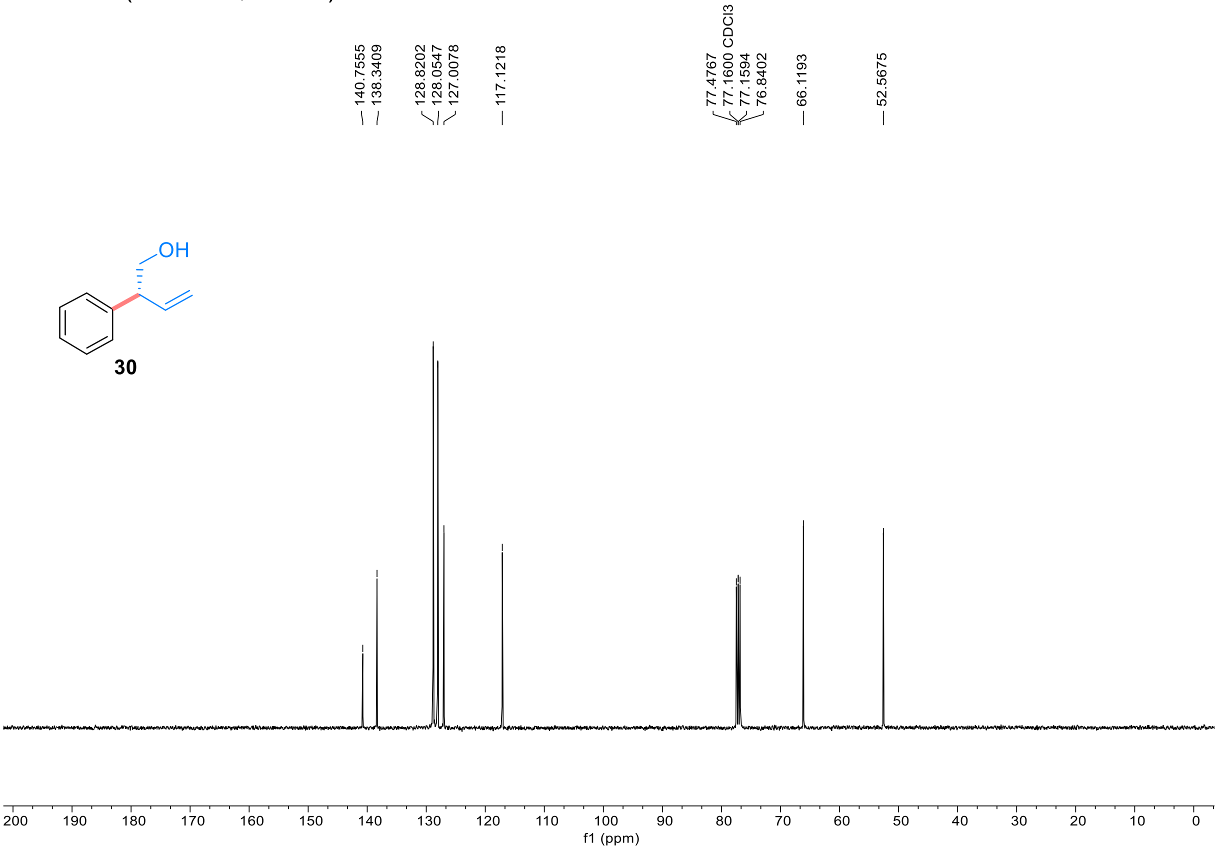

^{1}H NMR (400 MHz, $CDCl_3$) of **31** (*see NMR data*)

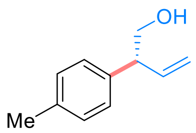

31

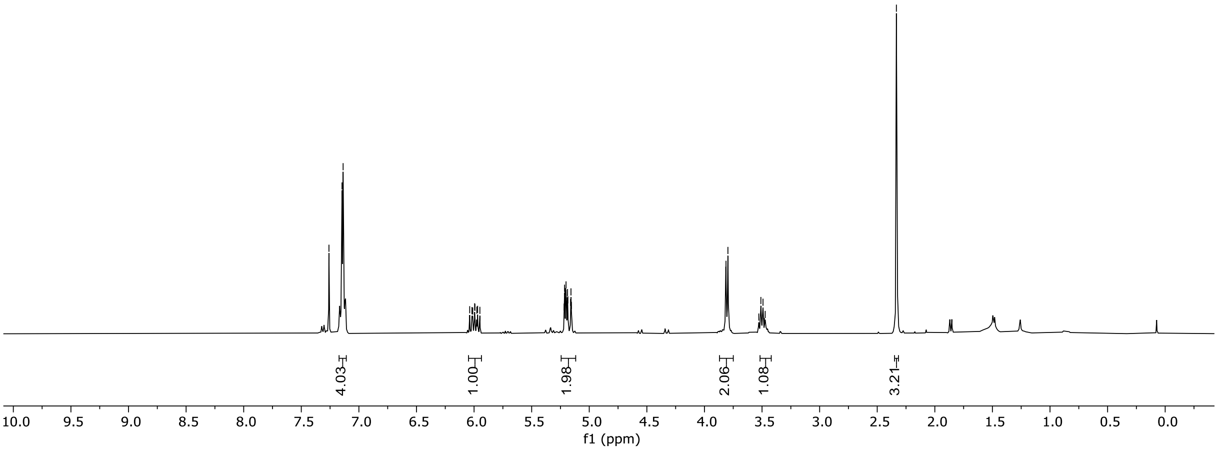

^{13}C NMR (101 MHz, $CDCl_3$) of **31**

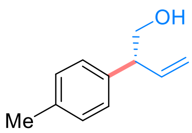

31

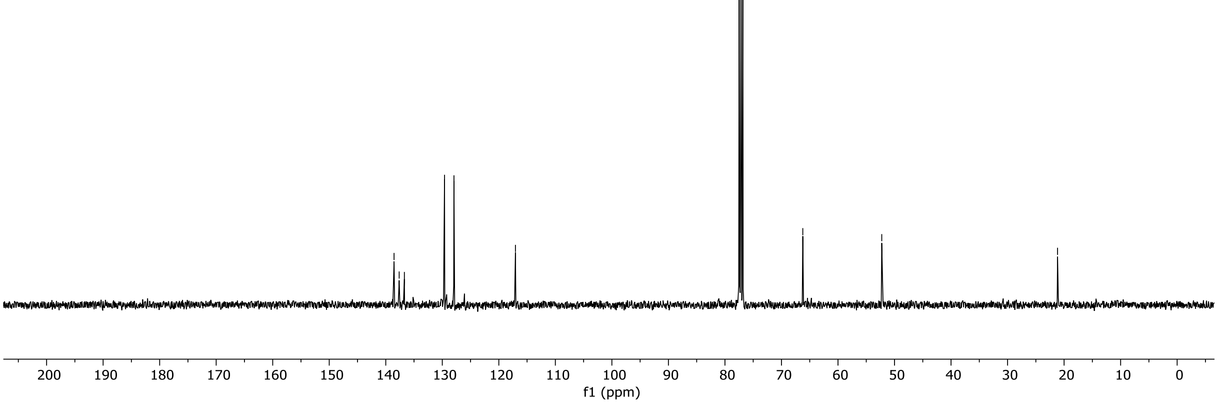

^{1}H NMR (400 MHz, $CDCl_3$) of **32** (<u>*see NMR data*</u>)

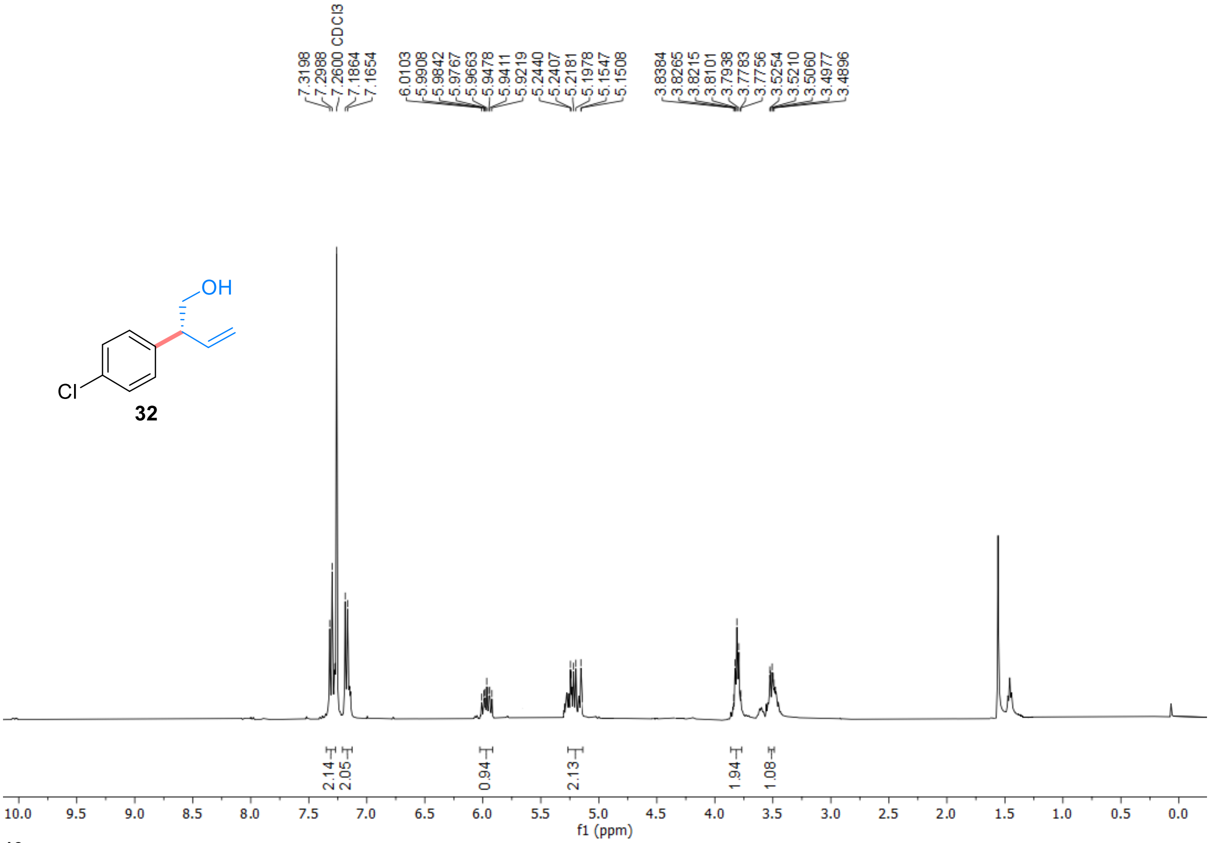

^{13}C NMR (101 MHz, $CDCl_3$) of **32**

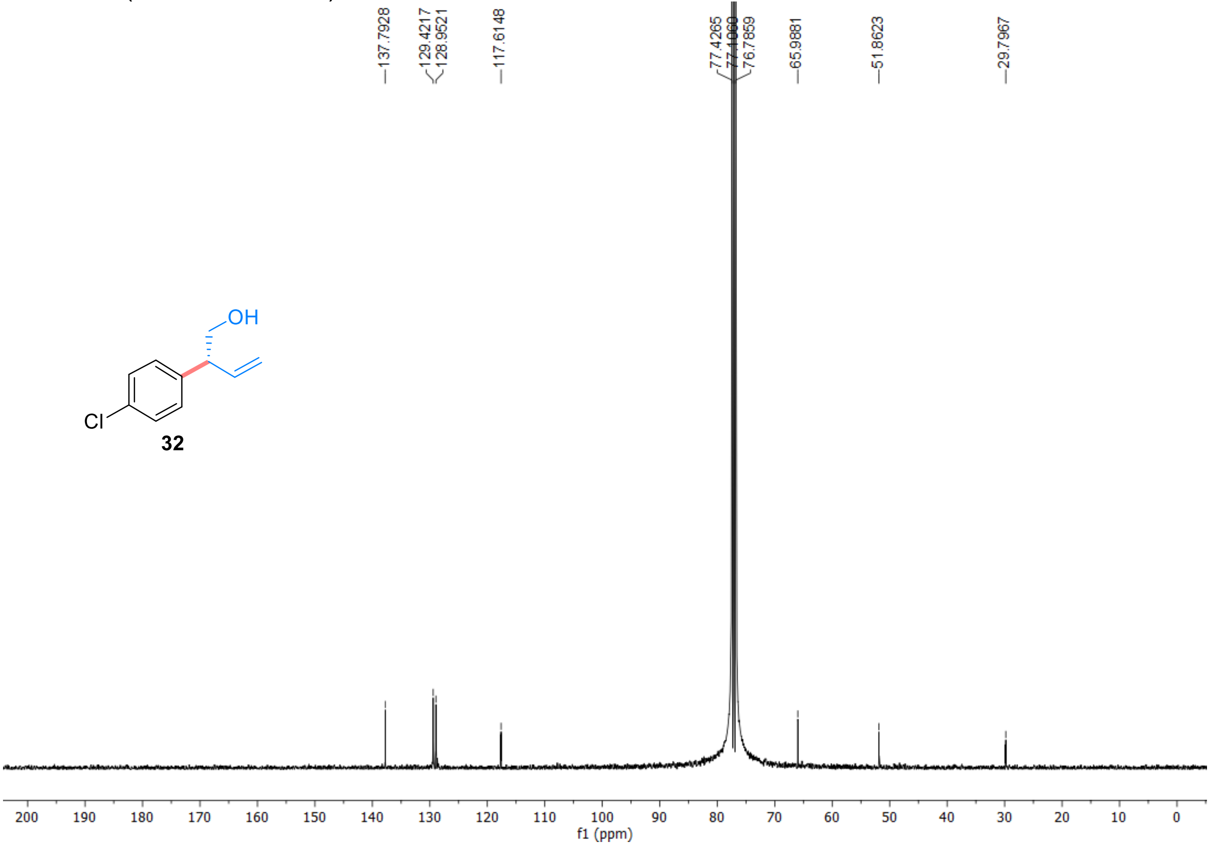

^{1}**H NMR** (400 MHz, $CDCl_3$) of **33** (*see NMR data*)

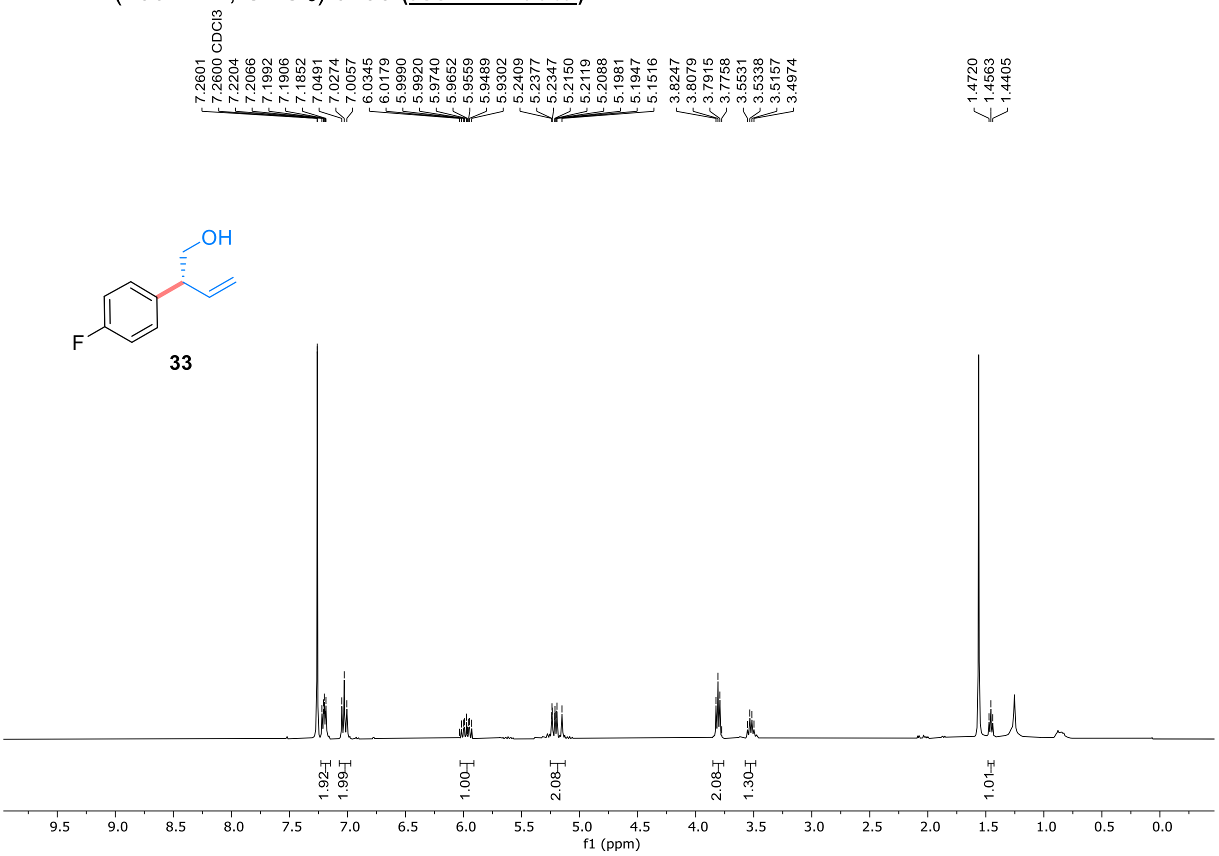

^{13}**C NMR**(101 MHz, $CDCl_3$) of **33**

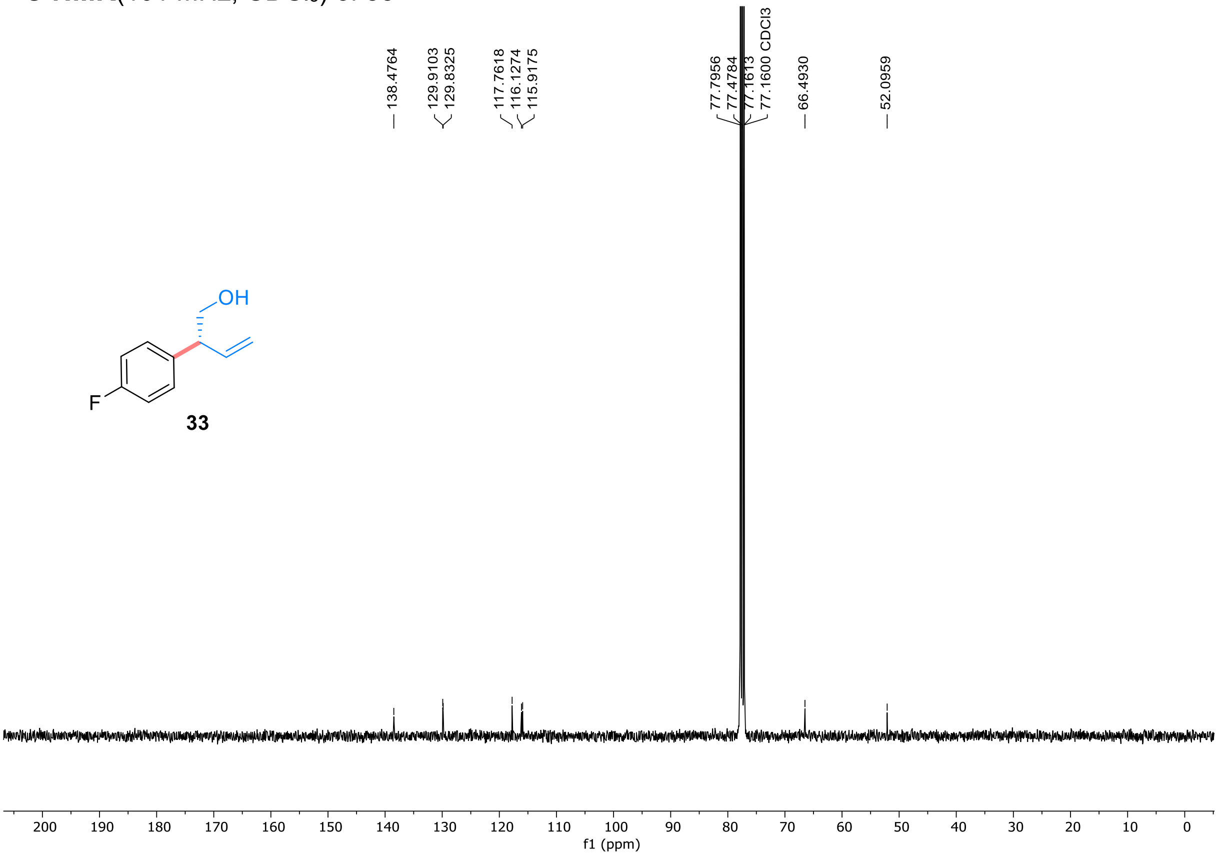

^{19}F NMR (376MHz, $CDCl_3$) of **33**

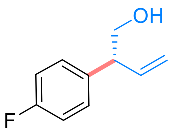

33

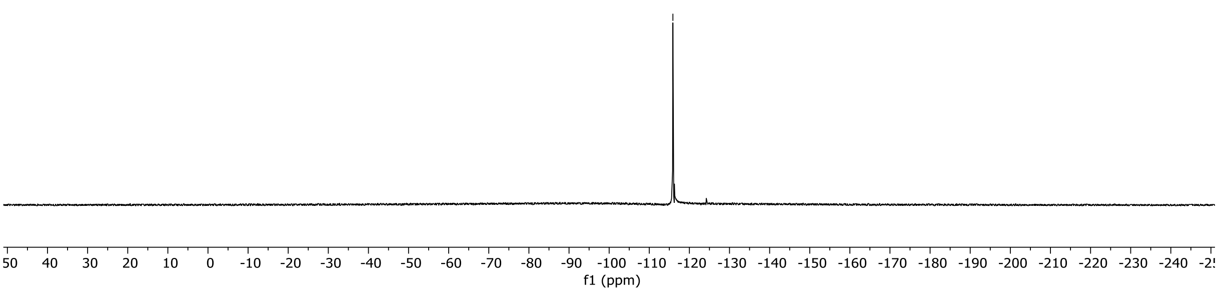

^{1}H NMR (400 MHz, $CDCl_3$) of **34** (*see NMR data*)

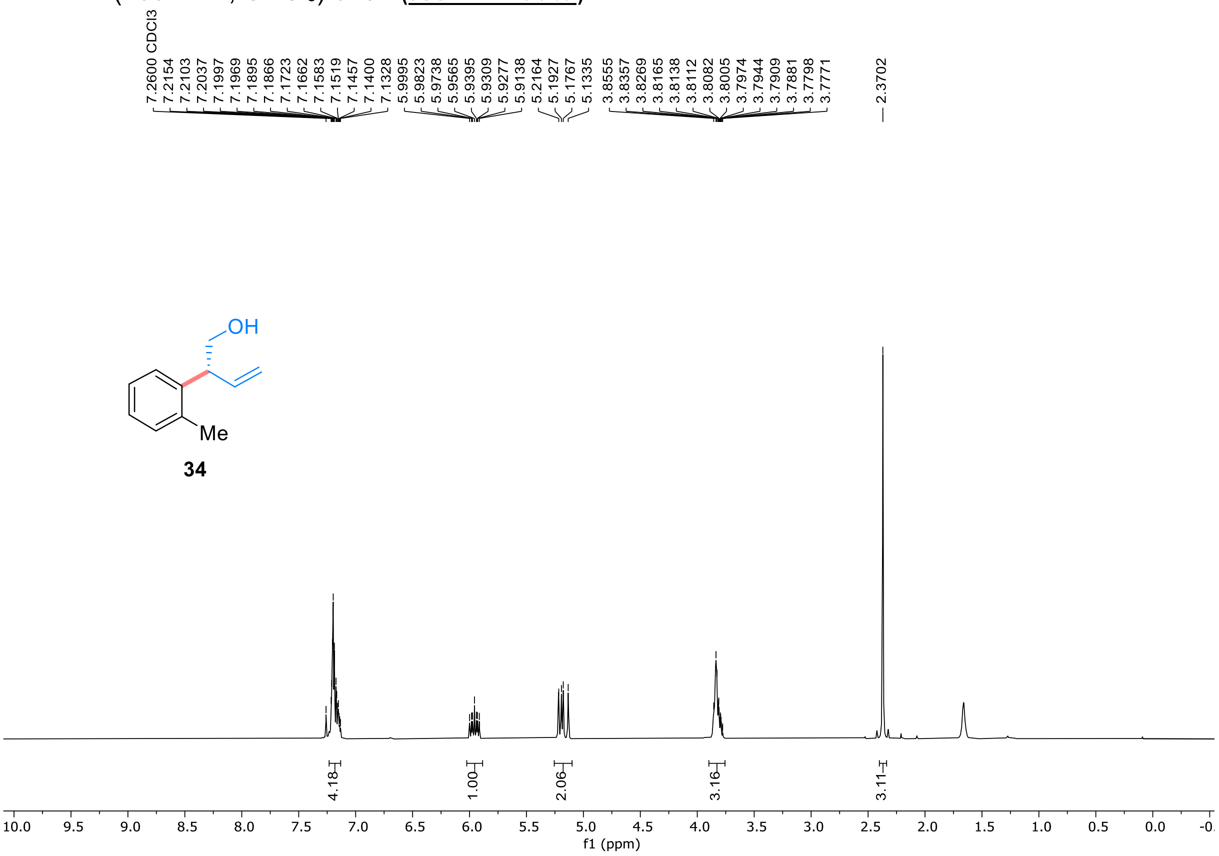

^{13}C NMR (101 MHz, $CDCl_3$) of **34**

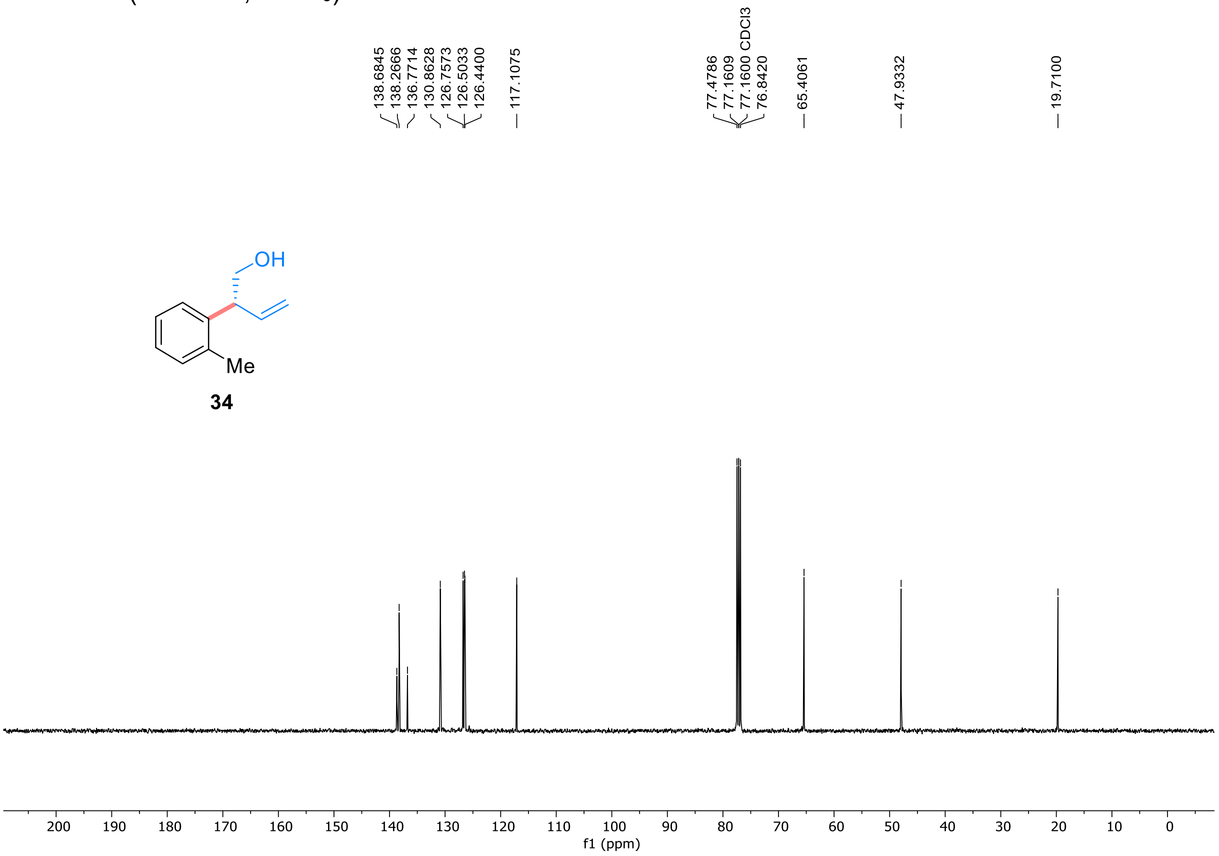

¹H NMR (400 MHz, CDCl₃) of **35** (*see NMR data*)

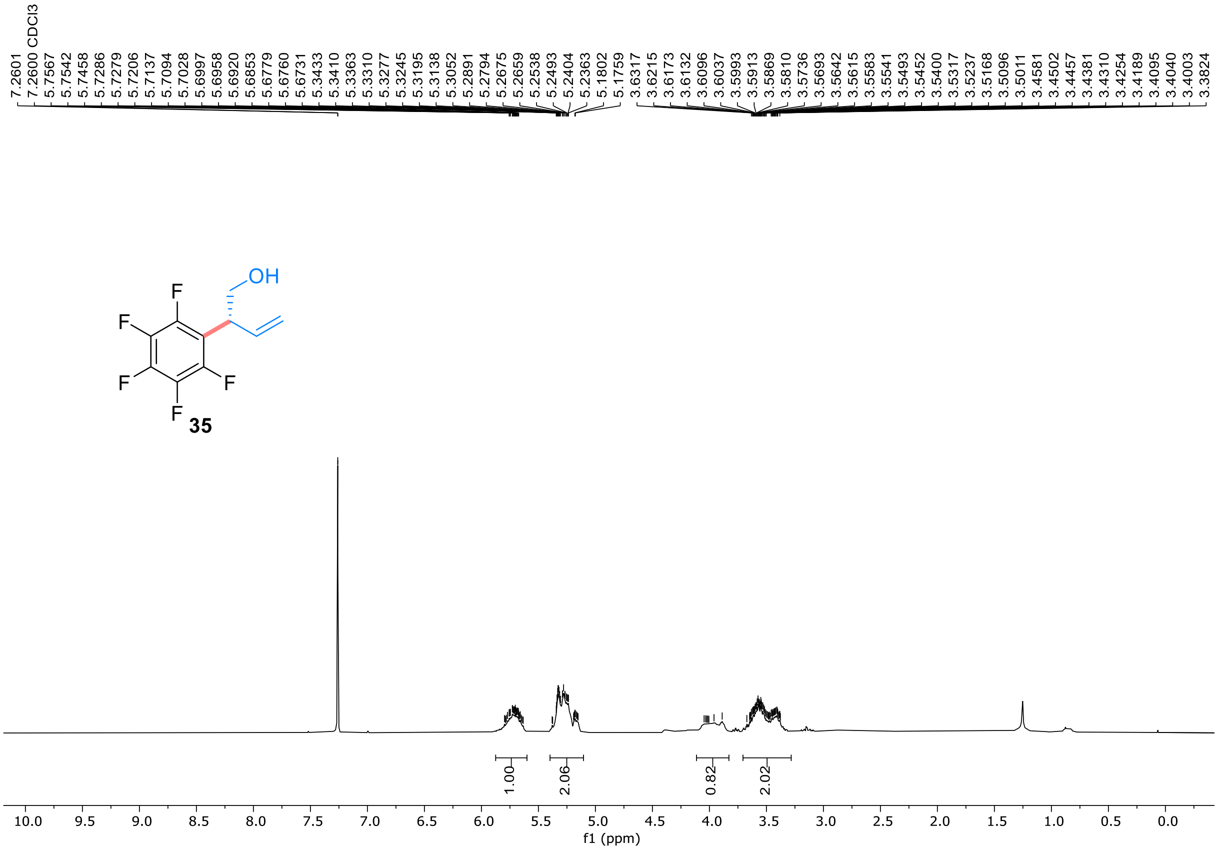

¹³C NMR (101 MHz, CDCl₃) of **35**

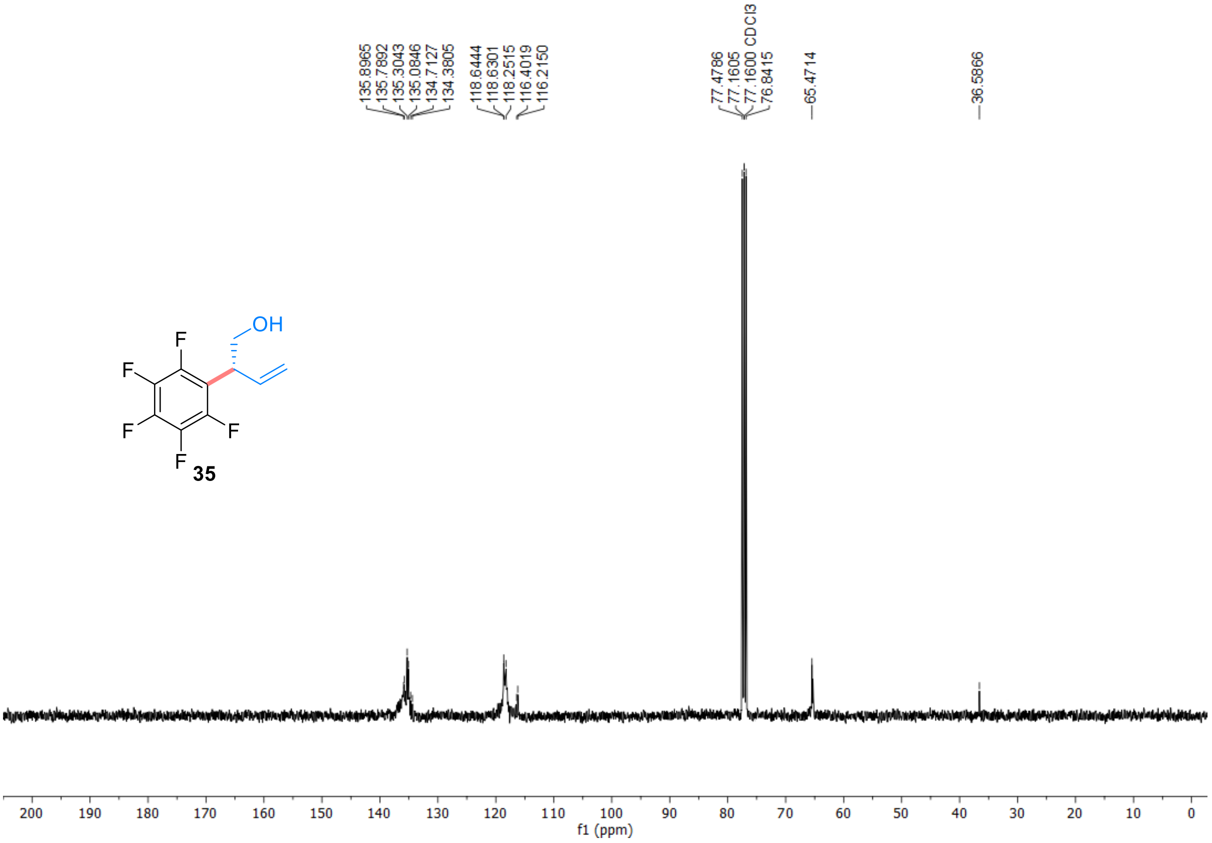

^{19}F NMR (376 MHz, $CDCl_3$) of **35**

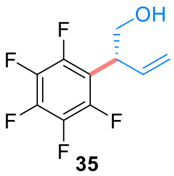

35

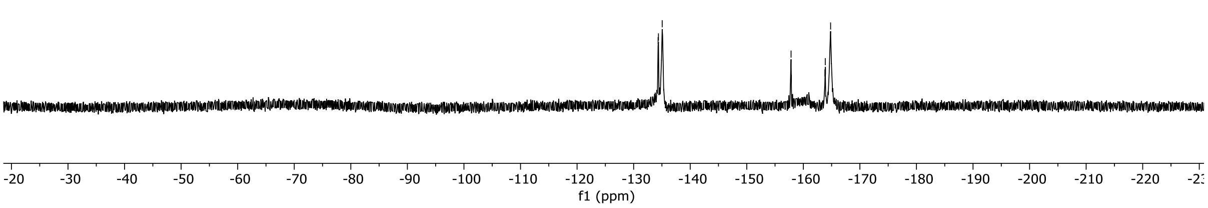

^{1}H NMR (400 MHz, $CDCl_3$) of **36** (*see NMR data*)

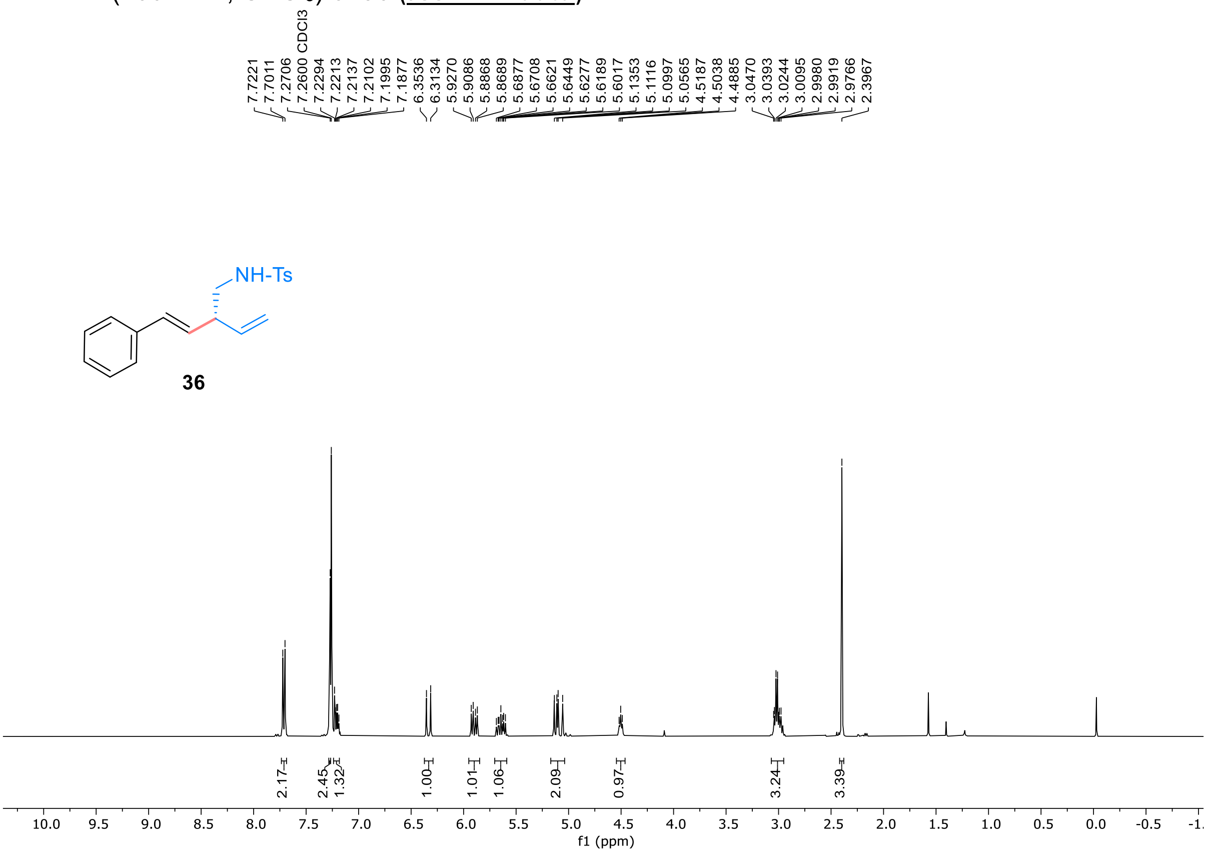

^{13}C NMR (101 MHz, $CDCl_3$) of **36**

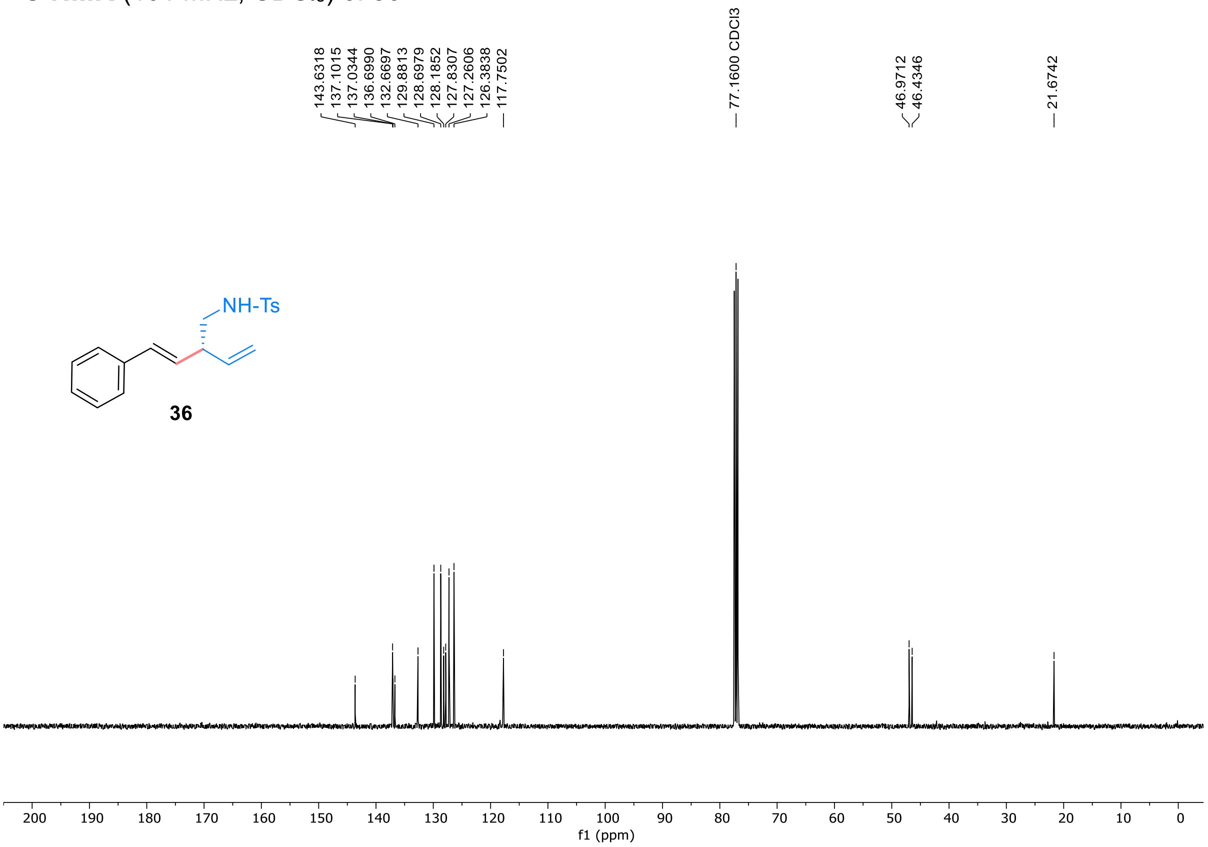

^{1}H NMR (400 MHz, CDCl$_3$) of **37** (*see NMR data*)

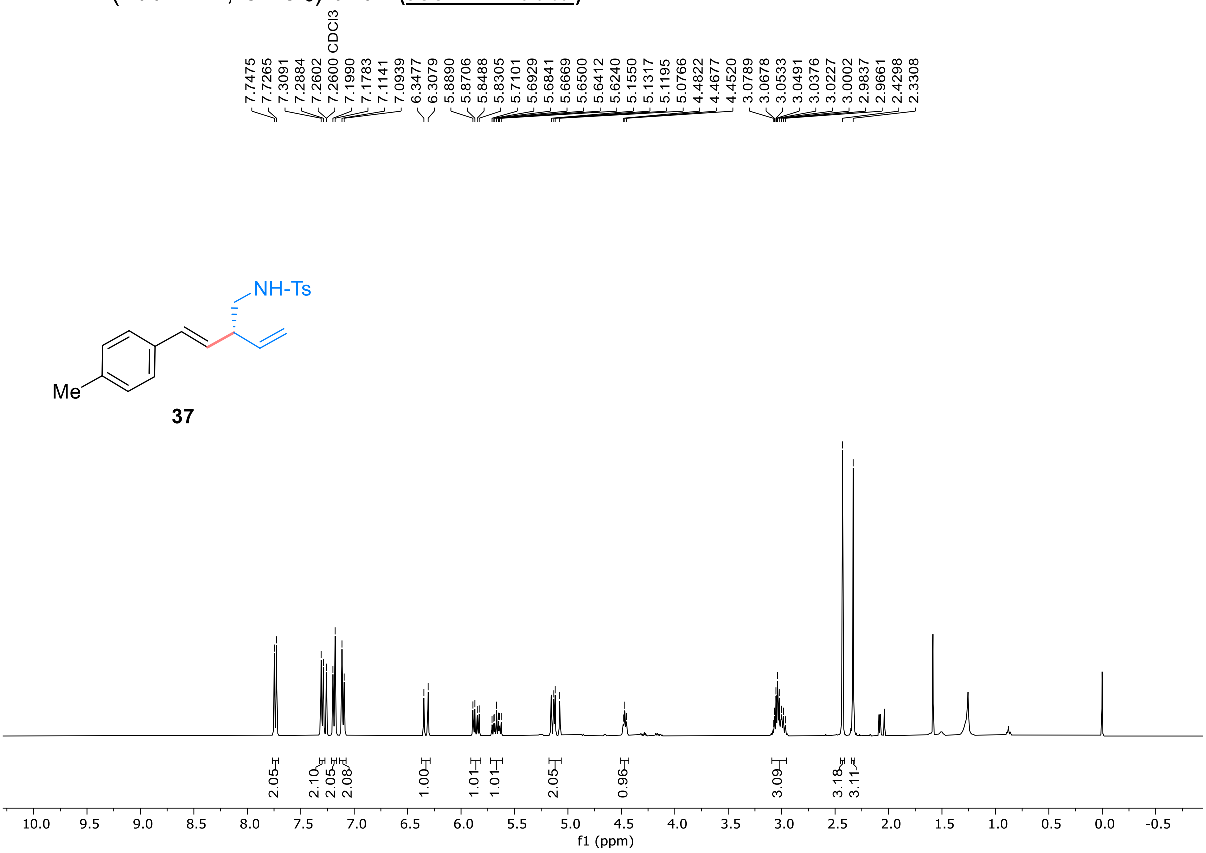

^{13}C NMR (101 MHz, CDCl$_3$) of **37**

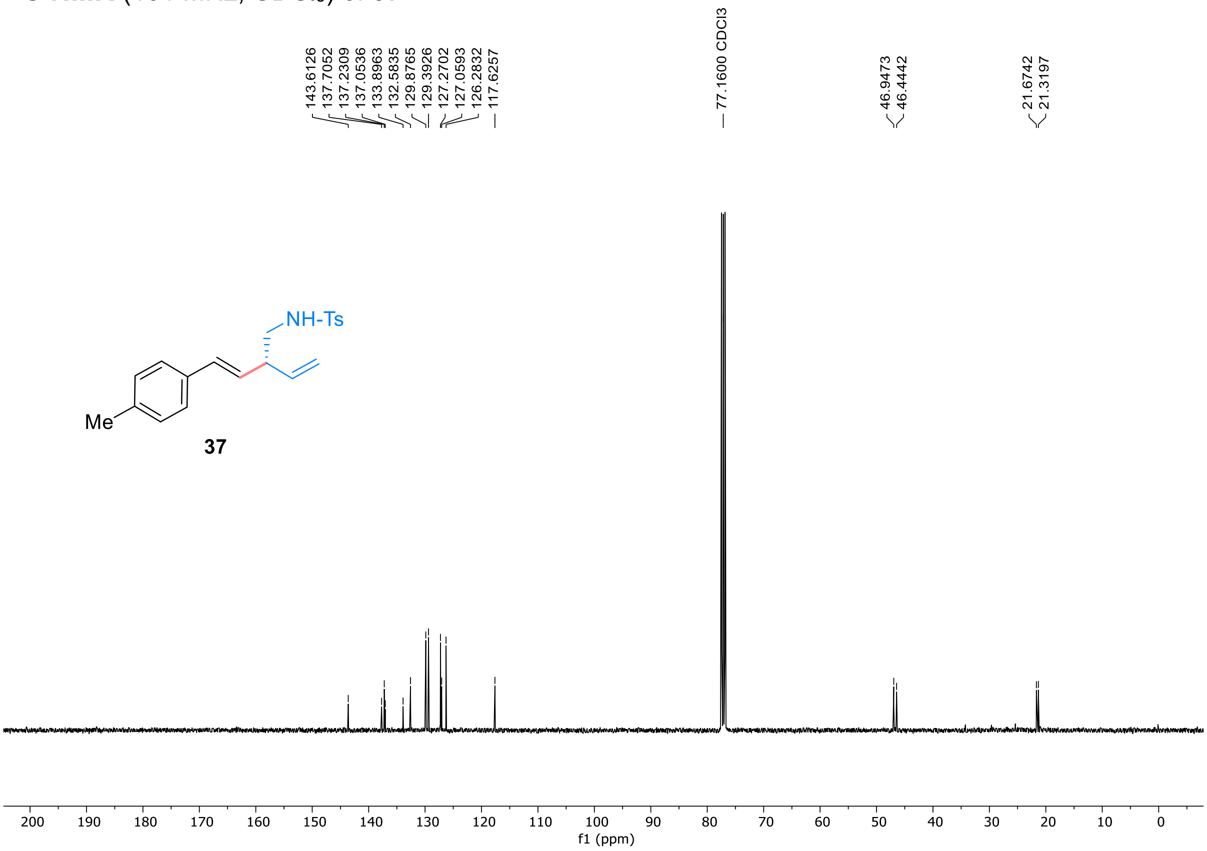

^{1}H NMR (400 MHz, $CDCl_3$) of **38** (*see NMR data*)

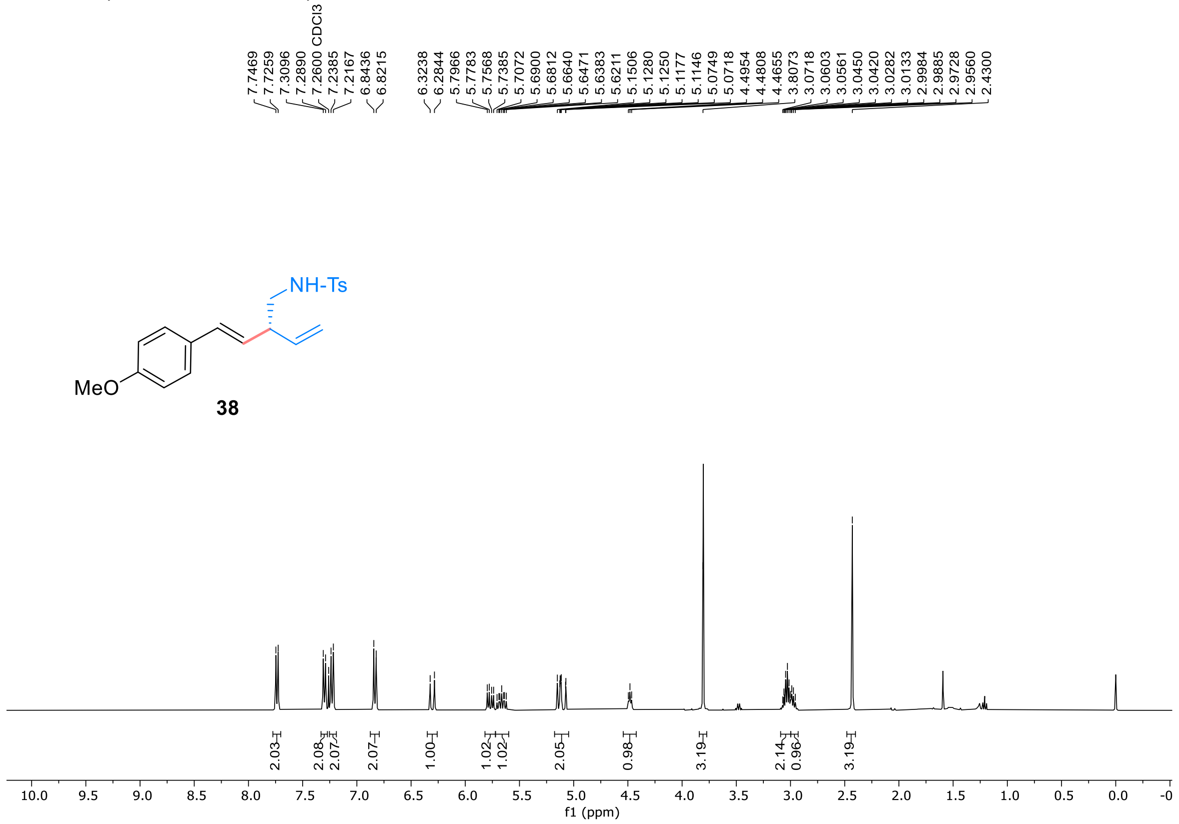

^{13}C NMR (101 MHz, $CDCl_3$) of **38**

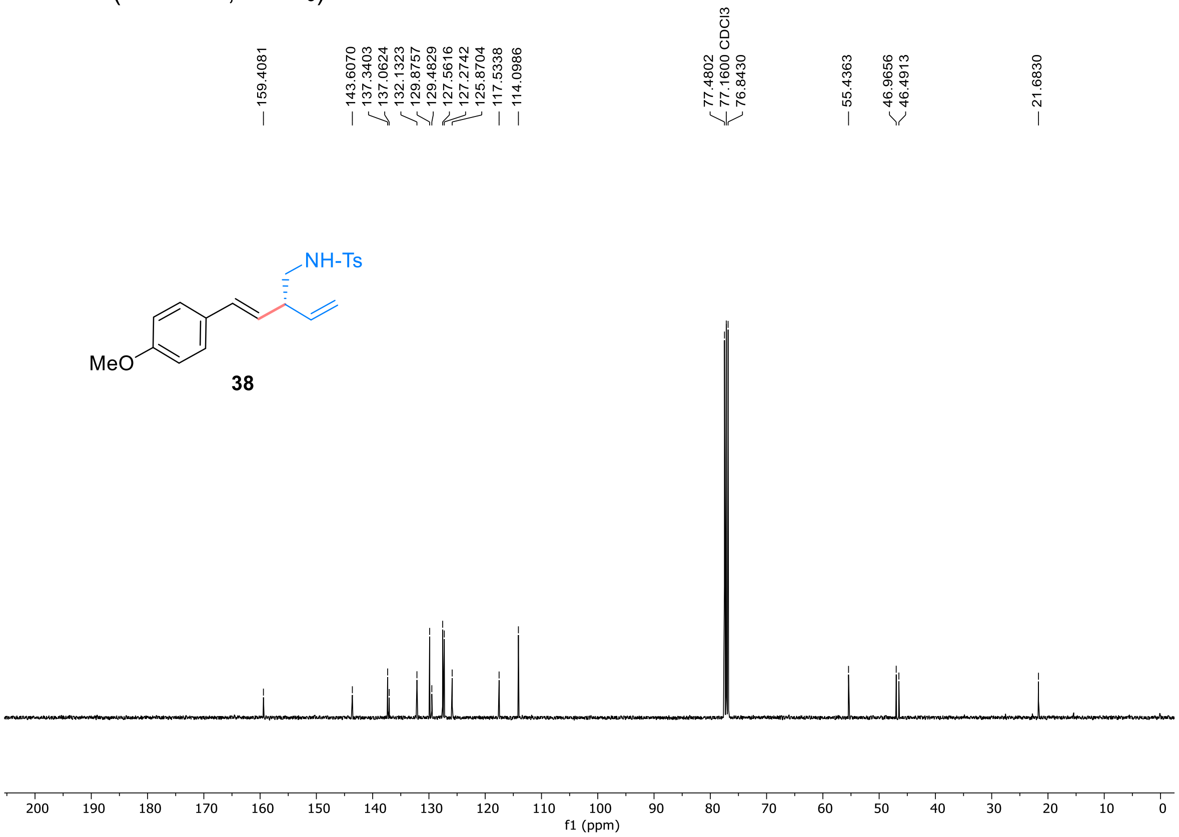

^{1}H NMR (400 MHz, $CDCl_3$) of **39** (*see NMR data*)

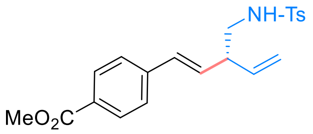

39

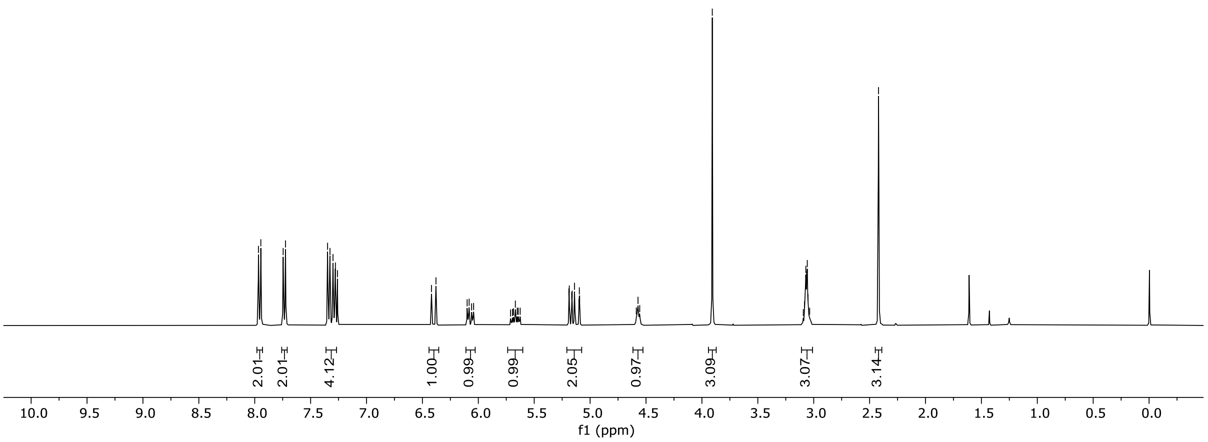

^{13}C NMR (101 MHz, $CDCl_3$) of **39**

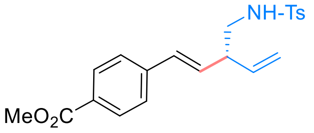

39

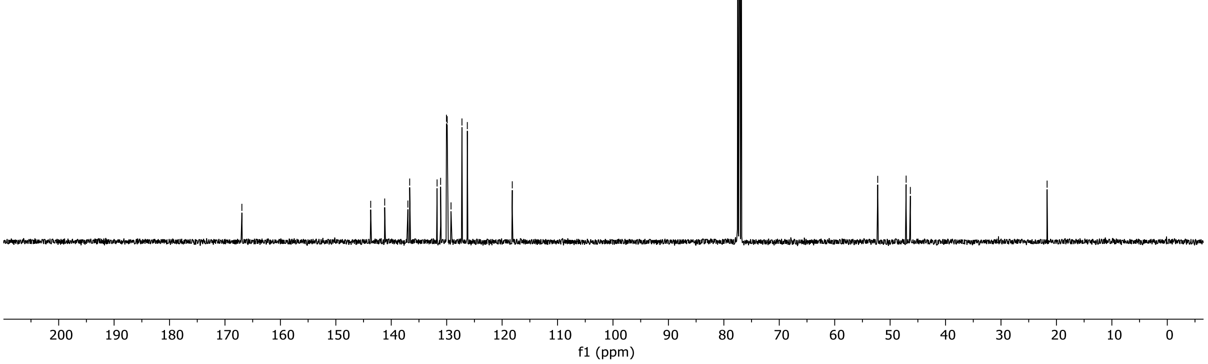

^{1}H NMR (400 MHz, $CDCl_3$) of **40** (*see NMR data*)

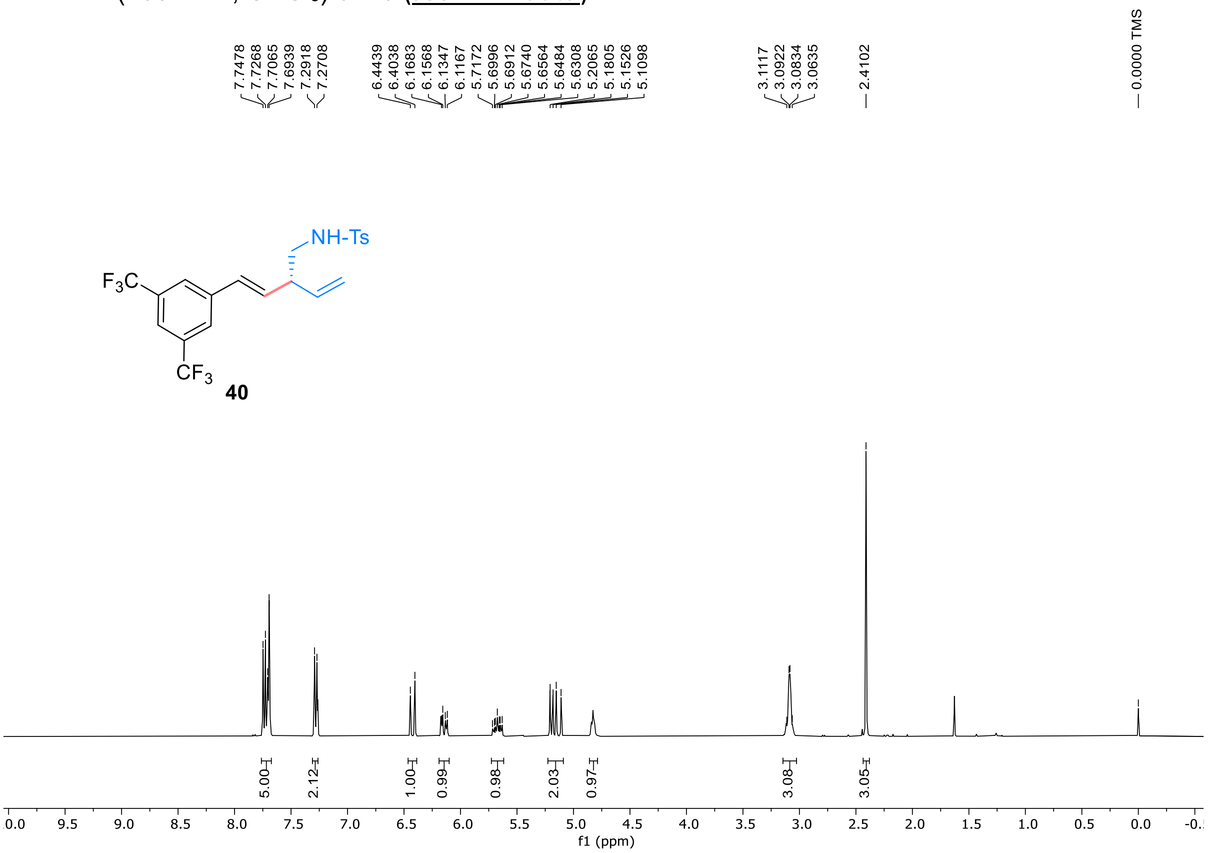

^{13}C NMR (101 MHz, $CDCl_3$) of **40**

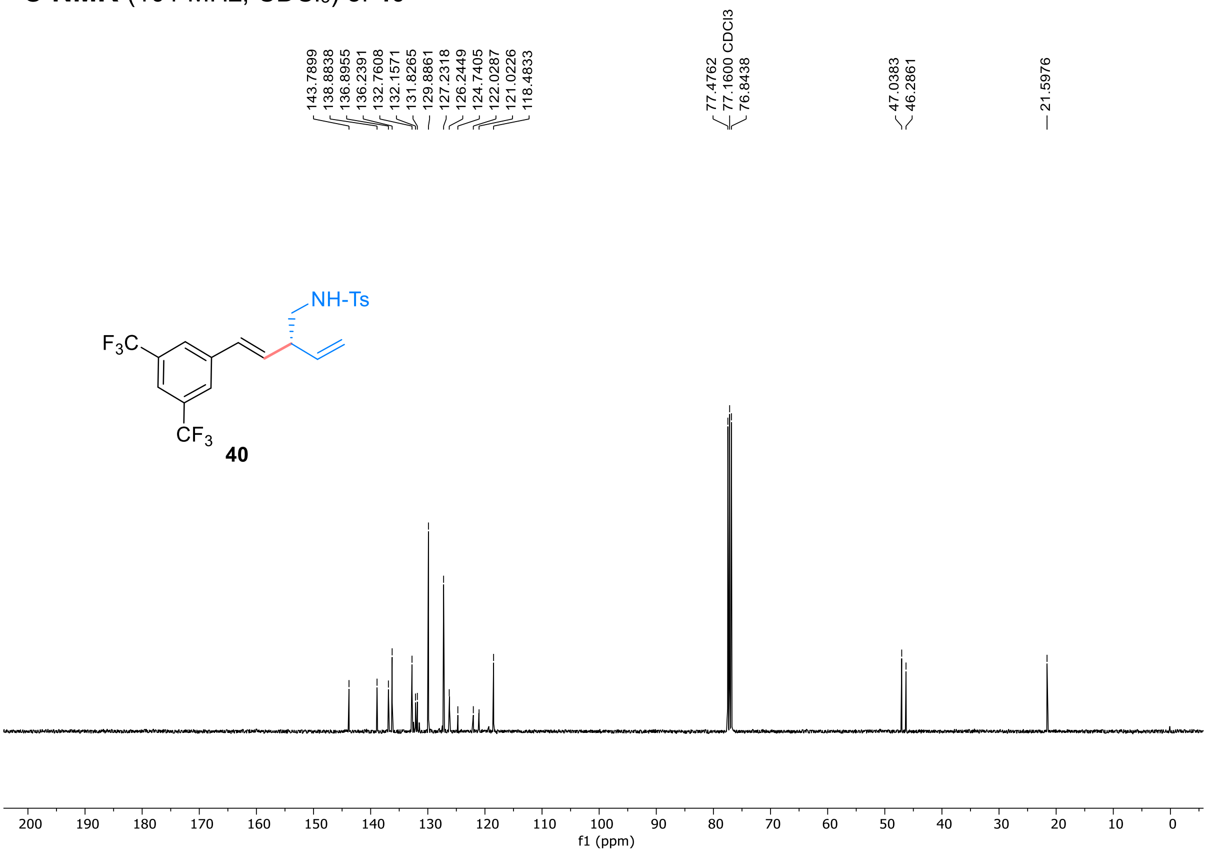

^{1}H NMR (400 MHz, CDCl$_3$) of 41 (*see NMR data*)

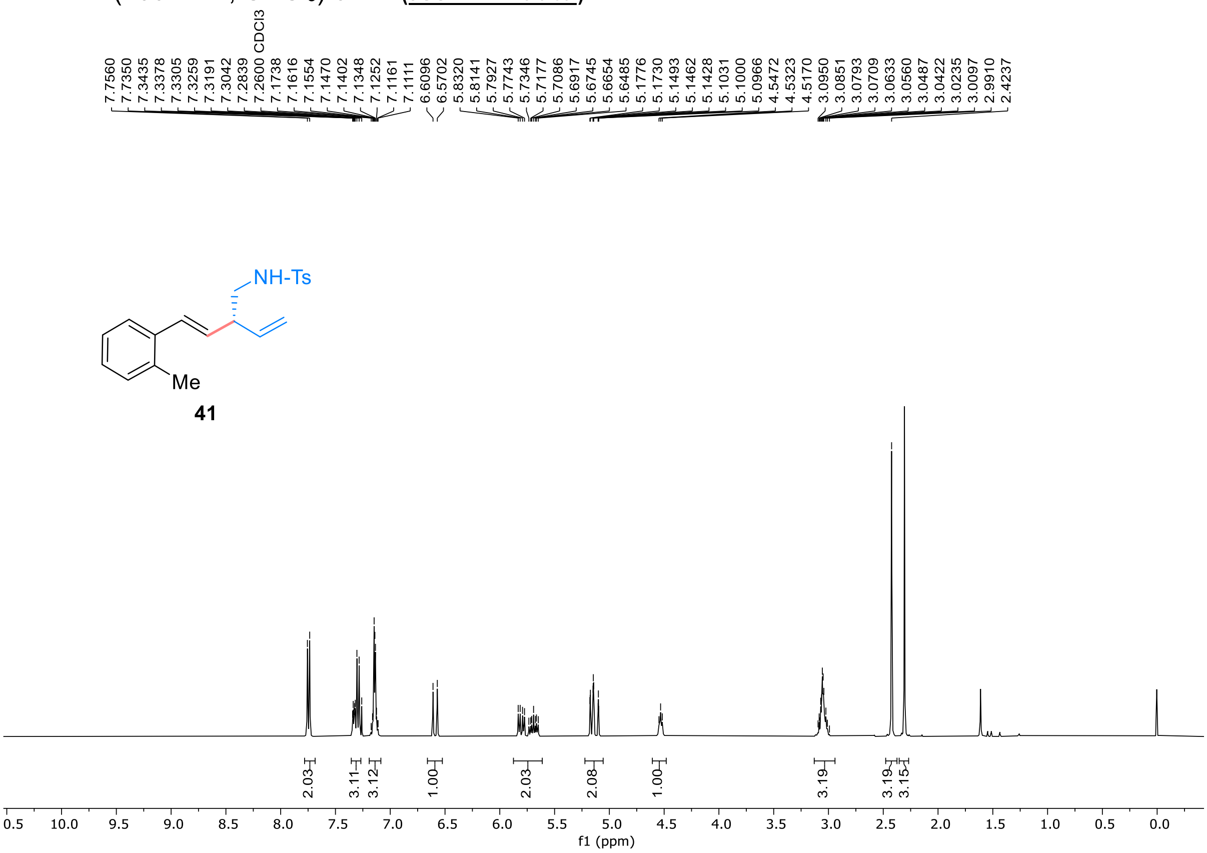

^{13}C NMR (101 MHz, CDCl$_3$) of 41

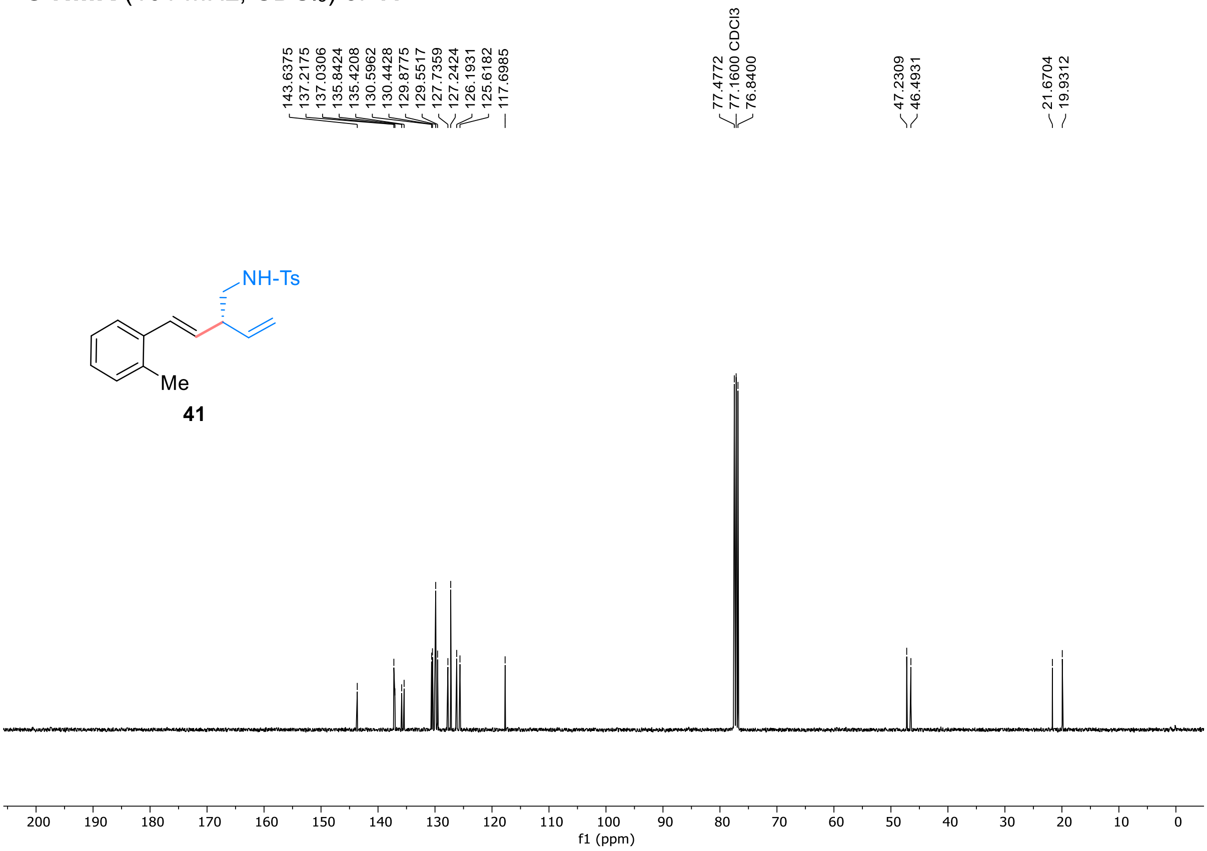

¹H NMR (400 MHz, $CDCl_3$) of **42** (*see NMR data*)

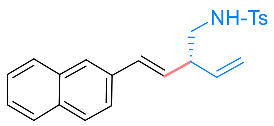

42

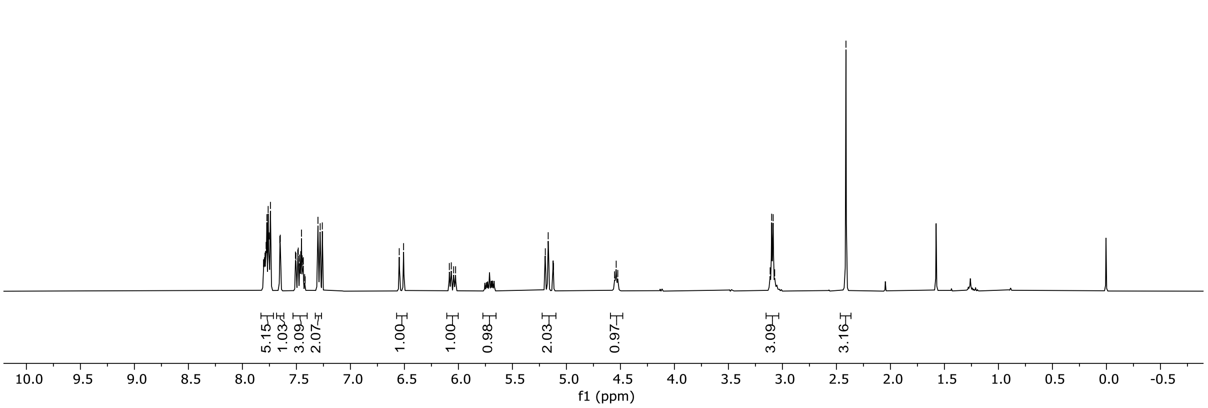

¹³C NMR (101 MHz, $CDCl_3$) of **42**

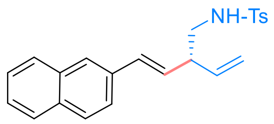

42

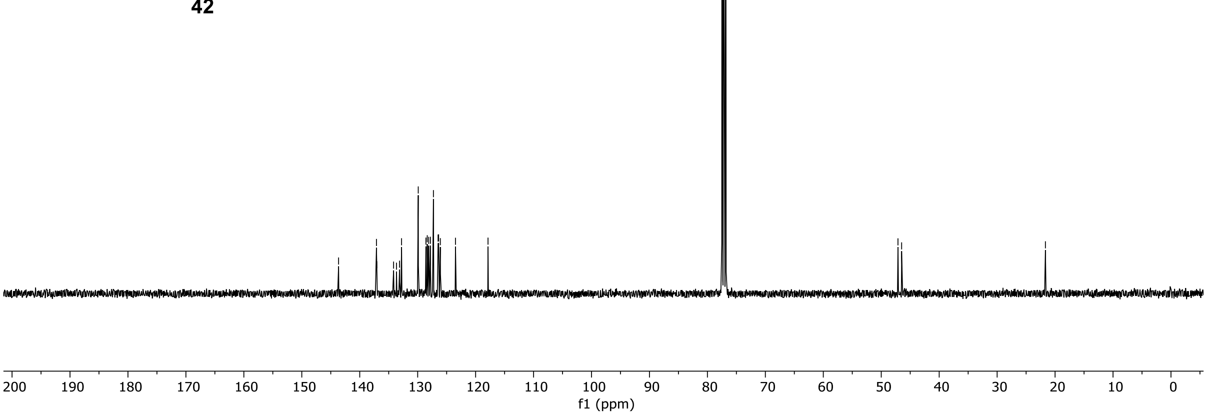

^{1}H NMR (400 MHz, $CDCl_3$) of **43** (*see NMR data*)

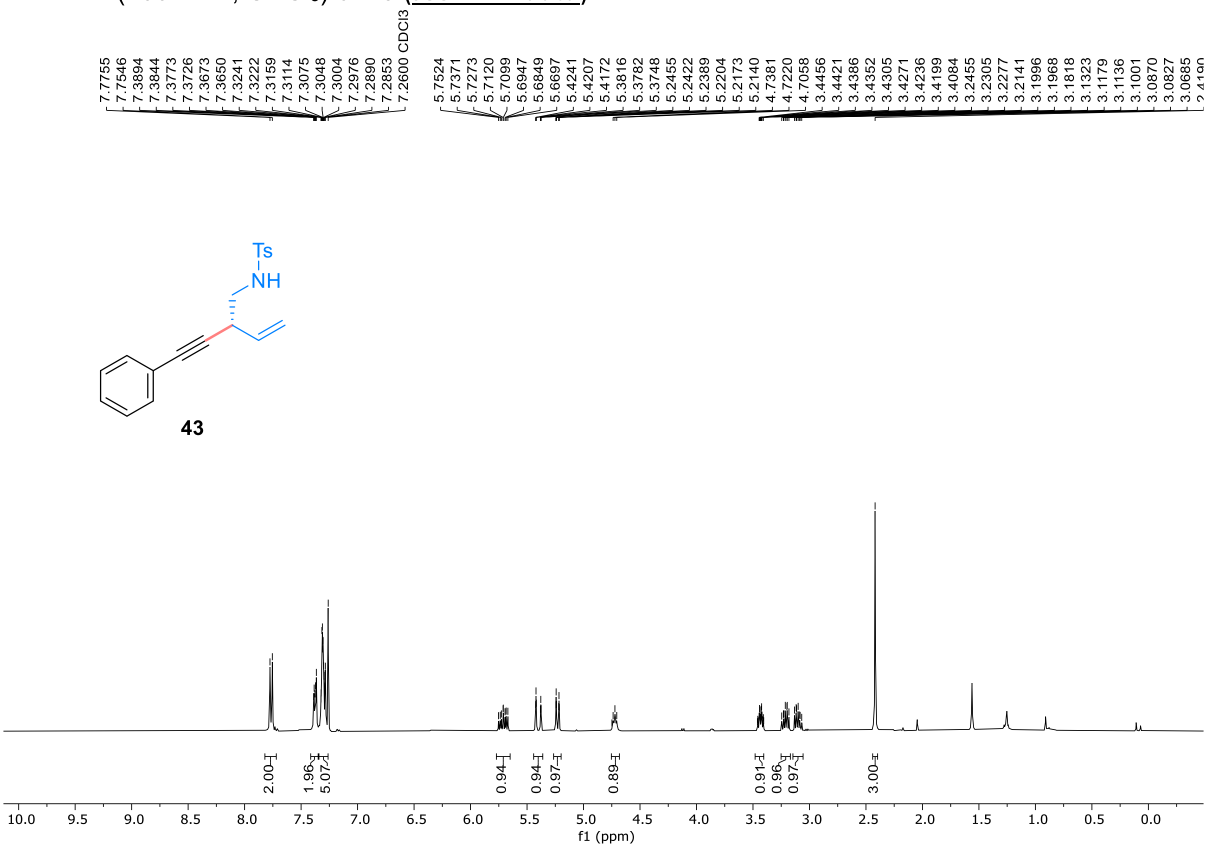

^{13}C NMR (101 MHz, $CDCl_3$) of **43**

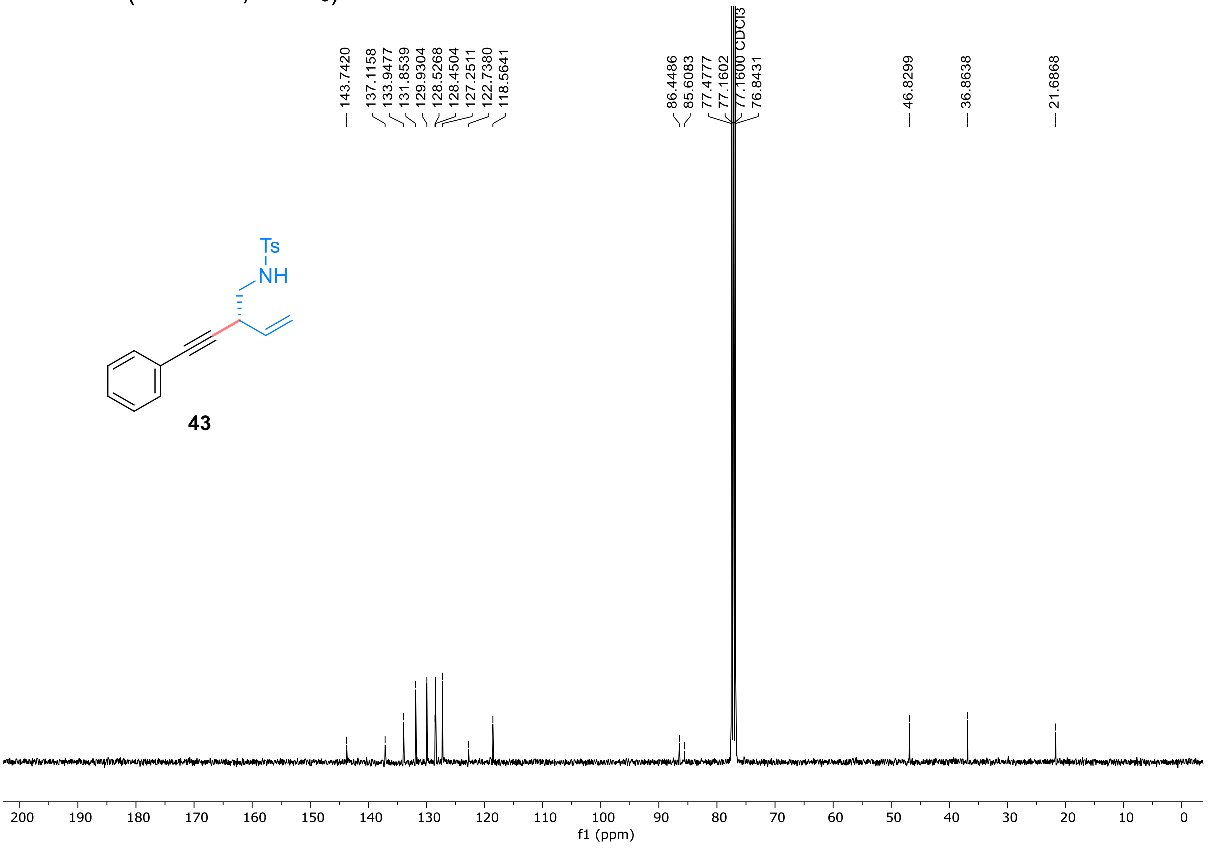

^{1}H NMR (400 MHz, $CDCl_3$) of **44** (*see NMR data*)

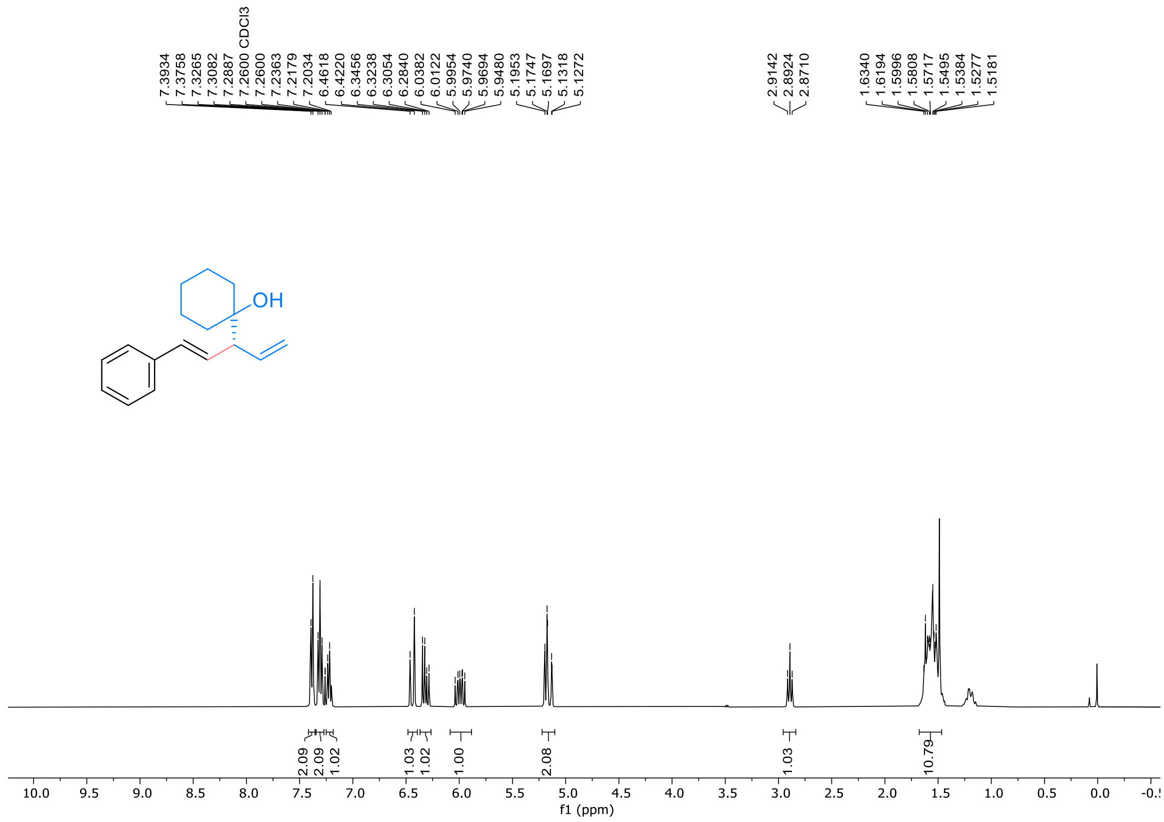

^{13}C NMR (101 MHz, $CDCl_3$) of **44**

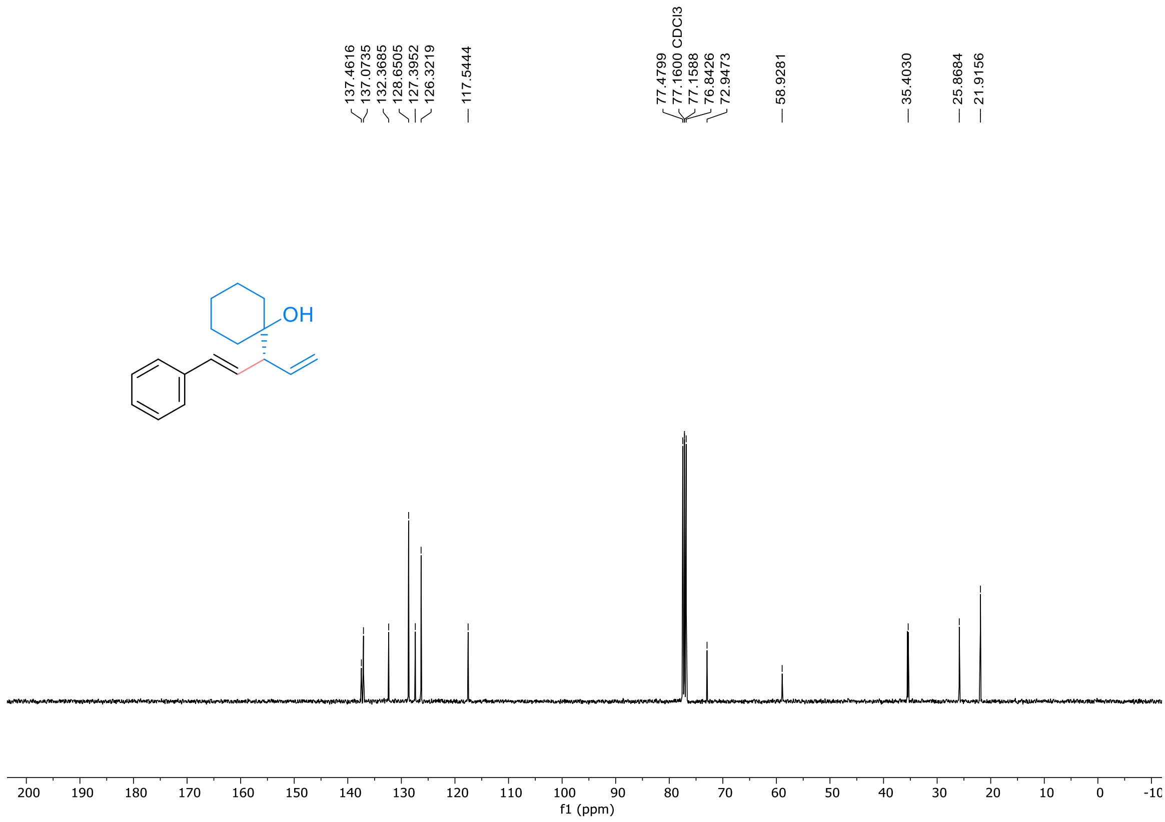

^{1}H NMR (400 MHz, CDCl3) of **45** (*see NMR data*)

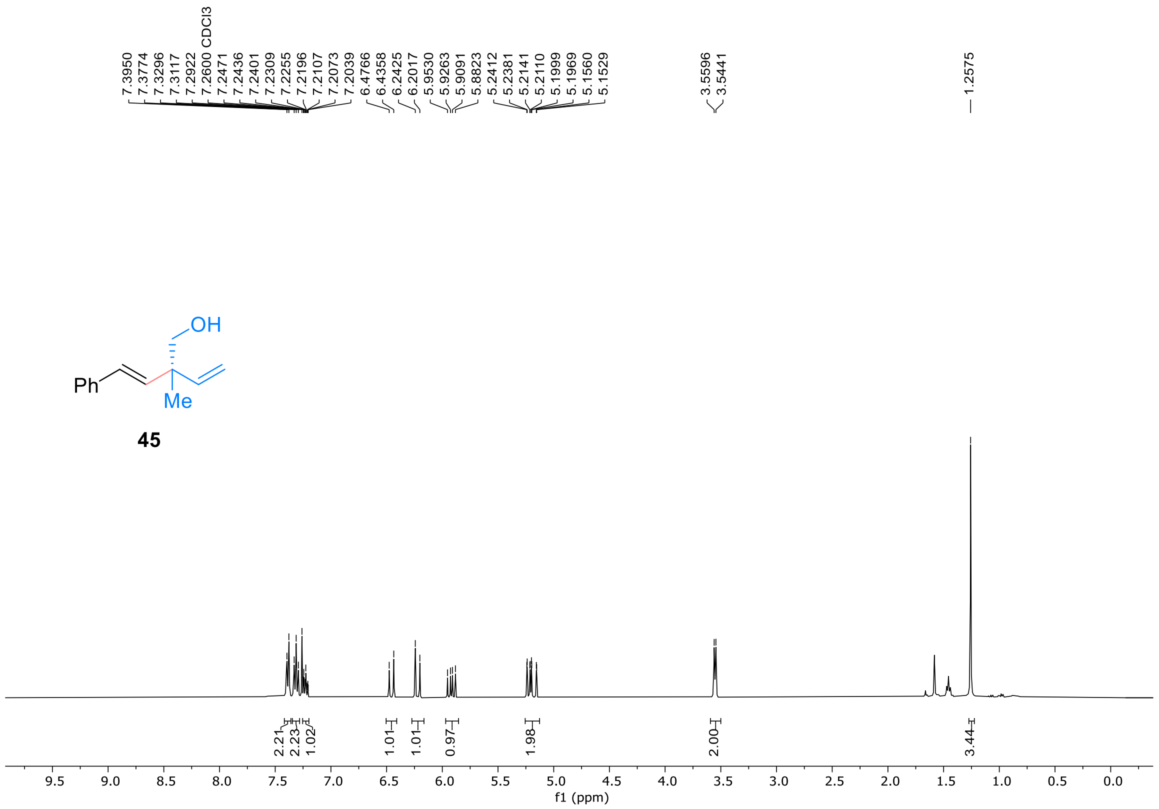

^{13}C NMR (101 MHz, CDCl3) of **45**

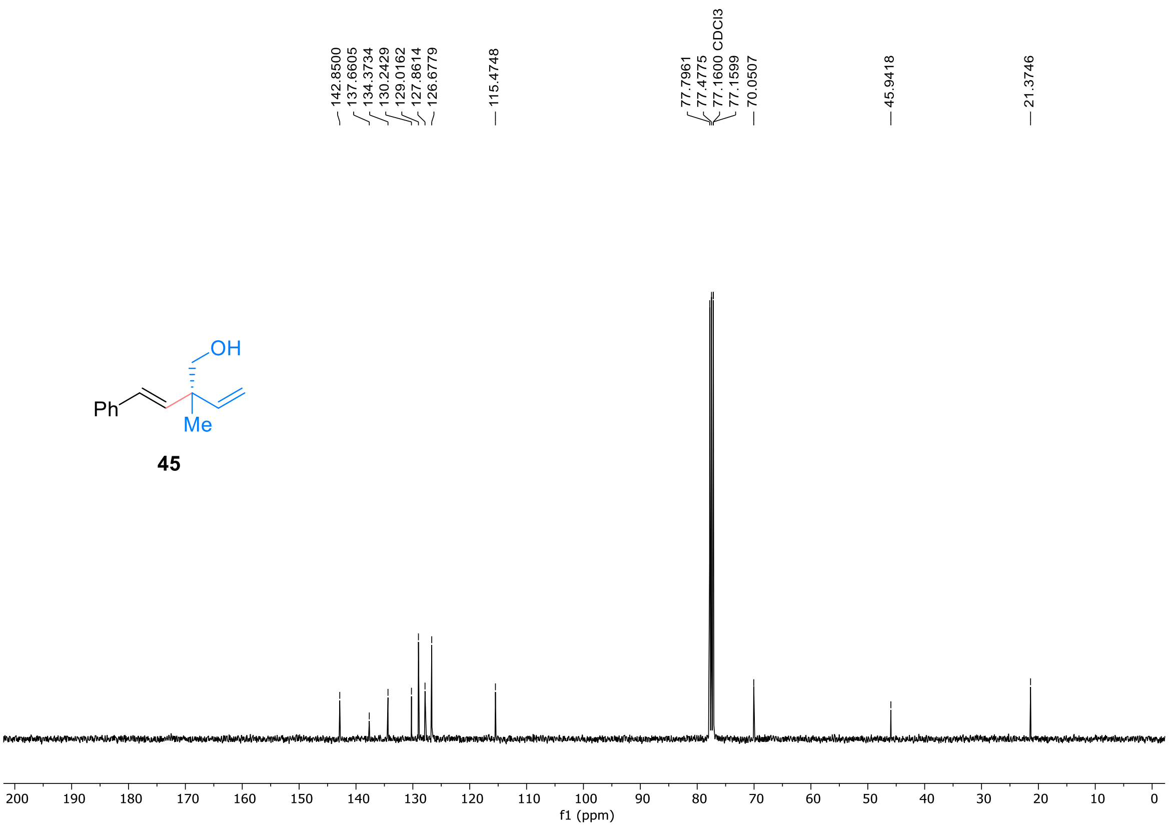

^{1}H NMR (500 MHz, $CDCl_3$) of **46** (*see NMR data*)

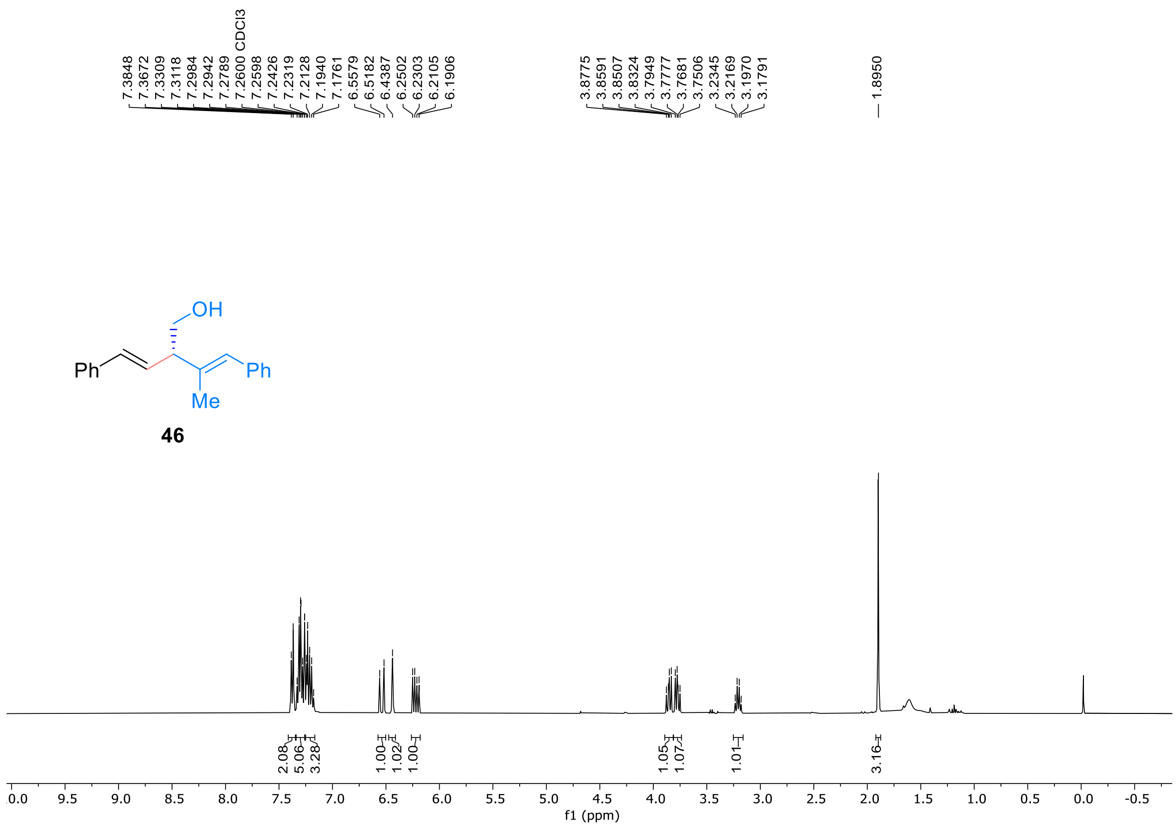

^{13}C NMR (126 MHz, $CDCl_3$) of **46**

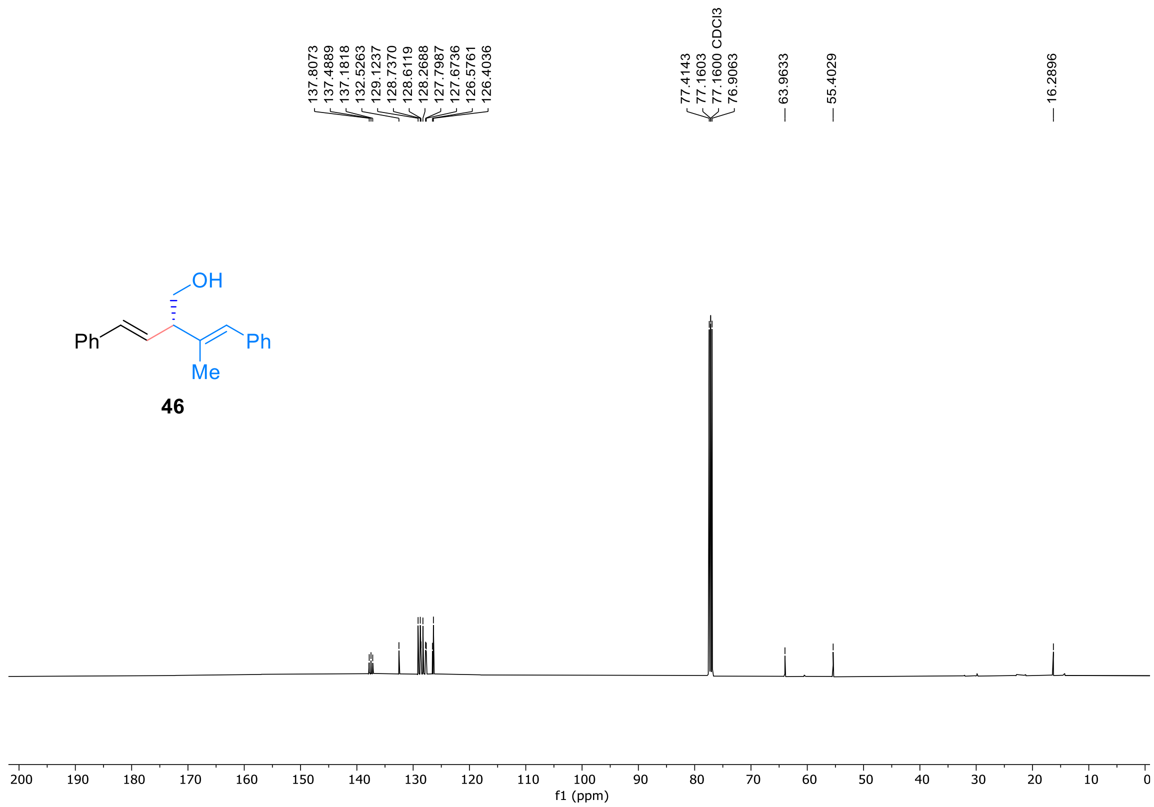

^{1}H NMR (400 MHz, $CDCl_3$) of **47** (<u>*see NMR data*</u>)

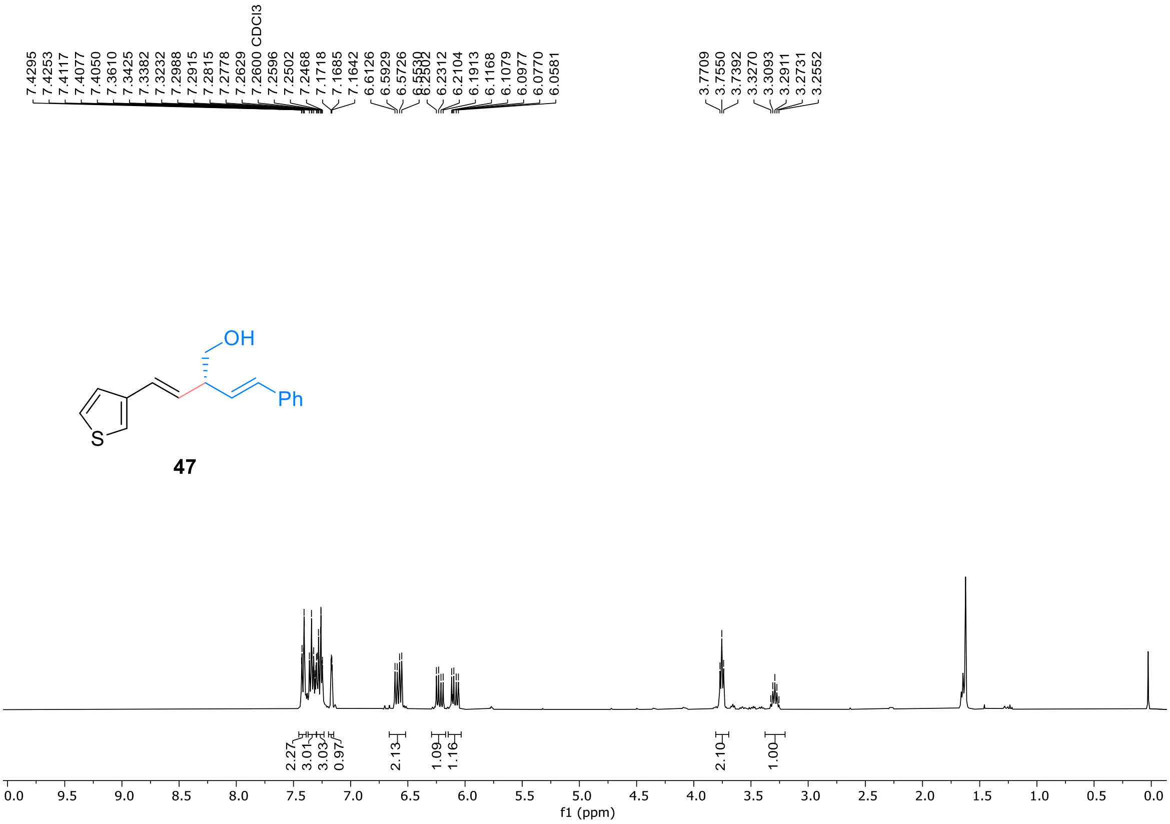

^{13}C NMR (101 MHz, $CDCl_3$) of **47**

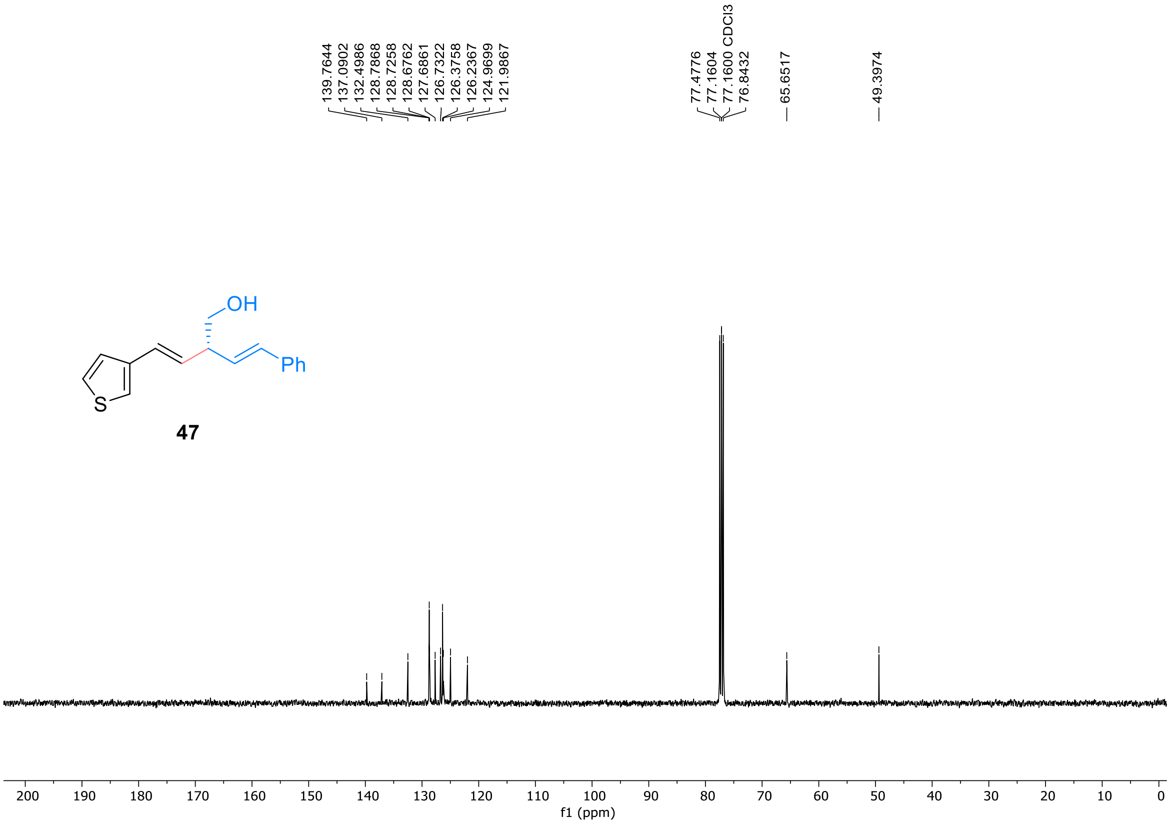

^{1}H NMR (500 MHz, $CDCl_3$) of **48** (*see NMR data*)

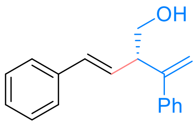

48

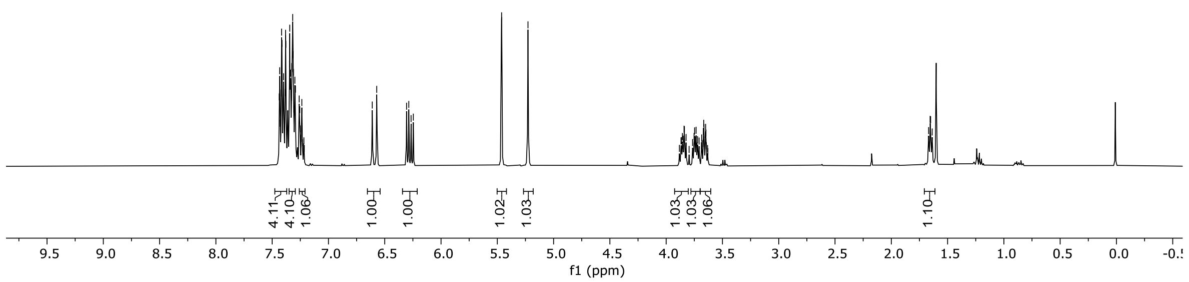

^{13}C NMR (126 MHz, $CDCl_3$) of **48**

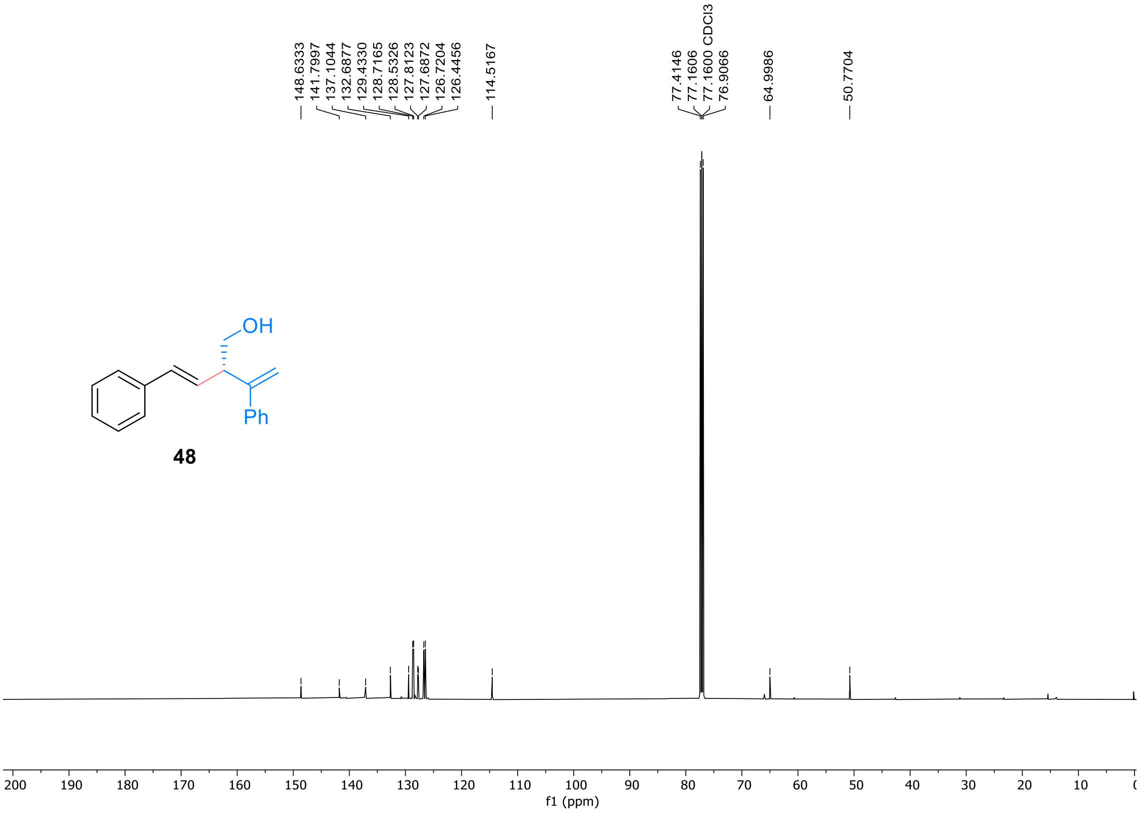
148.6333
141.7997
137.1044
132.6877
129.4330
128.7165
128.5326
127.8123
127.6872
126.7204
126.4456
114.5167
77.4146
77.1606
77.1600 CDCl3
76.9066
64.9986
50.7704
OH
Ph
200 190 180 170 160 150 140 130 120 110 100 90 80 70 60 50 40 30 20 10
f1 (ppm)

48

^{1}H NMR (400 MHz, $CDCl_3$) of **49** (<u>*see NMR data*</u>)

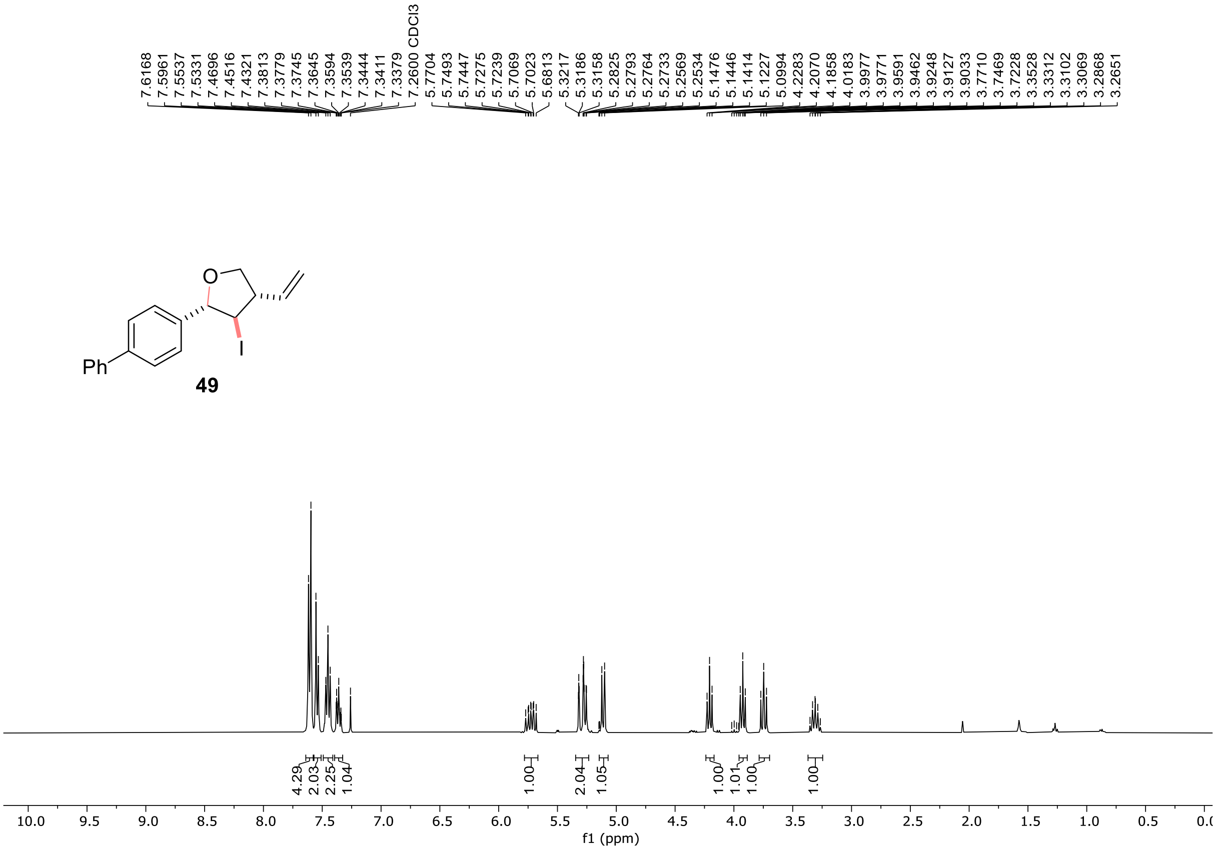

^{13}C NMR (101 MHz, $CDCl_3$) of **49**

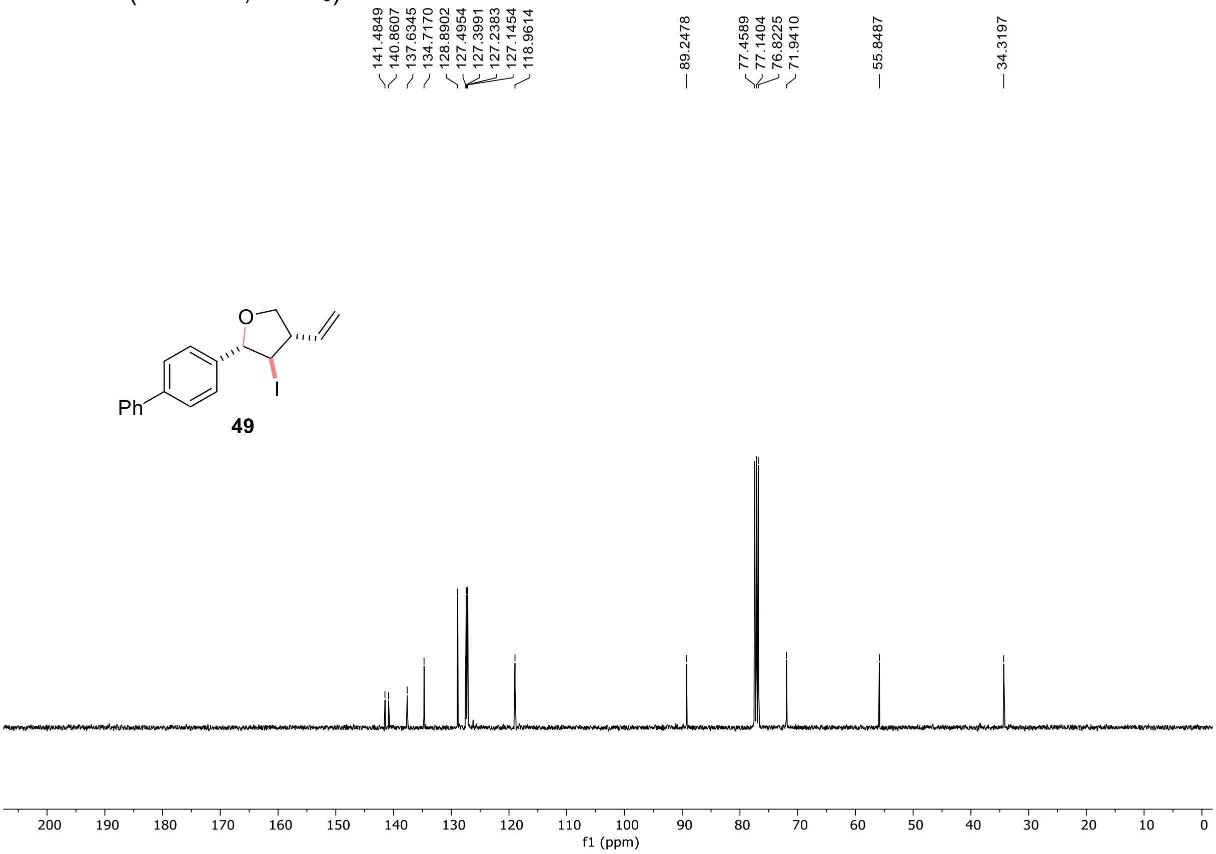

^{1}H NMR (400 MHz, $CDCl_3$) of **50** (*see NMR data*)

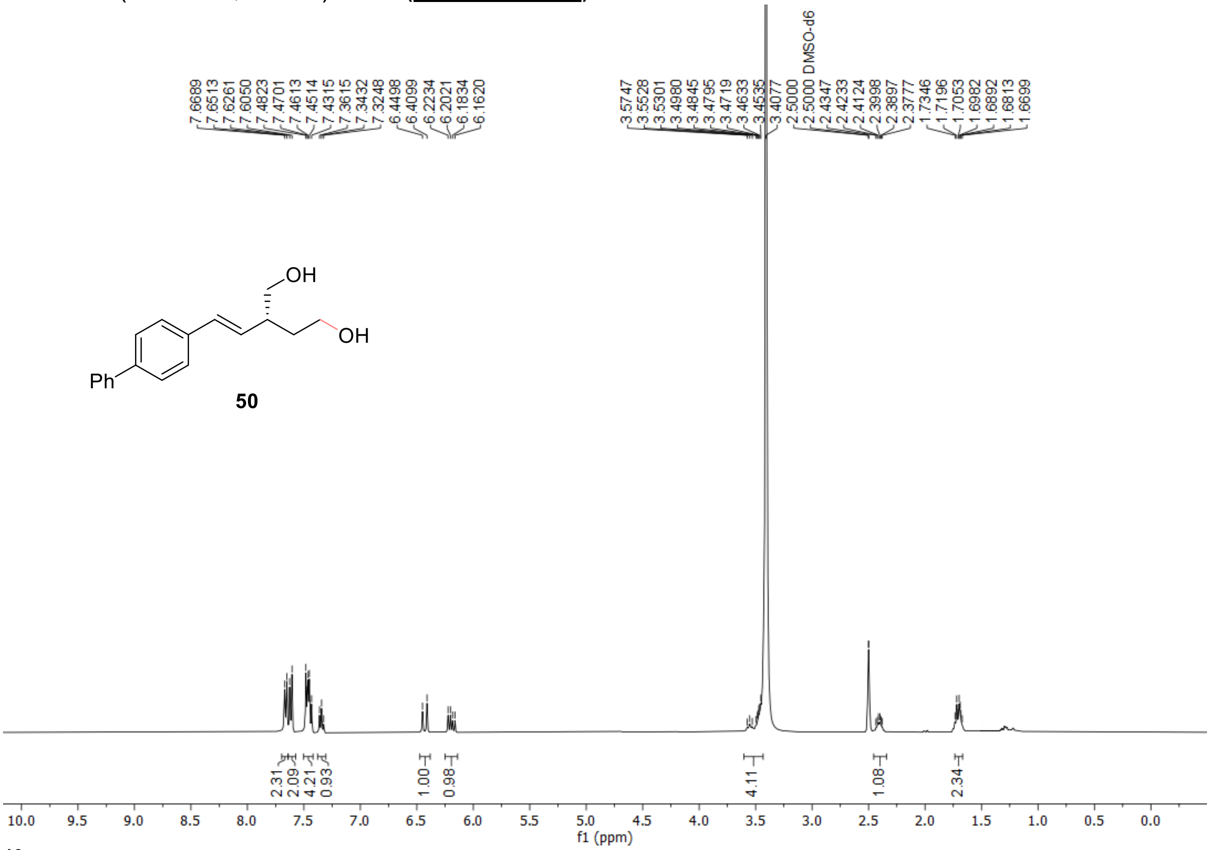

^{13}C NMR (101 MHz, $CDCl_3$) of **50**

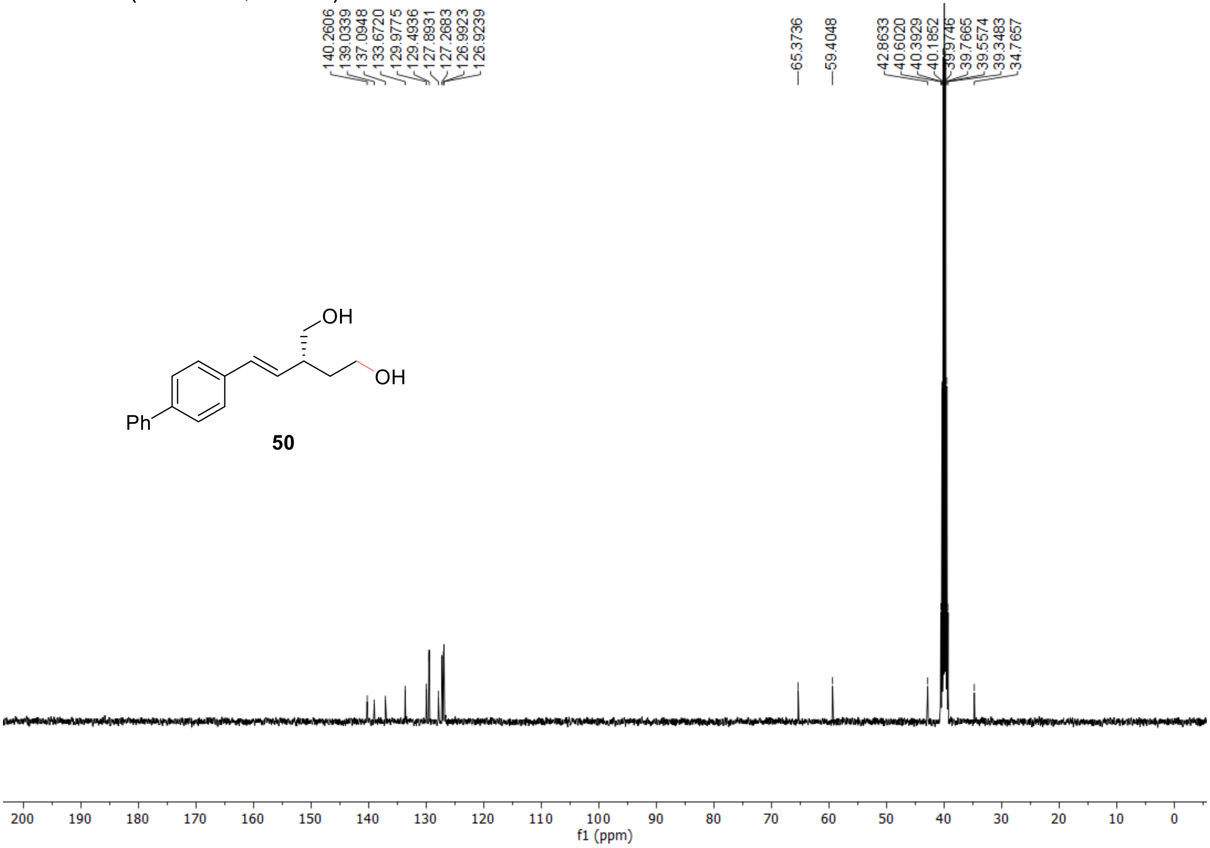

^{1}H NMR (400 MHz, $CDCl_3$) of **51** (*see NMR data*)

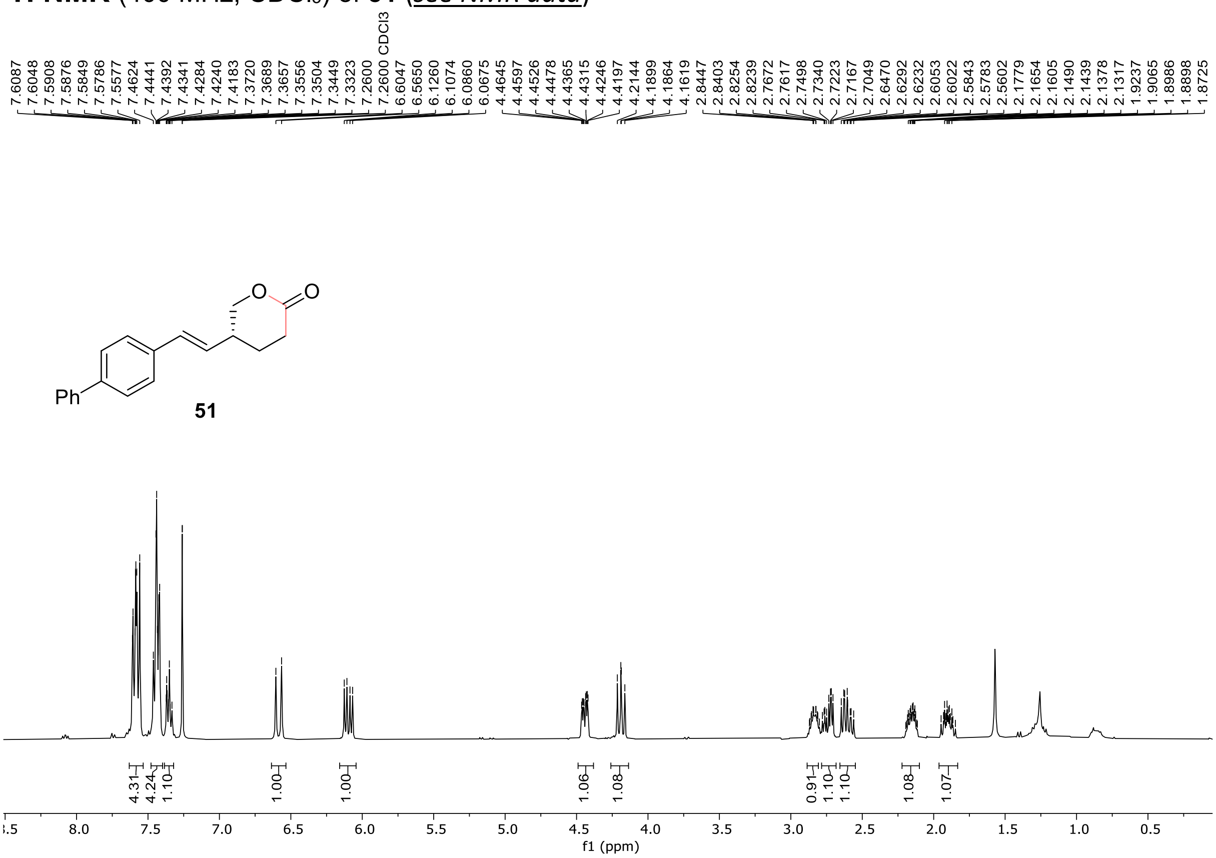

^{13}C NMR (101 MHz, $CDCl_3$) of **51**

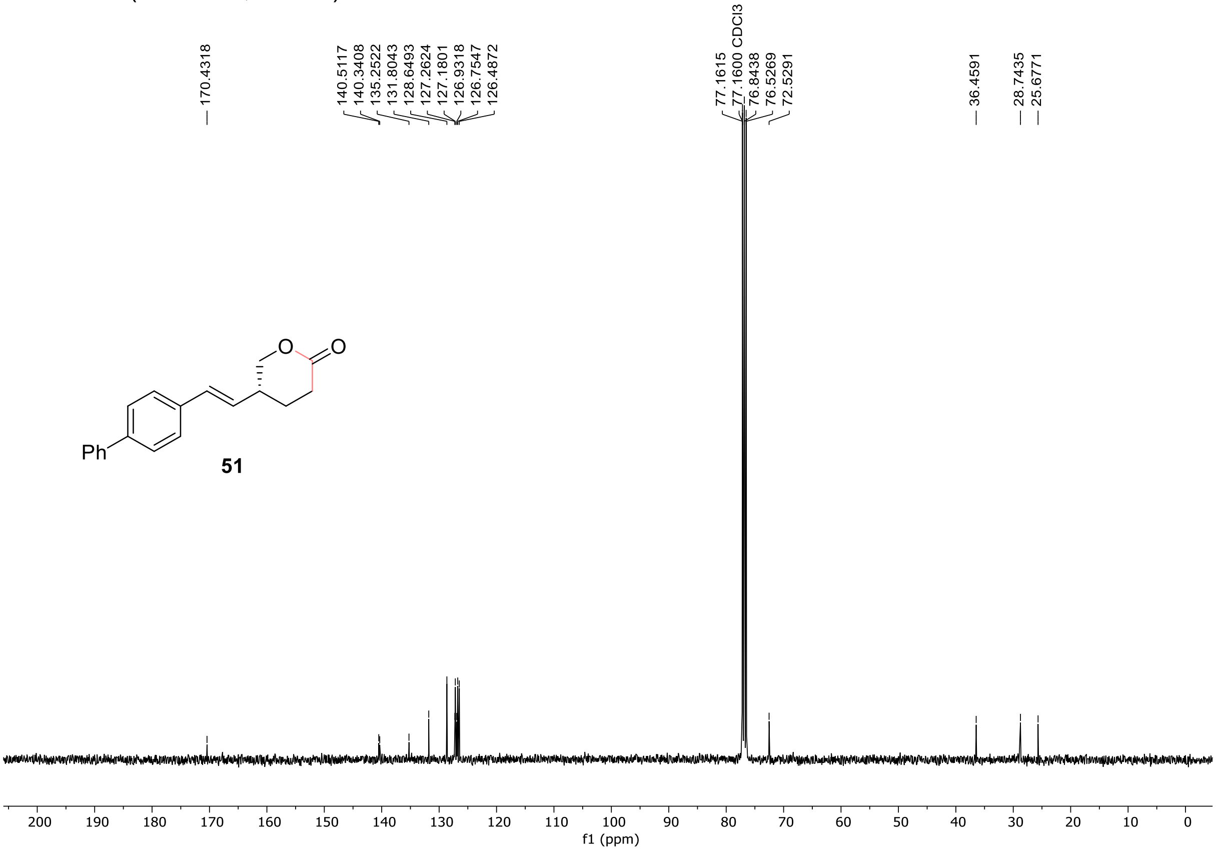

^{1}H NMR (400 MHz, $CDCl_3$) of **52** (*see NMR data*)

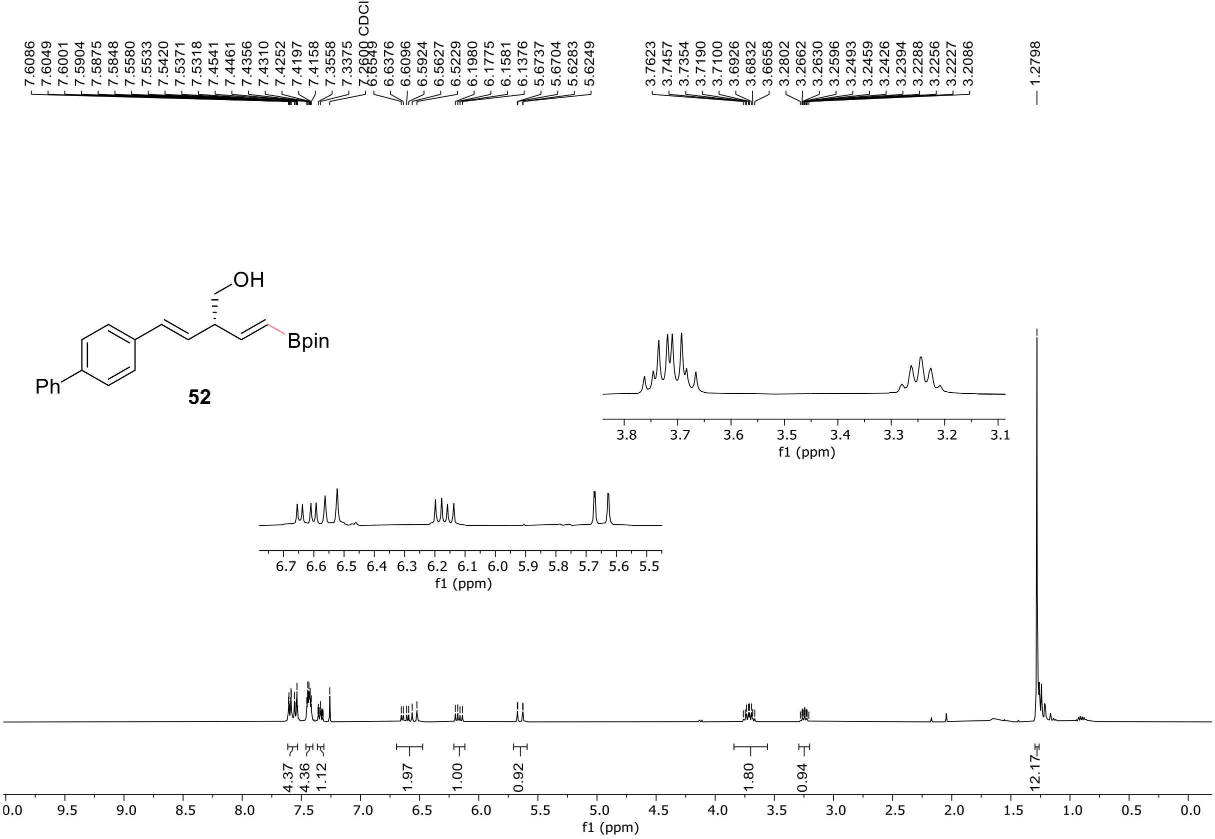

^{13}C NMR (101 MHz, $CDCl_3$) of **52**

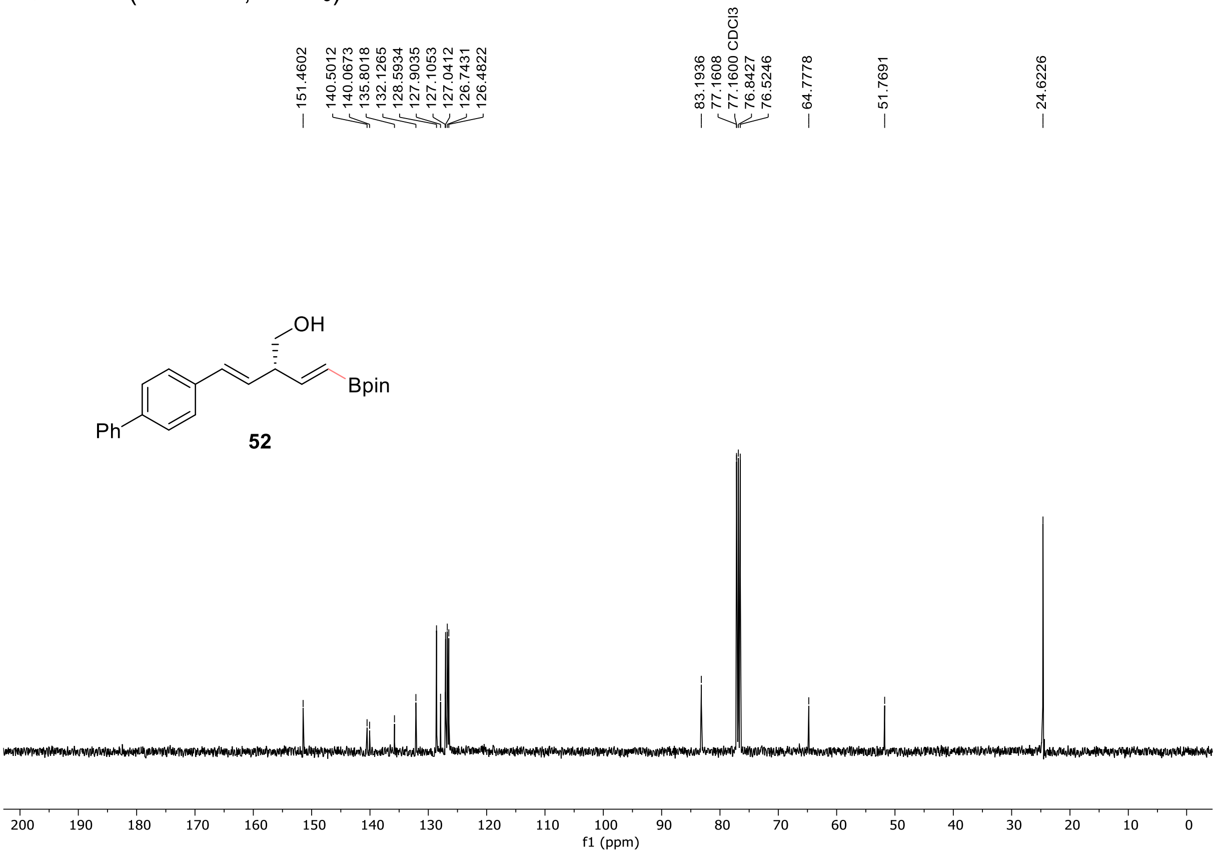

^{1}H NMR (400 MHz, $CDCl_3$) of **54** (*see NMR data*)

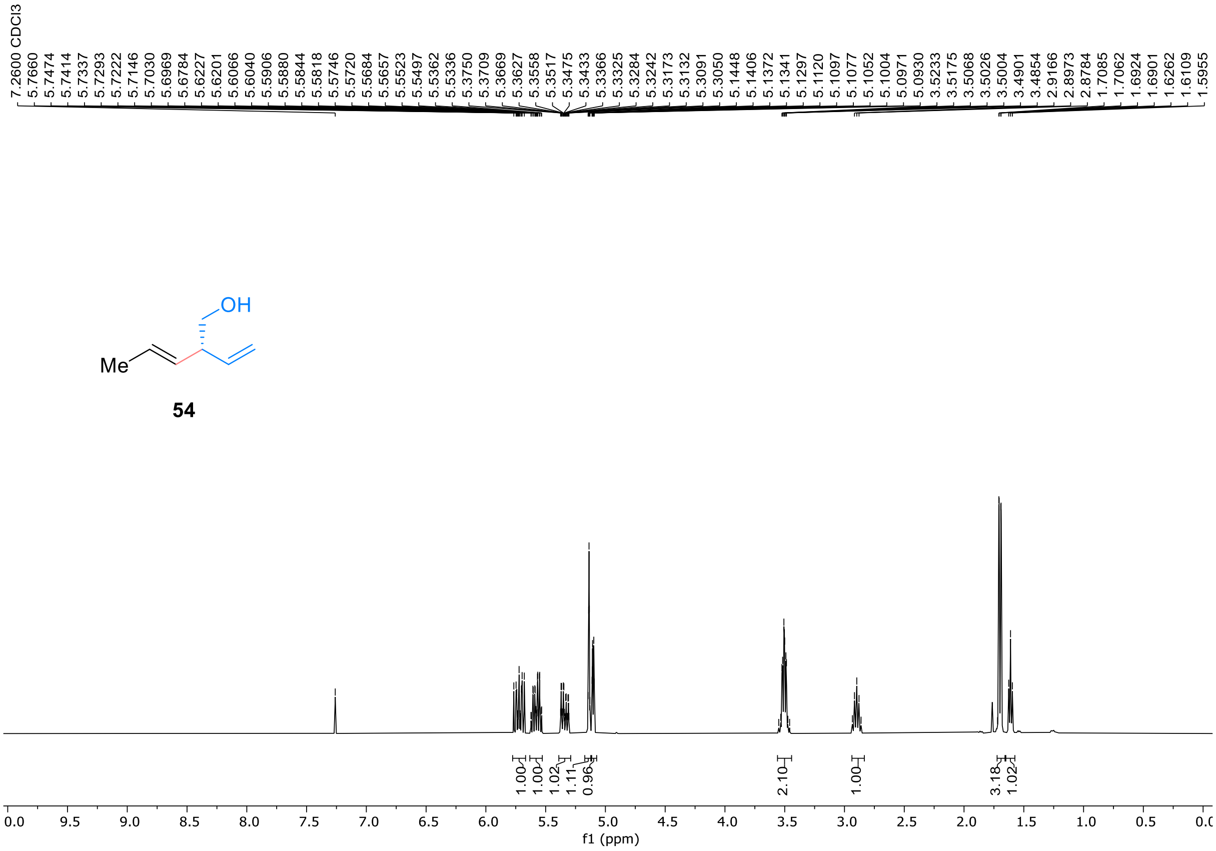

^{13}C NMR (101 MHz, $CDCl_3$) of **54**

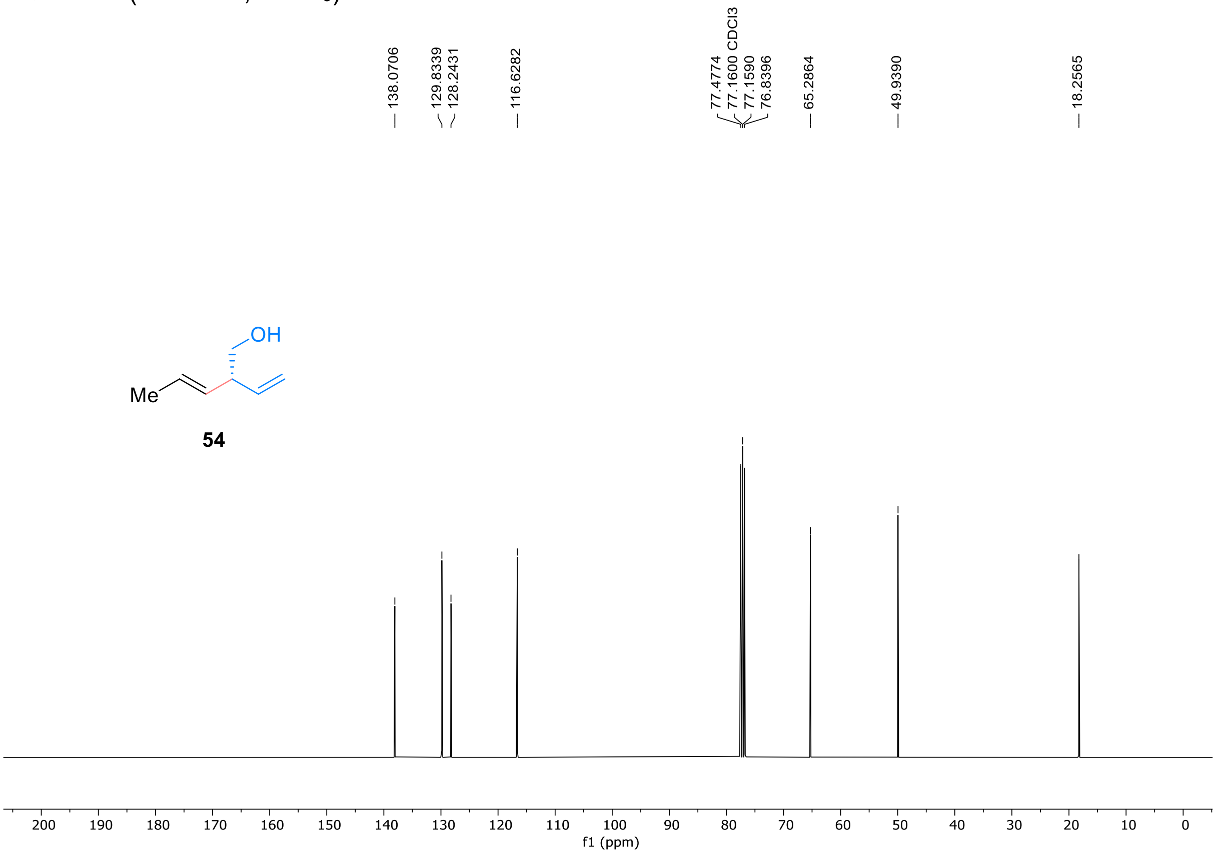

^{1}H NMR (500 MHz, $CDCl_3$) of **56** (*see NMR data*)

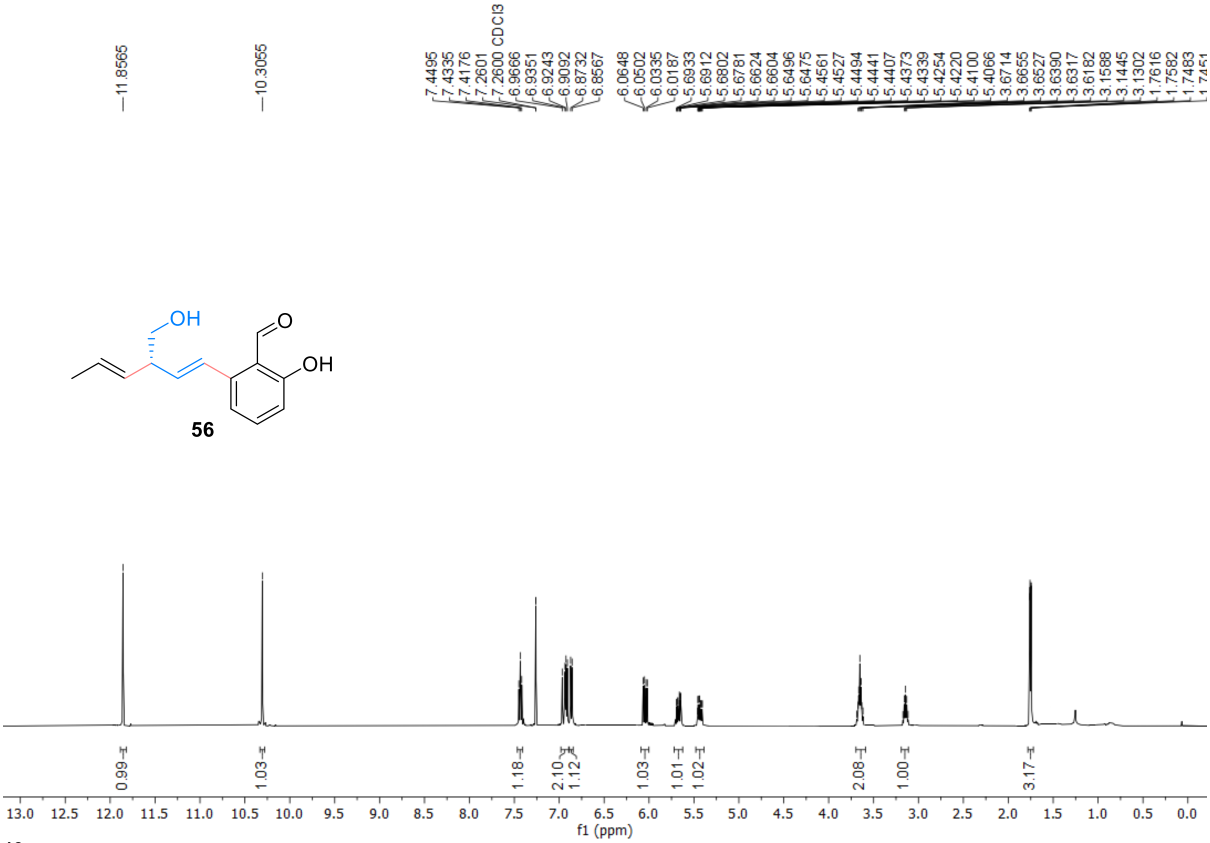

^{13}C NMR (126 MHz, $CDCl_3$) of **56**

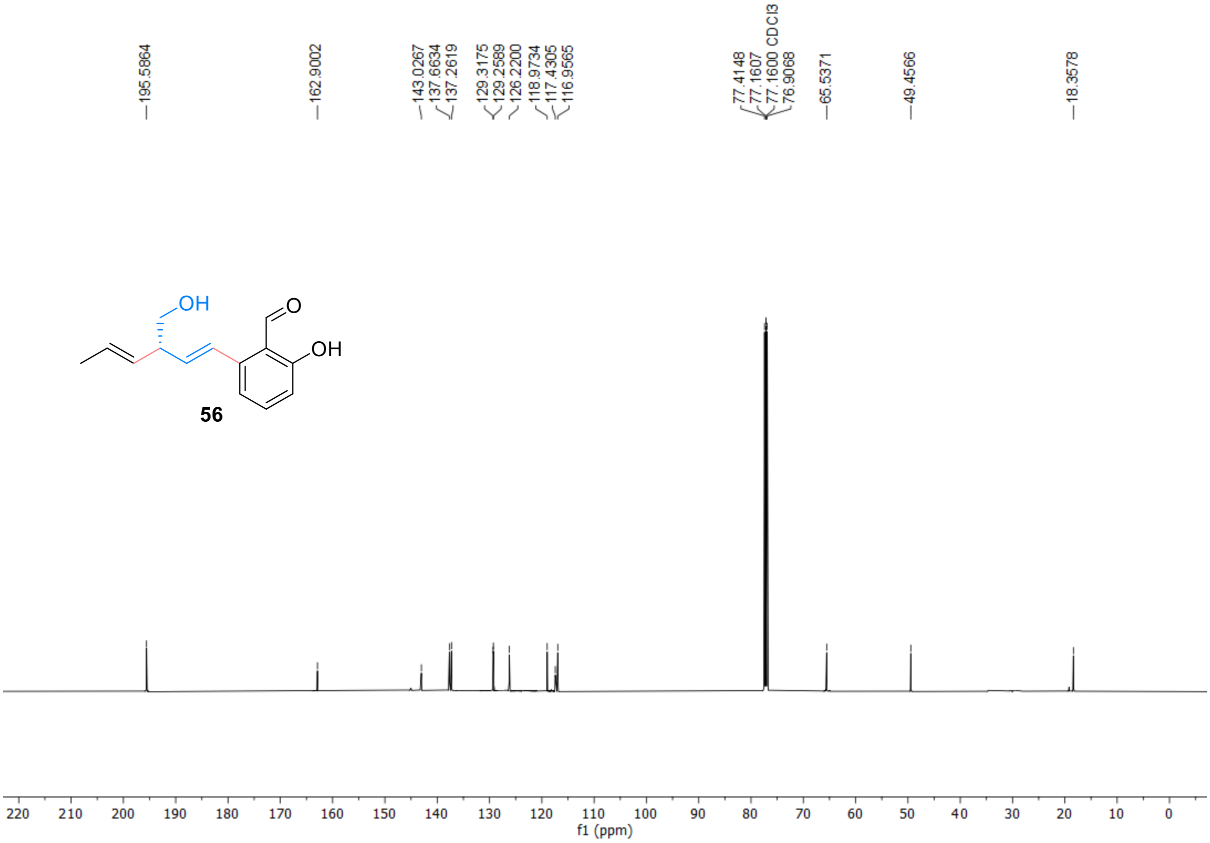

Supplement: Supplementary file 1 [file ja5c10680_si_001.pdf]
